# Anomeric DNA: Functionalization of α‐d Anomers of 7‐Deaza‐2′‐deoxyadenosine and 2′‐Deoxyuridine with Clickable Side Chains and Click Adducts in Homochiral and Heterochiral Double Helices

**DOI:** 10.1002/chem.202103872

**Published:** 2022-01-14

**Authors:** Aigui Zhang, Peter Leonard, Frank Seela

**Affiliations:** ^1^ Laboratory of Bioorganic Chemistry and Chemical Biology Center for Nanotechnology Heisenbergstrasse 11 48149 Münster Germany; ^2^ Laboratorium für Organische und Bioorganische Chemie Institut für Chemie neuer Materialien Universität Osnabrück Barbarastrasse 7 49069 Osnabrück Germany

**Keywords:** anomeric DNA, chirality, click chemistry, hybridization, oligonucleotides

## Abstract

Anomeric base pairs in heterochiral DNA with strands in the α‐d and β‐d configurations and homochiral DNA with both strands in α‐d configuration were functionalized. The α‐d anomers of 2′‐deoxyuridine and 7‐deaza‐2′‐deoxyadenosine were synthesized and functionalized with clickable octadiynyl side chains. Nucleosides were protected and converted to phosphoramidites. Solid‐phase synthesis furnished 12‐mer oligonucleotides, which were hybridized. Pyrene click adducts display fluorescence, a few of them with excimer emission. *T*_m_ values and thermodynamic data revealed the following order of duplex stability α/α‐d≫β/β‐d≥α/β‐d. CD spectra disclosed that conformational changes occur during hybridization. Functionalized DNAs were modeled and energy minimized. Clickable side chains and bulky click adducts are well accommodated in the grooves of anomeric DNA. The investigation shows for the first time that anomeric DNAs can be functionalized in the same way as canonical DNA for potential applications in nucleic acid chemistry, chemical biology, and DNA material science.

## Graphical abstract

**The impact of clickable** side chains and click adducts on heterochiral DNA with complementary strands in the α‐d and β‐d configurations and their homochiral counterparts with both strands in the α‐d or β‐d configuration is investigated. For this, the α‐anomers of 2′‐deoxyuridine and 7‐deaza‐2′‐deoxyadenosine were synthesized, converted to phosphoramidites, and employed in solid‐phase oligonucleotide synthesis. Functionalized α/α DNAs are much more stable than their α/β and β/β counterparts. This finding expands the toolbox of nucleic acid applications beyond canonical DNA.

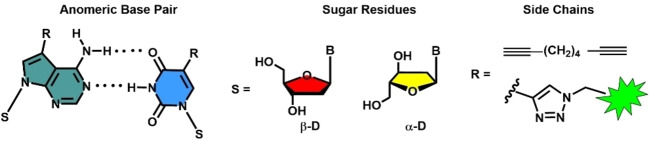

## Introduction

Anomeric DNA is formed when one strand of a duplex is in the α‐d and the other in the β‐d‐configuration.[^1^, ^2^] When DNA is constructed from two strands in the α‐d configuration, homochiral α/α‐DNA is generated; the anomeric counterpart to canonical DNA. The strand orientation in heterochiral α/β‐DNA is parallel, whereas homochiral α/α‐DNA displays an antiparallel alignment.^[1d]^ Here, the terms homochiral and heterochiral correspond solely to the stereochemistry at the anomeric center and not to d/l configuration. Recently, 2‐aminoadenine and the related 8‐aza‐7‐deazaadenine nucleobases (purine numbering is used throughout the results and discussion section) were used as replacements of the adenine moiety in an adenine–thymine pair. As a result, a significant stabilization of modified duplexes over that with canonical bases was observed. Also, the capability of anomeric nucleosides to form silver‐mediated base pairs was demonstrated on the basis of an anomeric α‐d/β‐d dC‐dC metal base pair.^[3]^

Herein, we report on the functionalization of the 2′‐deoxyadenosine‐2′‐deoxythymidine base pair. The 2′‐deoxyadenosine moiety was replaced by the α‐d anomer of 7‐deaza‐2′‐deoxyadenosine and the 2′‐deoxythymidine residue was substituted by anomeric 2′‐deoxyuridine moieties. Both nucleosides were functionalized; the 7‐deazapurine base at the 7‐position and the pyrimidine base at the 5‐position. Modifications were performed at one site of the base pair and double modifications at both sites. We anticipated that the positions of functionalization occurring in the major groove of canonical DNA will be also suitable for anomeric DNA and bulky residues might be well accommodated in anomeric double helices.

**Figure 1:**
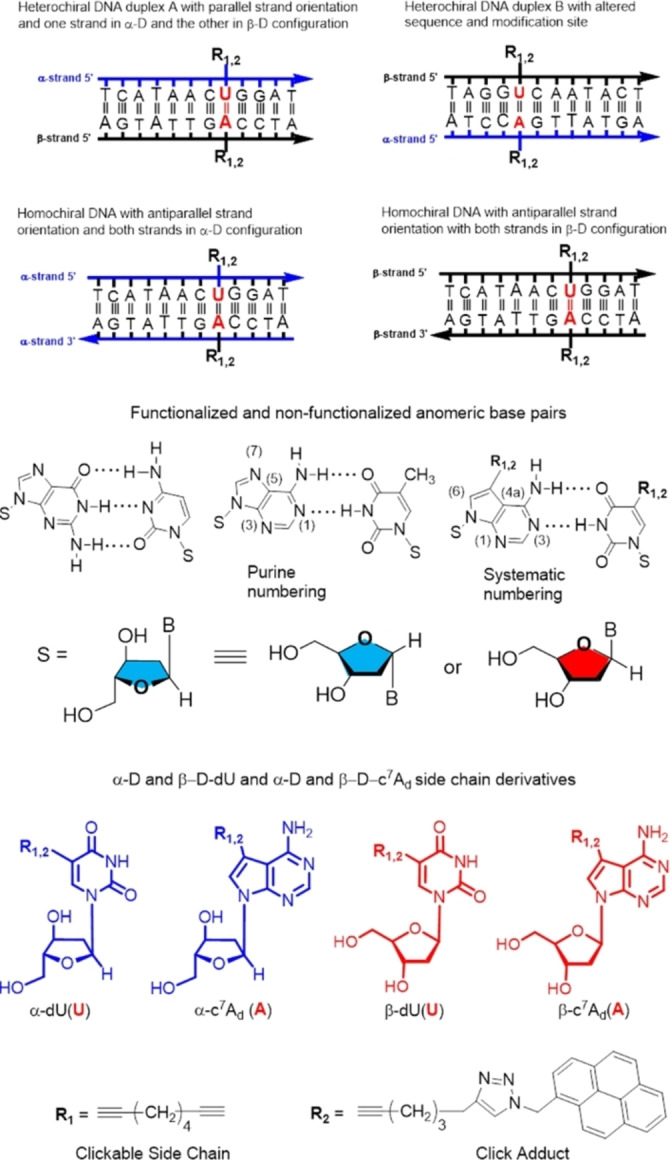
Schematic view of duplex structures with oligonucleotide strands in the α/β‐d, α/α‐d and β/β‐d configurations and with α/β‐d base pairs. Anomeric 5‐substituted 2′‐deoxyuridines and 7‐substituted 7‐deaza‐2′‐deoxyadenosines with clickable side chains and click adducts used in this study.

Octadiynyl residues with terminal triple bonds were introduced in the α‐d anomer of 7‐deaza‐2′‐deoxyadenosine (c^7^A_d_) and 2′‐deoxyuridine (dU) as they can be clicked to almost any other azide by the Huisgen‐Meldal‐Sharpless cycloaddition. Bulky pyrene azide was employed to form click adducts. To this end, nucleosides **2** and **9** were protected and converted to phosphoramidites and 12‐mer oligonucleotides were synthesized. The synthesis of the oligonucleotides with α‐d configuration made use of phosphoramidites of the α‐d anomers of 7‐deaza‐2′‐deoxyadenosine **12** and 2′‐deoxyuridine **4** together with those of the four α‐d nucleoside phosphoramidites with canonical bases. Then, strands were hybridized in various combinations to form heterochiral and homochiral duplexes (Figure 1). Within the anomeric strands, single incorporations of base‐modified α‐nucleosides were performed at the purine or the pyrimidine site and double modifications on both sites. Finally, copper(I)‐catalyzed click reactions were executed and bulky pyrene substituents were introduced. In addition, the sequential order of heterochiral DNAs was altered to evaluate sequence dependencies between anomeric and canonical DNAs.

With duplexes in hand, temperature‐dependent melting profiles were recorded and *T*_m_ values and thermodynamic data were calculated. CD‐spectra were measured to detect global helical changes. Fluorescence studies with DNA pyrene adducts gave information on DNA dye conjugates. Finally, AMBER force field energy minimization were undertaken on the functionalized DNA. These studies visualize the size of bulky substituents and the available space of the grooves.

## Results and Discussion

### Synthesis and characterization of α‐d anomers of 5‐octadiynyl‐2′‐deoxyuridine (2) and 7‐octadiynyl‐7‐deaza‐2′‐deoxyadenosine (9), conversion to phosphoramidites and pyrene click adducts

**Scheme 1:**
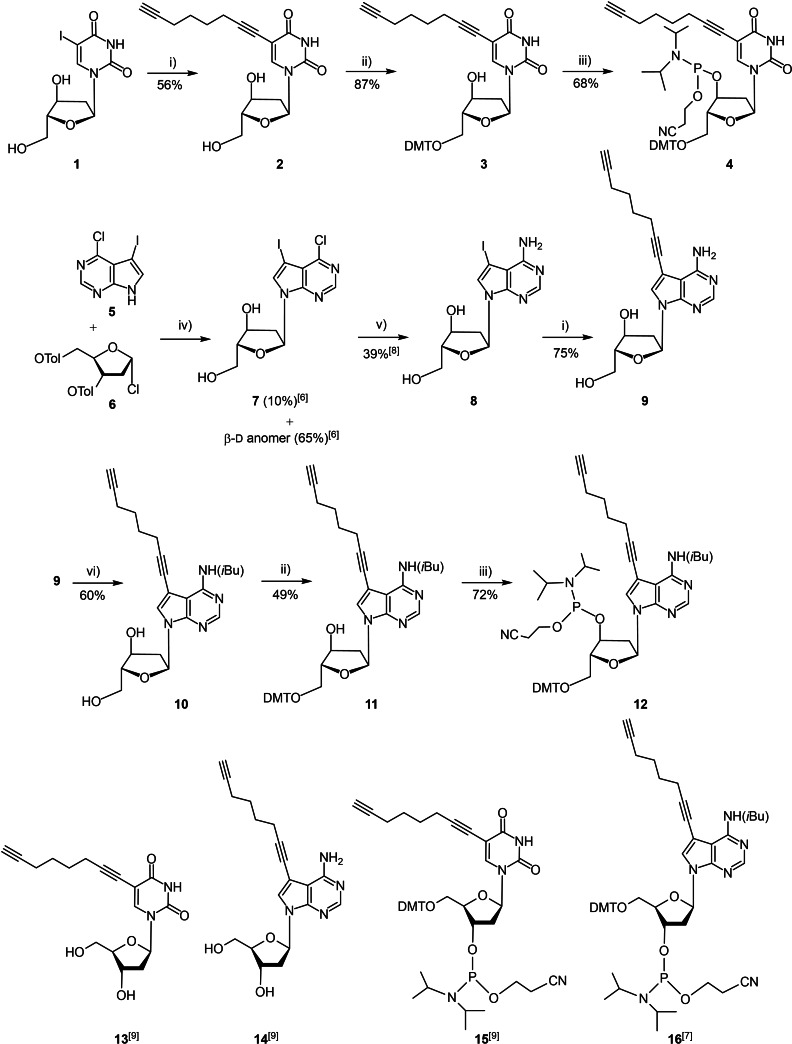
Top: Synthesis of the phosphoramidites **4** and **12** derived from 5‐octadiynyl‐α‐d‐2′‐deoxyuridine (**2**) and 7‐octadiynyl‐α‐d‐7‐deaza‐2′‐deoxyadenosine (**9**). i) CuI, Pd(PPh_3_)_4_, triethylamine, octa‐1,7‐diyne; ii) DMT−Cl, pyridine, RT, 3 h; iii) NC(OCH_2_)_2_P(Cl)N(*i*Pr)_2_, (*i*Pr)_2_NEt, RT; iv) KOH, TDA‐1, CH_3_CN, RT; v) 28 % aq. NH_3_/dioxane (2 : 1) 130 °C, 16 h; vi) 1: TMSiCl, pyridine, 30 min; 2: *i*Bu_2_O, 3 h, RT; 3: 14 % aq. NH_3_ ⋅  H_2_O, 30 min, RT. Bottom: Corresponding β‐d nucleosides and phosphoramidites reported earlier.[^7^, ^9^]

The α‐d iodonucleoside **1** ^[4]^ was chosen as starting material for the synthesis of the 5‐octadiynyl‐α‐d 2′‐deoxyuridine (**2**; Scheme 1). The octadiynyl side chain was introduced by Sonogashira cross‐coupling (Pd^0^/CuI) employing an excess of octa‐1,7‐diyne to assure mono‐functionalization. By this, the 5‐octadiynylated α‐d pyrimidine nucleoside **2** was isolated in 56 % yield after chromatographical purification. Protection of the 5′‐OH using DMT‐Cl afforded the 5′‐protected nucleoside **3** (87 % yield). Treatment of **3** with 2‐cyanoethyl diisopropylphosphoramidochloridite gave phosphoramidite **4** in 68 % yield. For the synthesis of 7‐octadiynylated α‐d‐7‐deaza‐2′‐deoxyadenosine (**9**) a similar route was employed. Nucleobase anion glycosylation of 6‐chloro‐7‐iodo‐7‐deazapurine (**5**) with Hoffers halogenose **6** ^[5]^ furnished the monomeric iodo nucleoside **7** and its β‐d anomer. Usually, anion glycosylation is stereoselective for the β‐d anomer but in this particular case (6‐chloro‐7‐iodo base **5**) the α‐d anomer **7** is formed as a side product in 10 % yield together with the β‐d anomer in 65 %.^[6]^ The anomeric mixture can be easily separated by flash column chromatography to give the pure anomers. For the β‐d anomer **14** the synthetic route to access the phosphoramidite **16** has been described,^[7]^ whereas the phosphoramidite of the α‐d anomer **9** is unknown. Consequently, 7‐iodinated α‐d‐7‐deaza‐2′‐deoxyadenosine (**8**) which was obtained from **7** according to a literature protocol^[8]^ was used in the Sonogashira cross‐coupling together with octa‐1,7‐diyne and Pd^0^/CuI as catalysts. By this, the 7‐octadiynyl‐α‐d‐7‐deaza‐2′‐deoxyadenosine **9** was obtained in 75 %. Then, octadiynylated **9** was protected at the amino group with an isobutyryl residue under conditions of transient protection giving the isobutyrylated compound **10** in 60 % yield. Tritylation with DMT‐Cl afforded the DMT derivative **11** (49 %) and phosphitylation at 3′‐OH gave the phosphoramidite **12** in 72 % yield. The syntheses of the corresponding β‐d nucleosides **13** ^[9]^ and **14** ^[9]^ and their phosphoramidites **15** ^[9]^ and **16** ^[7]^ have been described earlier by our laboratory.

**Scheme 2:**
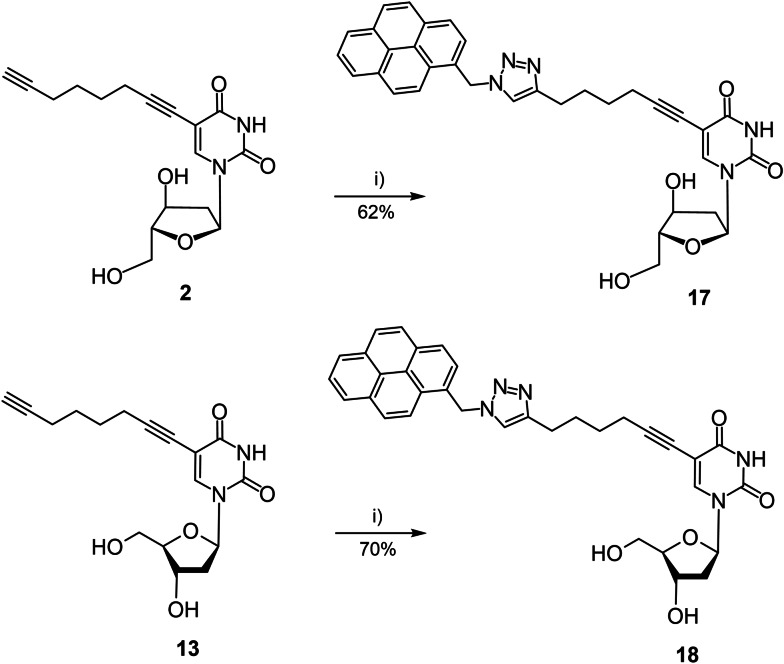
Synthesis of pyrene click adducts **17** and **18** from α‐d and β‐d 2′‐deoxyuridines **2** and **13**. i) Pyrene methyl azide, CuSO_4_ ⋅ 5 H_2_O, sodium ascorbate, and *t*BuOH/THF/H_2_O (3 : 1 : 1).

Next, the pyrene functionalized α‐dU and β‐dU nucleosides **17** and **18** were synthesized by using the Huisgen‐Meldal‐Sharpless click reaction (Scheme 2).[^10^, ^11^] For this, the 5‐octadiynylated compounds **2** and **13** were treated with an excess of pyrene azide in the presence of Cu^II^ sulfate pentahydrate and ascorbic acid as reducing agent in THF/*t*BuOH/H_2_O at RT overnight. By this, the functionalized nucleosides **17** and **18** were obtained in 62 % (α‐d) and 70 % (β‐d) yield. All new synthesized compounds were characterized by ^1^H, ^13^C NMR spectra as well as ESI‐TOF mass spectra. The ^1^H,^13^C correlated (HMBC and HSQC) NMR spectra were used to assign the ^13^C NMR signals (Tables S1 and S2 in the Supporting Information). Then, fluorescence spectra were recorded in various solvents to determine solvent dependent changes (Figure S7A and B). Both anomeric dU click conjugates show almost identical spectra and the same solvent dependence. Fluorescence was high in DMSO but was low in water. Monomer emission occurred in all cases and the compounds did not show excimer emission.

### Duplex stability, hypochromicity and thermodynamic data of click functionalized DNA with strands in α‐d/β‐d and β‐d/β‐d configuration

In the first part of this section, the thermal stabilities of functionalized heterochiral 12‐mer duplexes are explored and compared to their homochiral counterparts. To this end, dA or dT residues in a single dA–dT base pair were replaced by the anomeric dU nucleosides **2** and **13** or the anomeric 7‐deaza‐2′‐deoxyadenosine derivatives **9** and **14**. Furthermore, both nucleoside residues of the dA–dT base pair were substituted. Modifications were performed near the center of the duplexes. Six modified building blocks were required for the synthesis of 12‐mer oligonucleotide strands all in α‐d configuration. The same number of β‐nucleoside building blocks is necessary to access the β‐strands. The purity of all oligonucleotides was confirmed by RP‐18 HPLC (Figure S3) and MALDI‐TOF mass spectrometry (Table 1). Two different series of 12‐mer heterochiral duplexes were studied: i) α‐5′‐d(TCA TAA CTG GAT) (ODN‐**1**) ⋅ β‐5′‐d(AGT ATT GAC CTA) (ODN‐**11**; Table 2, below) and ii) β‐5′‐d(TAG GTC AAT ACT) (ODN‐**10**) ⋅ α‐5′‐d(ATC CAG TTA TGA) (ODN‐**5**; Table 3, below). The number of base pairs is identical but display different sequences. They were compared to their homochiral counterparts iii) β‐5′‐d(TCA TAA CTG GAT) (ODN‐**12**) ⋅ β‐3′‐d(AGT ATT GAC CTA) (ODN‐**13**; Table 2, below) and iv) β‐5′‐d(TAG GTC AAT ACT) (ODN‐**10**) ⋅ β‐5′‐d(ATC CAG TTA TGA) (ODN‐**11**; Table 3, below).

**Table 1:** Synthesized oligonucleotides and their molecular masses determined by MALDI‐TOF mass spectrometry.

	Oligonucleotide	M.W. (calcd.^[a]^/exp.^[b]^)		Oligonucleotide	M.W. (calcd.^[a]^/exp.^[b]^)
ODN‐**1**	α‐5′‐d(TCA TAA CTG GAT)	3644.4/3644.0	ODN‐**12**	β‐5′‐d(TCA TAA CTG GAT)	3644.4/3644.4
ODN‐**2**	α‐5′‐d(TCA TAA C **2** G GAT)	3734.5/3734.5	ODN‐**13**	β‐5′‐d(ATC CAG TTA TGA)	3644.4/3643.4
ODN‐**3**	α‐5′‐d(TCA TAA C **17** G GAT)	3991.8/3990.5	ODN‐**14**	β‐5′‐d(TAG G **13** C AAT ACT)	3734.5/3734.0
ODN‐**4**	α‐5′‐d(TAG GTC AAT ACT)	3644.4/3644.4	ODN‐**15**	β‐5′‐d(AGT ATT G **14** C CTA)	3747.6/3745.7
ODN‐**5**	α‐5′‐d(ATC CAG TTA TGA)	3644.4/3643.5	ODN‐**16**	β‐5′‐d(AGT ATT G **20** C CTA)	4004.9/4003.7
ODN‐**6**	α‐5′‐d(AGT ATT GAC CTA)	3644.4/3644.1	ODN‐**17**	β‐5′‐d(TAG G **18** C AAT ACT)	3991.8/3990.7
ODN‐**7**	α‐5′‐d(ATC C **9** G TTA TGA)	3747.6/3747.1	ODN‐**18**	β‐5′‐d(CGC GAA TTC GCG)	3646.4/3646.1
ODN‐**8**	α‐5′‐d(ATC C **19** G TTA TGA)	4004.9/4005.7	ODN‐**19**	β‐5′‐d(TCA TAA C **13** G GAT)	3734.5/3733.4
ODN‐**9**	α‐5′‐d(CGC GAA TTC GCG)	3646.4/3647.5	ODN‐**20**	β‐5′‐d(ATC C **14** G TTA TGA)	3747.6/3747.2
ODN‐**10**	β‐5′‐d(TAG GTC AAT ACT)	3643.6/3643.7	ODN‐**21**	β‐5′‐d(TCA TAA C **18** G GAT)	3992.8/3992.0
ODN‐**11**	β‐5′‐d(AGT ATT GAC CTA)	3643.6/3643.7	ODN‐**22**	β‐5′‐d(ATC C **20** G TTA TGA)	4004.9/4005.3
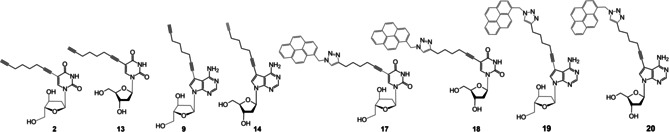					

[a] Calculated on the basis of the molecular mass of [*M*+H]^+^. [b] Determined by MALDI‐TOF mass‐spectrometry as [*M*+H] ^+^ in the linear positive mode.

**Table 2:** *T*_m_ values and thermodynamic data for antiparallel‐ and parallel‐strand duplexes containing α‐5‐octadiynyl‐dU **2**, β‐5‐octadiynyl‐dU **13** and β‐7‐octadiynyl‐c^7^A_d_ **14** and pyrene click conjugates.^[a]^

Heterochiral (α/β) duplexes	*T*_m_^[b]^ [°C]/H [%]^[c]^	Δ*H*° [kcal mol^−1^]	Δ*S*° [cal mol^−1^ K^−1^]	Δ*G*°_310_ [kcal/mol]	Homochiral (β/β) duplexes	*T*_m_^[b]^ [°C]/H [%]^[c]^	Δ*H*° [kcal mol^−1^]	Δ*S*° [cal mol^−1^ K^−1^]	Δ*G*°_310_ [kcal mol^−1^]
Parallel strands					Antiparallel strands				
α‐dU functionalization					β‐dU functionalization				
α‐5′‐d(TCA TAA C **T** G GAT) (ODN‐**1**) β‐5′‐d(AGT ATT G **A** C CTA) (ODN‐**11**)	41/21	−66	−183	−9.5	β‐5′‐d(TCA TAA C **T** G GAT) (ODN‐**12**) β‐3′‐d(AGT ATT G **A** C CTA) (ODN‐**13**)	45/20	−80	−223	−10.3
α‐5′‐d(TCA TAA C **2** G GAT) (ODN‐**2**) β‐5′‐d(AGT ATT G **A** C CTA) (ODN‐**11**)	40/19	−65	−180	−9.0	β‐5′‐d(TCA TAA C **13** G GAT) (ODN‐**19**) β‐3′‐d(AGT ATT G **A** C CTA) (ODN‐**13**)	46/19	−81	−227	−10.8
α‐5′‐d(TCA TAA C **17** G GAT) (ODN‐**3**) β‐5′‐d(AGT ATT G **A** C CTA) (ODN‐**11**)	42^[d]/^17	−57^[d]^	−151^[d]^	−9.8^[d]^	β‐5′‐d(TCA TAA C **18** G GAT) (ODN‐**21**) β‐3′‐d(AGT ATT G **A** C CTA) (ODN‐**13**)	48^[d]^/14	−77^[d]^	−209^[d]^	−11.6^[d]^
β‐c^7^A_d_ functionalization					β‐c^7^A_d_ functionalization				
α‐5′‐d(TCA TAA C **T** G GAT) (ODN‐**1**) β‐5′‐d(AGT ATT G **14** C CTA) (ODN‐**15**)	38/19	−56	−153	−8.6	β‐5′‐d(TCA TAA C **T** G GAT) (ODN‐**12**) β‐3′‐d(AGT ATT G **14** C CTA) (ODN‐**20**)	43/18	−74	−208	−9.8
α‐5′‐d(TCA TAA C **T** G GAT) (ODN‐**1**) β‐5′‐d(AGT ATT G **20** C CTA) (ODN‐**16**)	45^[d]^/17	−59^[d]^	−157^[d]^	−10.5^[d]^	β‐5′‐d(TCA TAA C **T** G GAT) (ODN‐**12**) β‐3′‐d(AGT ATT G **20** C CTA) (ODN‐**22**)	47^[d]^/14	−67^[d]^	−182^[d]^	−11.0^[d]^

Double functionalization					Double functionalization				
α‐5′‐d(TCA TAA C **2** G GAT) (ODN‐**2**) β‐5′‐d(AGT ATT G **14** C CTA) (ODN‐**15**)	37/19	−59	−163	−8.4	β‐5′‐d(TCA TAA C **13** G GAT) (ODN‐**19**) β‐3′‐d(AGT ATT G **14** C CTA) (ODN‐**20**)	45/19	−77	−214	−10.3
α‐5′‐d(TCA TAA C **17** G GAT) (ODN‐**3**) β‐5′‐d(AGT ATT G **14** C CTA) (ODN‐**15**)	41^[d]^/15	−52^[d]^	−140^[d]^	−8.2^[d]^	β‐5′‐d(TCA TAA C **18** G GAT) (ODN‐**21**) β‐3′‐d(AGT ATT G **14** C CTA) (ODN‐**20**)	49^[d]^/14	−73^[d]^	−198^[d]^	−11.8^[d]^
α‐5′‐d(TCA TAA C **2** G GAT) (ODN‐**2**) β‐5′‐d(AGT ATT G **20** C CTA) (ODN‐**16**)	45^[d]^/16	−56^[d]^	−148^[d]^	−10.4^[d]^	β‐5′‐d(TCA TAA C **13** G GAT) (ODN‐**19**) β‐3′‐d(AGT ATT G **20** C CTA) (ODN‐**22**)	50^[d]^/14	−67^[d]^	−179^[d]^	−11.7^[d]^
α‐5′‐d(TCA TAA C **17** G GAT) (ODN‐**3**) β‐5′‐d(AGT ATT G **20** C CTA) (ODN‐**16**)	47^[d]^/17	−49^[d]^	−123^[d]^	−10.5^[d]^	β‐5′‐d(TCA TAA C **18** G GAT) (ODN‐**21**) β‐3′‐d(AGT ATT G **20** C CTA) (ODN‐**22**)	52^[d]^/15	−64^[d]^	−169^[d]^	−11.9^[d]^
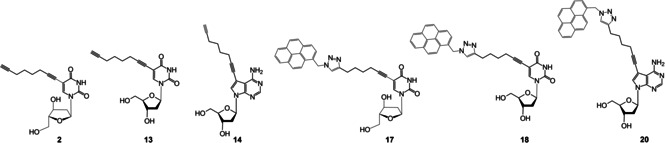									

[a] Measured at 260 nm at a concentration of 5 μM+5 μM single strand at a heating rate of 1.0 °C min^−1^ in 100 mM NaCl, 10 mM MgCl_2_, and 10 mM Na‐cacodylate (pH 7.0). [b] *T*_m_ values were calculated from the heating curves using the program Meltwin 3.0.^[12]^ [c] H=hypochromicity. [d] For duplexes containing pyrene click adducts, a concentration of 2 μM+2 μM single strand was used.

**Table 3:** *T*_m_ values and thermodynamic data for antiparallel‐ and parallel‐strand duplexes containing α‐7‐octadiynyl‐c^7^A_d_ **9**, β‐5‐octadiynyl‐dU **13**, β‐7‐octadiynyl‐c^7^A_d_ **14** and pyrene click conjugates.^[a]^

Heterochiral (α/β) duplexes	*T*_m_^[b]^ [°C]/H [%]^[c]^	Δ*H*° [kcal mol^−1^]	Δ*S*° [cal mol^−1^ K^−1^]	Δ*G*°_310_ [kcal mol^−1^]	Homochiral (β/β) duplexes	*T*_m_^[b]^ [°C]/H [%]^[c]^	Δ*H*° [kcal mol^−1^]	Δ*S*° [cal mol^−1^ K^−1^]	Δ*G*°_310_ [kcal mol^−1^]
Parallel strands					Antiparallel strands				
β‐dU functionalization					β‐dU functionalization				
β‐5′‐d(TAG G **T** C AAT ACT) (ODN‐**10**) α‐5′‐d(ATC C **A** G TTA TGA) (ODN‐**5**)	45/19 %	−69	−191	−10.1	β‐5′‐d(TAG G **T** C AAT ACT) (ODN‐**10**) β‐3′‐d(ATC C **A** G TTA TGA) (ODN‐**11**)	47**/**19	−81	−225	−10.9
β‐5′‐d(TAG G **13** C AAT ACT) (ODN‐**14**) α‐5′‐d(ATC C **A** G TTA TGA) (ODN‐**5**)	44/18 %	−69	−189	−9.9	β‐5′‐d(TAG G **13** C AAT ACT) (ODN‐**14**) β‐3′‐d( ATC C **A** G TTA TGA) (ODN‐**11**)	46**/**18	−82	−231	−10.7
β‐5′‐d(TAG G **18** C AAT ACT) (ODN‐**17**) α‐5′‐d(ATC C **A** G TTA TGA) (ODN‐**5**)	47^[d]^/15 %	−66^[d]^	−176^[d]^	−11.0^[d]^	β‐5′‐d(TAG G **18** C AAT ACT) (ODN‐**17**) β‐3′‐d(ATC C **A** G TTA TGA) (ODN‐**11**)	51^[d]^ **/**16	−76^[d]^	−207^[d]^	−12.2^[d]^

α‐c^7^A_d_ functionalization					β‐c^7^A_d_ functionalization				
β‐5′‐d(TAG G **T** C AAT ACT) (ODN‐**10**) α‐5′‐d(ATC C **9** G TTA TGA) (ODN‐**7**)	43/18 %	−70	−194	−9.7	β‐5′‐d(TAG G **T** C AAT ACT) (ODN‐**10**) β‐3′‐d(ATC C **14** G TTA TGA) (ODN‐**15**)	45**/**17	−80	−223	−10.4
β‐5′‐d(TAG G **T** C AAT ACT) (ODN‐**10**) α‐5′‐d(ATC C **19** G TTA TGA) (ODN‐**8**)	46^[d]^/17 %	−70^[d]^	−190^[d]^	−10.8^[d]^	β‐5′‐d(TAG G **T** C AAT ACT) (ODN‐**10**) β‐3′‐d(ATC C **20** G TTA TGA) (ODN‐**16**)	51^[d]^ **/**16	−71^[d]^	−192^[d]^	−12.0^[d]^

Double functionalization					Double functionalization				
β‐5′‐d(TAG G **13** C AAT ACT) (ODN‐**14**) α‐5′‐d(ATC C **9** G TTA TGA) (ODN‐**7**)	43/18 %	−69	−192	−9.7	β‐5′‐d(TAG G **13** C AAT ACT) (ODN‐**14**) β‐3′‐d(ATC C **14** G TTA TGA) (ODN‐**15**)	45**/**18	−78	−218	−10.2
β‐5′‐d(TAG G **18** C AAT ACT) (ODN‐**17**) α‐5′‐d(ATC C **9** G TTA TGA) (ODN‐**7**)	47^[d]^/16 %	−65^[d]^	−174^[d]^	−11.1^[d]^	β‐5′‐d(TAG G **18** C AAT ACT) (ODN‐**17**) β‐3′‐d(ATC C **14** G TTA TGA) (ODN‐**15**)	51^[d]^ **/**17	−64^[d]^	−172^[d]^	−10.3^[d]^
β‐5′‐d(TAG G **13** C AAT ACT) (ODN‐**14**) α‐5′‐d(ATC C **19** G TTA TGA) (ODN‐**8**)	47^[d]^/16 %	−66^[d]^	−178^[d]^	−11.0^[d]^	β‐5′‐d(TAG G **13** C AAT ACT) (ODN‐**14**) β‐3′‐d(ATC C **20** G TTA TGA) (ODN‐**16**)	52^[d]^ **/**17	−70^[d]^	−186^[d]^	−12.2^[d]^
β‐5′‐d(TAG G **18** C AAT ACT) (ODN‐**17**) α‐5′‐d(ATC C **19** G TTA TGA) (ODN‐**8**)	52^[d]^/18 %	−66^[d]^	−173^[d]^	−12.0^[d]^	β‐5′‐d(TAG G **18** C AAT ACT) (ODN‐**17**) β‐3′‐d(ATC C **20** G TTA TGA) (ODN‐**16**)	61^[d]^ **/**16	−60^[d]^	−151^[d]^	−13.2^[d]^
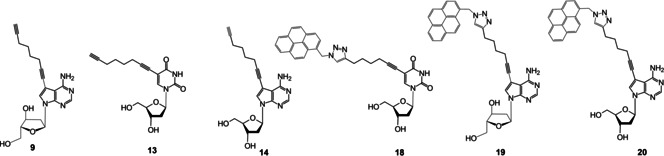									

[a] Measured at 260 nm at a concentration of 5 μM+5 μM single strand at a heating rate of 1.0 °C min^−1^ in 100 mM NaCl, 10 mM MgCl_2_, and 10 mM Na‐cacodylate (pH 7.0). [b] *T*_m_ values were calculated from the heating curves using the program Meltwin 3.0.^[12]^ [c] H=hypochromicity. [d] For duplexes containing pyrene click adducts, a concentration of 2 μM+2 μM single strand was used.

**Figure 2:**
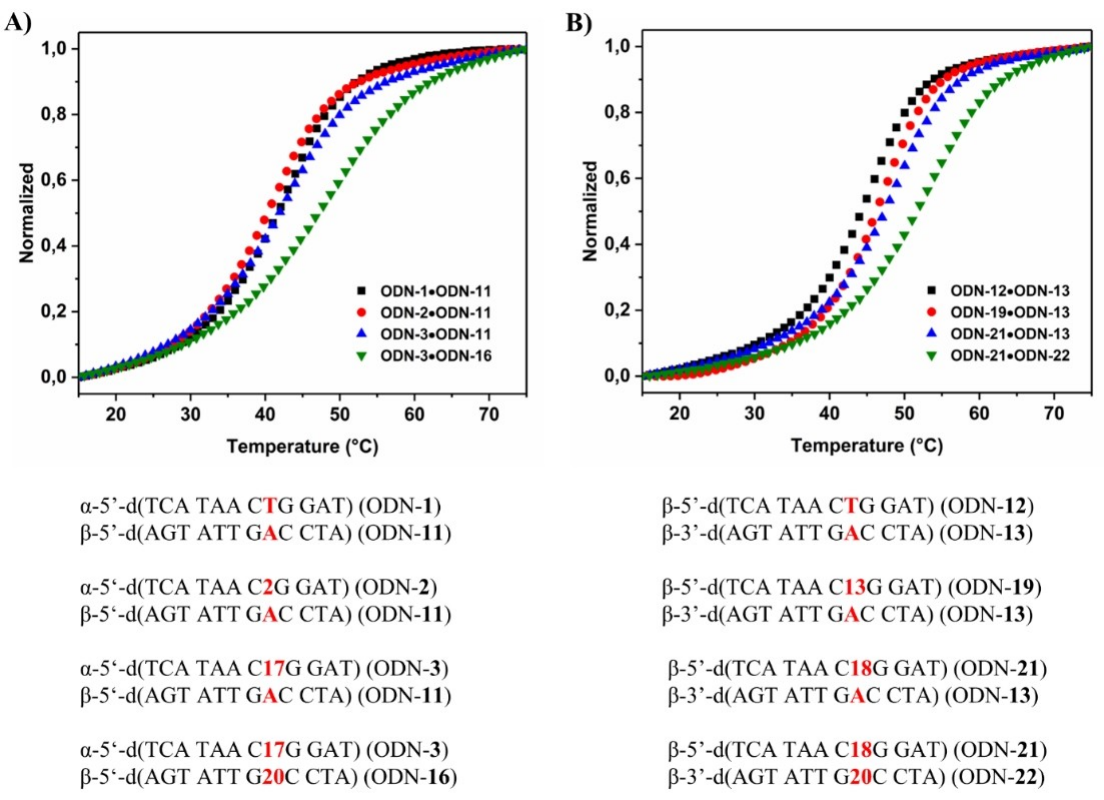
Melting curves of heterochiral parallel α/β‐d duplexes and the corresponding homochiral antiparallel β/β‐d ones measured at 260 nm at a concentration of 5 μM+5 μM single strand at a heating rate of 1.0 °C min^−1^ in 100 mM NaCl, 10 mM MgCl_2_, and 10 mM Na‐cacodylate (pH 7.0). For duplexes containing pyrene click adducts, a concentration of 2 μM+2 μM single strand was used.

Typical melting profiles are shown in Figure 2. All curves show cooperative melting. In Tables 2 and 3, *T*_m_ values and thermal hypochromicity data are summarized and duplex stability of α/β‐DNA is compared to β/β‐DNA.

According to the *T*_m_ data only small stability changes occur in homochiral DNAs, whereas significantly stronger changes are observed for heterochiral DNA. For heterochiral DNA, already the stabilities of the nonfunctionalized duplexes differ and the same effect is observed for duplexes with single or double modifications within the base pairs (Table 2 vs. Table 3). Apparently, heterochiral duplexes with parallel chain orientation are more sensitive to sequence changes than their homochiral antiparallel counterparts.

In more detail, when side chains were introduced in the 5‐position of dU or 7‐position of c^7^A_d_ in canonical homochiral β‐DNA (both series of duplexes, Tables 2 and 3) modifications have only a slight impact on the duplex stability with respect to the unmodified duplexes. For heterochiral duplexes incorporating dU or c^7^A_d_ side‐chain derivatives the *T*_m_ values are usually around 4 to 8 °C lower compared to homochiral DNA. This is valid for single or double modifications.

Furthermore, pyrene side chains contribute extra stability to both homo‐ and heterochiral duplexes. However, in homochiral DNA stabilization by pyrene residues is particularly strong which might be related to the intercalation of the pyrene moiety. Thermal hypochromicities are similar for heterochiral and homochiral duplexes and values between 15 to 20 % were measured in both series.

Next, thermodynamic data were determined. To this end, the program Meltwin 3.0^[12]^ was used and data were extracted from melting curve shape analyses. Δ*H*° values were higher for homochiral DNA duplexes with both strands in β‐d configuration with respect to heterochiral α/β DNA. Data indicate that loss of duplex stability might be due to altered stacking forces and/or weaker H‐bonding.^[13]^

The global helix conformation of duplex DNA can be monitored by CD spectra.[^14^, ^15^] Various factors contribute to the shape of the CD‐spectra: i) the conformation of the monomers which can be *syn* or *anti*, ii) the conformation of the sugar residues (*N* vs. *S*), iii) the hydrogen‐bonding network formed between nucleobases, iv) stacking interactions between nucleobases or base pairs and v) the helicity of the duplex (+ or −). Previous CD experiments have shown that CD spectra of α‐anomeric single strands display spectra with mirror‐like Cotton effects with respect to the β‐strand.[^1^, ^2^, ^3^] Complete mirror images are not expected as diastereoisomers are compared and not enantiomeric molecules with strands in d‐ and l‐configuration. The phenomenon has been already discussed for nonfunctionalized α‐d hexamer oligonucleotide duplexes with all‐purine or all‐pyrimidine bases in either the α‐ or the β‐strand.^[1b]^ Our DNA fragment represent a full helix turn that contains all four DNA bases in random composition with and without clickable side chains and click adducts.

**Figure 3:**
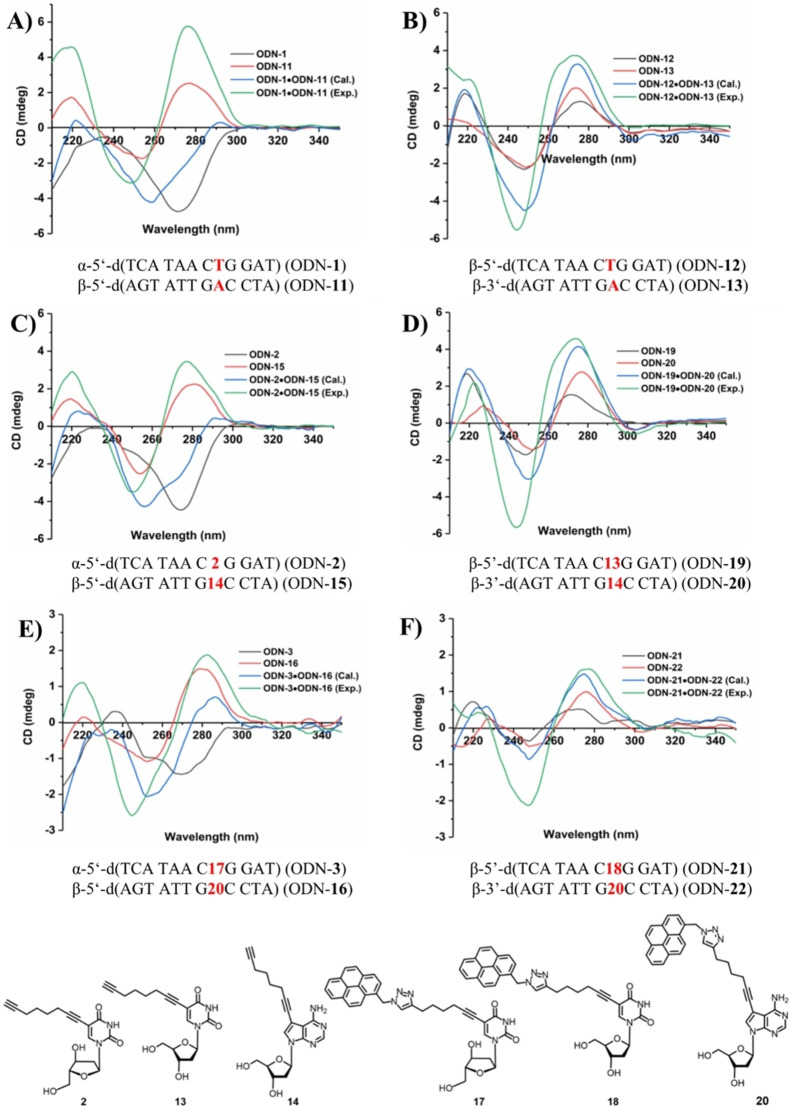
CD spectra of single‐ and double‐stranded oligonucleotides with α‐d and β‐d configurations. All measurements were performed at a concentration of 5 μM+5 μM single strand in 100 mM NaCl, 10 mM MgCl_2_, and 10 mM Na‐cacodylate, pH 7.0. The cell path length of the cuvette for the CD measurements was 5 mm. For pyrene click adducts, a 2 μM+2 μM single strand concentration was used. Black and red curves show the CD spectra of the single strands. Blue curves show the calculated spectra (sum of the CD spectra of the single strands). Green curves show the experimentally determined spectra.

According to Figure 3 remarkable strong negative Cotton effects are observed for the α‐strands. These negative CD signals around 280 nm disappear when the α‐strand is hybridized with the β‐strand to form a duplex. Now, the Cotton effect becomes positive and displays a similar shape as the β‐strand. These strong changes of the CD spectra are typical for all heterochiral DNA duplexes used in this study – modified or not. It shows that the strands adopt the conformation of the β‐strand and a strong conformational change occurs during hybridization. It has been already known for canonical DNA that single strands are more flexible than duplexes. Single strands show a persistence length of a few nanometers whereas double stranded helices form stiff rods with a persistence length of around 40 nm. Here, the β‐strand dictates the conformation of the final duplex. Duplex DNA is then much more difficult to bent as single stranded DNA. This was shown for canonical DNA but is now also anticipated for anomeric DNA.

Also, experimentally determined CD spectra of duplex DNAs were compared with calculated spectra (sum of the CD spectra of the single strands, Figure 3). The shape of CD curves of homochiral β/β duplexes from calculated and measured spectra is similar, whereas completely different spectra were obtained for the measured heterochiral α/β duplexes with respect to the calculated spectra. Measured α/β‐ duplexes exhibit a similar shape as homochiral β/β‐d duplexes, calculated spectra of α/β‐duplexes display curves similar to α‐d single strands. Apparently, upon duplex formation the β‐anomer seems to dictate the sign of CD spectra and therefore the structure of the heterochiral duplexes. This phenomenon is observed for all α/β duplexes with and without side chains. All data indicate that base pair overlaps and stacking interactions are similar in heterochiral (α/β) DNA with respect to homochiral (β/β) DNA. From the spectra of duplexes one can conclude that clickable side chains of moderate size (octadiynyl) and more space demanding residues (pyrene) are well accommodated in the grooves of heterochiral α/β DNA. Thus, data confirm that the position‐5 for pyrimidines and position‐7 for 7‐deazapurines are ideally suited for functionalization.

### Functionalized homochiral DNA with both side chains in α‐d configuration

**Figure 4:**
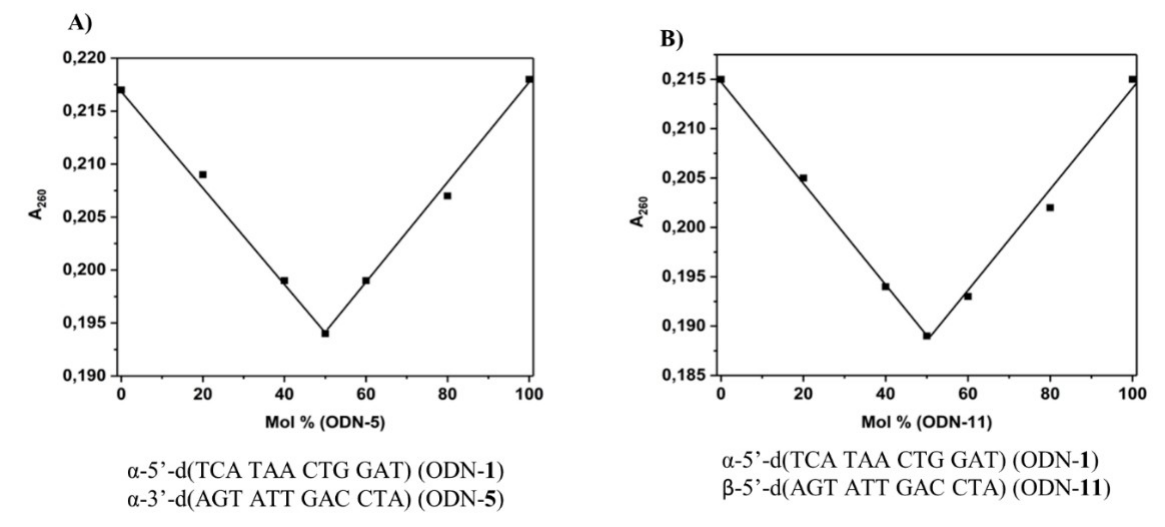
Mixing experiments of A) ODN‐**1** ⋅ ODN‐**5** and B) ODN‐**1** ⋅ ODN‐**11**. Experiments were performed at 260 nm at a single‐strand concentration of 2 μM+2 μM in 100 mM NaCl, 10 mM MgCl_2_, and 10 mM Na‐cacodylate (pH 7.0).

Next, duplexes were studied with both strands in an α‐d configuration.^[16]^ To determine the strand stoichiometry, so‐called mixing experiments of a series of homochiral duplexes were performed. For each synthetic duplex, a series of mixtures were prepared with varying ratios of oligonucleotide and a constant total oligonucleotide concentration. The absorbance of each mixture was measured three times at a wavelength of 260 nm resulting in a titration graph (Figures 4 and S2). According to Figure 4, all duplexes showed one to one strand stoichiometry confirming that only duplexes are formed and that the formation of other assemblies, for example triplexes, is excluded.

**Figure 5:**
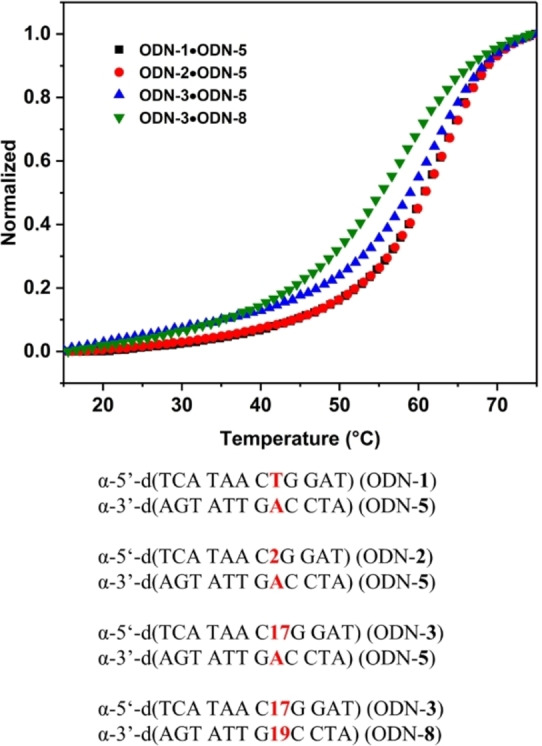
Thermal denaturation experiments of homochiral antiparallel‐strand α/α‐d duplexes. Measured at 260 nm at a concentration of 5 μM+5 μM single strand at a heating rate of 1.0 °C min^−1^ in 100 mM NaCl, 10 mM MgCl_2_, and 10 mM Na‐cacodylate (pH 7.0). For duplexes containing pyrene click adducts, a concentration of 2 μM+2 μM single strand was used.

Figure 5 shows melting curves for α/α‐d duplexes measured at 260 nm. According to this figure, all curves showed cooperative melting with high *T*_m_ values around 60 °C. From that thermodynamic data were calculated (Table 4). Single modification of a dT residue by the side‐chain derivative **2** shows a significantly higher *T*_m_ value than replacement of dA by the derivative **9**. On the contrary, the α‐anomeric dU click adduct **17** retains the *T*_m_ value of the nonfunctionalized duplex ODN‐**1** ⋅ ODN‐**5**. Apparently, side‐chain derivatives with α‐dU modification have no negative impact on the stability of homochiral α/α DNA.

**Table 4:** *T*_m_ values and thermodynamic data for antiparallel‐ and parallel‐strand duplexes containing α‐7‐octadiynyl‐c^7^A_d_ **9**, α‐5‐octadiynyl‐dU **2** and pyrene click conjugates.^[a]^

	*T*_m_^[b]^ [°C]/H [%]^[c]^	Δ*H*° [kcal mol^−1^]	Δ*S*° [cal mol^−1^ K^−1^]	Δ*G*°_310_ [kcal mol^−1^]
Homochiral (α/α) duplexes with antiparallel strands and single modifications				
α‐5′‐d(TCA TAA C **T** G GAT) (ODN‐**1**) α‐3′‐d(AGT ATT G **A** C CTA) (ODN‐**5**)	62/14	−118	−325	−17.0
α‐5′‐d(TCA TAA C **T** G GAT) (ODN‐**1**) α‐3′‐d(AGT ATT G **9** C CTA) (ODN‐**7**)	59/13	−94	−255	−14.7
α‐5′‐d(TCA TAA C **T** G GAT) (ODN‐**1**) α‐3′‐d(AGT ATT G **19** C CTA) (ODN‐**8**)	57^[d]^/14	−73^[d]^	−191^[d]^	−13.4^[d]^
α‐5′‐d(TCA TAA C **2** G GAT) (ODN‐**2**) α‐3′‐d(AGT ATT G **A** C CTA) (ODN‐**5**)	62/14	−119	−327	−17.1
α‐5′‐d(TCA TAA C **17** G GAT) (ODN‐**3**) α‐3′‐d(AGT ATT G **A** C CTA) (ODN‐**5**)	61^[d]^/12	−109^[d]^	−298^[d]^	−16.2^[d]^

Homochiral (α/α) duplexes with double modification				
α‐5′‐d(TCA TAA C **2** G GAT) (ODN‐**2**) α‐3′‐d(AGT ATT G **9** C CTA) (ODN‐**7**)	58/12	−81	−218	−13.6
α‐5′‐d(TCA TAA C **2** G GAT) (ODN‐**2**) α‐3′‐d(AGT ATT G **19** C CTA) (ODN‐**8**)	58^[d]^/11	−80^[d]^	−213^[d]^	−14.1^[d]^
α‐5′‐d(TCA TAA C **17** G GAT) (ODN‐**3**) α‐3′‐d(AGT ATT G **9** C CTA) (ODN‐**7**)	58^[d]^/11	−75^[d]^	−197^[d]^	−13.7^[d]^
α‐5′‐d(TCA TAA C **17** G GAT) (ODN‐**3**) α‐3′‐d(AGT ATT G **19** C CTA) (ODN‐**8**)	57^[d]^/12	−60^[d]^	−153^[d]^	−12.5^[d]^
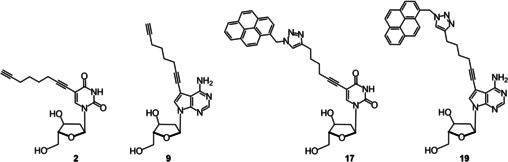				

[a] Measured at 260 nm at a concentration of 5 μM+5 μM single strand at a heating rate of 1.0 °C min^−1^ in 100 mM NaCl, 10 mM MgCl_2_, and 10 mM Na‐cacodylate (pH 7.0). [b] *T*_m_ values were calculated from the heating curves using the program Meltwin 3.0.^[12]^ [c] H % corresponds to hypochromicity in %. [d] For duplexes containing pyrene click adducts, a concentration of 2 μM+2 μM single strand was used. A few single stranded α‐d oligonucleotides showed weak cooperative melting which disappeared after hybridization (data are shown in Table S3 and Figure S4).

Compared to that 7‐deazapurine functionalized duplexes are generally less stable. This phenomenon is also apparent from duplexes with double modifications and is in line with previous results on homochiral DNA formed by both strands in β‐d configuration.[^3^, ^17^] Hypochromicities were low for α/α duplexes (ca. 12 %) with respect to α/β or β/β duplexes (ca. 20 %). Obviously, the base overlap is significantly different in homochiral α/α DNA compared to β/β or α/β DNA. Nevertheless, base pairing is extremely strong.

From the thermodynamic data it is obvious that a favorable enthalpy is responsible for this phenomenon. Encouraged by these results, we synthesized the α‐d anomeric counterpart of the self‐complementary Dickerson Drew dodecamer^[18]^ β‐5′‐d(CGC GAA TTC GCG)_2_ (ODN‐**18**), namely α‐5′‐d(CGC GAA TTC GCG)_2_ (ODN‐**9**). The *T*_m_ of this α‐anomeric Dickerson duplex was extremely high under buffer conditions used in this manuscript and a complete melting profile could not be recorded. Consequently, a low salt buffer was chosen. Now, a complete sigmoidal melting curve was observed with a *T*_m_ value of 75 °C (Figure S4). The β‐configurated Dickerson dodecamer showed a *T*_m_ value of 40 °C for duplex melting. It is obvious that non‐self‐complementary and also self‐complementary α/α duplexes exhibit a much higher stability than those with β/β‐configuration.

**Figure 6:**
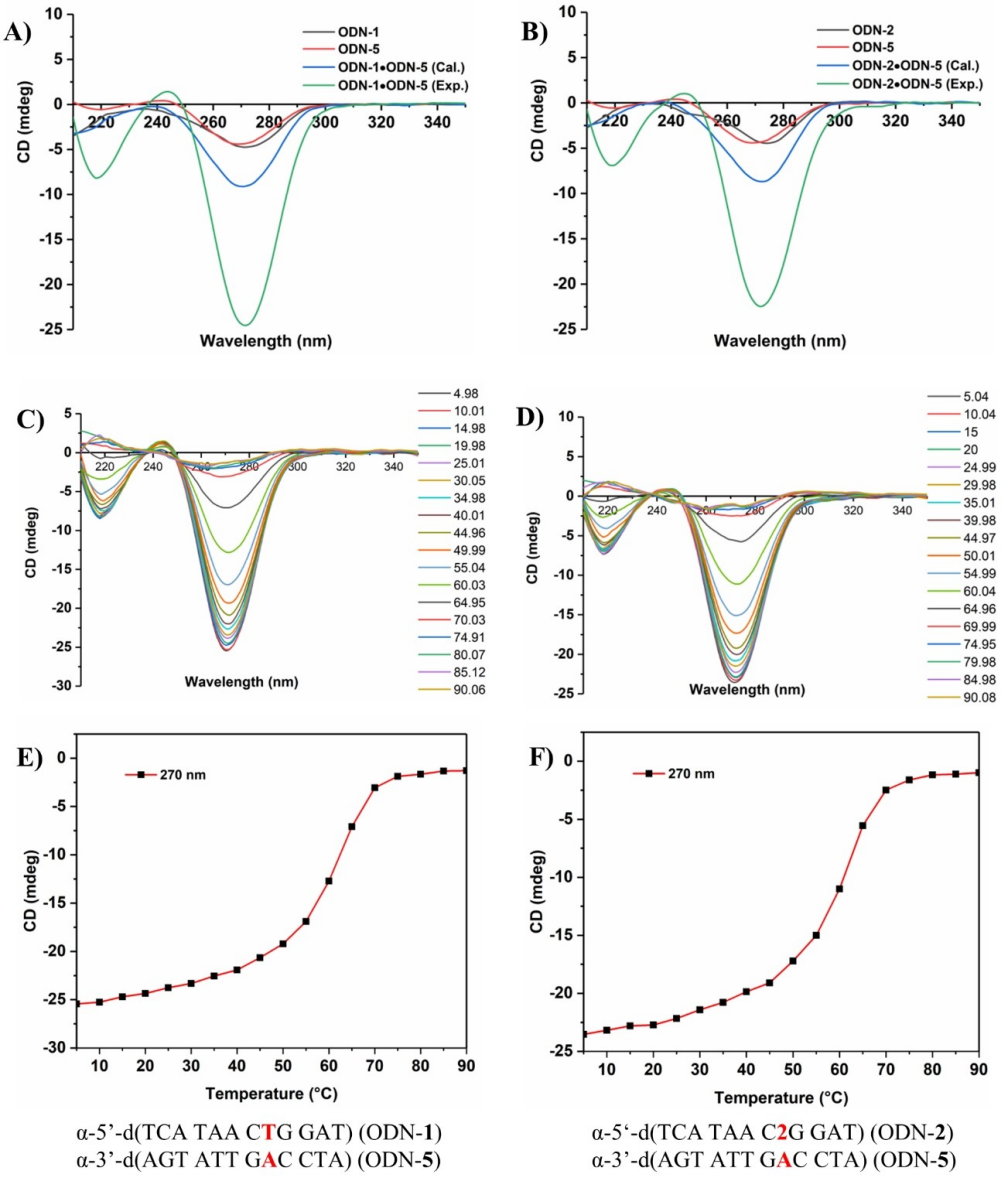
A) and B) CD spectra of single‐ and double‐stranded oligonucleotides with α‐d configuration. Blue curves show the calculated spectra (sum of the CD spectra of the single strands). Green curves show the experimental determined spectra. Temperature‐dependent CD‐spectra of C) ODN‐**1** ⋅ ODN‐**5** and D) (ODN‐**2** ⋅ ODN‐**5**). CD melting curves of duplexes obtained from temperature‐dependent CD spectra of E) ODN‐**1** ⋅ ODN‐**5** and F) ODN‐**2** ⋅ ODN‐**5**. All measurements were performed in 100 mM NaCl, 10 mM MgCl_2_, and 10 mM Na‐cacodylate, pH 7.0. The cell path length of the cuvette for the CD spectra was 5 mm.

To get information on global changes of oligonucleotide duplexes formed by two α‐d strands CD spectra were measured. In Figure 6A, B, the measured and calculated spectra of oligonucleotide duplexes with α/α‐configuration as well as spectra of the corresponding single strands are displayed. All CD spectra showed maxima with negative Cotton effects around 278 nm. This is different to the CD spectra of the heterochiral α/β duplexes all showing positive lobes. Temperature dependent CD spectra were used to determine *T*_m_ values (Figure 6C, D). The *T*_m_ data obtained by CD spectra (Figure 6E, F) were almost identical to those measured by UV showing high *T*_m_ values around 60 °C.

**Figure 7:**
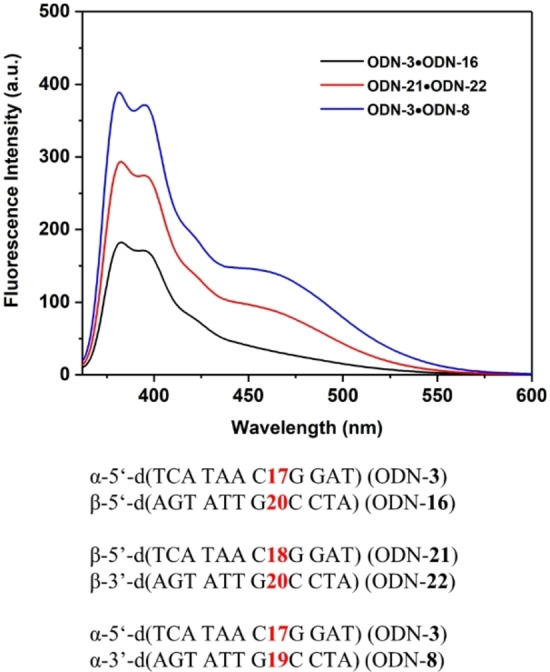
Fluorescence emission spectra of the heterochiral α/β duplex ODN‐**3** ⋅ ODN‐**16**, the homochiral β/β (ODN‐**21** ⋅ ODN‐**22**) and α/α (ODN‐**3** ⋅ ODN‐**8**) oligonucleotide duplexes containing pyrene click conjugates. All spectra were measured in 100 mM NaCl, 10 mM MgCl_2_, and 10 mM Na‐cacodylate (pH 7.0) with a concentration of 2 μM+2 μM. The excitation wavelength was 344 nm in all cases.

A few homochiral and heterochiral duplexes reported in this study contain pyrene residues linked to the α‐d or the β‐d oligonucleotide strands. As discussed above, pyrene residues contribute stability to duplexes. Pyrene is a fluorescent molecule with five emission peaks (375–405 nm) and an additional band (excimer) when two pyrenes are in proximal position.^[19]^ As excimer emission is sensitive to environmental changes, it can be used to determine intermolecular interactions.[^20^, ^21^] Thus, differences might exist among the various duplexes with oligonucleotides in anomeric configuration. To this end, three duplexes were chosen with homochiral α/α, β/β strands and heterochiral α/β configuration and fluorescence measurements were performed. According to Figure 7, all duplexes show monomer fluorescence but only the homochiral duplexes ODN‐**3** ⋅ ODN‐**8** (α/α) and ODN‐**21** ⋅ ODN‐**22** (β/β) show significant excimer emission.

### Molecular models of anomeric DNA and impact of nucleobases and side chains

**Figure 8:**
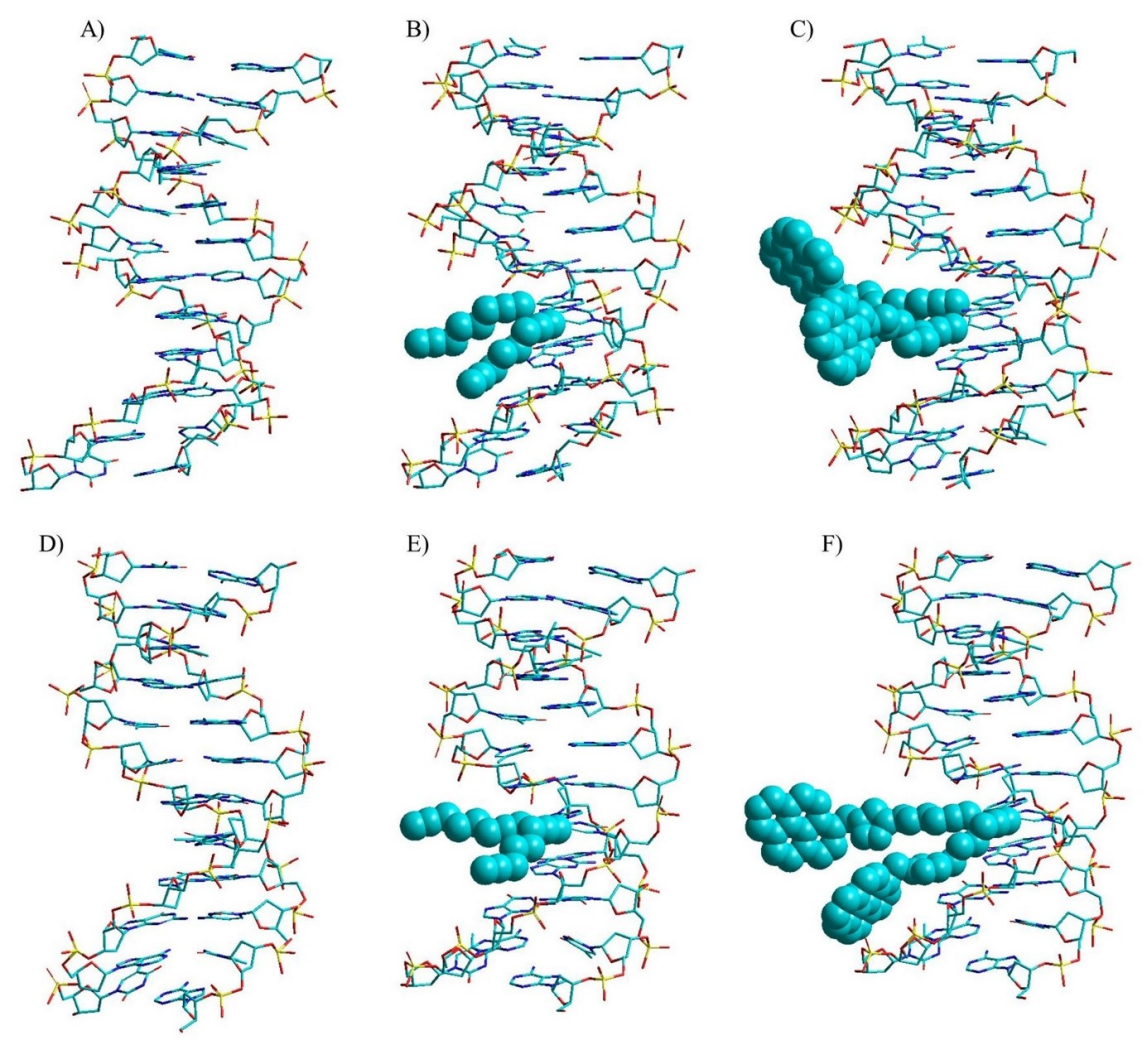
Calculated minimum‐energy structures of heterochiral parallel α/β‐d duplexes. A) ODN‐**1** ⋅ ODN‐**11**; B) ODN‐**2** ⋅ ODN‐**15**; C) ODN‐**3** ⋅ ODN‐**16**. Calculated minimum‐energy structures of homochiral antiparallel α/α‐d duplexes. D) ODN‐**1** ⋅ ODN‐**5**; E) ODN‐**2** ⋅ ODN‐**7**; F) ODN‐**3** ⋅ ODN‐**8** using the AMBER force field implemented in the software package HyperChem 8.0. The side chains are presented as space‐filling balls in cyan.

Molecular models of anomeric DNA were constructed by Amber force field incorporated in the software package HyperChem 8.0 for Windows (Hypercube, Inc.). Original Amber parameters were used (see the Experimental Section) and no water or counter ions were added. All duplexes were energy minimized but not refined. To this end, the β‐nucleosides from one strand of the standard β/β duplex ODN‐**12** ⋅ ODN‐**13** were replaced by α‐nucleosides including those with functionalized side chains. For homochiral duplexes with both strands in α/α‐d configuration and antiparallel strand alignments, all β‐nucleoside residues were substituted by α‐nucleosides including the nucleosides with clickable side chains or pyrene click adducts. Figure 8A–C shows the impact of octadiynyl side chains and pyrene click adducts on the particular double‐helix structures. Structures of the corresponding β/β duplexes and space filling models of all structures are shown in Figures S8A–C and S9A–I. For clarity, helices are shown as tubes and to visualize the space requirements of side chains they are presented as cyan space filling balls. Figure S9A–I provides information on the available space in the major grooves of the different DNA structures. From that it is obvious, that the clickable octadiynyl residues as well as the bulky pyrene click adducts are well accommodated in heterochiral and homochiral double helices and do not disturb the global double‐helix structure.

Anomeric DNA with strands in α‐d and β‐d configurations form duplexes with parallel strand orientation. The same strand alignment was reported for duplexes containing iG_d_‐dC, iC_d_‐dG and reversed Watson‐Crick base pairs (Donohue pair; iG_d_=2′‐deoxyisoguanosine, iC_d_=2′‐deoxy‐5‐methylisocytidine).^[22]^

According to Figure 8, heterochiral duplexes with parallel strand alignment form adenine‐thymine and guanine‐cytosine base pairs in the Watson‐Crick mode. Furthermore, Hoogsteen pairing involving nitrogen‐7 of purine bases can be excluded due to the absence of this purine nitrogen. This principle has been already reported for heterochiral α/β DNA in which all purine bases of the β‐strand are replaced by nonfunctionalized 7‐deazapurines.^[3]^ The absence of purine nitrogen‐7 and the replacement of the electronegative nitrogen atom of dA by an electropositive CH group (→c^7^A_d_) affect the electrostatic potential in the major groove. Base stacking interactions are reduced and as a result a slight enthalpy destabilization of the c^7^A_d_‐dT base pair is observed with respect to the dA‐dT pair. Nevertheless, base pairing geometry of the Watson‐Crick mode was retained.^[17]^ Our *T*_m_ measurements and thermodynamic data indicate that this is also valid for anomeric DNA.

## Conclusion

DNA possesses an intrinsic polymorphism that depends on the sequence, the structure of nucleobases, and the sugar‐phosphodiester backbone.[^23^, ^24^, ^25^] Base recognition and helix conformation play vital roles. This work reports the impact of clickable side chains and click adducts on heterochiral DNA with complementary strands in the α‐d and β‐d configurations and their homochiral counterparts with both strands in an α‐d or β‐d configuration. Strand communication occurs in anomeric DNA. To this end, the α‐anomer of 2′‐deoxyuridine was functionalized with clickable octadiynyl side chains at the nucleobase 5‐position, and the α‐anomer of 7‐deaza‐2′‐deoxyadenosine at position‐7. Functionalized nucleosides were protected and converted to phosphoramidite building blocks, and oligonucleotides were synthesized. They were clicked to a bulky fluorescent pyrene azide. Heterochiral and homochiral duplexes were formed after hybridization. According to the *T*_m_ values and thermodynamic data, alkynyl side chains of moderate size and with bulky pyrene residues are well accommodated in homochiral and heterochiral DNAs. Side‐chain functionalization has only a minor effect on the stability of α/β‐DNA or on DNA with both strands in the α/α configuration. Remarkably, α/α‐DNAs are much more stable than their α/β and β/β counterparts. The order of duplex stability was α/α‐d≫β/β‐d≥α/β‐d. CD spectra of all α‐d single strands show mirror‐like behavior with respect to β‐d oligomers. After hybridization, α/β duplexes exhibit positive Cotton effects similar to those of their β/β counterparts, whereas α/α‐d duplexes display negative signs. The global changes in the α‐strands can be attributed to conformational helix adaption during base‐pair formation. In all of our cases, the β‐strands dictated the sign of the CD spectrum in the final duplex. The functionalized DNAs were modeled and energy minimized. HyperChem 8.0 dynamic simulations followed by AMBER force‐field energy minimization showed that heterochiral and homochiral duplexes containing clickable side chains or click adducts form stable duplex structures. Side chains have sufficient space in double helices. With the knowledge from this investigation, almost any functionality or covalent label can be incorporated into anomeric DNAs by base modification without disturbing the helix structure. Clickable DNAs^[26]^ can be used as hybridization probes in nucleic acids diagnostics, chemical biology, and material science to expand the toolbox of nucleic acid applications beyond canonical DNA.[^7^, ^9^]

## Experimental Section

**General**: All chemicals and solvents were of laboratory grade as obtained from Acros Organics or Sigma Aldrich and were used without further purification. Flash column chromatography (FC): silica gel 60 from VWR (40–60 μM) at 0.4 bar. UV‐spectra were recorded on a Hitachi U‐3000 UV spectrophotometer: *λ*_max_ (*ϵ*) in nm, *ϵ* in dm^3^ mol^−1^ cm^−1^. ^13^C NMR spectra were measured on a Varian AS 400 or AS 600 Mercuryplus or Bruker Autoflex 300 NMR spectrophotometer at 599.74, 399.89, or 300.15 MHz for ^1^H at 150.82, 100.56, or 75.47 MHz for ^13^C, and at 121.5 MHz for ^31^P. ^1^H,^13^C correlated (HMBC, HSQC) NMR spectra were used for the assignment of the ^13^C signals (Table S1). The *J* values are given in Hz; *δ* values in ppm relative to Me_4_Si as internal standard. For NMR spectra recorded in [D_6_]DMSO, the chemical shift of the solvent peak was set to 2.50 ppm for ^1^H NMR and 39.50 ppm for ^13^C NMR. ESI‐TOF mass spectra of nucleosides were recorded on a Micro‐TOF spectrometer.

**Oligonucleotide syntheses and characterization**: Solid‐phase oligonucleotide syntheses were performed on an ABI 392‐08 synthesizer at 1 μmol scale (trityl‐on mode) employing the phosphoramidites **4**, **12**, **15** ^[9]^ and **16** ^[7]^ as well as the standard building blocks with an average coupling yield over 95 %. After cleavage from the solid support, the oligonucleotides were deprotected in 28 % aqueous ammonia at 55 °C for 2 h. The DMT‐containing oligonucleotides were purified by reversed‐phase HPLC (RP‐18) with the gradient system at 260 nm: A) MeCN, B) 0.1 M (Et_3_NH)OAc (pH 7.0)/MeCN, 95 : 5; gradient *I*: 0–3 min 10–15 % A in B, 3–15 min 15–50 % A in B; flow rate 0.7 mL/min. The purified “trityl‐on” oligonucleotides were treated with 2.5 % CHCl_2_COOH/CH_2_Cl_2_ for 2 min at 0 °C to remove the 4,4′‐dimethoxytrityl residues. The detritylated oligomers were purified again by reversed‐phase HPLC with gradient *II*: 0–20 min 0–20 % A in B; 20–25 min, 20 % A in B; flow rate 0.7 mL/min. The oligonucleotides were desalted on a reversed‐phase column (RP‐18) using water for elution of salt, while the oligonucleotides were eluted with H_2_O/CH_3_OH (2 : 3). The oligonucleotides were lyophilized on a Speed‐Vac evaporator to yield colorless solids which were frozen at −24 °C. The molecular masses of the oligonucleotides were determined by MALDI‐TOF mass spectrometry on a Bruker Autoflex Speed in the linear positive mode with 3‐hydroxypicolinic acid (3‐HPA) as a matrix. The thermal melting curves were measured with an Agilent Technologies Cary 100 Bio UV‐vis spectrophotometer equipped with a thermoelectrical controller. The temperature was measured continuously in the reference cell with a Pt‐100 resistor with a heating rate of 1 °C min^−1^. *T*_m_ values were determined from the melting curves using the software Meltwin, version 3.0.^[12]^ CD spectra were recorded at 25 °C on a Jasco J‐815 spectrometer.

The extinction coefficients *ϵ*_260_ of the nucleosides are: dA 15 400, dG 11 700, dT 8800, dC 7300, α‐5‐octadiynyl‐dU (**2**) 3200, β‐5‐octadiynyl‐dU (**13**) 3800,^[7]^ β‐7‐octadiynyl‐c^7^A_d_ (**14**) 5300,^[7]^ α‐7‐octadiynyl‐c^7^A_d_ (**9**) 6400 and their pyrene click conjugates **17** 14 600, **18** 13 800 and **20** 19 400 mol^−1^ dm^−3^ cm^−1^. The extinction coefficients of the oligonucleotides were calculated from the sum of the extinction coefficients of nucleoside constituents considering the hypochromic change for the particular single strands. Click reactions were made by post‐modification of modified oligonucleotide single strands. The DNA synthesizer cycle was the same for α‐d oligonucleotides as for their β‐d counterparts. Fluorescence spectra were recorded on a fluorescence spectrophotometer (Hitachi, Tokyo, Japan) in the wavelength range between 350 and 600 nm. Molecular modeling was performed with the Amber force field as incorporated in the software package HyperChem 8.0 (Hypercube Inc., Gainesville, FL, USA). Only original Amber parameters were used and no counter ions or water were included. A distance dependent scale factor of *ϵ*=1 was used. One to four non‐bonded interactions were scaled by 0.5. No cutoffs were applied. All duplex structures were built on the basis of a B‐DNA energy minimized but not refined.

**1‐(2‐Deoxy‐α‐d‐*erythro*‐pentofuranosyl)‐5‐(octa‐1,7‐diynyl)uracil (2)**: To a suspension of α‐5‐iodo‐2′‐deoxyuridine (**1**)^[4]^ (500 mg, 1.41 mmol) and CuI (54 mg, 0.28 mmol) in anh. DMF (7.5 mL) was added successively Pd(PPh_3_)_4_ (163 mg, 0.14 mmol), anh. Et_3_N (286 mg, 2.82 mmol), and octa‐1,7‐diyne (1.5 g, 14.1 mmol). The mixture was stirred at RT under Ar until the starting material was consumed (TLC monitoring). The reaction mixture was extracted with CH_2_Cl_2_ and H_2_O. The organic layer was collected, dried with Na_2_SO_4_ and evaporated. The combined filtrate was concentrated and the residue purified by FC (silica gel, column 15×3 cm, CH_2_Cl_2_/MeOH, 92 : 8) furnishing **2** (263 mg, 56 %) as colorless amorphous solid. TLC (CH_2_Cl_2_/MeOH, 9 : 1): *R*_f_=0.36; ^1^H NMR (600 MHz, [D_6_]DMSO, 26 °C): *δ*=11.51 (s, 1H, NH), 8.05 (s, 1H, H‐6), 6.09 (dd, *J*=7.8, 2.2 Hz, 1H, H‐1′), 5.36 (d, *J*=2.9 Hz, 1H, HO‐3′), 4.85 (t, *J*=5.6 Hz, 1H, HO‐5′), 4.23 (ddt, *J*=5.9, 3.0, 1.5 Hz, 1H, H‐3′), 4.18 (td, *J*=4.8, 1.5 Hz, 1H, H‐4′), 3.42–3.35 (m, 2H, H‐5′), 2.76 (t, *J*=2.6 Hz, 1H, C≡CH), 2.56 (ddd, *J*=14.0, 7.8, 5.9 Hz, 1H, H‐2′_β_), 2.39 (t, *J*=6.7 Hz, 2H, CH_2_), 2.19 (td, *J*=6.7, 2.6 Hz, 2H, CH_2_), 1.92 (dt, *J*=14.3, 1.9 Hz, 1H, H‐2′_α_), 1.62–1.51 (m, 4H, 2x CH_2_); ^13^C NMR (151 MHz, [D_6_]DMSO, 26 °C): *δ*=161.9 (C‐6), 149.6 (C‐2), 143.7 (C‐4), 98.3 (C‐5), 92.6 (C≡C), 89.7 (C‐4′), 86.2 (C‐1′), 84.3 (C≡C), 73.1 (C≡C), 71.4 (C≡C), 70.5 (C‐3′), 61.7 (C‐5′), 40.0 (C‐2′), 27.3 (CH_2_), 27.1 (CH_2_), 18.3 (CH_2_), 17.3 (CH_2_); UV (MeOH): *λ*_max_ (*ϵ*)=230 (11 900), 293 (11 700 mol^−1^ dm^3^ cm^−1^); HRMS (ESI‐TOF): *m*/*z* calcd for C_17_H_20_N_2_NaO_5_^+^: 355.1270 [*M*+Na]^+^; found: 355.1260.

**1‐(2‐Deoxy‐5‐*O*‐(4,4′‐dimethoxytriphenylmethyl)‐α‐d–*erythro*‐pentofuranosyl)‐5‐(octa‐1,7‐diynyl)uracil (3)**: Compound **2** (100 mg, 0.3 mmol) was dried by repeated co‐evaporation with dry pyridine (3×3 mL) and suspended in dry pyridine (3.5 mL). Then, 4,4′‐dimethoxytrityl chloride (142 mg, 0.42 mmol) was added and the mixture stirred for 3 h at RT. Then, the mixture was diluted with CH_2_Cl_2_ (20 mL) and 5 % aq. NaHCO_3_ soln. (20 mL) was added. The organic phase was dried over Na_2_SO_4_ and evaporated, and the residue was separated by FC (silica gel, column 10×3 cm, CH_2_Cl_2_/MeOH, 96 : 4) to give **3** (165 mg, 87 %) as colorless foam. *R*_f_=0.38 (CH_2_Cl_2_/MeOH, 95 : 5); ^1^H NMR (600 MHz, [D_6_]DMSO, 26 °C): *δ*=11.55 (s, 1H, NH), 8.05 (s, 1H, H‐6), 7.37 (dd, *J*=8.4, 1.3 Hz, 2H, arom. H), 7.32 (dd, *J*=8.5, 7.0 Hz, 2H, arom. H), 7.24 (dt, *J*=9.1, 2.2 Hz, 5H, arom. H), 6.93–6.89 (m, 4H, arom. H), 6.19 (dd, *J*=7.8, 2.5 Hz, 1H, H‐1′), 5.43 (d, *J*=3.0 Hz, 1H, HO‐3′), 4.34 (td, *J*=4.6, 1.5 Hz, 1H, H‐3′), 4.27–3.99 (m, 1H, H‐4′), 3.74 (s, 6H, OCH_3_), 3.06 (dd, *J*=10.3, 4.3 Hz, 1H, H‐5′), 2.96 (dd, *J*=10.2, 4.8 Hz, 1H, H‐5”), 2.75 (t, *J*=2.6 Hz, 1H, C≡CH), 2.59 (ddd, *J*=14.1, 7.7, 5.9 Hz, 1H, H‐2′_β_), 2.39 (t, *J*=6.7 Hz, 2H, CH_2_), 2.19 (td, *J*=6.7, 2.6 Hz, 2H, CH_2_), 1.98 (dt, *J*=14.4, 2.1 Hz, 1H, H‐2′_α_), 1.56 (dddd, *J*=6.9, 5.3, 3.6, 1.7 Hz, 4H, 2x CH_2_); ^13^C NMR (151 MHz, [D_6_]DMSO, 26 °C): *δ*=161.9 (C‐6), 158.1 (Ar−C), 149.6 (C‐2), 144.7 (Ar−C), 143.5 (C‐4), 135.5 (Ar−C), 135.4 (Ar−C), 129.7 (Ar−C), 129.7 (Ar−C), 127.9 (Ar−C), 127.6 (Ar−C), 126.7 (Ar−C), 113.3 (Ar−C), 98.4 (C‐5), 92.7 (C≡C), 87.8 (qC), 86.2 (C‐1′), 85.7 (C‐4′), 84.3 (C≡C), 73.1 (C≡C), 71.4 (C≡C), 70.9 (C‐3′), 63.9 (C‐5′), 55.0 (OCH_3_), 40.1 (C‐2′), 27.3 (CH_2_), 27.1 (CH_2_), 18.3 (CH_2_), 17.3 (CH_2_); UV (MeOH): *λ*_max_ (*ϵ*)=233 (31 000), 284 (11 500 mol^−1^ dm^3^ cm^−1^); HRMS (ESI‐TOF): *m*/*z* calcd for C_38_H_38_N_2_NaO_7_^+^: 657.2577 [*M*+Na]^+^; found: 657.2572.

**1‐(2‐Deoxy‐5‐*O*‐(4,4′‐dimethoxytriphenylmethyl)‐α‐d‐*erythro*‐pentofuranosyl)‐5‐(octa‐1,7‐diynyl)uracil 3′‐(2‐cyanoethyl)‐*N***, ***N*** **‐diisopropylphosphoramidite (4)**: To a solution of compound **3** (100 mg, 0.16 mmol) and anhydrous iPr_2_EtN (53 μL, 0.8 mmol) in anhydrous CH_2_Cl_2_ (8.0 mL), 2‐cyanoethyl diisopropylphosphoramidochloridite (47 μL, 0.27 mmol) was added at RT. After stirring for 20 min, the mixture was diluted with CH_2_Cl_2_ (15 mL) and the reaction was quenched by adding 5 % aq. NaHCO_3_ solution (25 mL). Then, the aqueous layer was extracted with CH_2_Cl_2_ (60 mL), the combined organic layer was dried (Na_2_SO_4_) and evaporated. The residual colorless oil was applied to FC (silica gel, column 10×2 cm, CH_2_Cl_2_/acetone, 9 : 1). From the main zone a colorless foam of compound **4** was obtained as a mixture of diastereoisomers (90 mg, 68 %). TLC (silica gel, CH_2_Cl_2_/acetone, 80 : 20) *R_f_* =0.4; ^31^P NMR (121 MHz, CDCl_3_, 26 °C): *δ*=149.14; 149.37 ppm. HRMS (ESI‐TOF) *m*/*z*: [*M*+H]^+^ calcd for C_47_H_55_N_4_NaO_8_P^+^ 857.3655; found 857.3654.

**1‐(2‐Deoxy‐α‐d–*erythro*‐pentofuranosyl)‐5‐(6‐(1‐(pyren‐1‐ylmethyl)‐1*H*‐1,2,3‐triazol‐4‐yl)hex‐1‐yn‐1‐yl)uracil (17)**: Compound **2** (50 mg, 0.15 mmol) and pyrene methyl azide (54 mg, 0.21 mmol) were dissolved in THF/H_2_O/*t*BuOH (3 : 1 : 1, *v*/*v*, 4 mL), then sodium ascorbate (67 μL, 0.6 mmol) of a freshly prepared 1 M solution in water was added, followed by the addition of copper(II)sulfate pentahydrate 7.5 % in water (53 μL, 0.015 mmol). The reaction mixture was stirred overnight at room temperature. The solvent was evaporated, and the residue was purified by FC (silica gel, column 10×3 cm, CH_2_Cl_2_/MeOH, 25 : 1) to give **17** (54 mg, 62 %) as a light yellow solid. *R*_f_=0.40 (CH_2_Cl_2_/MeOH, 10 : 1); ^1^H NMR (600 MHz, [D_6_]DMSO, 26 °C): *δ*=11.52 (s, 1H, NH), 8.51 (d, *J*=9.3 Hz, 1H, arom. H), 8.36–8.27 (m, 4H, arom. H), 8.23–8.17 (m, 2H, arom. H), 8.10 (t, *J*=7.6 Hz, 1H, arom. H), 8.04 (s, 1H, H‐6), 7.98 (d, *J*=7.9 Hz, 1H, arom. H), 7.92 (s, 1H, C=CH), 6.32 (s, 2H, CH_2_), 6.10 (dd, *J*=7.7, 2.3 Hz, 1H, H‐1′), 5.37 (d, *J*=2.9 Hz, 1H, HO‐3′), 4.86 (t, *J*=5.6 Hz, 1H, HO‐5′), 4.23 (ddt, *J*=4.4, 2.8, 1.5 Hz, 1H, H‐3′), 4.18 (td, *J*=4.8, 1.6 Hz, 1H, H‐4′), 3.37 (t, *J*=5.1 Hz, 2H, H‐5′), 2.60 (t, *J*=7.5 Hz, 2H, CH_2_), 2.58–2.52 (m, 1H, H‐2′_β_), 2.35 (t, *J*=7.2 Hz, 2H, CH_2_), 1.91 (dt, *J*=14.4, 2.0 Hz, 1H, H‐2′_α_), 1.66 (dq, *J*=9.2, 7.6 Hz, 2H, CH_2_), 1.49 (dq, *J*=9.6, 7.2 Hz, 2H, CH_2_); ^13^C NMR (151 MHz, [D_6_]DMSO, 26 °C): *δ*=161.9 (C‐6), 149.6 (C‐2), 146.9 (Ar−C), 143.6 (C‐4), 130.9 (triazole‐C), 130.7 (Ar−C), 130.2 (Ar−C), 129.3 (Ar−C), 128.4 (Ar−C), 128.2 (Ar−C), 127.7 (Ar−C), 127.5 (Ar−C), 127.3 (Ar−C), 126.5 (Ar−C), 125.7 (Ar−C), 125.5 (Ar−C), 125.1 (Ar−C), 124.0 (triazole‐C), 123.7 (Ar−C), 122.8 (Ar−C), 122.1 (Ar−C), 98.3 (C‐5), 92.8 (C≡C), 89.7 (C‐4′), 86.2 (C‐1′), 73.0 (C≡C), 70.5 (C‐3′), 61.7 (C‐5′), 50.7 (CH_2_), 40.0 (C‐2′), 28.1 (CH_2_), 27.7 (CH_2_), 24.4 (CH_2_), 18.5 (CH_2_); UV (MeOH): *λ*_max_ (*ϵ*)=266 (25 600), 276 (48 200), 311 (16 500), 326 (26 500), 342 (37 500 mol^−1^ dm^3^ cm^−1^); HRMS (ESI‐TOF): *m*/*z* calcd for C_34_H_31_N_5_NaO_5_^+^: 612.2223 [*M*+Na]^+^; found: 612.2217.

**1‐(2‐Deoxy‐β‐d‐*erythro*‐pentofuranosyl)‐5‐(6‐(1‐(pyren‐1‐ylmethyl)‐1*H*‐1,2,3‐triazol‐4‐yl)hex‐1‐yn‐1‐yl)uracil (18)**: Compound **13** ^[4]^ (50 mg, 0.15 mmol) and pyrene methyl azide (54 mg, 0.21 mmol) were dissolved in THF/H_2_O/*t*BuOH (3 : 1 : 1, *v*/*v*, 4 mL), then sodium ascorbate (67 μL, 0.6 mmol) of a freshly prepared 1 M solution in water was added, followed by the addition of copper(II) sulfate pentahydrate 7.5 % in water (53 μL, 0.015 mmol). The reaction mixture was stirred overnight at RT. The solvent was evaporated, and the residue was purified by FC (silica gel, column 10×3 cm, CH_2_Cl_2_/MeOH, 25 : 1) to give **18** (61 mg, 70 %) as a light yellow solid. *R*_f_=0.39 (CH_2_Cl_2_/MeOH, 10 : 1); ^1^H NMR (600 MHz, [D_6_]DMSO, 26 °C): *δ*=11.55 (s, 1H, NH), 8.51 (d, *J*=9.2 Hz, 1H, arom. H), 8.36–8.27 (m, 4H, arom. H), 8.24–8.16 (m, 2H, arom. H), 8.14–8.08 (m, 2H, arom. H), 7.99 (d, *J*=7.9 Hz, 1H, H‐6), 7.91 (s, 1H, C=CH), 6.32 (s, 2H, CH_2_), 6.11 (t, *J*=6.7 Hz, 1H, H‐1′), 5.23 (d, *J*=4.3 Hz, 1H, HO‐3′), 5.12 (t, *J*=5.0 Hz, 1H, HO‐5′), 4.33–4.15 (m, 1H, H‐3′), 3.78 (q, *J*=3.4 Hz, 1H, H‐4′), 3.67–3.50 (m, 2H, H‐5′), 2.60 (t, *J*=7.5 Hz, 2H, CH_2_), 2.35 (t, *J*=7.1 Hz, 2H, CH_2_), 2.10 (ddd, *J*=6.4, 4.8, 3.3 Hz, 2H, H‐2′), 1.66 (tt, *J*=8.0, 6.7 Hz, 2H, CH_2_), 1.55–1.44 (m, 2H, CH_2_); ^13^C NMR (151 MHz, [D_6_]DMSO, 26 °C): *δ*=161.8 (C‐6), 149.5 (C‐2), 146.9 (Ar−C), 142.7 (C‐4), 131.0 (triazole‐C), 130.7 (Ar−C), 130.2 (Ar−C), 129.3 (Ar−C), 128.4 (Ar−C), 128.2 (Ar−C), 127.8 (Ar−C), 127.5 (Ar−C), 127.3 (Ar−C), 126.5 (Ar−C), 125.7 (Ar−C), 125.6 (Ar−C), 125.1 (Ar−C), 124.0 (triazole‐C), 123.7 (Ar−C), 122.8 (Ar−C), 122.2 (Ar−C), 99.0 (C‐5), 93.0 (C≡C), 87.5 (C‐4′), 84.6 (C‐1′), 72.9 (C≡C), 70.1 (C‐3′), 61.0 (C‐5′), 50.8 (CH_2_), 40.0 (C‐2′), 28.1 (CH_2_), 27.6 (CH_2_), 24.4 (CH_2_), 18.5 (CH_2_); UV (MeOH): *λ*_max_ (*ϵ*)=266 (24 800), 276 (48 100), 311 (16 000), 326 (26 300), 342 (37 800 mol^−1^ dm^3^ cm^−1^); HRMS (ESI‐TOF): *m*/*z* calcd for C_34_H_31_N_5_NaO_5_^+^: 612.2223 [*M*+Na]^+^; found: 612.2219.

**4‐Amino‐7‐(2‐deoxy‐α‐d‐*erythro*‐pentofuranosyl)‐5‐(octa‐1,7‐diynyl)‐7*H*‐pyrrolo‐[2,3‐*d*]pyrimidine (9)**: To a suspension of α‐7‐iodo‐7‐deaza‐2′‐deoxyadenosine (**8**)^[8]^ (500 mg, 1.32 mmol) and CuI (51 mg, 0.26 mmol) in anh. DMF (5 mL) was added successively Pd(PPh_3_)_4_ (160 mg, 0.14 mmol), anh. Et_3_N (274 mg, 2.70 mmol), and octa‐1,7‐diyne (2.4 g, 22.6 mmol). The mixture was stirred at RT under Ar for 2 h. The solvent was evaporated and the remaining oily residue adsorbed on silica gel (25 g) and applied to FC (silica gel, column 15×3 cm, CH_2_Cl_2_/MeOH, 96 : 4) furnishing **9** (353 mg, 75 %) as yellowish amorphous solid. TLC (CH_2_Cl_2_/MeOH, 9 : 1): *R*_f_=0.40; ^1^H NMR (400 MHz, [D_6_]DMSO, 26 °C): *δ*=8.11 (s, 1H, H‐2), 7.78 (s, 1H, H‐6), 6.60 (br s, 2H, NH_2_), 6.49 (dd, *J*=8.1, 3.2 Hz, 1H, H‐1′), 5.57 (d, *J*=4.1 Hz, 1H, HO‐3′), 4.80 (t, *J*=5.6 Hz, 1H, HO‐5′), 4.29 (ddt, *J*=7.0, 4.2, 2.9 Hz, 1H, H‐3′), 4.05 (td, *J*=4.5, 3.0 Hz, 1H, H‐4′), 3.36–3.49 (m, 2H, H‐5′), 2.77 (t, *J*=2.7 Hz, 1H, C≡CH), 2.69–2.76 (m, 1H, H‐2′_β_), 2.50 (m, 2H, CH_2_), 2.22 (td, *J*=6.8, 2.7 Hz, 2H, CH_2_), 2.15 (t, *J*=3.0 Hz, 1H, H‐2′_α_), 1.54–1.69 (m, 4H, 2x CH_2_); ^13^C NMR (101 MHz, [D_6_]DMSO, 26 °C): *δ*=157.5 (C‐6), 152.5 (C‐2), 149.0 (C‐4), 126.3 (C‐8), 102.1 (C‐5), 95.1 (C‐7), 92.0 (C≡C), 88.1 (C‐4′), 84.3 (C≡C), 82.9 (C‐1′), 73.8 (C≡C), 71.4 (C≡C), 70.8 (C‐3′), 61.7 (C‐5′), 40.0 (C‐2′), 27.3 (CH_2_), 27.2 (CH_2_), 18.4 (CH_2_), 17.3 (CH_2_); UV (MeOH): *λ*_max_ (*ϵ*)=239 (14 700), 280 (10 300 mol^−1^ dm^3^ cm^−1^); HRMS (ESI‐TOF): *m*/*z* calcd for C_17_H_22_N_4_NaO_3_^+^: 377.1590 [*M*+Na]^+^; found: 377.1584.

**7‐(2‐Deoxy‐α‐d–*erythro*‐pentofuranosyl)‐4‐(isobutyryl)amino‐5‐(octa‐1,7‐diynyl)‐7*H*‐pyrrolo[2,3‐*d*]pyrimidine (10)**: To a solution of compound **9** (500 mg, 1.41 mmol) in anhydrous pyridine (6 mL) was added Me_3_SiCl (1.82 mL, 14.28 mmol) and stirred at RT. After 45 min, the isobutyryl chloride (180 mg, 1.7 mmol) was introduced, and the solution was stirred for another 3 h. The mixture was cooled to 0 °C, diluted with H_2_O (6 mL), and stirred for 10 min. After the addition of 14 % aq. NH_3_ (6 mL), stirring was continued for 1 h at room temperature. The solution was evaporated, and the residue was applied to FC (silica gel, column 10×3 cm, CH_2_Cl_2_/MeOH, 95 : 5). Compound **10** was isolated as colorless foam (322 mg, 60 %). TLC (CH_2_Cl_2_/MeOH, 9 : 1): *R*_f_=0.50; ^1^H NMR (400 MHz, [D_6_]DMSO, 26 °C): *δ*=9.92 (s, 1H, NH), 8.59 (s, 1H, H‐2), 8.09 (s, 1H, H‐6), 6.64 (dd, *J*=8.0, 2.9 Hz, 1H, H‐1′), 5.53 (d, *J*=3.7 Hz, 1H, HO‐3′), 4.83 (t, *J*=5.7 Hz, 1H, HO‐5′), 4.39 (m, 1H, H‐3′), 4.10 (td, *J*=4.5, 2.7 Hz, 1H, H‐4′), 3.36–3.52 (m, 2H, H‐5′), 2.89 (p, *J*=6.9 Hz, 1H, CH), 2.73–2.83 (m, 2H, C≡CH, H‐2′_β_), 2.42 (t, *J*=6.8 Hz, 2H, CH_2_), 2.20 (m, 3H, CH_2_, H‐2′_α_), 1.53–1.68 (m, 4H, 2x CH_2_), 1.16 (dd, *J*=6.9, 1.6 Hz, 6H, 2x CH_3_); ^13^C NMR (101 MHz, [D_6_]DMSO, 26 °C): *δ*=175.6 (C=O), 151.30 (C‐6), 151.28 (C‐4), 150.8 (C‐2), 130.6 (C‐8), 110.1 (C‐5), 96.5 (C‐7), 91.0 (C≡C), 88.5 (C‐4′), 84.2 (C≡C), 83.2 (C‐1′), 73.8 (C≡C), 71.3 (C≡C), 70.8 (C‐3′), 61.7 (C‐5′), 39.9 (C‐2′), 34.5 (Me_2_C), 27.4 (CH_2_), 27.3 (CH_2_), 19.09 (Me), 19.08 (Me), 18.6 (CH_2_), 17.3 (CH_2_); UV (MeOH): *λ*_max_ (*ϵ*)=211 (20 200), 240 (21 000), 279 (7800 mol^−1^ dm^3^ cm^−1^); HRMS (ESI‐TOF): *m*/*z* calcd for C_23_H_28_N_4_NaO_4_^+^: 447.2008 [*M*+Na]^+^; found: 477.2000.

**7‐(2‐Deoxy‐5‐*O*‐(4,4′‐dimethoxytrityl)‐α‐d–*erythro*‐pentofuranosyl)‐4‐(isobutyryl)amino‐5‐(octa‐1,7‐diynyl)‐7*H*‐pyrrolo[2,3‐*d*]pyrimidine (11)**: Compound **10** (280 mg 0.66 mmol) was dissolved in anhydrous pyridine (5 mL) and treated with 4,4′‐dimethoxytrityl chloride (291 mg, 0.86 mmol). The reaction mixture was stirred for 1 h at RT. Then, CH_2_Cl_2_ (20 mL) was added and the org. layer washed with 5 % NaHCO_3_ (35 mL). The org. phase was dried over Na_2_SO_4_ filtrated and evaporated. The remaining residue was applied to FC (silica gel, column 12×3 cm, CH_2_Cl_2_/acetone, 85 : 15). From the main zone **11** was obtained as colorless foam (238 mg, 49 %). TLC (CH_2_Cl_2_/acetone, 85 : 15): *R*_f_=0.50; ^1^H NMR (600 MHz, [D_6_]DMSO, 26 °C): *δ*=9.97 (s, 1H, NH), 8.62 (s, 1H, H‐2), 8.10 (s, 1H, H‐6), 7.37–7.42 (m, 2H, arom. H), 7.33 (dd, *J*=8.5, 7.2 Hz, 2H, arom. H), 7.25–7.29 (m, 4H, arom. H), 6.88‐6.93 (m, 4H, arom. H), 6.71 (dd, *J*=7.9, 3.3 Hz, 1H, H‐1′), 5.60 (dd, *J*=3.9, 1.1 Hz, 1H, HO‐3′), 4.28 (m, 2H, H‐3′, H‐4′), 3.12 (dd, *J*=10.2, 3.8 Hz, 1H, H‐5′), 3.01 (dd, *J*=10.2, 4.9 Hz, 1H, H‐5′), 2.89 (p, *J*=6.9 Hz, 1H, CH), 2.81 (p, *J*=7.6 Hz, 1H, H‐2′_β_), 2.76 (t, *J*=2.7 Hz, 1H, C≡CH), 2.42 (t, *J*=7.0 Hz, 2H, CH_2_), 2.25 (dt, *J*=14.1, 3.2 Hz, 1H, H‐2′_α_), 2.20 (td, *J*=6.9, 2.6 Hz, 2H, CH_2_), 1.61–1.67 (m, 2H, CH_2_), 1.55–1.61 (m, 2H, CH_2_), 1.17 (dd, *J*=6.9, 2.5 Hz, 6H, 2x CH_3_); ^13^C NMR (125 MHz, [D_6_]DMSO, 26 °C): *δ*=175.6 (C=O), 158.1 (Ar−C), 151.4 (C‐6), 151.3 (C‐4), 150.9 (C‐2), 144.8 (Ar−C), 135.6 (Ar−C), 135.5 (Ar−C), 130.5 (Ar−C), 129.73 (Ar−C), 129.7 (C‐8), 128.9 (Ar−C), 127.9 (Ar−C), 127.7 (Ar−C), 127.6 (Ar−C), 126.7 (Ar−C), 113.3 (Ar−C), 112.8 (Ar−C), 110.1 (C‐5), 96.7 (C‐7), 91.1 (C≡C), 86.5 (qC), 85.6 (C‐4′), 84.2 (C≡C), 83.3 (C‐1′), 73.8 (C≡C), 71.4 (C≡C), 71.1 (C‐3′), 64.0 (C‐5′), 55.0 (OCH_3_), 39.8 (C‐2′), 34.5 (Me_2_C), 27.3 (CH_2_), 27.2 (CH_2_), 19.12 (Me), 19.10 (Me), 18.6 (CH_2_), 17.3 (CH_2_); UV (MeOH): *λ*_max_ (*ϵ*)=236 (37 300), 276 (11 300 mol^−1^ dm^3^ cm^−1^); HRMS (ESI‐TOF): *m*/*z* calcd for C_44_H_46_N_4_NaO_6_^+^: 749.3315 [*M*+Na]^+^; found: 749.3309.

**7‐(2‐Deoxy‐5‐*O*‐(4,4′‐dimethoxytrityl)‐α‐d‐*erythro*‐pentofuranosyl)‐4‐(isobutyryl)amino‐5‐(octa‐1,7‐diynyl)‐7*H*‐pyrrolo[2,3‐*d*]pyrimidine 3′‐(2‐cyanoethyl)‐*N***, ***N*** **‐diisopropylphosphoramidite (12)**: Compound **11** (200 mg, 0.27 mmol) was dissolved in anh. CH_2_Cl_2_ (5 mL). Then, *N*,*N*‐diisopropylethylamine (85 μL, 0.849 mmol), and 2‐cyanoethyl‐*N*,*N*‐diisopropylphosphoramidochloridite (85 μL, 0.39 mmol) were added and the reaction mixture was stirred for 15 min. at RT. The reaction mixture was diluted with CH_2_Cl_2_ (20 mL) and was washed with 5 % NaHCO_3_, dried (Na_2_SO_4_) and purified by FC (silica gel, 10×2 cm, CH_2_Cl_2_/acetone, 95 : 5). A diastereoisomeric mixture of compound **12** was obtained as colorless foam (181 mg, 72 %). TLC (CH_2_Cl_2_/acetone, 90 : 10): *R*_f_=0.70. ^31^P NMR (121 MHz, CDCl_3_, 26 °C): *δ*=148.98; HRMS (ESI‐TOF): *m*/*z* calcd for C_53_H_63_N_6_NaO_7_P^+^: 949.4394 [*M*+Na]^+^; found: 949.4382.

**General procedure for Huisgen‐Meldal‐Sharpless [3+2] cycloaddition performed on oligonucleotides in aqueous solution with 1‐azidomethylpyrene**: To a ss‐oligonucleotide (5 A_260_ units) were added CuSO_4_⋅TBTA (1 : 2) ligand complex (50 μL of a 20 mM stock solution in H_2_O/DMSO/*t*BuOH, 4 : 3 : 1), tris(carboxyethyl)‐phosphine (TCEP, 50 μL of a 20 mM stock solution in water), NaHCO_3_ (50 μL, 200 mM stock solution in water), 1‐azidomethylpyrene (100 μL, 20 mM stock solution in H_2_O/dioxane/DMSO, 1 : 1 : 1), and DMSO (30 μL), and the reaction mixture was stirred at room temperature for 12 h. The reaction mixture was concentrated in a speed‐vac and dissolved in 500 μL bi‐distilled water and centrifuged for 30 min at 14 000 rpm. The supernatant solution was collected and further purified by reversed‐phase HPLC with the gradient 0–3 min 10–15 % B in A, 3–15 min 15–50 % B in A, 15–20 min 50–10 % B in A, flow rate 0.7 cm^3^ min^−1^. The molecular masses of the oligonucleotides were determined by MALDI‐TOF spectra (Table 1).

## Conflict of interest

The authors declare no conflict of interest.

1

## Supporting information

As a service to our authors and readers, this journal provides supporting information supplied by the authors. Such materials are peer reviewed and may be re‐organized for online delivery, but are not copy‐edited or typeset. Technical support issues arising from supporting information (other than missing files) should be addressed to the authors.

Supporting Information

## Data Availability

The data that support the findings of this study are available from the corresponding author upon reasonable request.
