# Supplementary material for: Anomeric DNA: Functionalization of α‐d Anomers of 7‐Deaza‐2′‐deoxyadenosine and 2′‐Deoxyuridine with Clickable Side Chains and Click Adducts in Homochiral and Heterochiral Double Helices
Source: Chemistry. 2022 Jan 14;28(9):e202103872. doi: 10.1002/chem.202103872 (PMC9304229; doi:10.1002/chem.202103872)
Supplement: Supplementary file 1 — Supporting Information [file CHEM-28-0-s001.pdf]

Chemistry–A European Journal

Supporting Information

Anomeric DNA: Functionalization of α-D Anomers of 7-Deaza-2′-deoxyadenosine and 2′-Deoxyuridine with Clickable Side Chains and Click Adducts in Homochiral and Heterochiral Double Helices

Aigui Zhang, Peter Leonard, and Frank Seela*

Table of Contents

Table S1. ^{13}C NMR chemical shifts of α-5-octadiynyl-dU derivatives (**2**, **3**, **17**) and β-5-octadiynyl-dU click conjugate **18** S2

Table S2. ^{13}C NMR chemical shifts of α-7-octadiynyl-7-deaza-2'-deoxyadenosine derivatives S2

Figure S1. Structures of nucleosides used in this study S3

Table S3. T_m values and thermodynamic data for single-stranded α-oligonucleotides S4

Figure S2. Mixing experiments of oligonucleotide duplexes S5

Figure S3. Reversed-phase HPLC elution profiles of purified oligonucleotides S6-9

Figure S4. Melting profiles of oligonucleotide duplexes S10-19

Figure S5. CD-spectra of oligonucleotide duplexes S20-29

Figure S6. Temperature-dependent CD-spectra of duplexes S30-32

Figure S7. Fluorescence spectra of nucleosides, oligonucleotides and duplexes containing pyrene click conjugates S33-37

Figure S8. Calculated minimum energy structures of homochiral antiparallel β/β-D duplexes (tubes) S38

Figure S9. Calculated minimum energy structures of homochiral antiparallel β/β-D duplexes (balls) S39

References S39

Figures S10-S56. NMR spectra of synthesized compounds S40-63

Table S1. ^{13}C NMR chemical shifts of α-5-octadiynyl-dU derivatives (**2**, **3**, **17**) and β-5-octadiynyl-dU click conjugate **18**.[a]

Cpd.	C(2)	C(4)	C(5)	C(6)	C≡C	C≡C	CH_2	Py-C	Triazole	OCH_3	C(1’)	C(2’)	C(3’)	C(4’)	C(5’)
**2**	149.6	143.7	98.3	161.9	71.4<br>84.3	73.1<br>92.6	17.3<br>18.3<br>27.1<br>27.3	-	-	-	86.2	40.0	70.5	89.7	61.7
**3**	149.6	143.5	98.4	161.9	71.4<br>84.3	73.1<br>92.7	17.2<br>18.3<br>27.1<br>27.3	-	-	55.0	86.2	40.1	70.9	85.7	63.9
**17**	149.6	143.6	98.3	161.9	-	73.0<br>92.8	18.5<br>24.4<br>27.7<br>28.1<br>50.7	122.1-<br>146.9	124.0<br>130.9	-	86.2	40.0	70.5	89.7	61.7
**18**	149.5	142.7	99.0	161.8	-	72.9<br>93.0	18.5<br>24.4<br>27.6<br>28.1<br>50.8	122.2-<br>147.0	124.0<br>131.0	-	84.6	40.0	70.1	87.5	61.0

[a] Measured in DMSO-d_6 at 298 K.

Table S2. ^{13}C NMR chemical shifts of α-7-octadiynyl-7-deaza-2’-deoxyadenosine derivatives.[a]

Cpd.	C(2)[b]<br>C(2)[c]	C(4)[b]<br>C(7*a*)[c]	C(5)[b]<br>C(4*a*)[c]	C(6)[b]<br>C(4)[c]	C(7)[b]<br>C(5)[c]	C(8)[b]<br>C(6)[c]	C≡C	C≡C	CH_2	Me_2CH<br>C=O	OCH_3	C(1’)	C(2’)	C(3’)	C(4’)	C(5’)
**8** [1]	151.8	149.6	102.9	157.1	53.1	127.6	-	-	-	-	-	83.9	- [d]	70.7	88.0	61.7
**9**	152.5	149.0	102.1	157.5	95.1	126.3	73.8<br>84.3	71.4<br>95.1	17.3<br>18.4<br>27.2<br>27.3	-	-	82.9	40.0	70.8	88.1	61.7
**10**	150.8	151.3	110.1	151.3	96.5	130.6	73.8<br>84.2	71.3<br>91.0	17.3<br>18.6<br>27.3<br>27.4	19.09<br>19.08<br>175.6	-	83.2	39.9	70.8	88.5	61.7
**11**	150.9	151.3	110.1	151.4	96.7	129.7	73.8<br>84.2	71.4<br>91.1	17.3<br>18.6<br>27.2<br>27.3	19.12<br>19.10<br>175.6	55.0	83.3	39.8	71.1	85.6	64.0

[a] Measured in DMSO-d_6 at 298 K. [b] Purine numbering. [c] Systematic numbering. [d] Superimposed by DMSO.

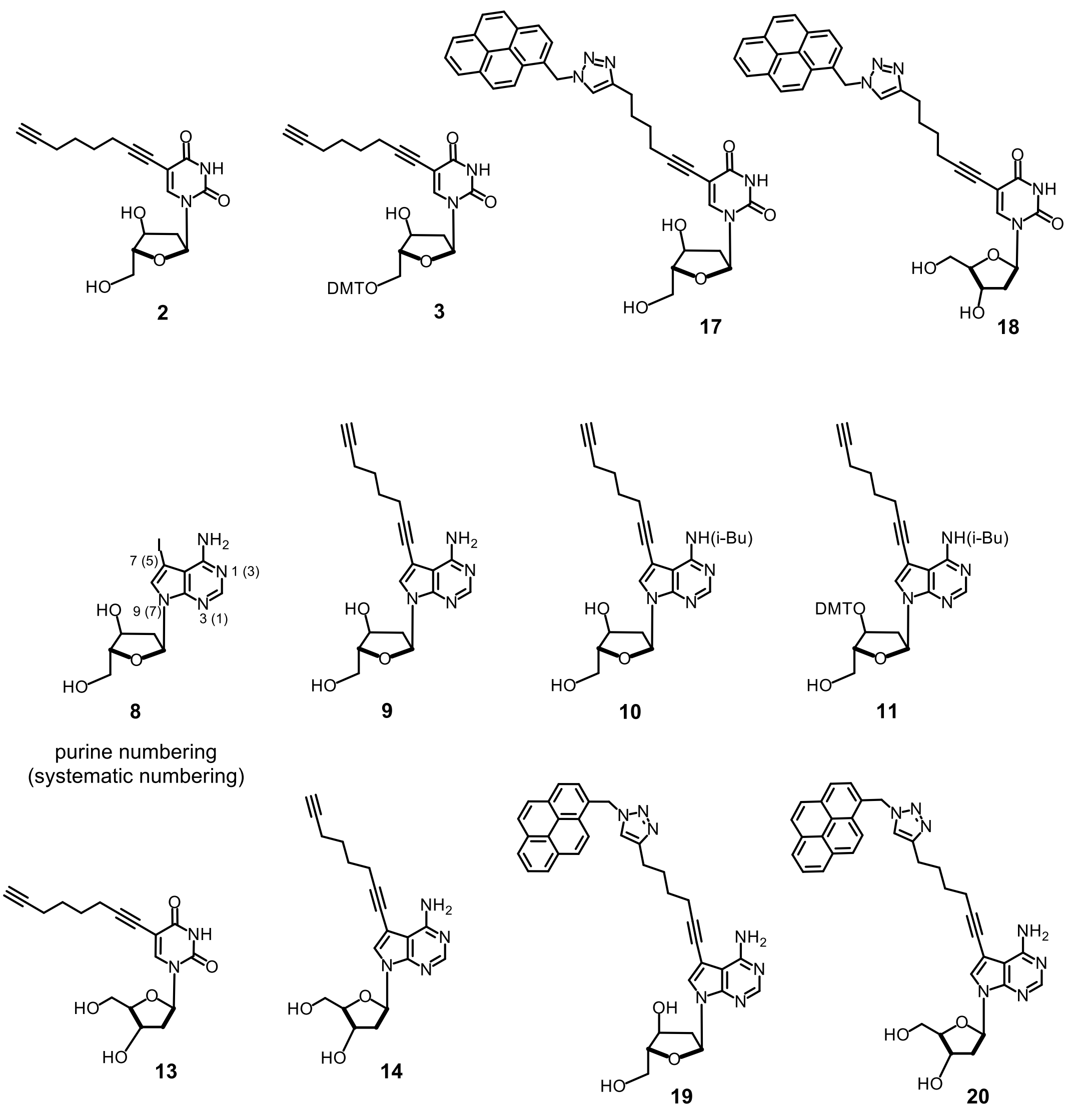

Figure S1. Structures of compounds used in this study.

Table S3. T_m values and thermodynamic data for single-stranded α-oligonucleotides.[a]

Oligonucleotide Single Strands	T_m [b] [°C]<br>H% [c]	$\Delta H°$<br>[kcal/mol]	$\Delta S°$<br>[cal/K mol]	$\Delta G°_{310}$<br>[kcal/mol]
α-5'-d(TAG GTC AAT ACT) (ODN-**4**)	No T_m	-	-	-
α-5'-d(ATC CAG TTA TGA) (ODN-**5**)	No T_m	-	-	-
α-5'-d(TCA TAA CTG GAT) (ODN-**1**)	45<br>9%	-35	-84	-8.4
α-5'-d(TCA TAA C**2**G GAT) (ODN-**2**)	45<br>6%	-35	-84	-9.3
α-5'-d(TCA TAA C**17**G GAT) (ODN-**3**)	47 [d]<br>6%	-38 [d]	-90 [d]	-10.2 [d]
α-5'-d(AGT ATT GAC CTA) (ODN-**6**)	45<br>6%	-34	-81	-9.3

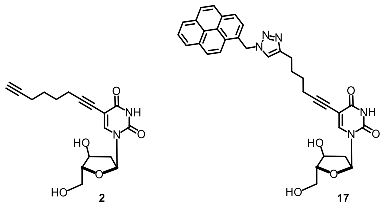

2 **17**

[a] Measured at 260 nm at a concentration of 5 µM + 5 µM single strand at a heating rate of 1.0°C/min in 100 mM NaCl, 10 mM $MgCl_2$, and 10 mM Na-cacodylate (pH 7.0). [b] T_m values were calculated from the heating curves using the program *Meltwin 3.0* [2]. [c] H% corresponds to hypochromicity in %. [d] For duplexes containing pyrene click adducts, a concentration of 2 µM + 2 µM single strand was used.

Mixing experiments of oligonucleotide duplexes

a)

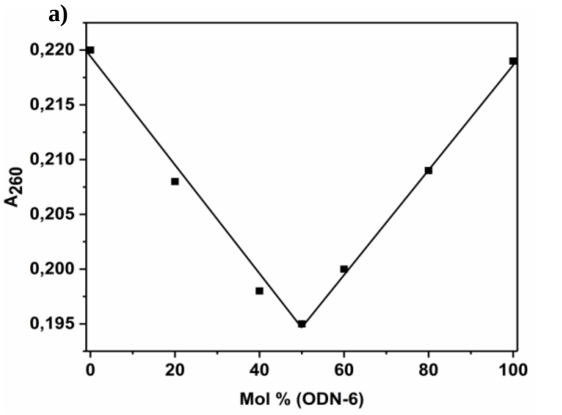

α-5’-d(TAG GTC AAT ACT) (ODN-**4**)
α-3’-d(ATC CAG TTA TGA) (ODN-**6**)

b)

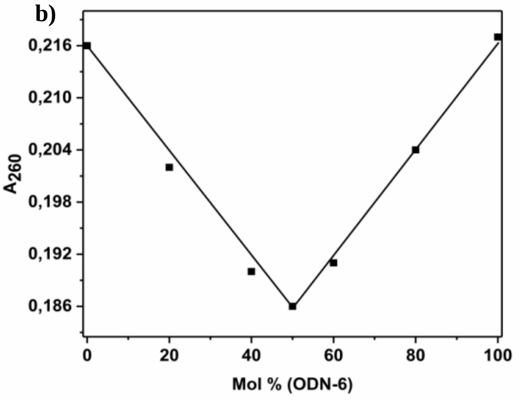

β-5’-d(TCA TAA CTG GAT) (ODN-**12**)
α-5’-d(ATC CAG TTA TGA) (ODN-**6**)

c)

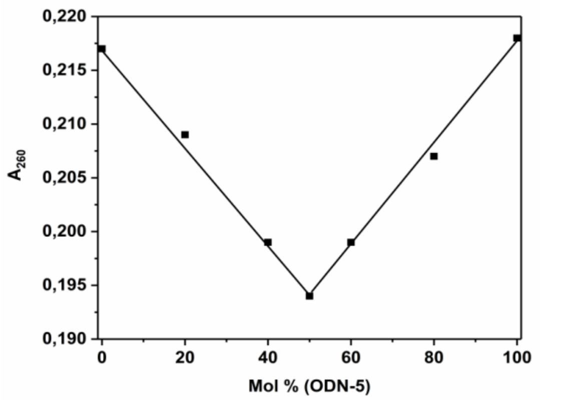

α-5’-d(TCA TAA CTG GAT) (ODN-**1**)
α-3’-d(AGT ATT GAC CTA) (ODN-**5**)

d)

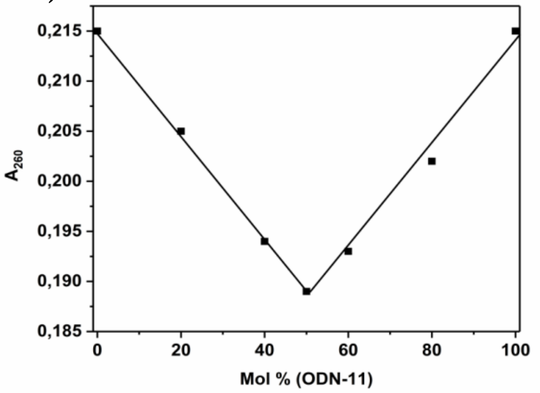

α-5’-d(TCA TAA CTG GAT) (ODN-**1**)
β-5’-d(AGT ATT GAC CTA) (ODN-**11**)

Figure S2. Mixing experiments of oligonucleotide duplexes. a) ODN-**4**•ODN-**6**, b) ODN-**12**•ODN-**6**, c) ODN-**1**•ODN-**5**, d) ODN-**1**•ODN-**11**. Experiments were performed at 260 nm at a single-strand concentration of 2 µM + 2 µM in 100 mM NaCl, 10 mM $MgCl_2$, and 10 mM Na-cacodylate (pH 7.0).

Reversed-phase (RP-18) HPLC elution profiles of purified oligonucleotides

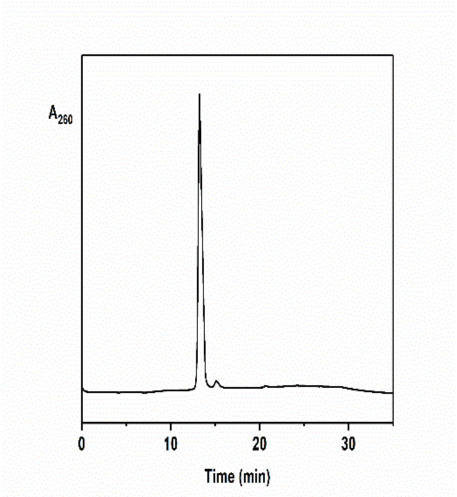

α-5‘-d(TCA TAA CTG GAT) (ODN-**1**)

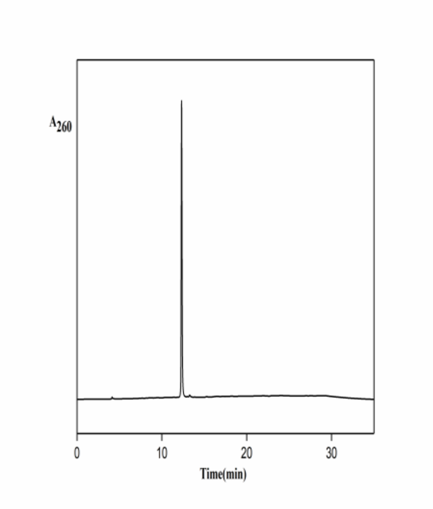

α-5‘-d(TCA TAA C2G GAT) (ODN-**2**)

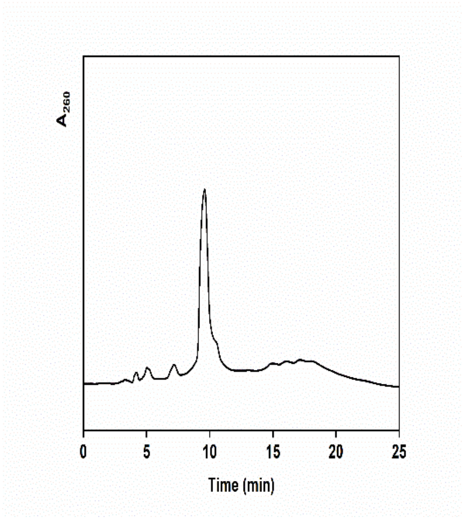

α-5‘-d(TCA TAA C17G GAT) (ODN-**3**)

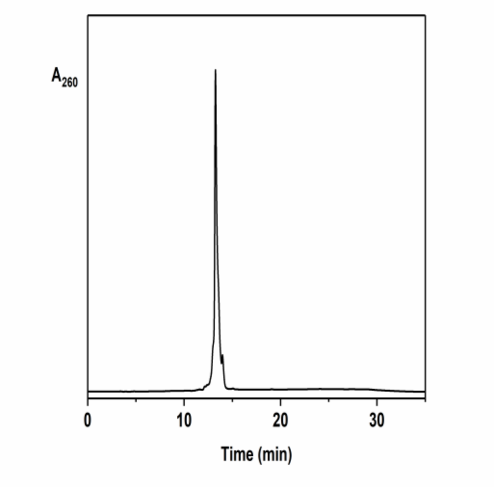

α-5‘-d-(TAG GTC AAT ACT) (ODN-**4**)

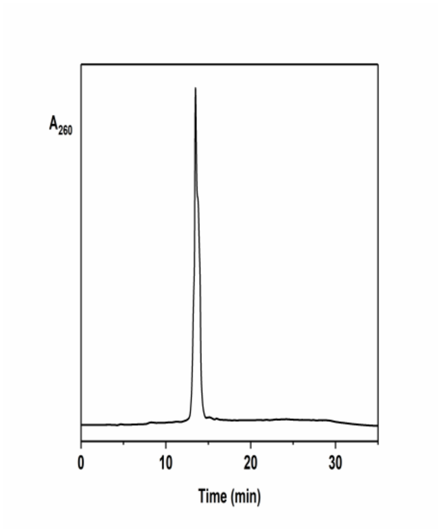

α-5‘-d(ATC CAG TTA TGA) (ODN-**5**)

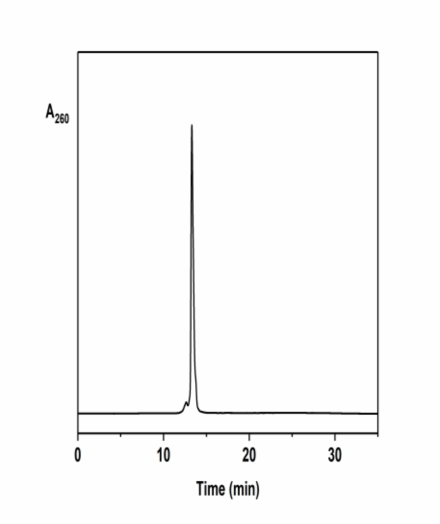

α-5‘-d(AGT ATT GAC CTA) (ODN-**6**)

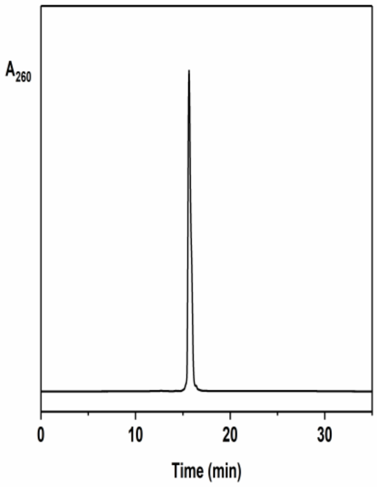

α-5'-d(ATC C**9**G TTA TGA) (ODN-**7**)

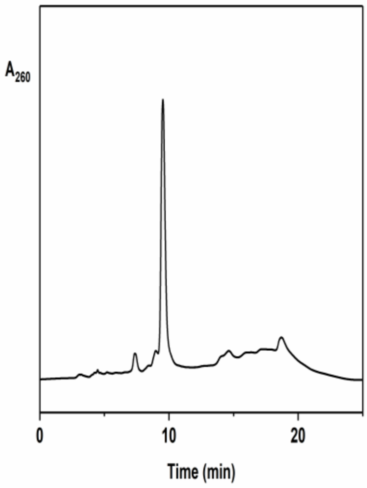

α-5'-d(ATC C**19**G TTA TGA) (ODN-**8**)

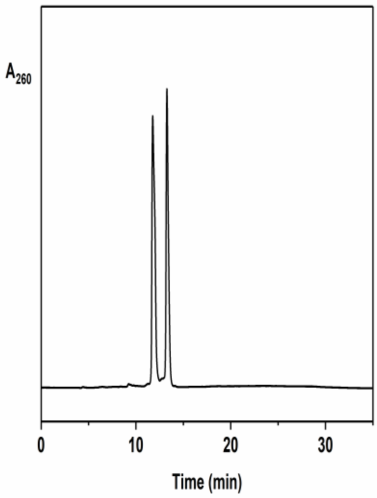

α-5'-d(CGC GAA TTC GCG) (ODN-**9**)*

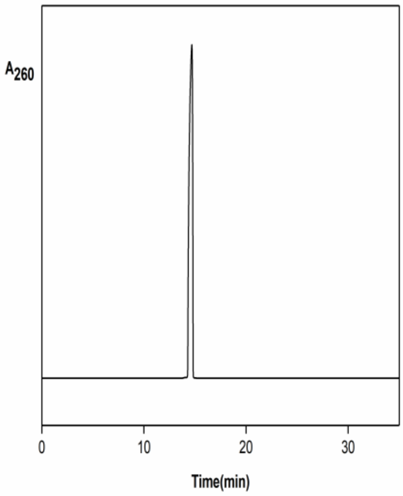

β-5'-d(TAG GTC AAT ACT) (ODN-**10**)

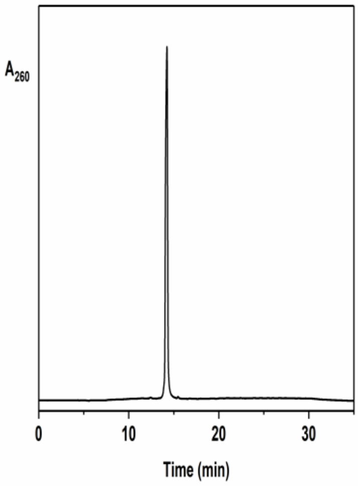

β-5'-d(AGT ATT CAG CTA) (ODN-**11**)

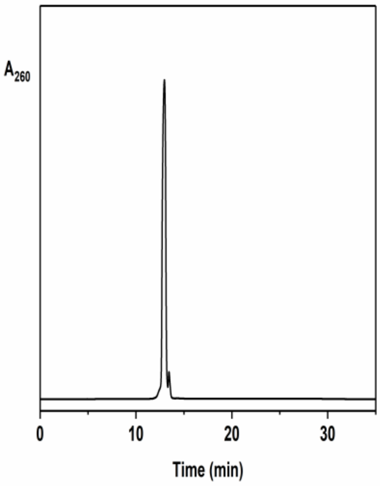

β-5'-d(TCA TAA CTG GAT) (ODN-**12**)

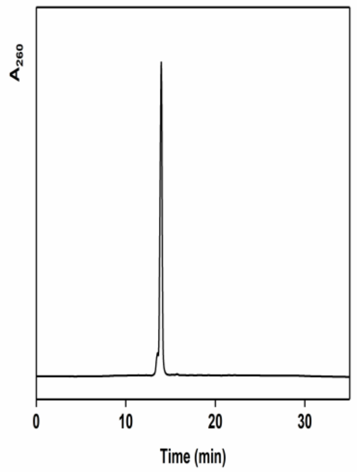

β-5’-d(ATC CAG TTA TGA) (ODN-**13**)

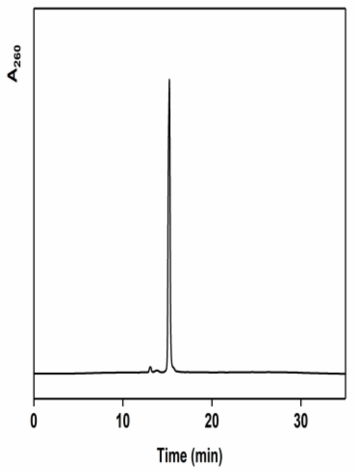

β-5‘-d(TAG G13C AAT ACT) (ODN-**14**)

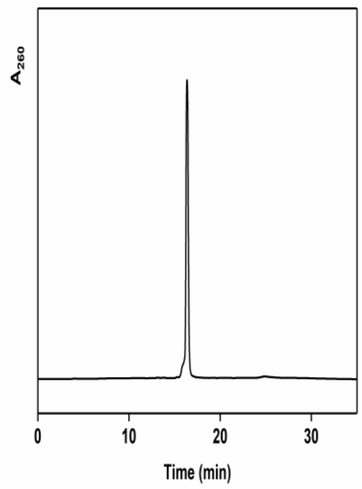

β-5‘-d(AGT ATT G14C CTA) (ODN-**15**)

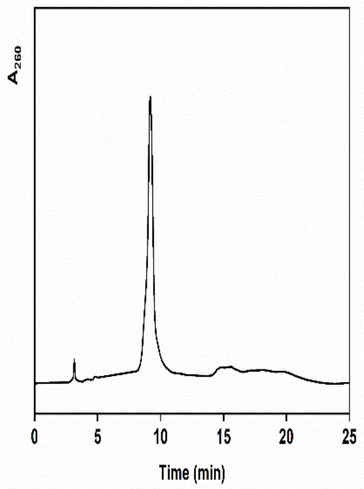

β-5‘-d(AGT ATT G20C CTA) (ODN-**16**)

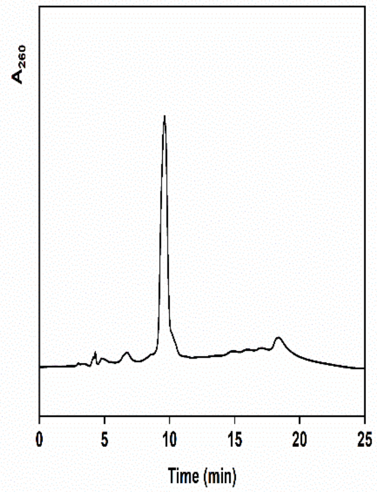

β-5‘-d(TAG G18C AAT ACT) (ODN-**17**)

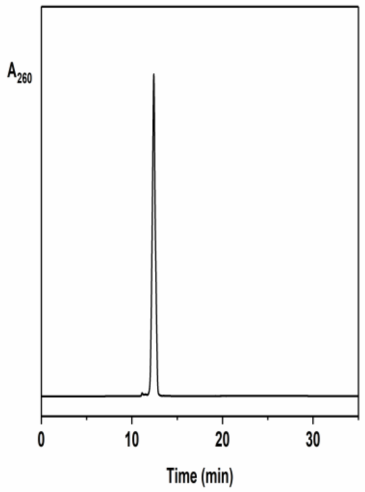

β-5’-d(CGC GAA TTC GCG) (ODN-**18**)

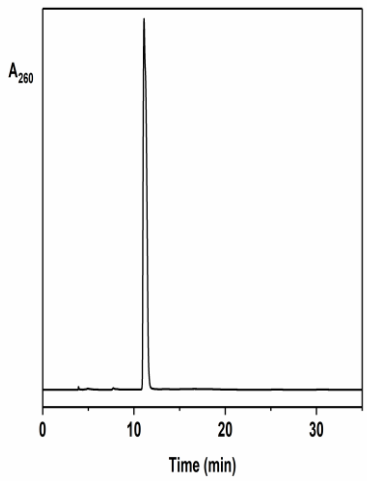

β-5’-d(TCA TAA C13G GAT) (ODN-**19**)

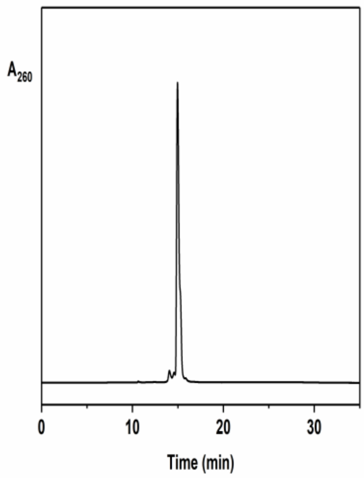

β-5’-d(ATC C14G TTA TGA) (ODN-**20**)

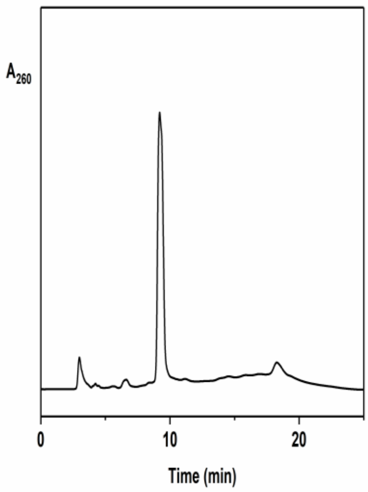

β-5’-d(TCA TAA C18G GAT) (ODN-**21**)

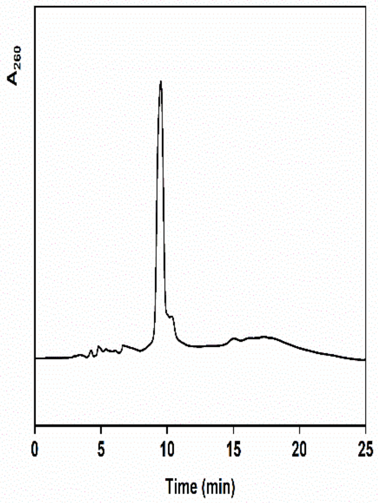

β-5’-d(ATC C20G TTA TGA) (ODN-**22**)

Figure S3. Reversed-phase (RP-18) HPLC elution profiles of purified oligonucleotides monitored at 260 nm. X-axis refers to retention time (min); Y-axis refers to UV absorbance at 260 nm, measured in mV. For elution, the following system was used: (A) MeCN, (B) 0.1 M $(Et_3NH)OAc$ (pH 7.0)/MeCN, 95:5; gradient: 0-20 min 0-20% A in B; 20-25 min, 20% A in B; flow rate 0.7 mL/min. For oligonucleotides containing click conjugates the following gradient was used: 0-10 min 0-20% A, 10-15 min 20-40% A in B, 15-20 min 40% A in B, 20-25 min 40-0% A in B with a flow rate of 0.7 mL/min. *Two peaks were found as this oligonucleotide is self-complementary.

Thermal denaturation curves

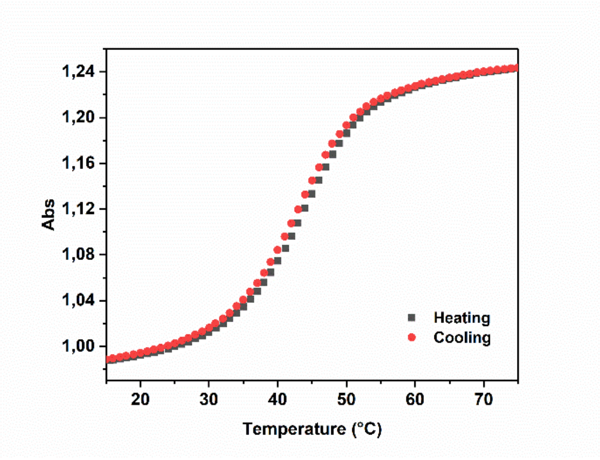

α-5‘-d(TCA TAA CTG GAT) (ODN-**1**)
β-5‘-d(AGT ATT GAC CTA) (ODN-**11**)

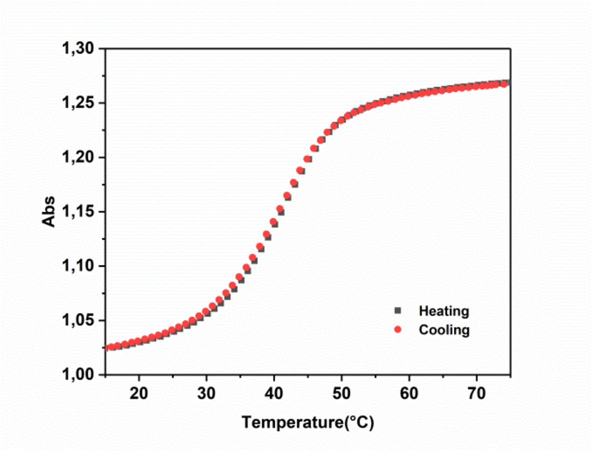

α-5‘-d(TCA TAA C**2**G GAT) (ODN-**2**)
β-5‘-d(AGT ATT GAC CTA) (ODN-**11**)

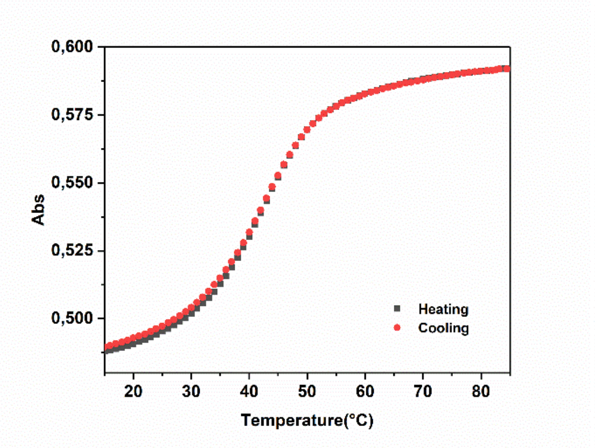

α-5‘-d(TCA TAA C**17**G GAT) (ODN-**3**)
β-5‘-d(AGT ATT GAC CTA) (ODN-**11**)

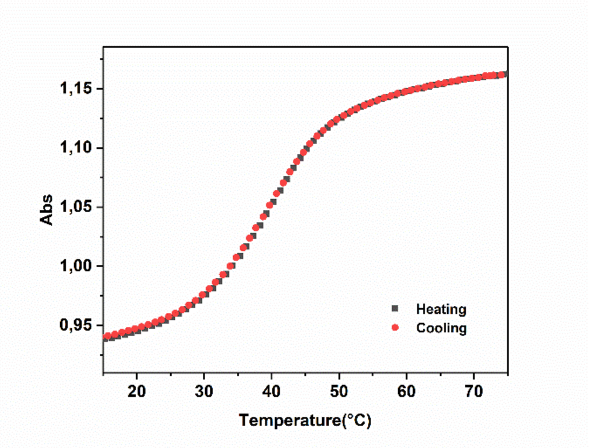

α-5‘-d(TCA TAA CTG GAT) (ODN-**1**)
β-5‘-d(AGT ATT G**14**C CTA) (ODN-**15**)

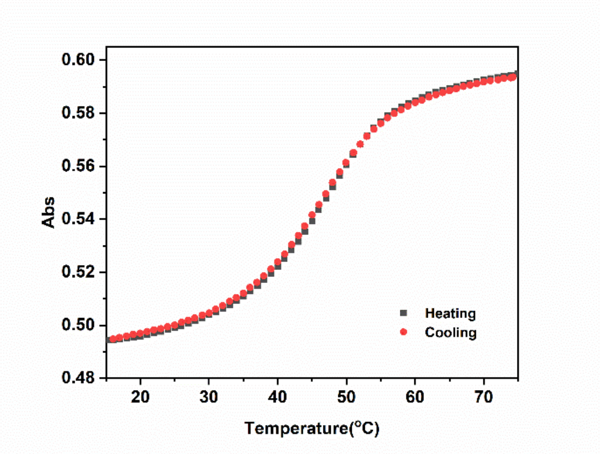

α-5‘-d(TCA TAA CTG GAT) (ODN-**1**)
β-5‘-d(AGT ATT G**20**C CTA) (ODN-**16**)

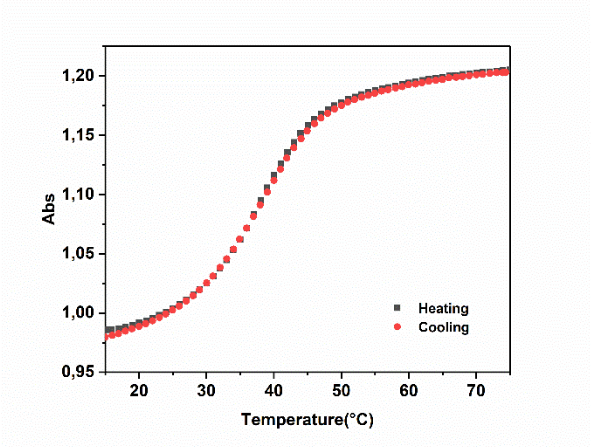

α-5‘-d(TCA TAA C **2** G GAT) (ODN-**2**)
β-5‘-d(AGT ATT G**14**C CTA) (ODN-**15**)

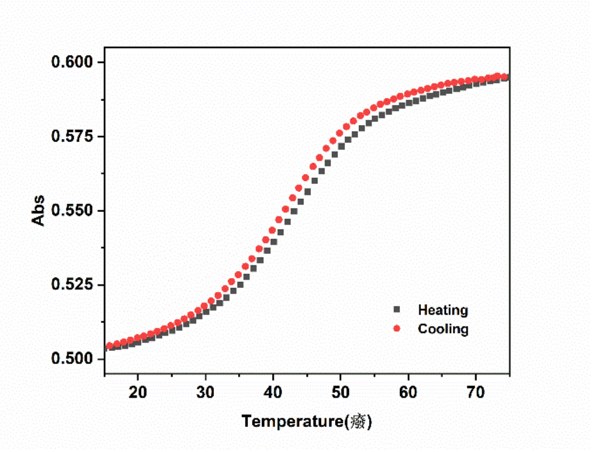

α-5‘-d(TCA TAA C17G GAT) (ODN-**3**)
β-5‘-d(AGT ATT G14C CTA) (ODN-**15**)

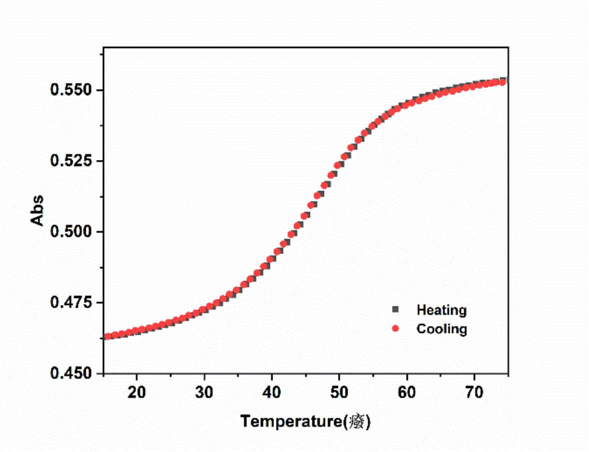

α-5‘-d(TCA TAA C2G GAT) (ODN-**2**)
β-5‘-d(AGT ATT G20C CTA) (ODN-**16**)

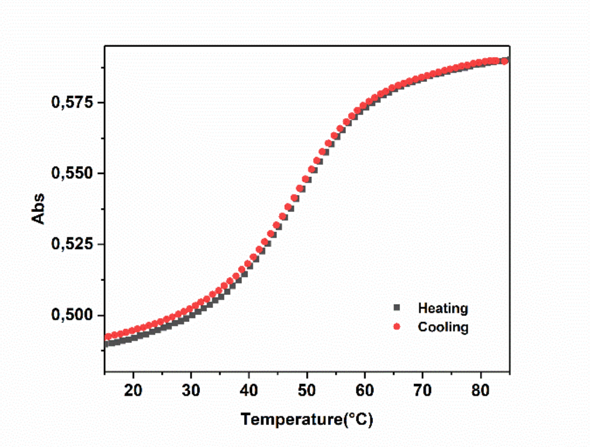

α-5‘-d(TCA TAA C17G GAT) (**ODN-3**)
β-5‘-d(AGT ATT G20C CTA) (**ODN-16**)

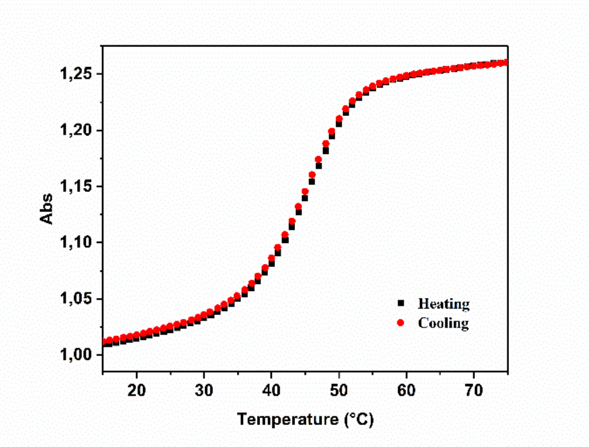

β-5’-d(TCA TAA CTG GAT) (ODN-**12**)
β-3’-d(AGT ATT GAC CTA) (ODN-**13**)

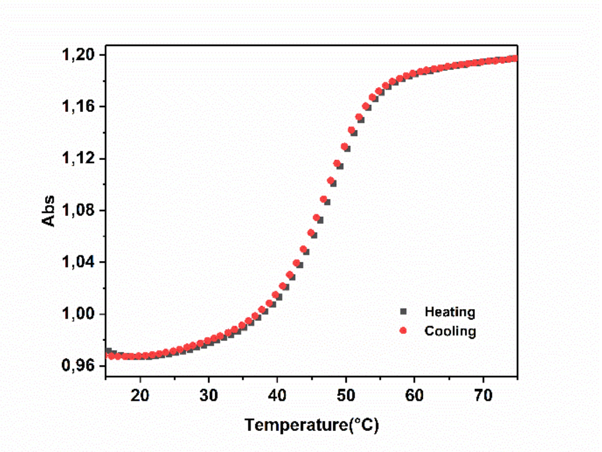

β-5’-d(TCA TAA C13G GAT) (ODN-**19**)
β-3’-d(AGT ATT GAC CTA) (ODN-**13**)

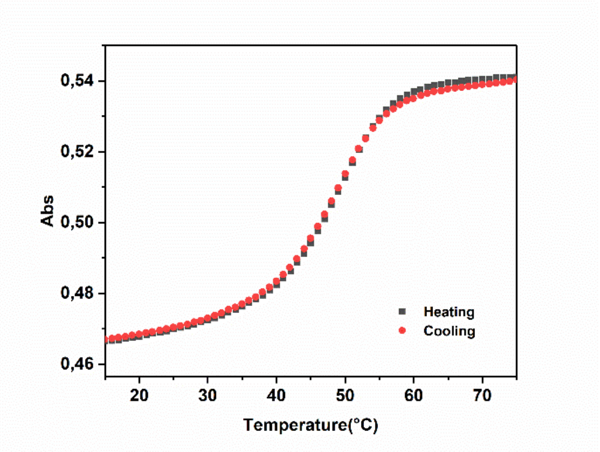

β-5’-d(TCA TAA C18G GAT) (ODN-**21**)
β-3’-d(AGT ATT GAC CTA) (ODN-**13**)

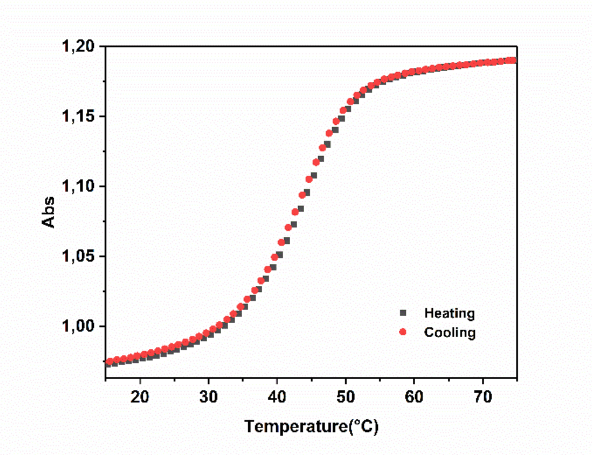

β-5’-d(TCA TAA CTG GAT) (ODN-**12**)
β-3’-d(AGT ATT G**14**C CTA) (ODN-**20**)

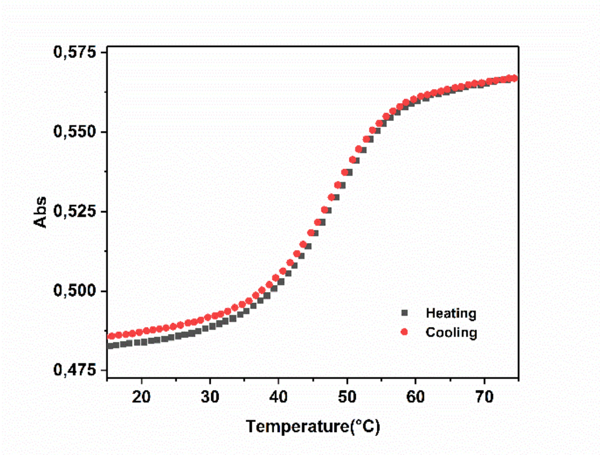

β-5’-d(TCA TAA CTG GAT) (ODN-**12**)
β-3’-d(AGT ATT G**20**C CTA) (ODN-**22**)

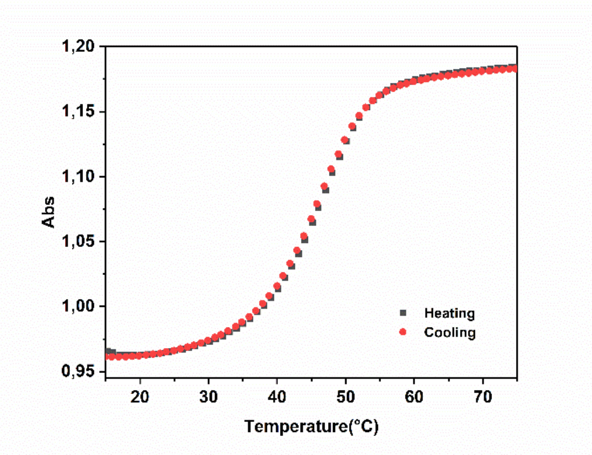

β-5’-d(TCA TAA C**13**G GAT) (ODN-**19**)
β-3’-d(AGT ATT G**14**C CTA) (ODN-**20**)

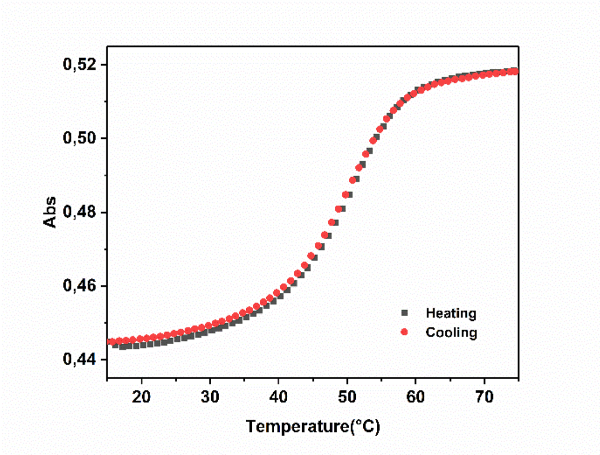

β-5’-d(TCA TAA C**18**G GAT) (ODN-**21**)
β-3’-d(AGT ATT G**14**C CTA) (ODN-**20**)

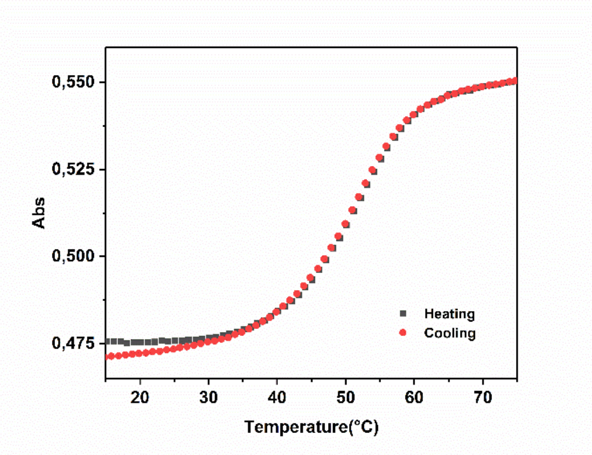

β-5’-d(TCA TAA C**13**G GAT) (ODN-**19**)
β-3’-d(AGT ATT G**20**C CTA) (ODN-**22**)

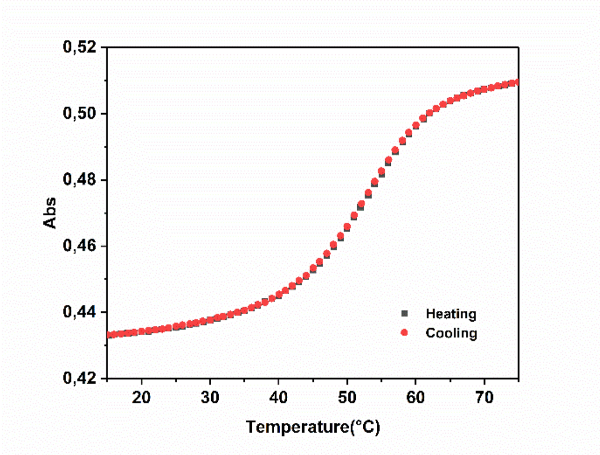

β-5’-d(TCA TAA C**18**G GAT) (ODN-**21**)
β-3’-d(AGT ATT G**20**C CTA) (ODN-**22**)

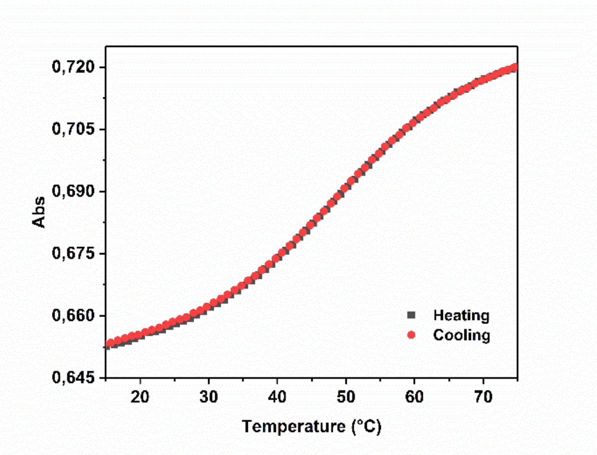

α-5‘-d(TCA TAA CTG GAT) (ODN-**1**)

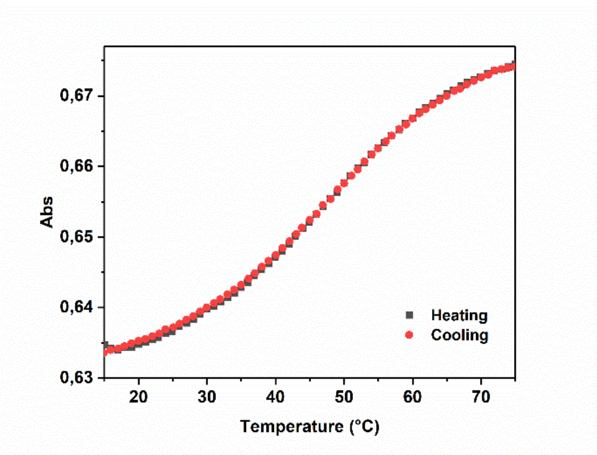

α-5‘-d(TCA TAA C**2**G GAT) (ODN-**2**)

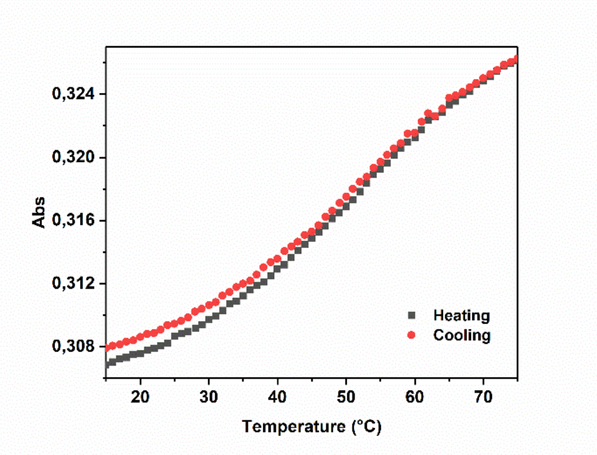

α-5‘-d(TCA TAA C**17**G GAT) (ODN-**3**)

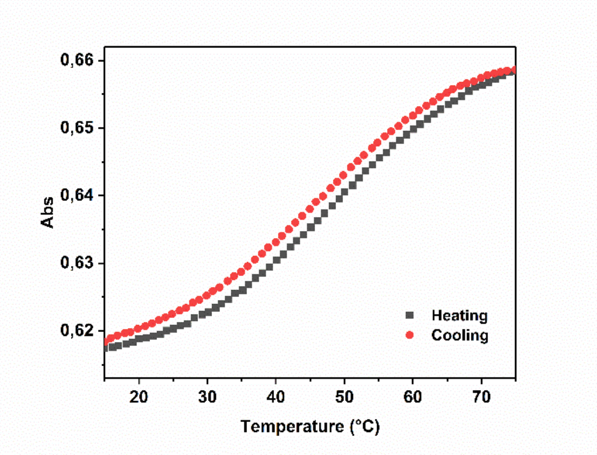

α-5’-d(AGT ATT GAC CTA) (ODN-**6**)

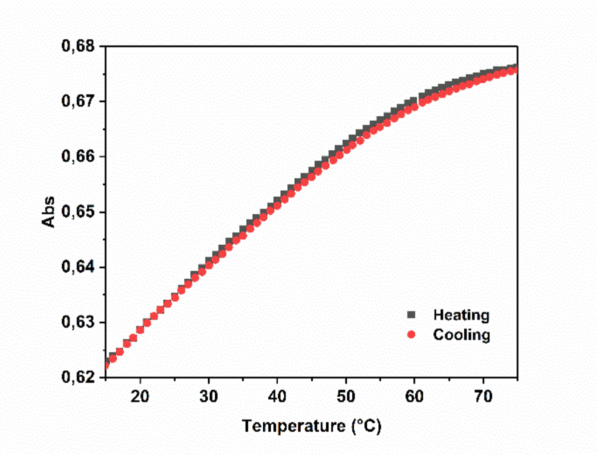

α-3’-d(AGT ATT GAC CTA) (ODN-**5**)

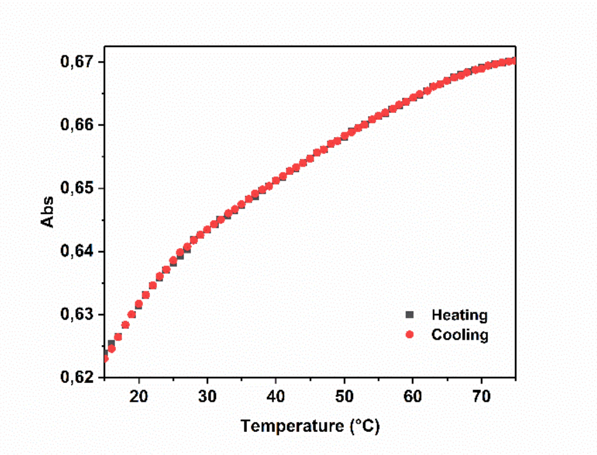

α-5’-d(TAG GTC AAT ACT) (ODN-**4**)

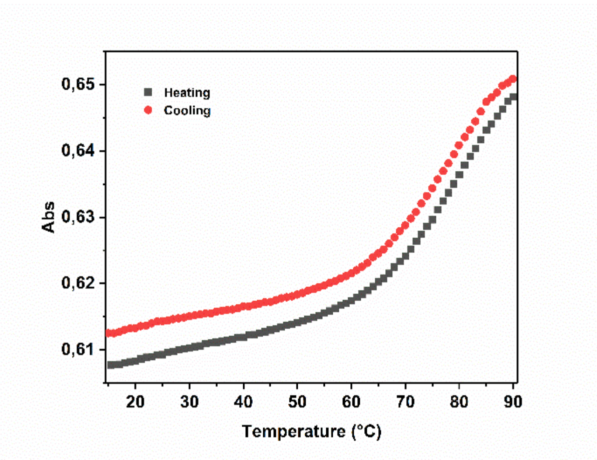

α-5’-d(CGC GAA TTC GCG) (ODN-**9**)

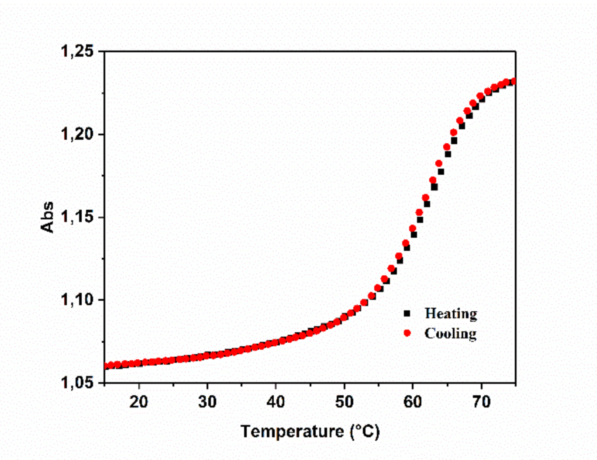

α-5’-d(TAG GTC AAT ACT) (ODN-**4**)
α-3’-d(ATC CAG TTA TGA) (ODN-**6**)

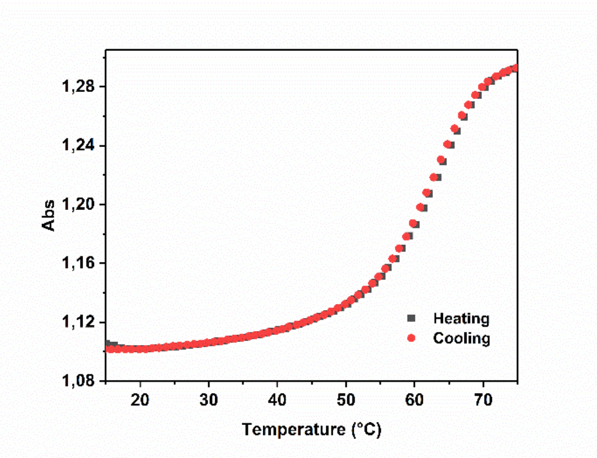

α-5’-d(TCA TAA C**T**G GAT) (ODN-**1**)
α-3’-d(AGT ATT G**A**C CTA) (ODN-**5**)

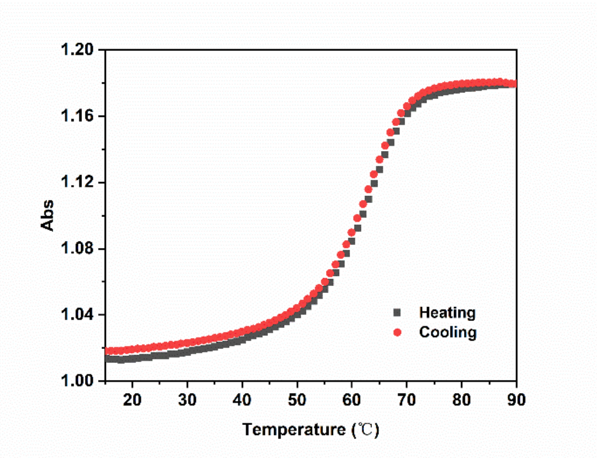

α-5‘-d(TCA TAA C**2**G GAT) (ODN-**2**)
α-3’-d(AGT ATT G**A**C CTA) (ODN-**5**)

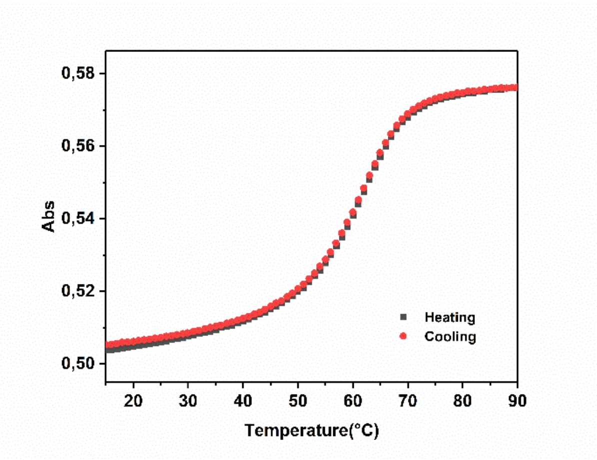

α-5‘-d(TCA TAA C**17**G GAT) (ODN-**3**)
α-3’-d(AGT ATT G**A**C CTA) (ODN-**5**)

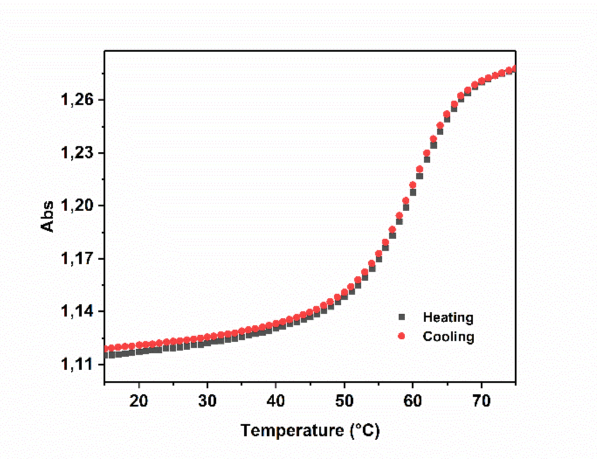

α-5’-d(TCA TAA C**T**G GAT) (ODN-**1**)
α-3’-d(AGT ATT G**9**C CTA) (ODN-**7**)

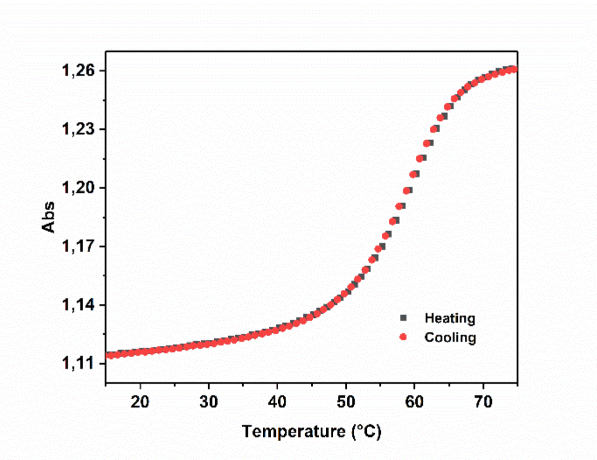

α-5‘-d(TCA TAA C2G GAT) (ODN-**2**)
α-3’-d(AGT ATT G9C CTA) (ODN-**7**)

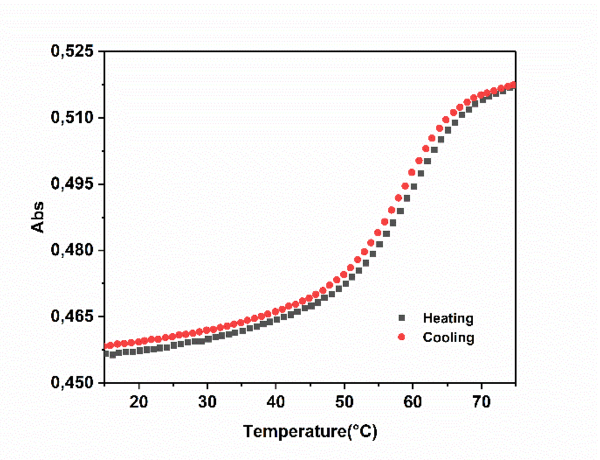

α-5‘-d(TCA TAA C17G GAT) (ODN-**3**)
α-3’-d(AGT ATT G9C CTA) (ODN-**7**)

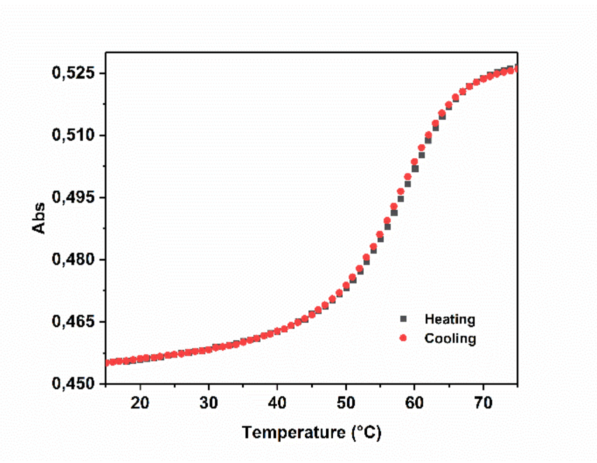

α-5’-d(TCA TAA CTG GAT) (ODN-**1**)
α-3’-d(AGT ATT G19C CTA) (ODN-**8**)

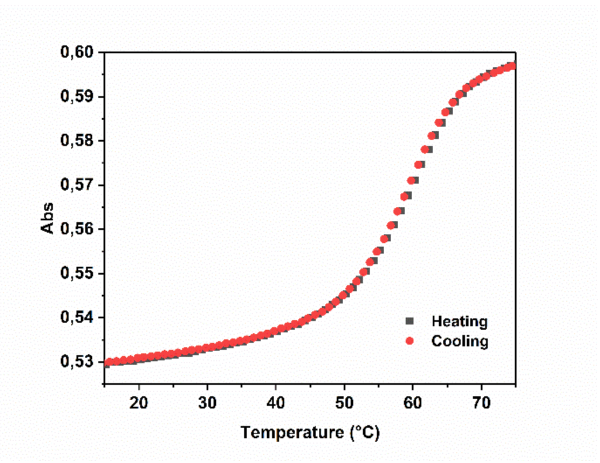

α-5‘-d(TCA TAA C2G GAT) (ODN-**2**)
α-3’-d(AGT ATT G19C CTA) (ODN-**8**)

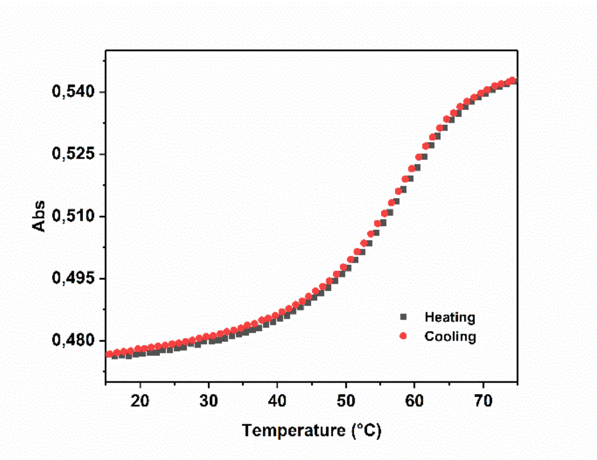

α-5’-d(TCA TAA C17G GAT) (ODN-**3**)
α-3’-d(AGT ATT G19C CTA) (ODN-**8**)

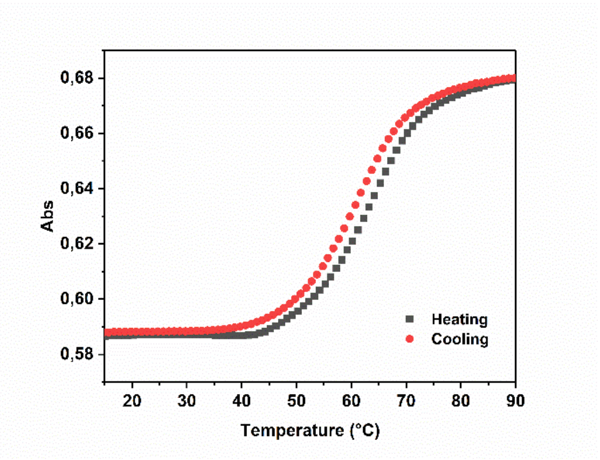

β-5’-d(CGC GAA TTC GCG) (ODN-**18**)

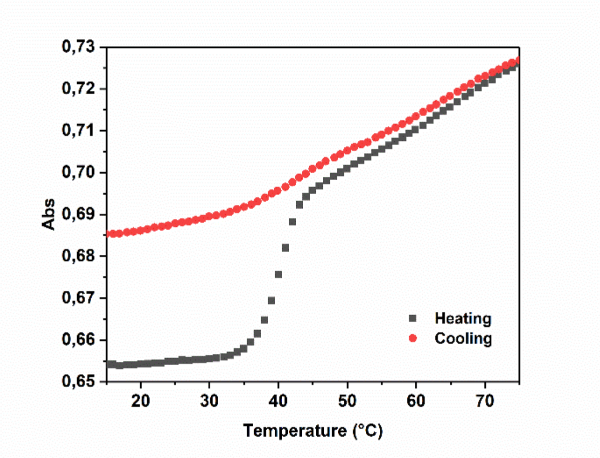

β-5’-d(CGC GAA TTC GCG) (ODN-**18**)*

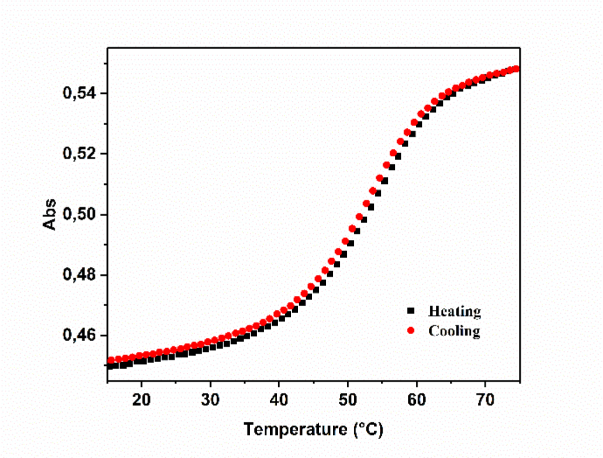

β-5’-d(TAG G**18**C AAT ACT) (ODN-**17**)
α-5’-d(ATC C**19**G TTA TGA) (ODN-**8**)

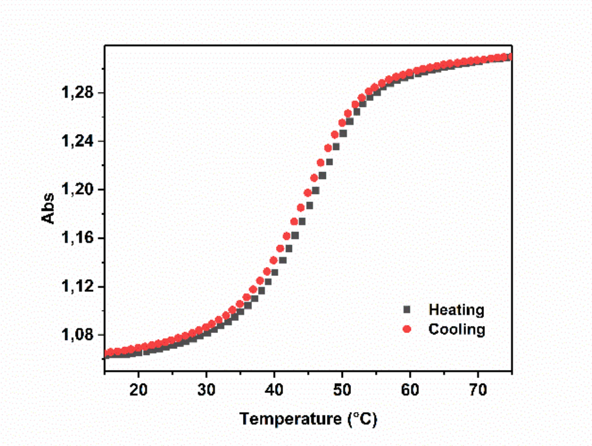

β-5’-d(TAG G**T**C AAT ACT) (ODN-**10**)
α-5’-d(ATC C**A**G TTA TGA) (ODN-**5**)

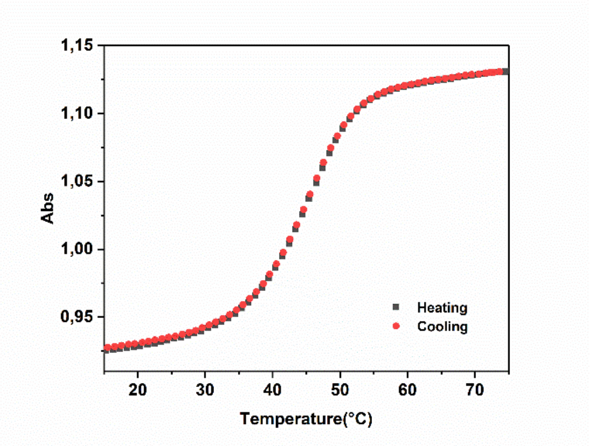

β-5’-d(TAG G**13**C AAT ACT) (ODN-**14**)
α-5’-d(ATC C**A**G TTA TGA) (ODN-**5**)

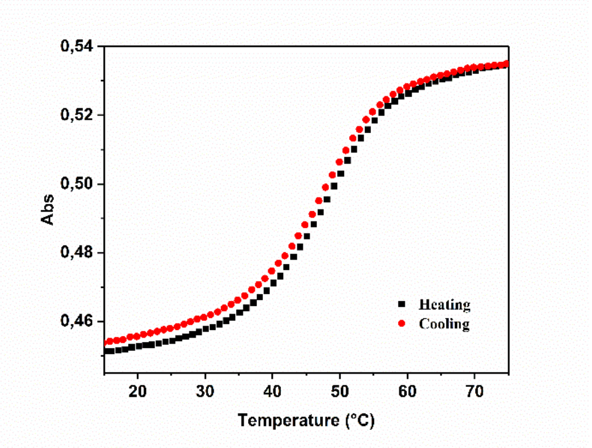

β-5’-d(TAG G**18**C AAT ACT) (ODN-**17**)
α-5’-d(ATC C**A**G TTA TGA) (ODN-**5**)

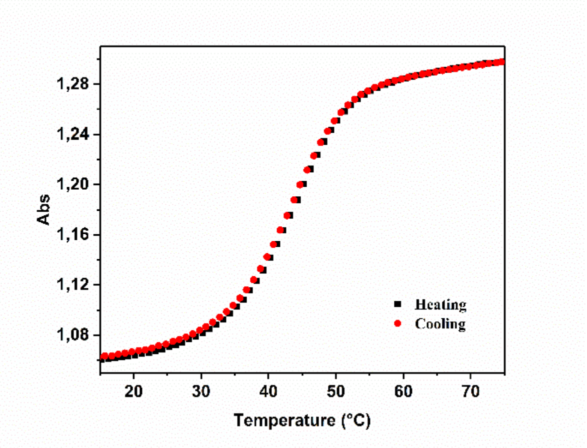

β-5’-d(TAG G**T**C AAT ACT) (ODN-**10**)
α-5’-d(ATC C**9**G TTA TGA) (ODN-**7**)

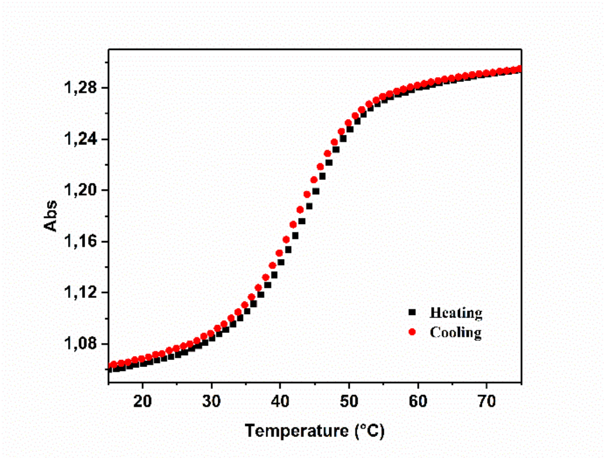

β-5’-d(TAG G**13**C AAT ACT) (ODN-**14**)
α-5’-d(ATC C**9**G TTA TGA) (ODN-**7**)

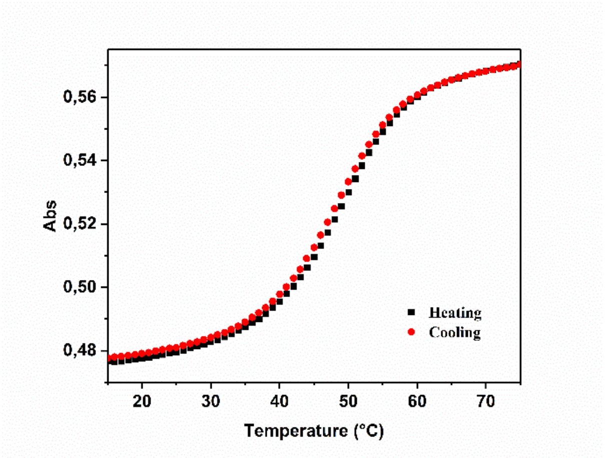

β-5’-d(TAG G**18**C AAT ACT) (ODN-**17**)
α-5’-d(ATC C**9**G TTA TGA) (ODN-**7**)

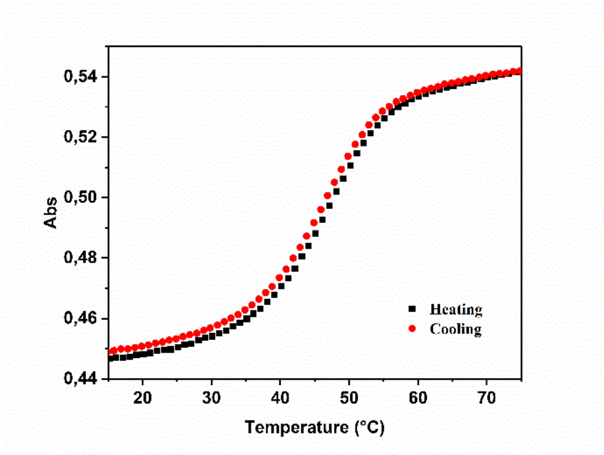

β-5’-d(TAG G**T**C AAT ACT) (ODN-**10**)
α-5’-d(ATC C**19**G TTA TGA) (ODN-**8**)

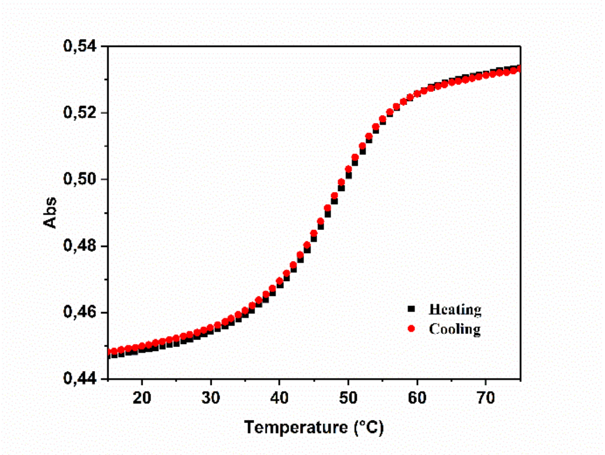

β-5’-d(TAG G**13**C AAT ACT) (ODN-**14**)
α-5’-d(ATC C**19**G TTA TGA) (ODN-**8**)

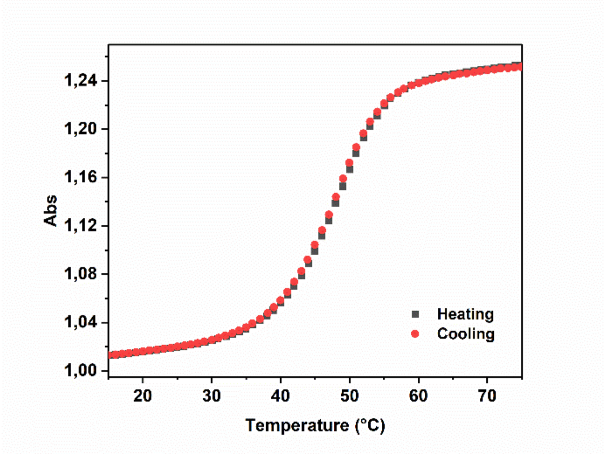

β-5‘-d(TAG G**T**C AAT ACT) (ODN-**10**)
β-3‘-d(ATC C**A**G TTA TGA) (ODN-**11**)

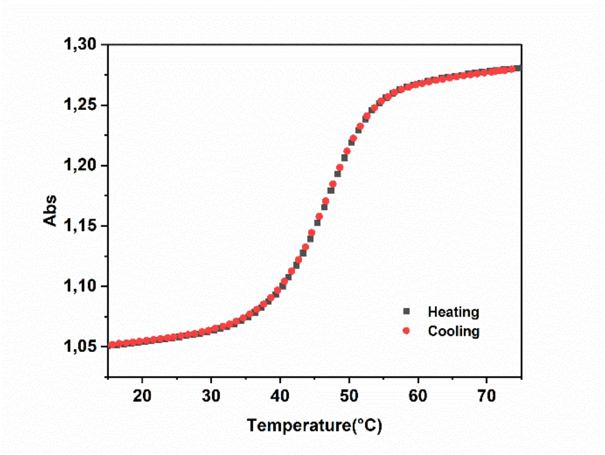

β-5‘-d(TAG G**13**C AAT ACT) (ODN-**14**)
β-3‘-d(ATC C**A**G TTA TGA) (ODN-**11**)

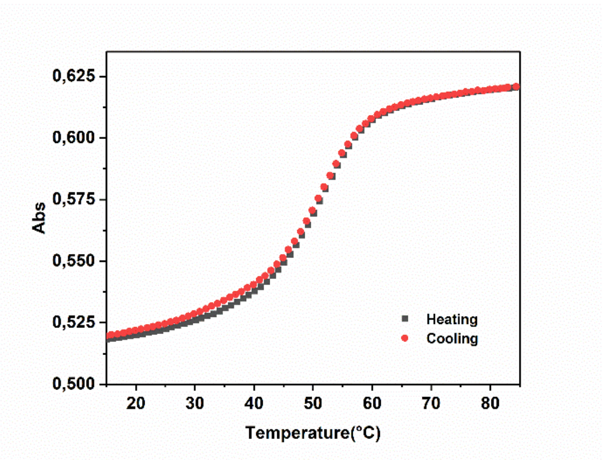

β-5‘-d(TAG G**18**C AAT ACT) (ODN-**17**)
β-3‘-d(ATC C**A**G TTA TGA) (ODN-**11**)

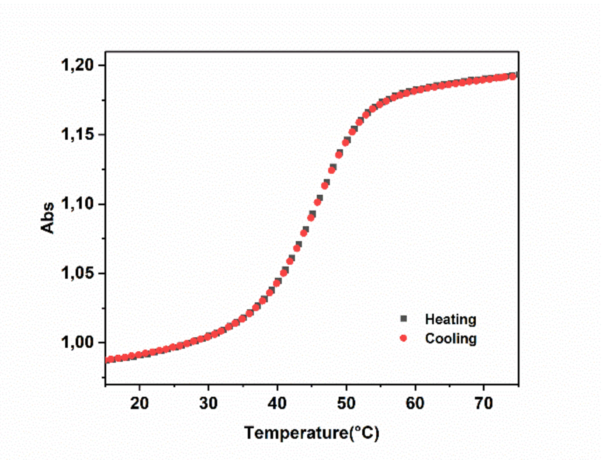

β-5‘-d(TAG G**T**C AAT ACT) (ODN-**10**)
β-3‘-d(ATC C**14**G TTA TGA) (ODN-**15**)

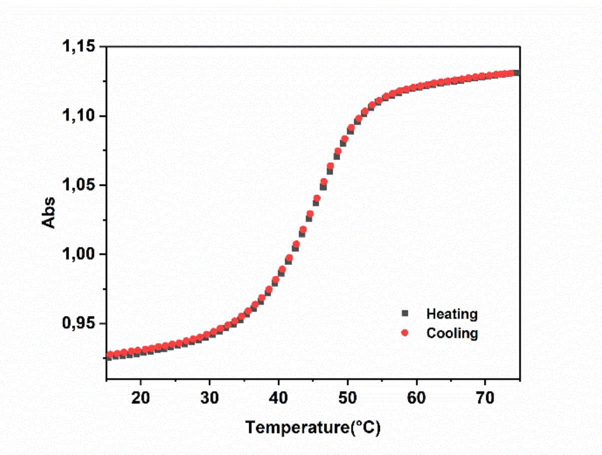

β-5‘-d(TAG G**13**C AAT ACT) (ODN-**14**)
β-3‘-d(ATC C**14**G TTA TGA) (ODN-**15**)

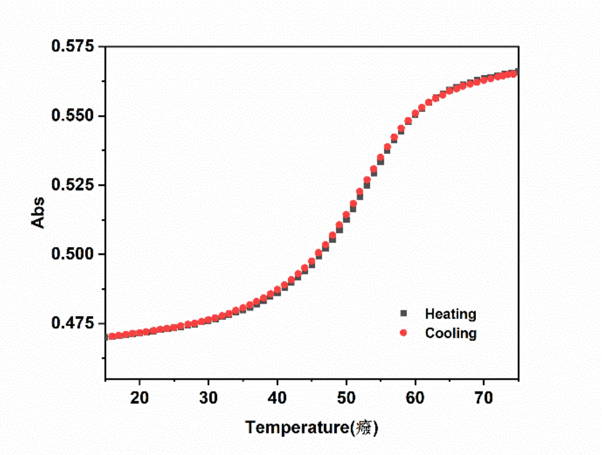

β-5‘-d(TAG G**18**C AAT ACT) (ODN-**17**)
β-3‘-d(ATC C**14**G TTA TGA) (ODN-**15**)

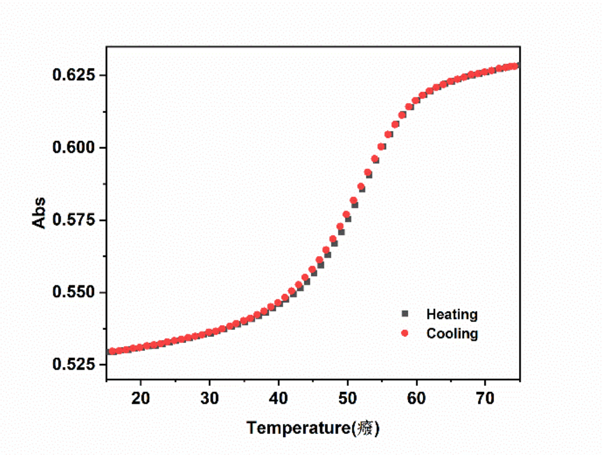

β-5‘-d(TAG G**T**C AAT ACT) (ODN-**10**)
β-3‘-d(ATC C**20**G TTA TGA) (ODN-**16**)

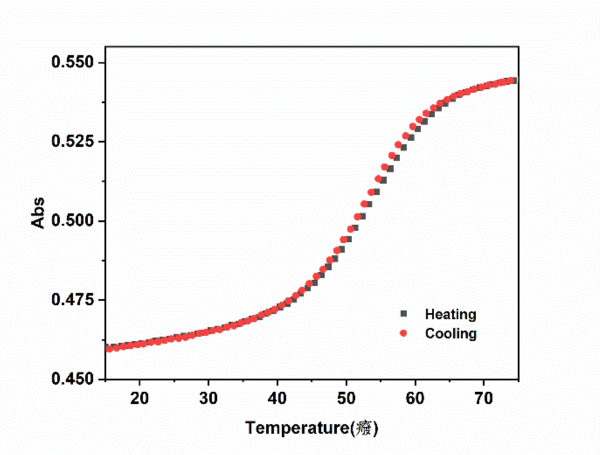

β-5‘-d(TAG G**13**C AAT ACT) (ODN-**14**)
β-3‘-d(ATC C**20**G TTA TGA) (ODN-**16**)

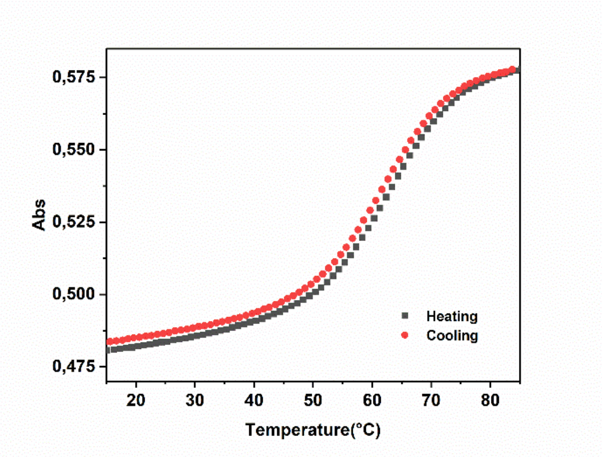

β-5‘-d(TAG G**18**C AAT ACT) (ODN-**17**)
β-3‘-d(ATC C**20**G TTA TGA) (ODN-**16**)

Figure S4. Thermal denaturation curves obtained from heating (black) and cooling (red) experiments monitored at 260 nm. All the experiments were performed with 5 µM duplex concentration at a heating rate of 1.0 °C/min in 100 mM NaCl, 10 mM $MgCl_2$, 10 mM Na-cacodylate (pH 7.0) buffer. For oligonucleotides containing click conjugates, the melting experiment was performed with 2 µM duplex concentration. *Measured in 10 mM NaCl, 10 mM NaH_2PO_4, 0.1 mM EDTA (pH 7).

CD-spectra of oligonucleotide duplexes

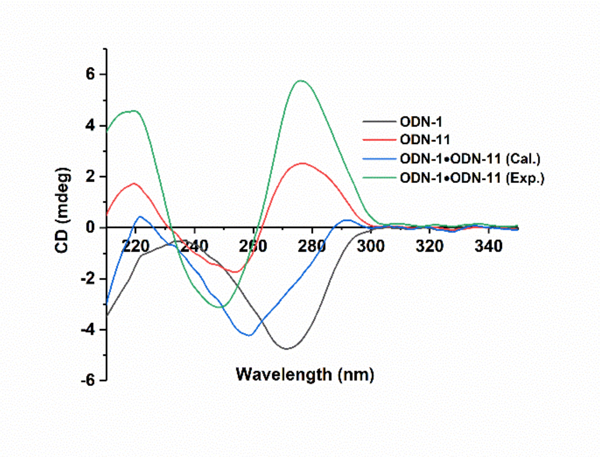

α-5‘-d(TCA TAA CTG GAT) (ODN-**1**)
β-5‘-d(AGT ATT GAC CTA) (ODN-**11**)

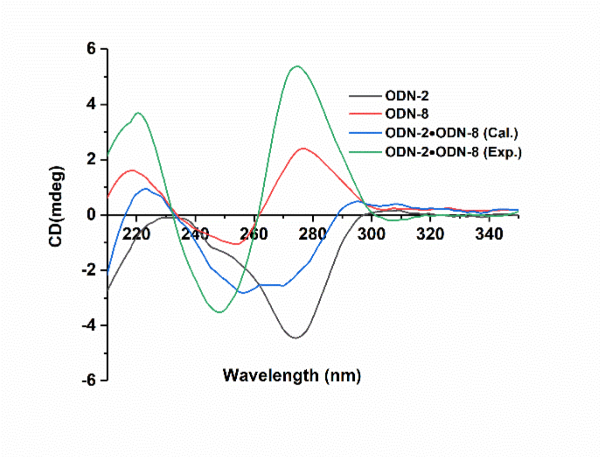

α-5‘-d(TCA TAA C**2**G GAT) (ODN-**2**)
β-5‘-d(AGT ATT GAC CTA) (ODN-**11**)

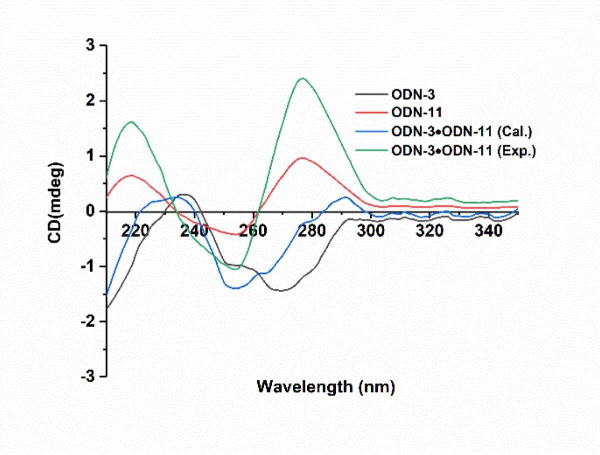

α-5‘-d(TCA TAA C**17**G GAT) (ODN-**3**)
β-5‘-d(AGT ATT GAC CTA) (ODN-**11**)

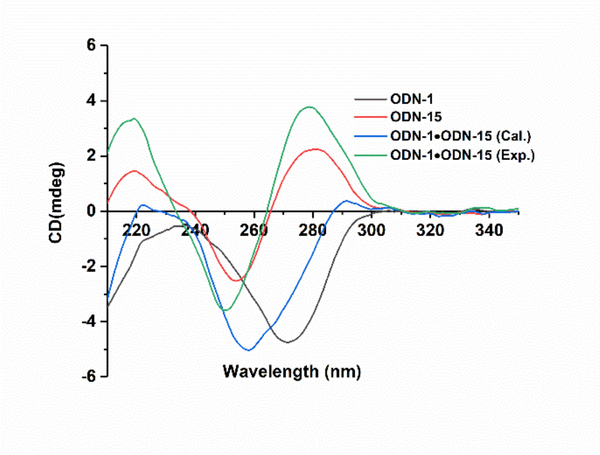

α-5‘-d(TCA TAA CTG GAT) (ODN-**1**)
β-5‘-d(AGT ATT G**14**C CTA) (ODN-**15**)

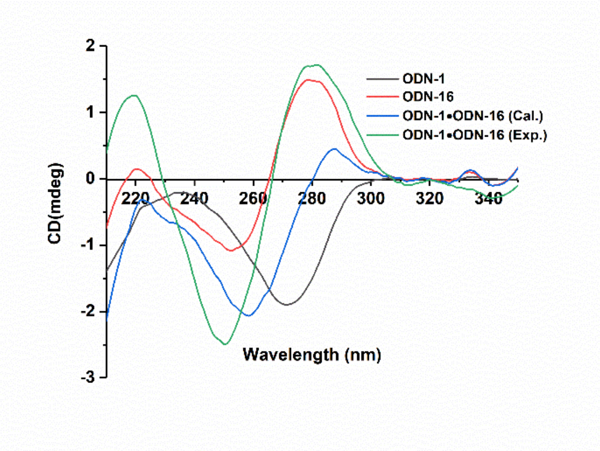

α-5‘-d(TCA TAA CTG GAT) (ODN-**1**)
β-5‘-d(AGT ATT G**20**C CTA) (ODN-**16**)

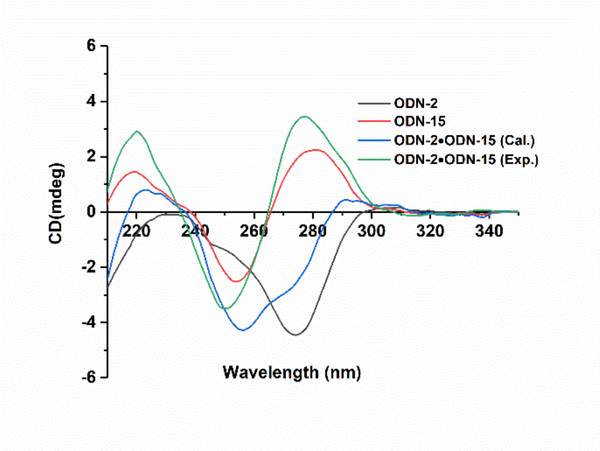

α-5‘-d(TCA TAA C**2**G GAT) (ODN-**2**)
β-5‘-d(AGT ATT G**14**C CTA) (ODN-**15**)

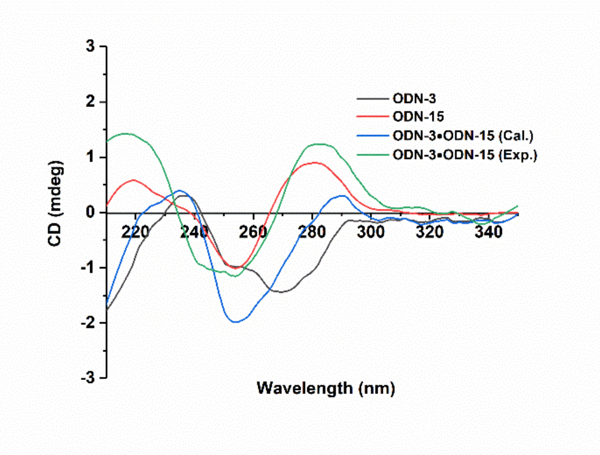

α-5‘-d(TCA TAA C17G GAT) (ODN-**3**)
β-5‘-d(AGT ATT G14C CTA) (ODN-**15**)

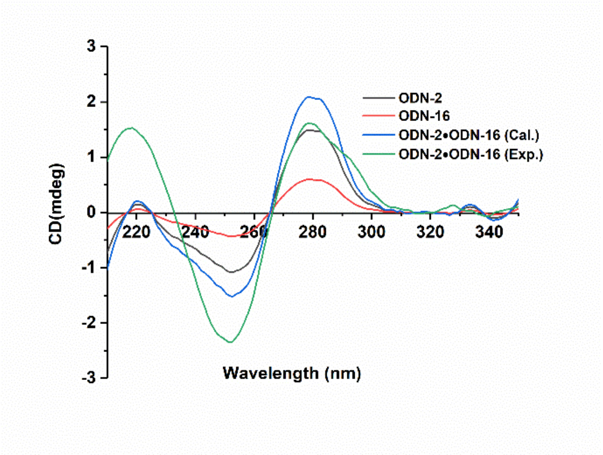

α-5‘-d(TCA TAA C2G GAT) (ODN-**2**)
β-5‘-d(AGT ATT G20C CTA) (ODN-**16**)

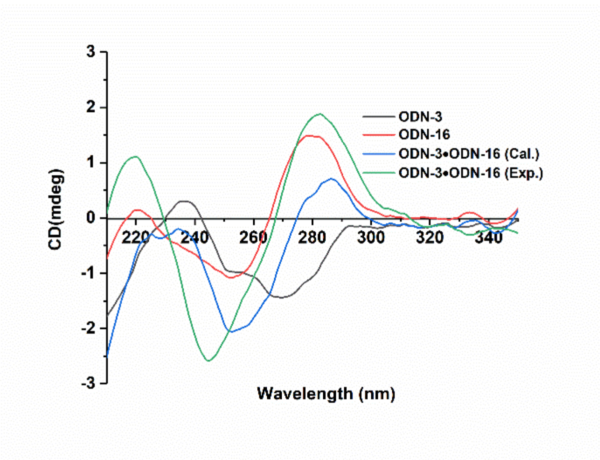

α-5‘-d-(TCA TAA C17G GAT) (ODN-**3**)
β-5‘-d-(AGT ATT G20C CTA) (ODN-**16**)

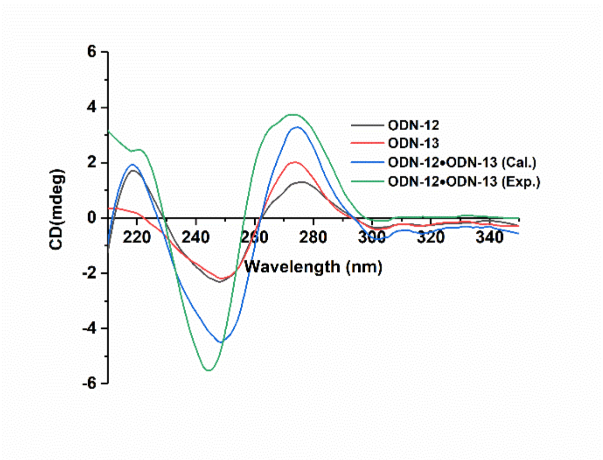

β-5’-d(TCA TAA CTG GAT) (ODN-**12**)
β-3’-d(AGT ATT GAC CTA) (ODN-**13**)

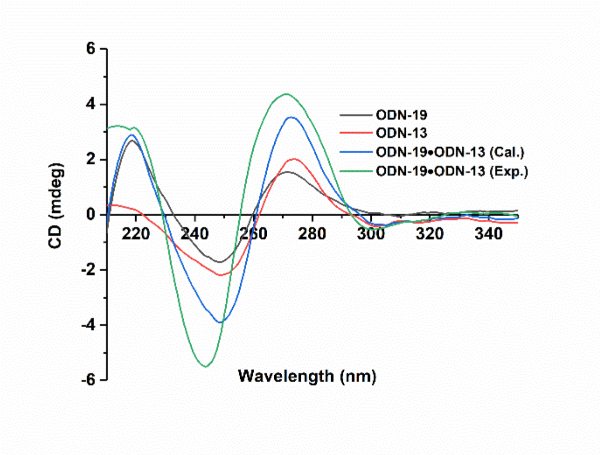

β-5’-d(TCA TAA C13G GAT) (ODN-**19**)
β-3’-d(AGT ATT GAC CTA) (ODN-**13**)

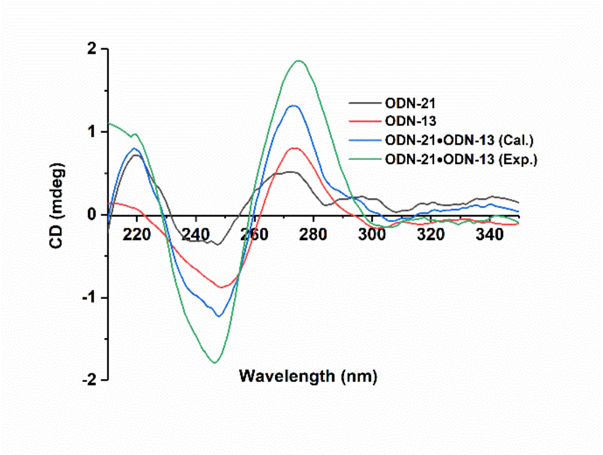

β-5’-d(TCA TAA C18G GAT) (ODN-**21**)
β-3’-d(AGT ATT GAC CTA) (ODN-**13**)

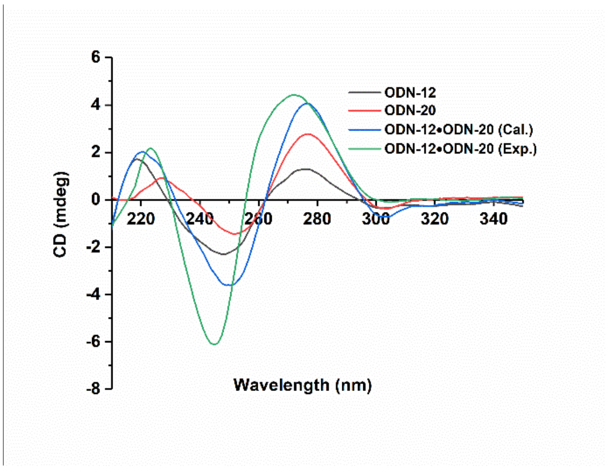

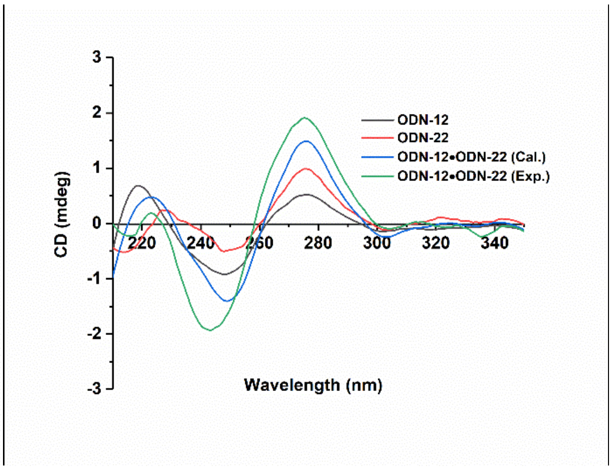

β-5’-d(TCA TAA C**T**G GAT) (ODN-**12**)
β-3’-d(AGT ATT G**14**C CTA) (ODN-**20**)

β-5’-d(TCA TAA C**T**G GAT) (ODN-**12**)
β-3’-d(AGT ATT G**20**C CTA) (ODN-**22**)

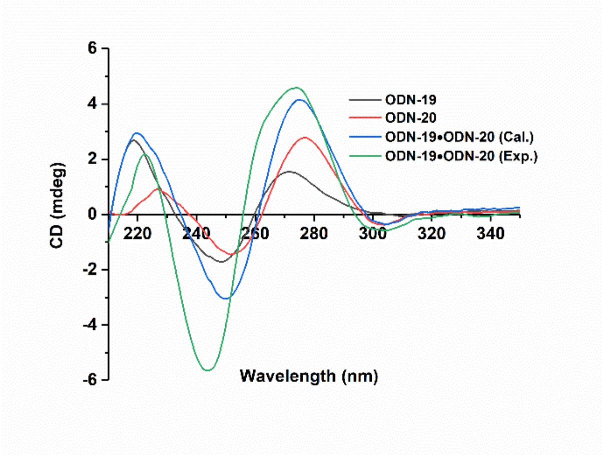

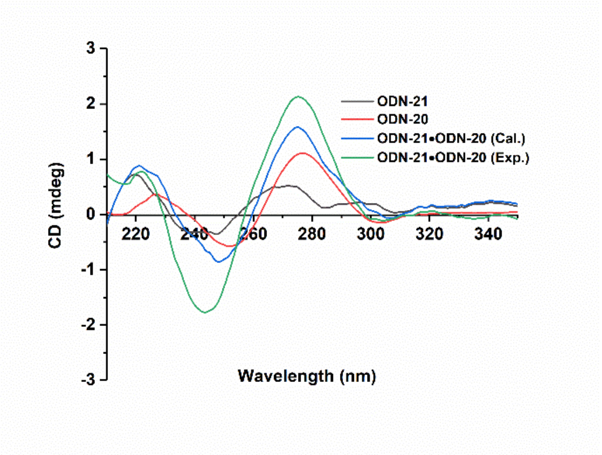

β-5’-d(TCA TAA C**13**G GAT) (ODN-**19**)
β-3’-d(AGT ATT G**14**C CTA) (ODN-**20**)

β-5’-d(TCA TAA C**18**G GAT) (ODN-**21**)
β-3’-d(AGT ATT G**14**C CTA) (ODN-**20**)

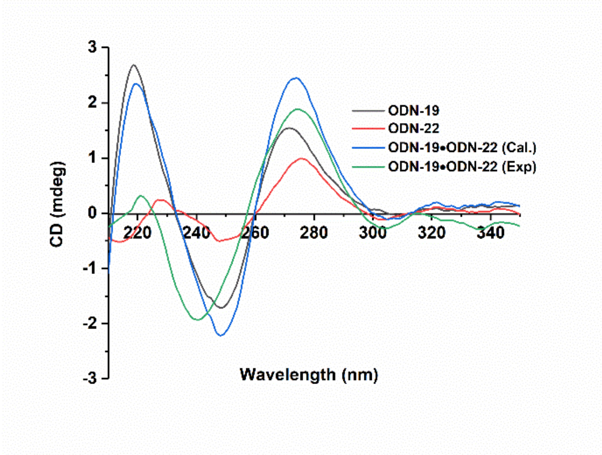

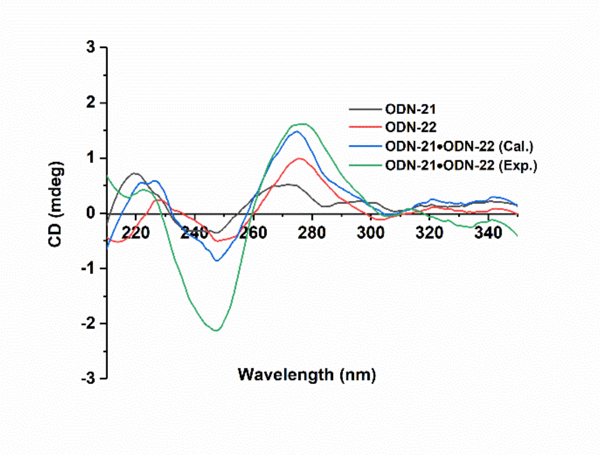

β-5’-d(TCA TAA C**13**G GAT) (ODN-**19**)
β-3’-d(AGT ATT G**20**C CTA) (ODN-**22**)

β-5’-d(TCA TAA C**18**G GAT) (ODN-**21**)
β-3’-d(AGT ATT G**20**C CTA) (ODN-**22**)

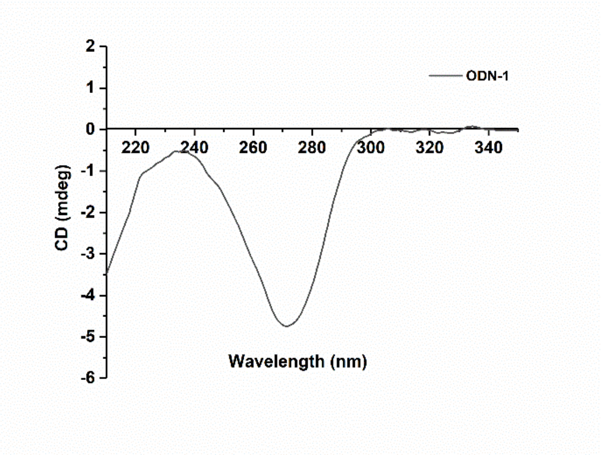

α-5‘-d(TCA TAA CTG GAT) (ODN-**1**)

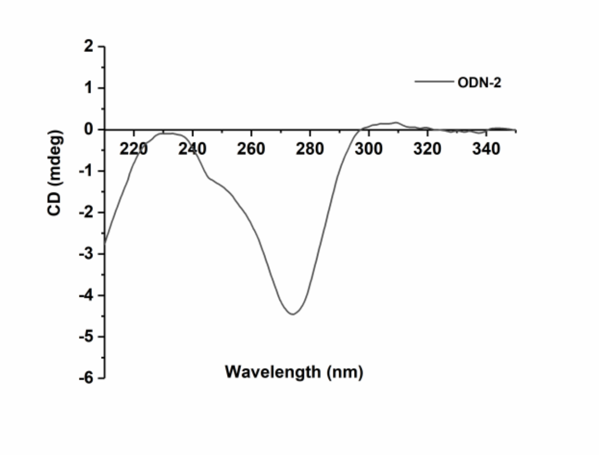

α-5‘-d(TCA TAA C2G GAT) (ODN-**2**)

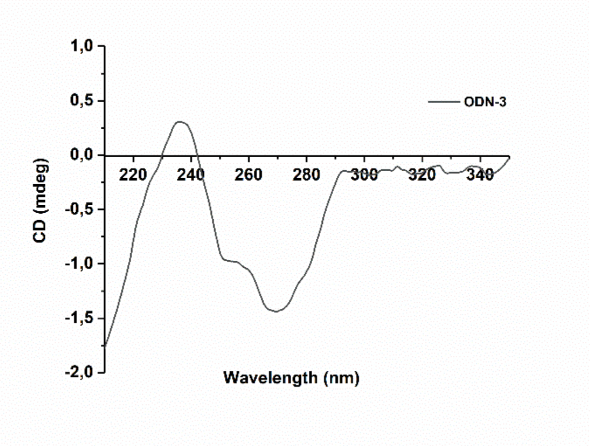

α-5‘-d(TCA TAA C17G GAT) (ODN-**3**)

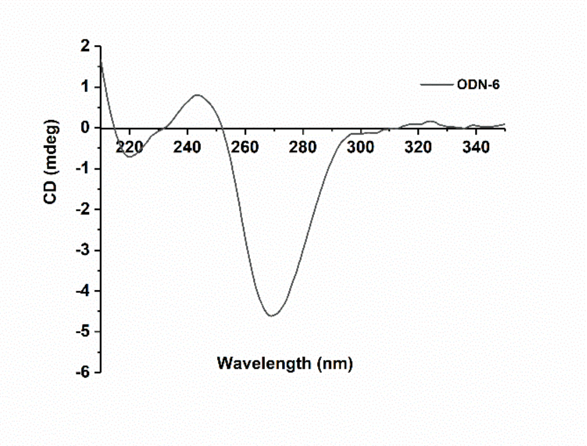

α-5’-d(AGT ATT GAC CTA) (ODN-**6**)

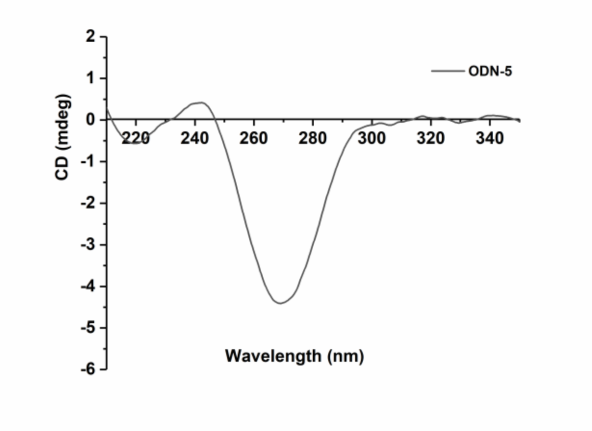

α-3’-d(AGT ATT GAC CTA) (ODN-**5**)

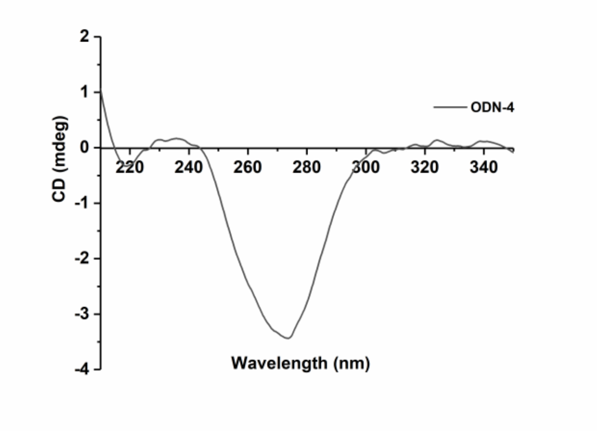

α-5’-d(TAG GTC AAT ACT) (ODN-**4**)

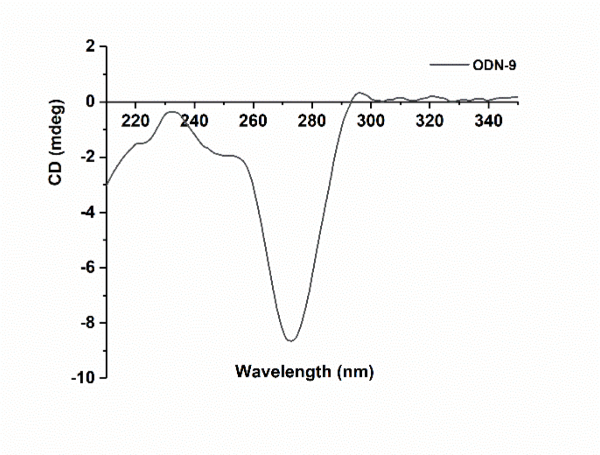

α-5’-d(CGC GAA TTC GCG) (ODN-**9**)

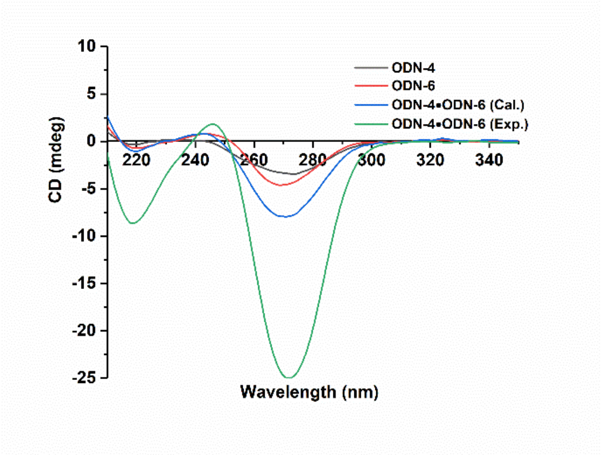

α-5’-d(TAG GTC AAT ACT) (ODN-**4**)
α-3’-d(ATC CAG TTA TGA) (ODN-**6**)

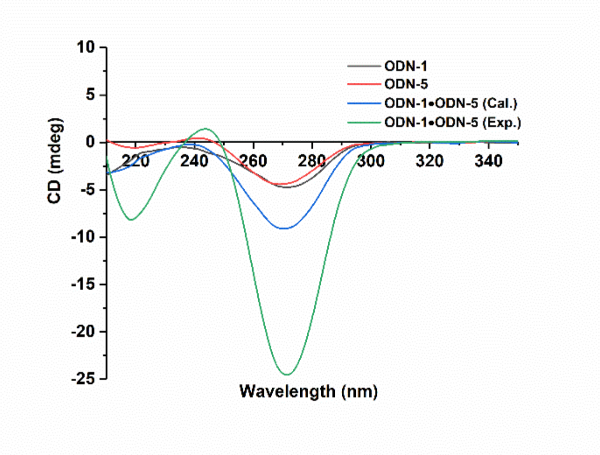

α-5’-d(TCA TAA C**T**G GAT) (ODN-**1**)
α-3’-d(AGT ATT G**A**C CTA) (ODN-**5**)

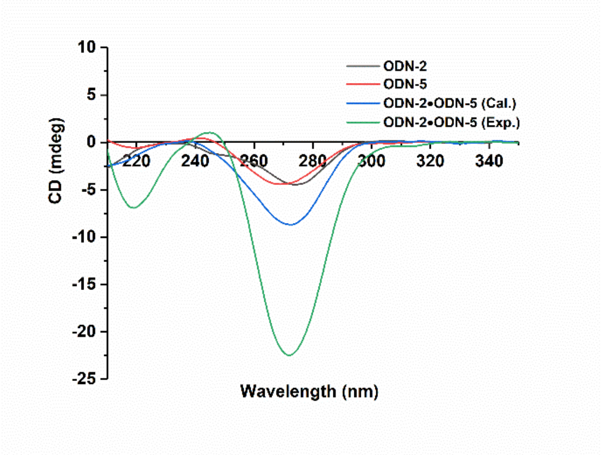

α-5‘-d(TCA TAA C**2**G GAT) (ODN-**2**)
α-3’-d(AGT ATT G**A**C CTA) (ODN-**5**)

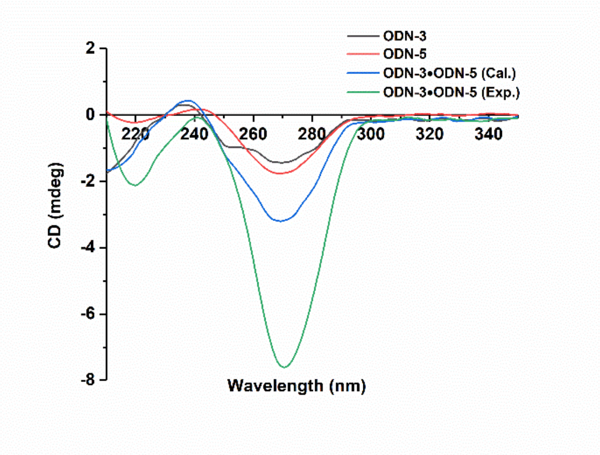

α-5‘-d(TCA TAA C**17**G GAT) (ODN-**3**)
α-3’-d(AGT ATT G**A**C CTA) (ODN-**5**)

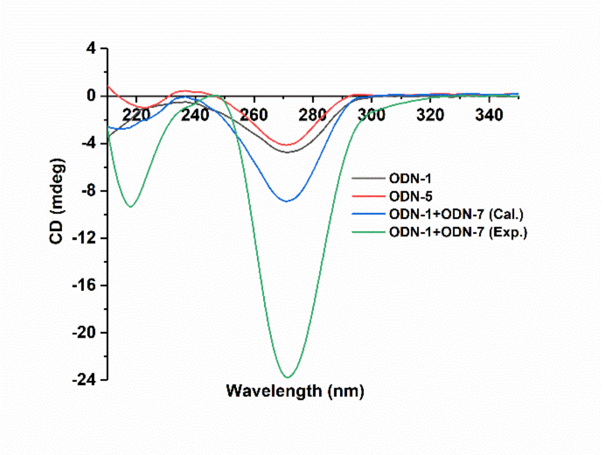

α-5’-d(TCA TAA C**T**G GAT) (ODN-**1**)
α-3’-d(AGT ATT G**9**C CTA) (ODN-**7**)

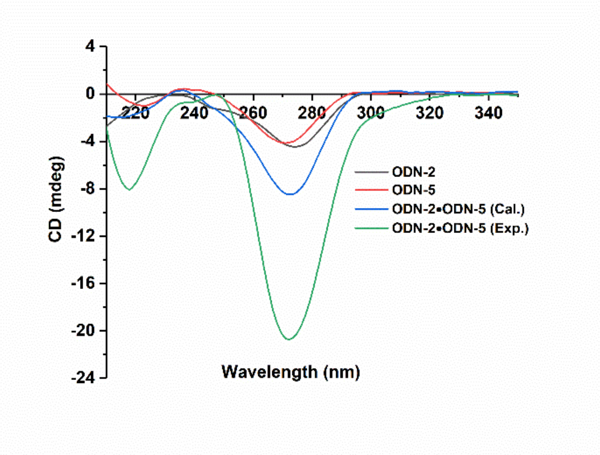

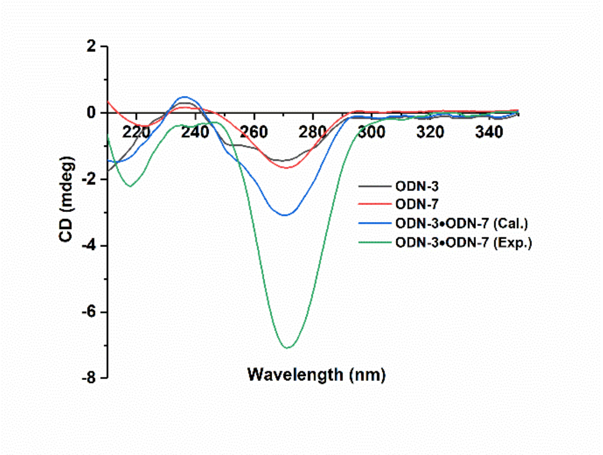

α-5‘-d(TCA TAA C2G GAT) (ODN-**2**)
α-3’-d(AGT ATT G9C CTA) (ODN-**7**)

α-5‘-d(TCA TAA C17G GAT) (ODN-**3**)
α-3’-d(AGT ATT G9C CTA) (ODN-**7**)

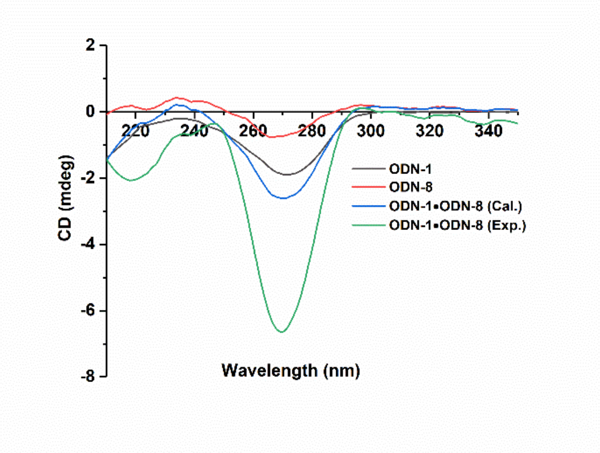

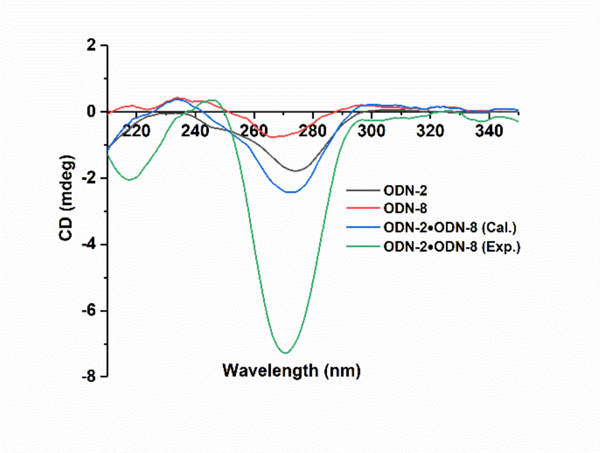

α-5’-d(TCA TAA CTG GAT) (ODN-**1**)
α-3’-d(AGT ATT G19C CTA) (ODN-**8**)

α-5‘-d(TCA TAA C2G GAT) (ODN-**2**)
α-3’-d(AGT ATT G19C CTA) (ODN-**8**)

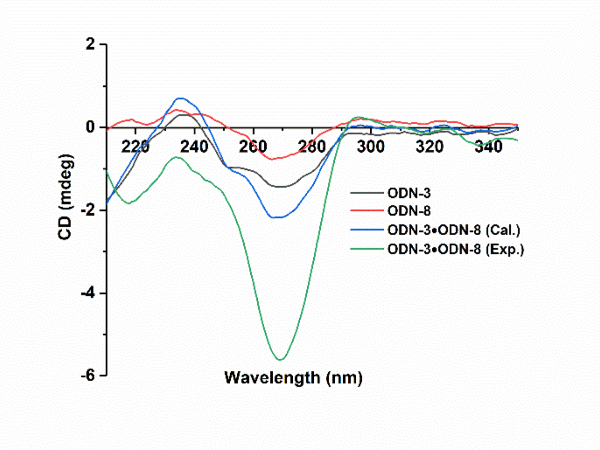

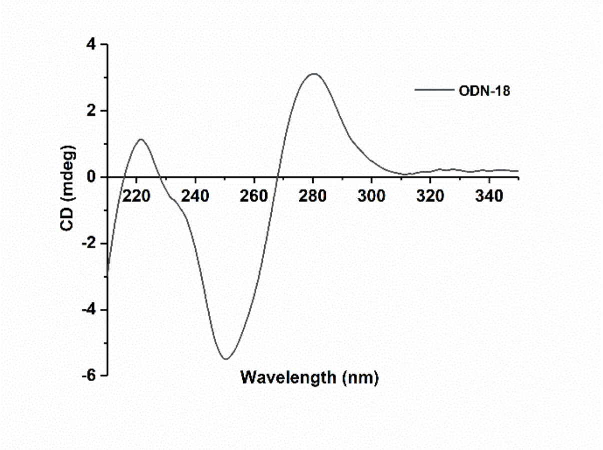

α-5’-d(TCA TAA C17G GAT) (ODN-**3**)
α-3’-d(AGT ATT G19C CTA) (ODN-**8**)

β-5’-d(CGC GAA TTC GCG) (ODN-**18**)

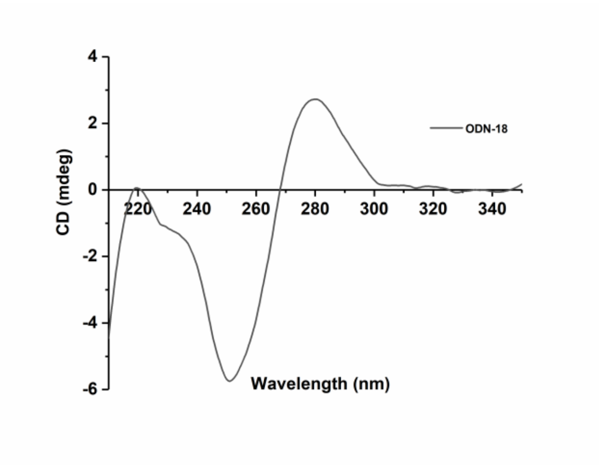

β-5’-d(CGC GAA TTC GCG) (ODN-**18**)*

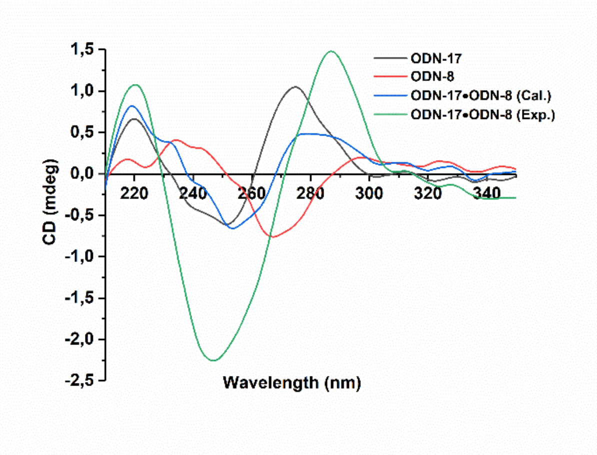

β-5’-d(TAG G**18**C AAT ACT) (ODN-**17**)
α-5’-d(ATC C**19**G TTA TGA) (ODN-**8**)

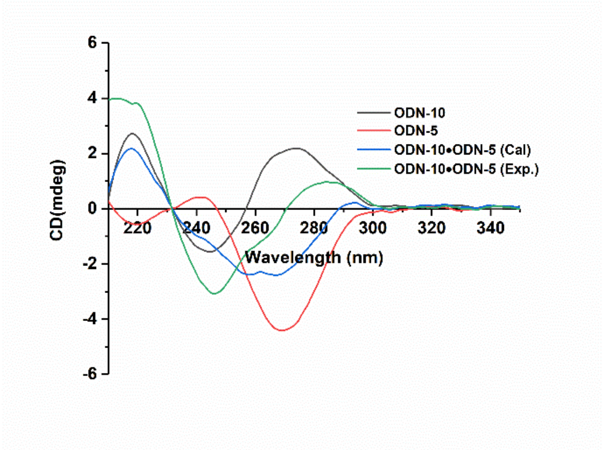

β-5’-d(TAG G**T**C AAT ACT) (ODN-**10**)
α-5’-d(ATC C**A**G TTA TGA) (ODN-**5**)

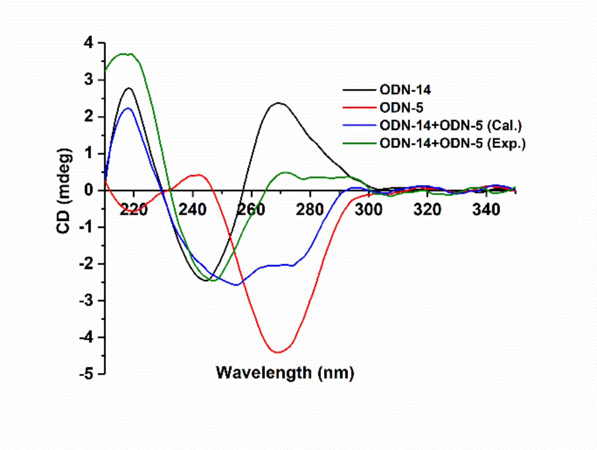

β-5’-d(TAG G**13**C AAT ACT) (ODN-**14**)
α-5’-d(ATC C**A**G TTA TGA) (ODN-**5**)

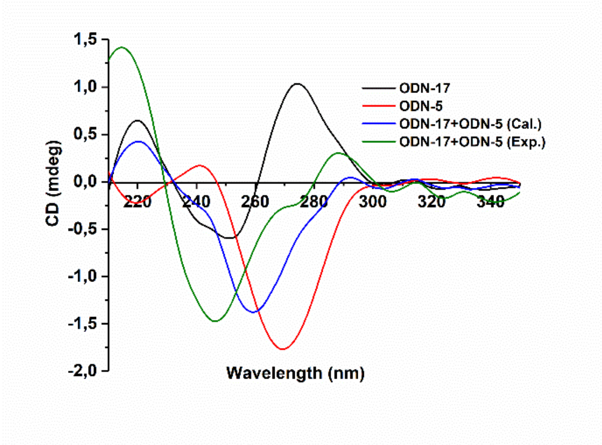

β-5’-d(TAG G**18**C AAT ACT) (ODN-**17**)
α-5’-d(ATC C**A**G TTA TGA) (ODN-**5**)

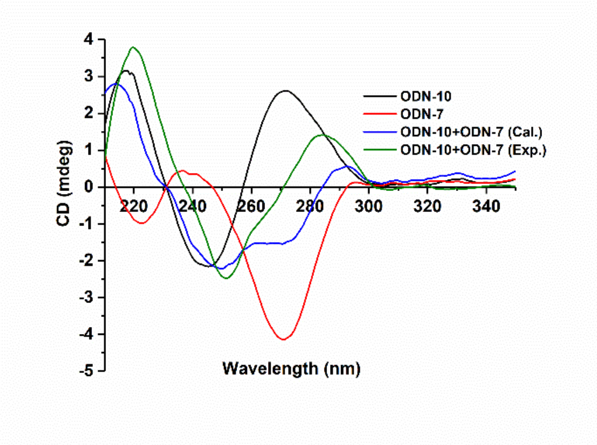

β-5’-d(TAG G**T**C AAT ACT) (ODN-**10**)
α-5’-d(ATC C**9**G TTA TGA) (ODN-**7**)

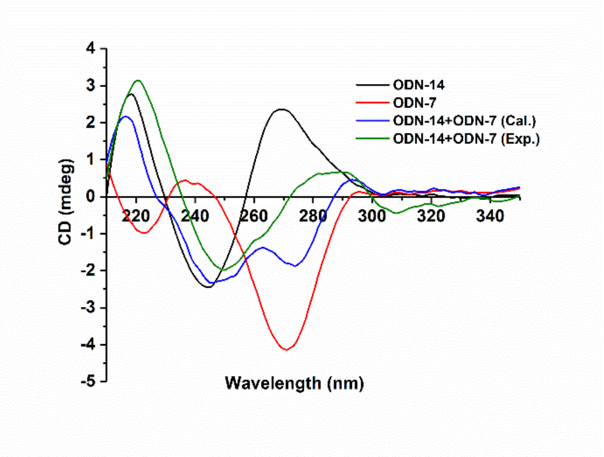

β-5’-d(TAG G**13**C AAT ACT) (ODN-**14**)
α-5’-d(ATC C**9**G TTA TGA) (ODN-**7**)

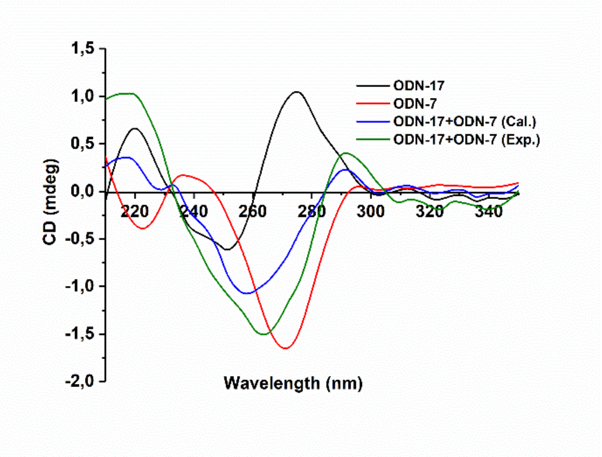

β-5’-d(TAG G**18**C AAT ACT) (ODN-**17**)
α-5’-d(ATC C**9**G TTA TGA) (ODN-**7**)

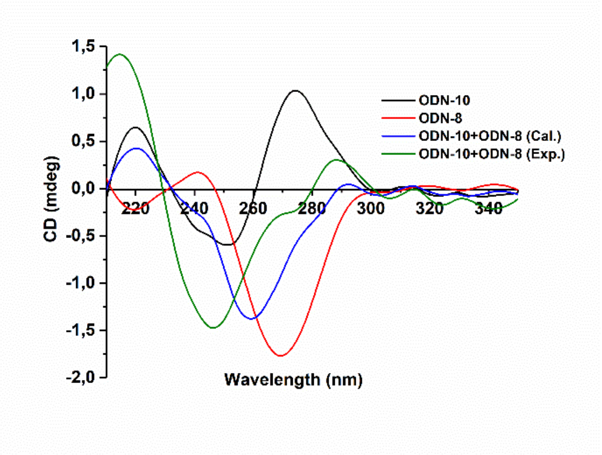

β-5’-d(TAG G**T**C AAT ACT) (ODN-**10**)
α-5’-d(ATC C**19**G TTA TGA) (ODN-**8**)

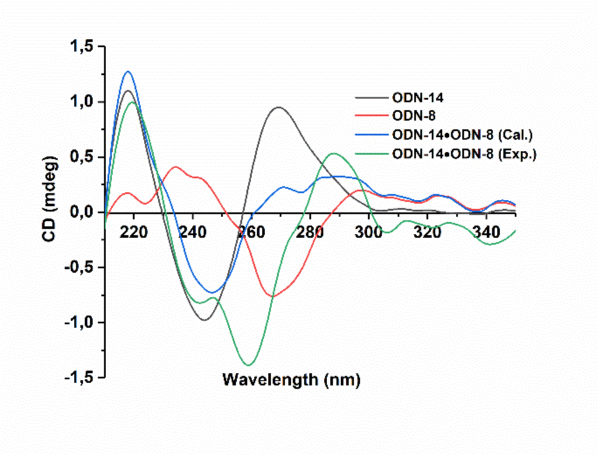

β-5’-d(TAG G**13**C AAT ACT) (ODN-**14**)
α-5’-d(ATC C**19**G TTA TGA) (ODN-**8**)

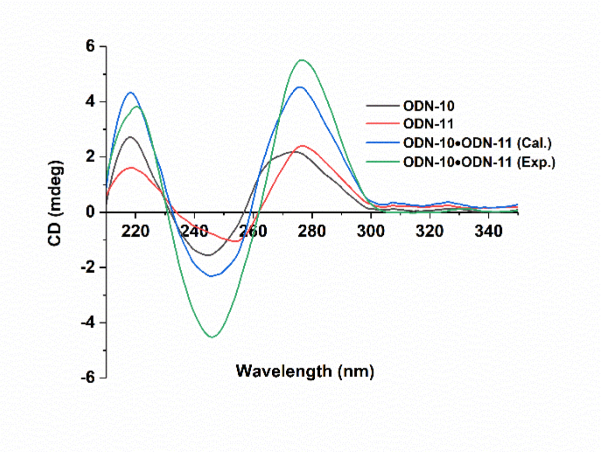

β-5‘-d(TAG G**T**C AAT ACT) (ODN-**10**)
β-3‘-d(ATC C**A**G TTA TGA) (ODN-**11**)

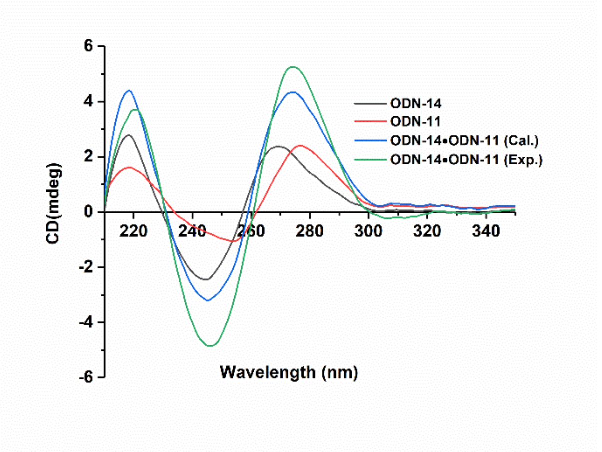

β-5‘-d(TAG G**13**C AAT ACT) (ODN-**14**)
β-3‘-d(ATC C**A**G TTA TGA) (ODN-**11**)

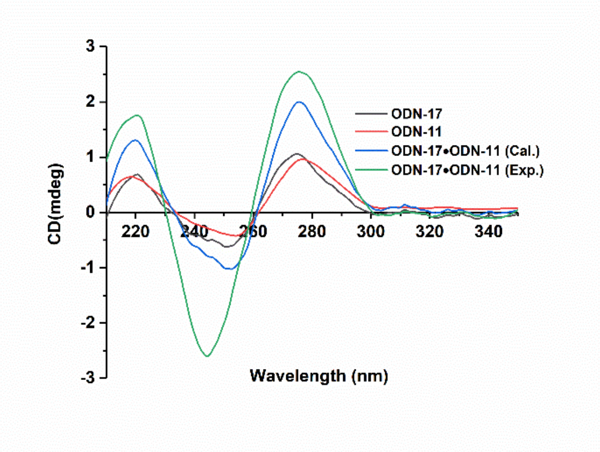

β-5‘-d(TAG G18C AAT ACT) (ODN-**17**)
β-3‘-d(ATC CAG TTA TGA) (ODN-**11**)

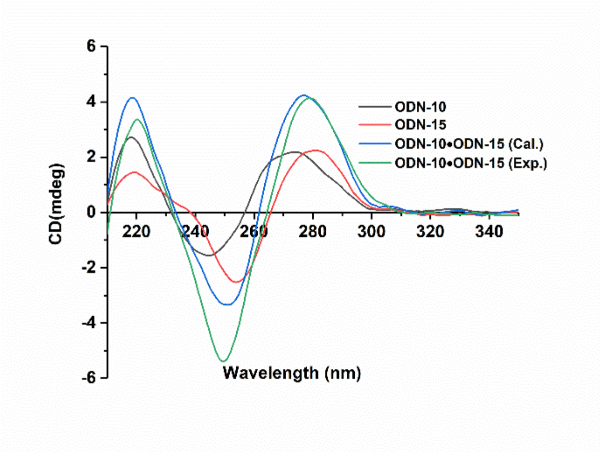

β-5‘-d(TAG GTC AAT ACT) (ODN-**10**)
β-3‘-d(ATC C14G TTA TGA) (ODN-**15**)

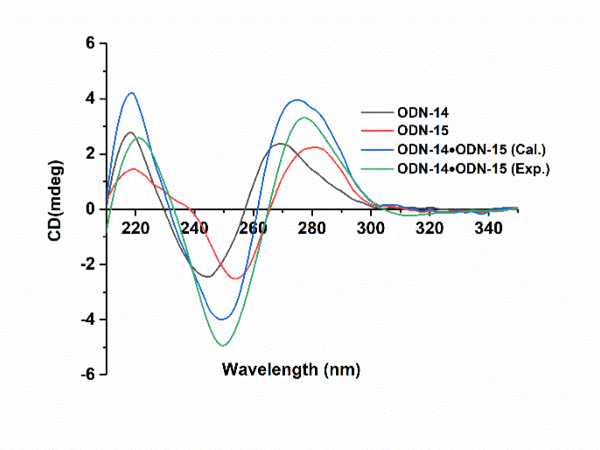

β-5‘-d(TAG G13C AAT ACT) (ODN-**14**)
β-3‘-d(ATC C14G TTA TGA) (ODN-**15**)

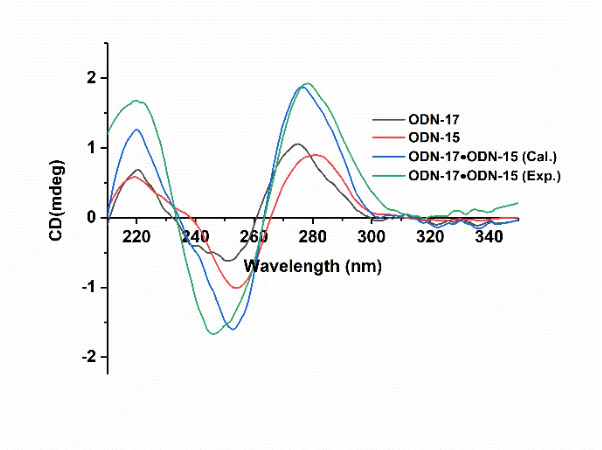

β-5‘-d(TAG G18C AAT ACT) (ODN-**17**)
β-3‘-d(ATC C14G TTA TGA) (ODN-**15**)

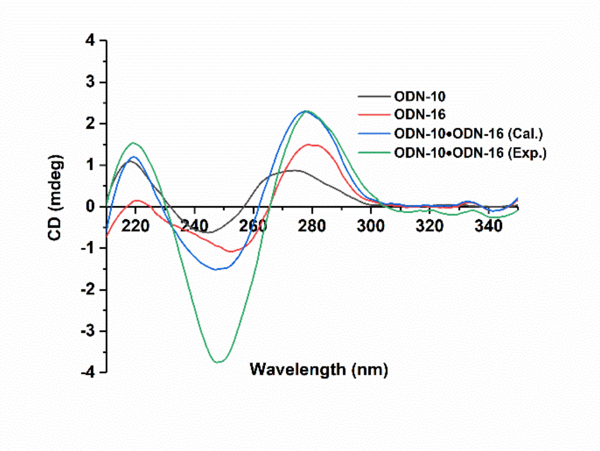

β-5‘-d(TAG GTC AAT ACT) (ODN-**10**)
β-3‘-d(ATC C20G TTA TGA) (ODN-**16**)

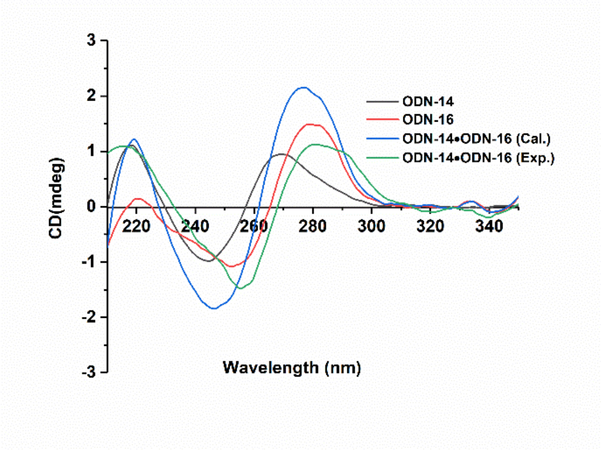

β-5‘-d(TAG G13C AAT ACT) (ODN-**14**)
β-3‘-d(ATC C20G TTA TGA) (ODN-**16**)

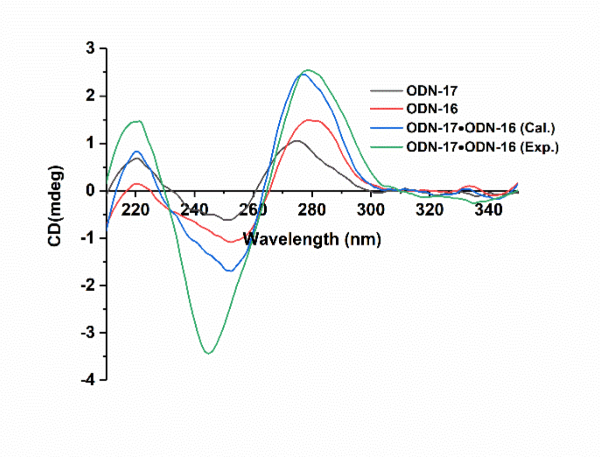

β-5'-d(TAG G**18**C AAT ACT) (ODN-**17**)
β-3'-d(ATC C**20**G TTA TGA) (ODN-**16**)

Figure S5. All measurements were performed at a concentration of 5 µM + 5 µM single strand in 100 mM NaCl, 10 mM $MgCl_2$, and 10 mM Na-cacodylate, pH 7.0. The cell path length of the cuvette for the CD measurements was 5 mm. For pyrene click adducts, 2 µM + 2 µM single strand concentration was used. *Measured in 10 mM NaCl, 10 mM NaH_2PO_4, 0.1 mM EDTA (pH 7). Black and red curves refer to the CD-spectra of the single strands. Blue curves refer to calculated spectra (sum of the CD spectra of the single strands). Green curves refer to the experimental determined spectra.

Temperature-dependent CD-spectra of duplexes

a)

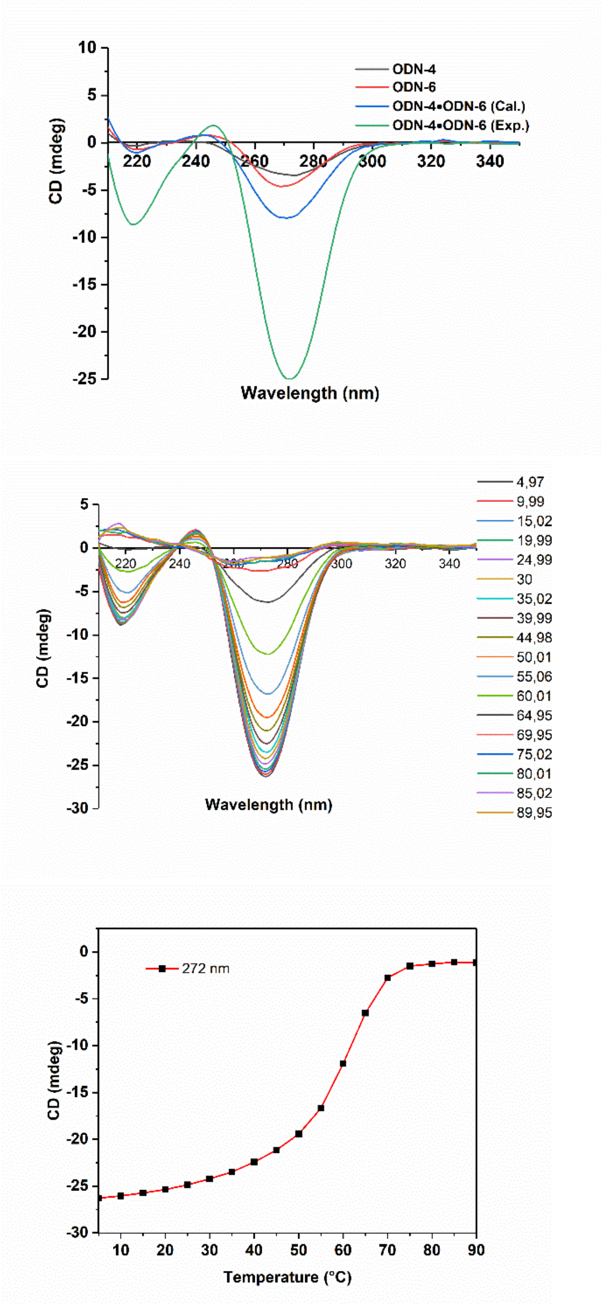

α-5’-d(TAG GTC AAT ACT) (ODN-**4**)
α-3’-d(ATC CAG TTA TGA) (ODN-**6**)

b)

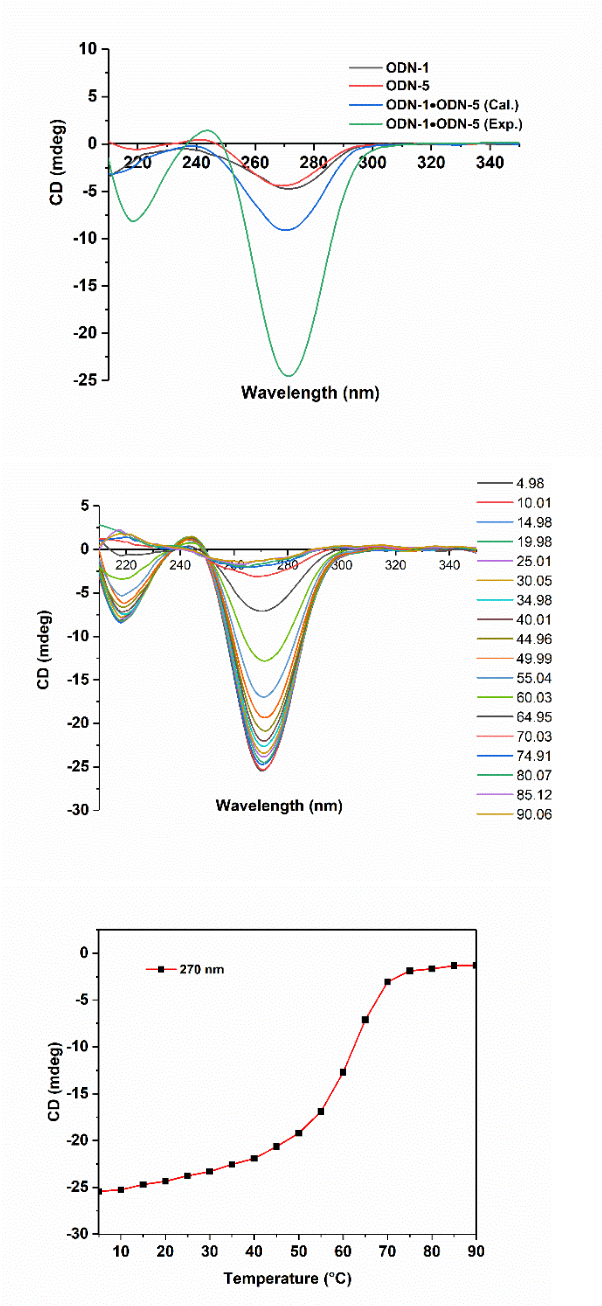

α-5’-d(TCA TAA C**T**G GAT) (ODN-**1**)
α-3’-d(AGT ATT G**A**C CTA) (ODN-**5**)

c)

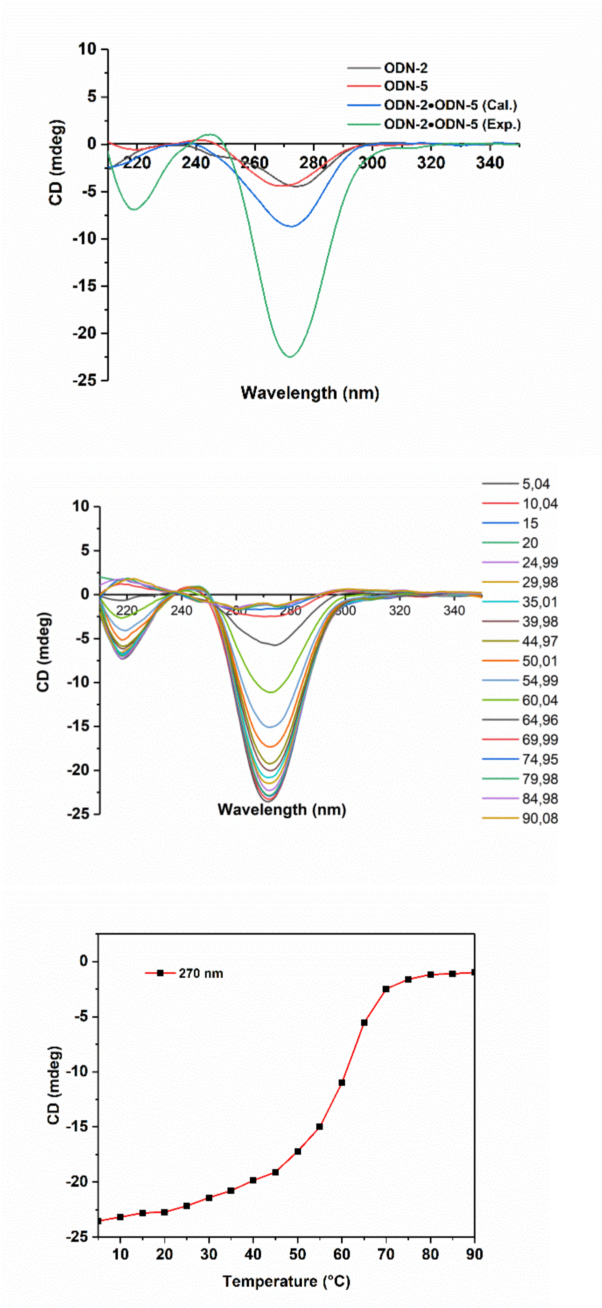

α-5'-d(TCA TAA C**2**G GAT) (ODN-**2**)

α-3'-d(AGT ATT G**A**C CTA) (ODN-**5**)

Figure S6. Temperature-dependent CD-spectra of duplexes. a) ODN-**4**•ODN-**6**; b) ODN-**1**•ODN-**5**; c) ODN-**2**•ODN-**5**. Upper graphs: CD spectra of single strands and duplexes (calculated and experimental). Middle graphs: Temperature-dependent CD-spectra. Lower graphs: CD melting curves of duplexes obtained from temperature-dependent CD spectra. All measurements were performed in 100 mM NaCl, 10 mM $MgCl_2$, and 10 mM Na-cacodylate, pH 7.0 with 5 µM +5 mM single strand concentration. The cell path length of the cuvette for the CD spectra was 5 mm.

Fluorescence spectra of nucleosides, oligonucleotides and duplexes containing pyrene click conjugates

A)

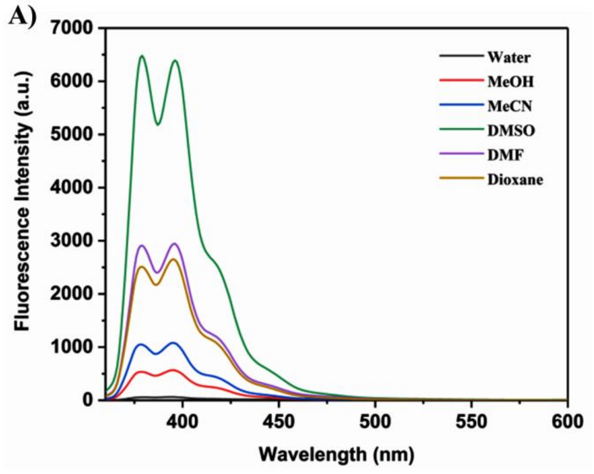

α-dU pyrene click conjugate **17**

B)

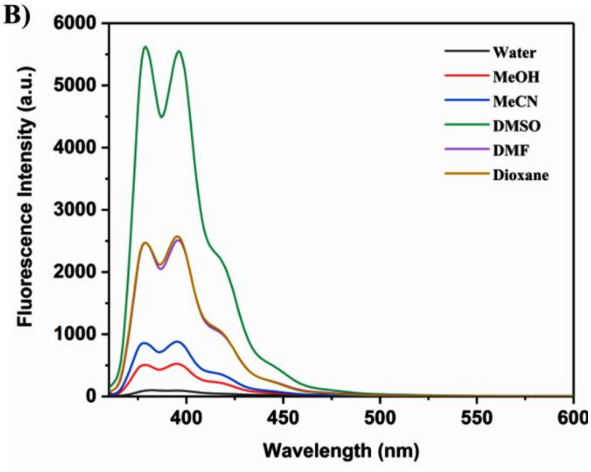

β-dU pyrene click conjugate **18**

C)

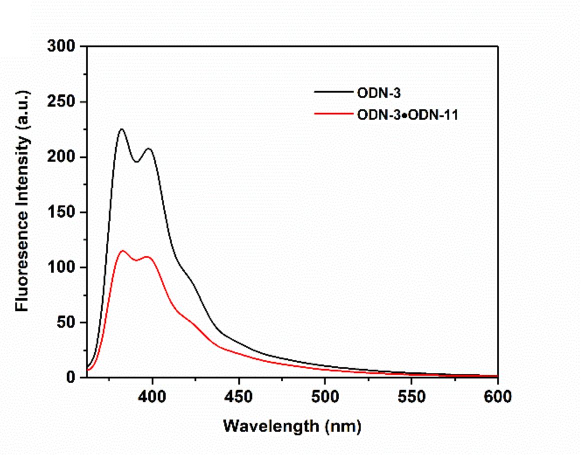

α-5‘-d(TCA TAA C17G GAT) (ODN-**3**)
β-5‘-d(AGT ATT GAC CTA) (ODN-**11**)

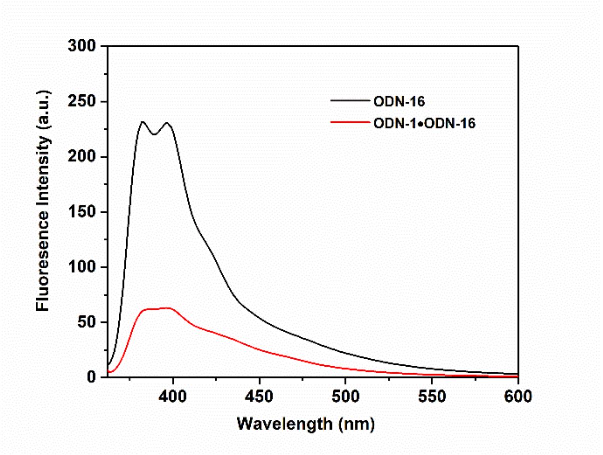

α-5‘-d(TCA TAA CTG GAT) (ODN-**1**)
β-5‘-d(AGT ATT G20C CTA) (ODN-**16**)

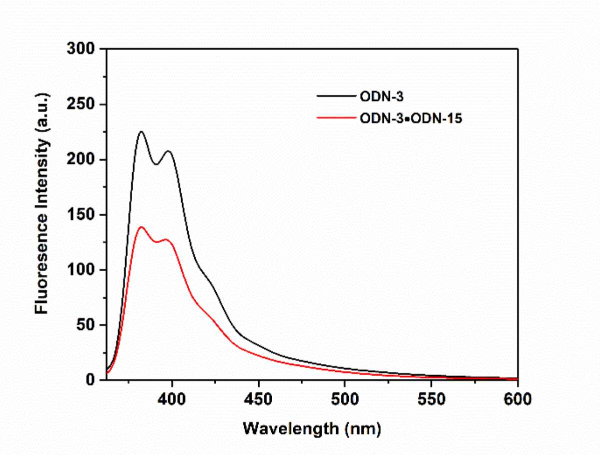

α-5‘-d(TCA TAA C17G GAT) (ODN-**3**)
β-5‘-d(AGT ATT G14C CTA) (ODN-**15**)

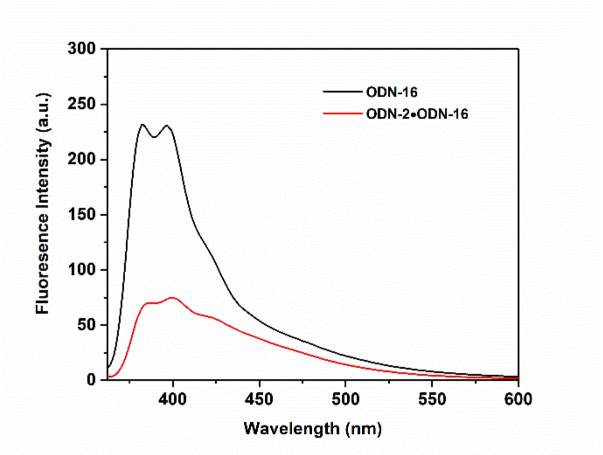

α-5‘-d(TCA TAA C2G GAT) (ODN-**2**)
β-5‘-d(AGT ATT G20C CTA) (ODN-**16**)

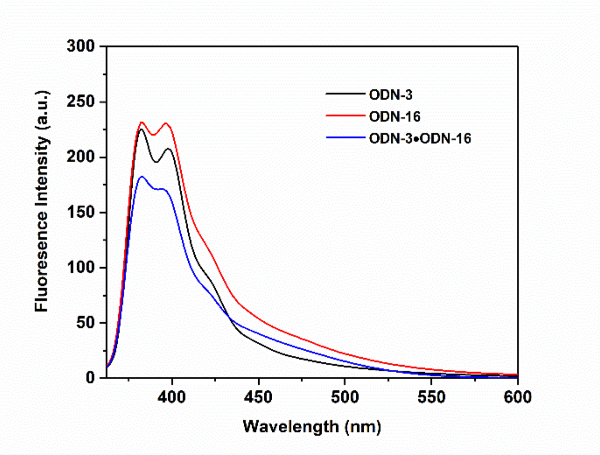

α-5‘-d(TCA TAA C**17**G GAT) (ODN-**3**)
β-5‘-d(AGT ATT G**20**C CTA) (ODN-**16**)

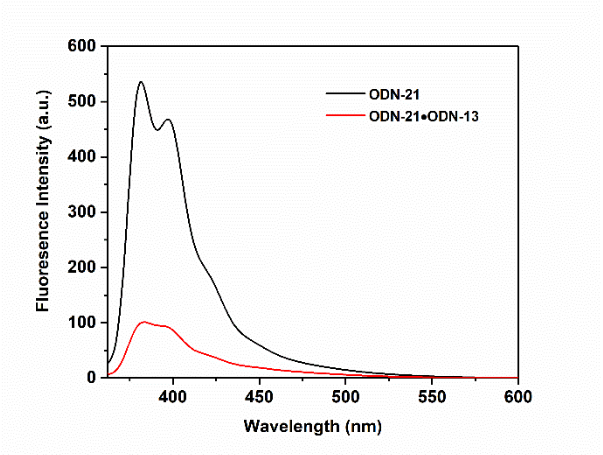

β-5’-d(TCA TAA C**18**G GAT) (ODN-**21**)
β-3’-d(AGT ATT G**A**C CTA) (ODN-**13**)

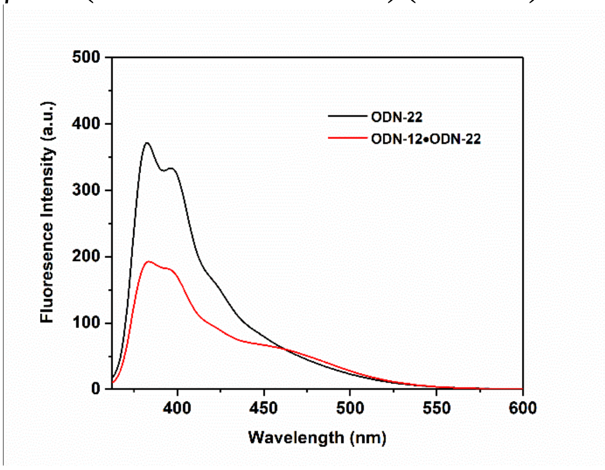

β-5’-d(TCA TAA C**T**G GAT) (ODN-**12**)
β-3’-d(AGT ATT G**20**C CTA) (ODN-**22**)

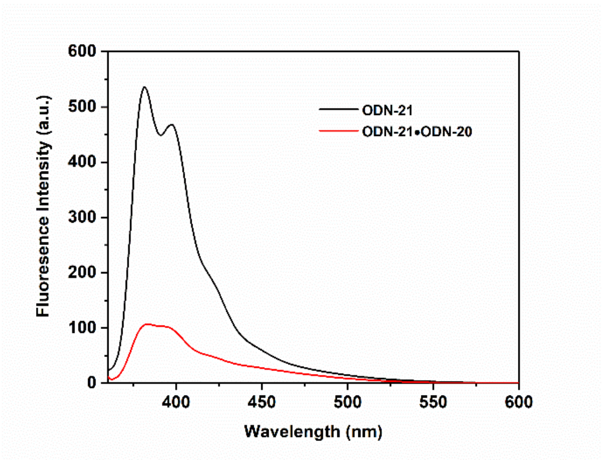

β-5’-d(TCA TAA C**18**G GAT) (ODN-**21**)
β-3’-d(AGT ATT G**14**C CTA) (ODN-**20**)

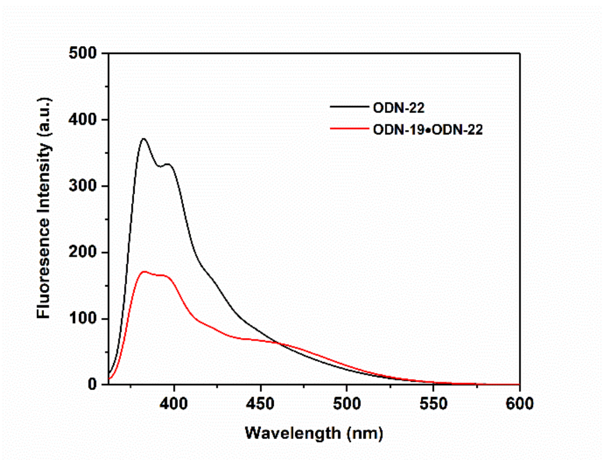

β-5’-d(TCA TAA C**13**G GAT) (ODN-**19**)
β-3’-d(AGT ATT G**20**C CTA) (ODN-**22**)

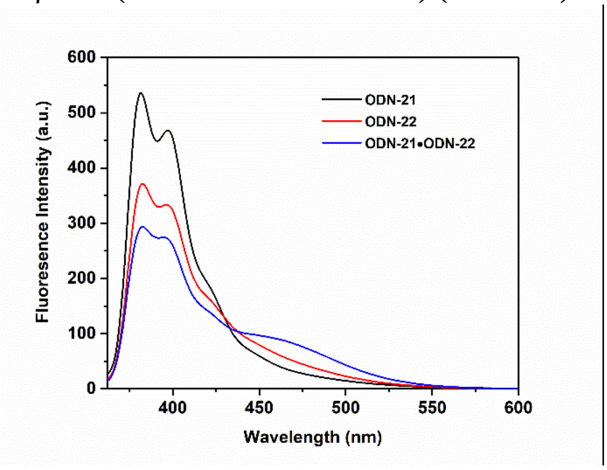

β-5’-d(TCA TAA C**18**G GAT) (ODN-**21**)
β-3’-d(AGT ATT G**20**C CTA) (ODN-**22**)

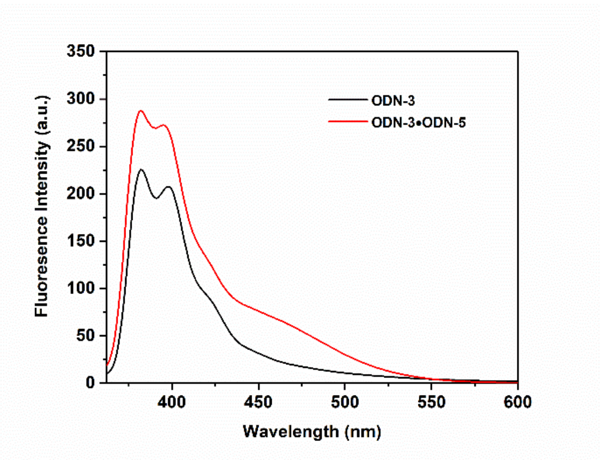

α-5‘-d(TCA TAA C17G GAT) (ODN-**3**)
α-3’-d(AGT ATT GAC CTA) (ODN-**5**)

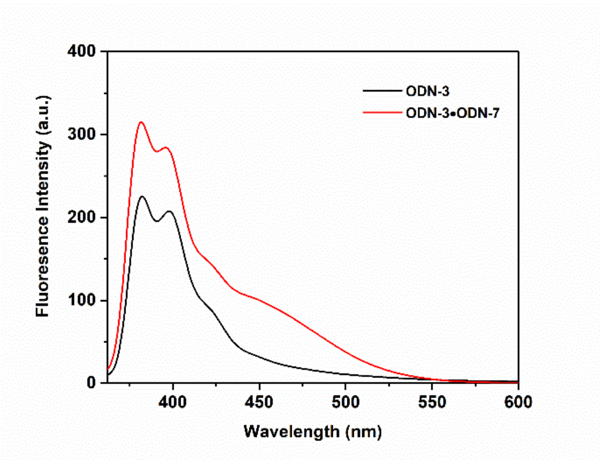

α-5‘-d(TCA TAA C17G GAT) (ODN-**3**)
α-3’-d(AGT ATT G9C CTA) (ODN-**7**)

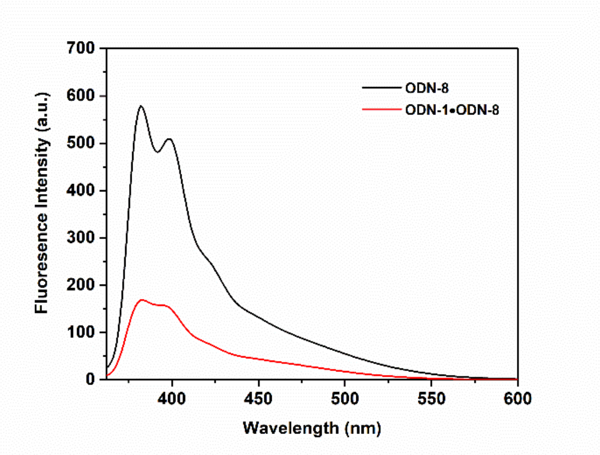

α-5’-d(TCA TAA CTG GAT) (ODN-**1**)
α-3’-d(AGT ATT G19C CTA) (ODN-**8**)

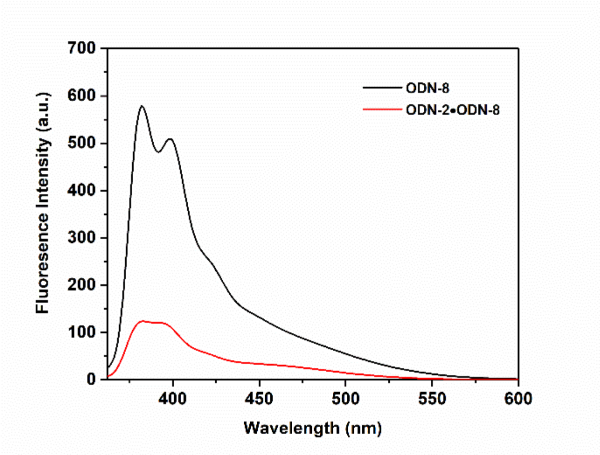

α-5‘-d(TCA TAA C2G GAT) (ODN-**2**)
α-3’-d(AGT ATT G19C CTA) (ODN-**8**)

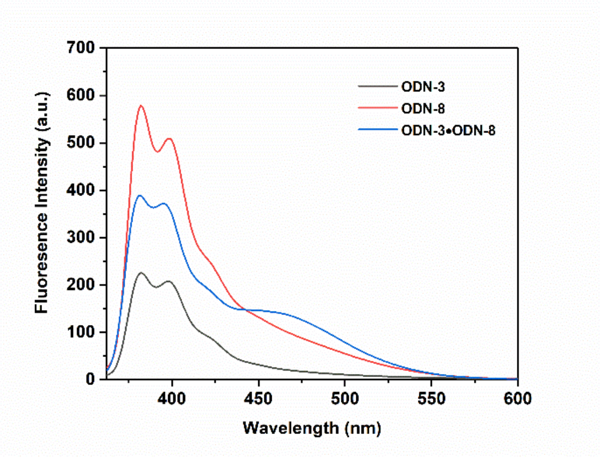

α-5’-d(TCA TAA C17G GAT) (ODN-**3**)
α-3’-d(AGT ATT G19C CTA) (ODN-**8**)

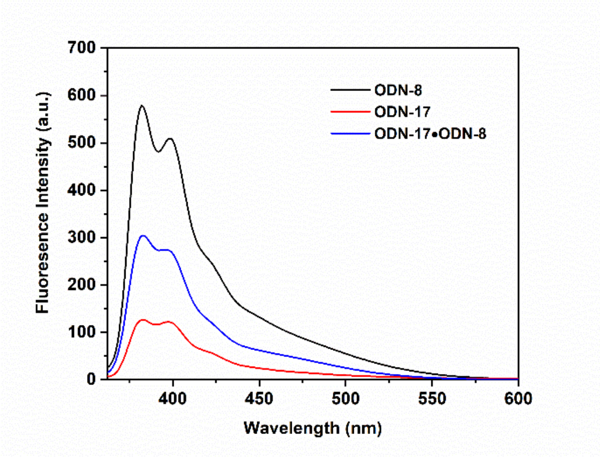

β-5’-d(TAG G18C AAT ACT) (ODN-**17**)
α-5’-d(ATC C19G TTA TGA) (ODN-**8**)

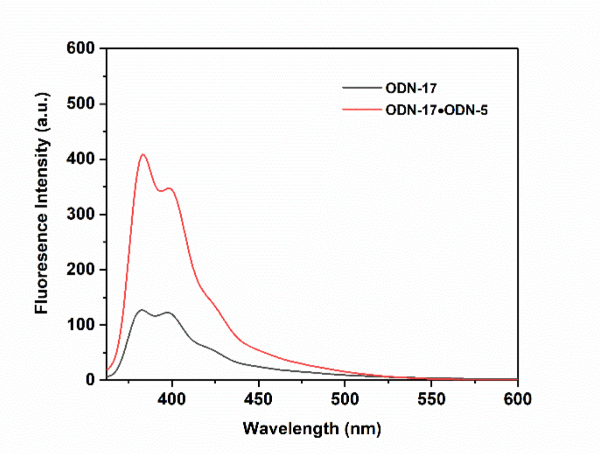

β-5’-d(TAG G**18**C AAT ACT) (ODN-**17**)
α-5’-d(ATC C**A**G TTA TGA) (ODN-**5**)

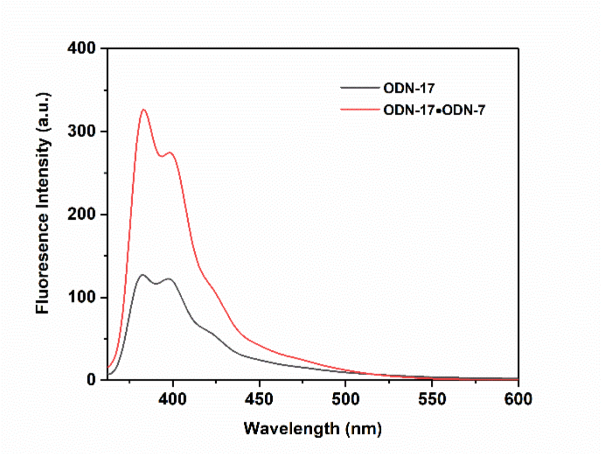

β-5’-d(TAG G**18**C AAT ACT) (ODN-**17**)
α-5’-d(ATC C**9**G TTA TGA) (ODN-**7**)

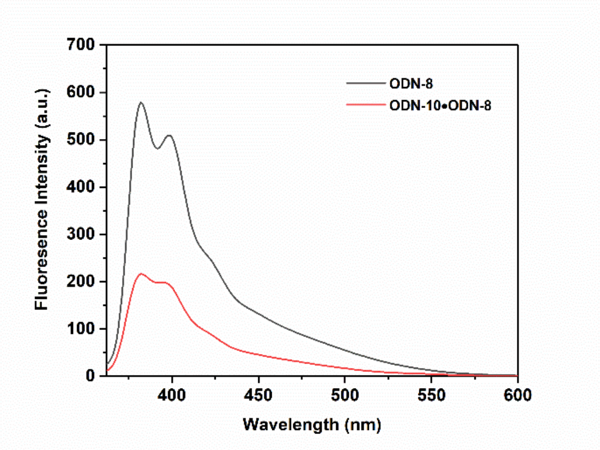

β-5’-d(TAG G**T**C AAT ACT) (ODN-**10**)
α-5’-d(ATC C**19**G TTA TGA) (ODN-**8**)

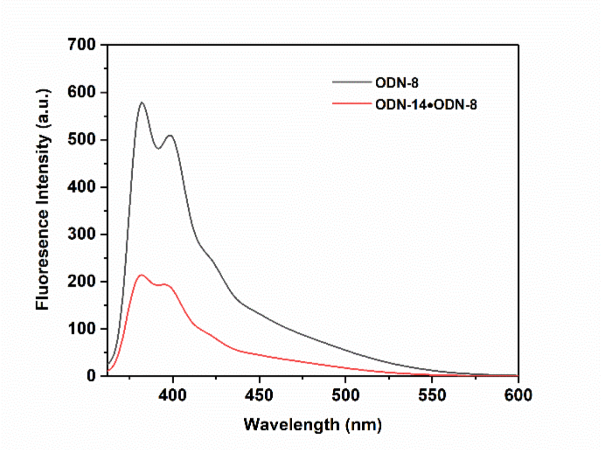

β-5’-d(TAG G**13**C AAT ACT) (ODN-**14**)
α-5’-d(ATC C**19**G TTA TGA) (ODN-**8**)

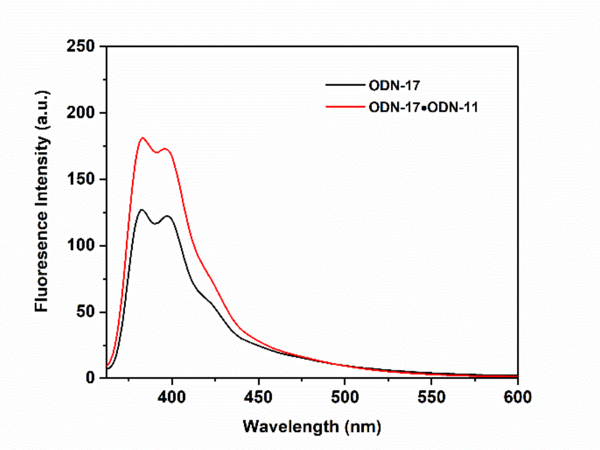

β-5‘-d(TAG G**18**C AAT ACT) (ODN-**17**)
β-3‘-d(ATC C**A**G TTA TGA) (ODN-**11**)

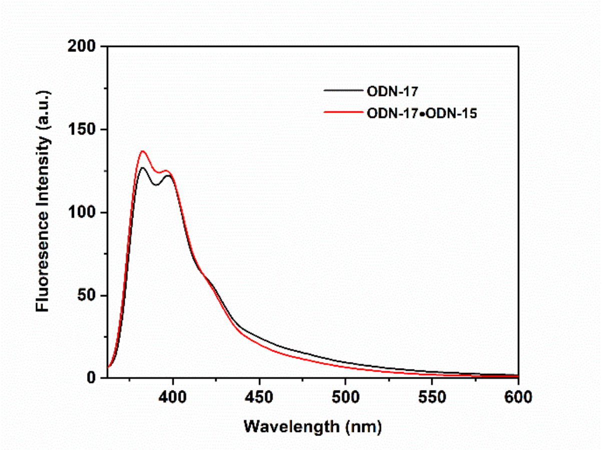

β-5‘-d(TAG G**18**C AAT ACT) (ODN-**17**)
β-3‘-d(ATC C**14**G TTA TGA) (ODN-**15**)

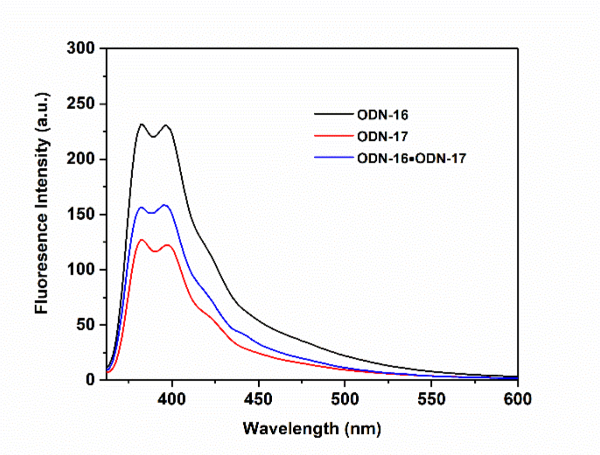

β-5'-d(TAG G18C AAT ACT) (ODN-**17**)
β-3'-d(ATC C20G TTA TGA) (ODN-**16**)

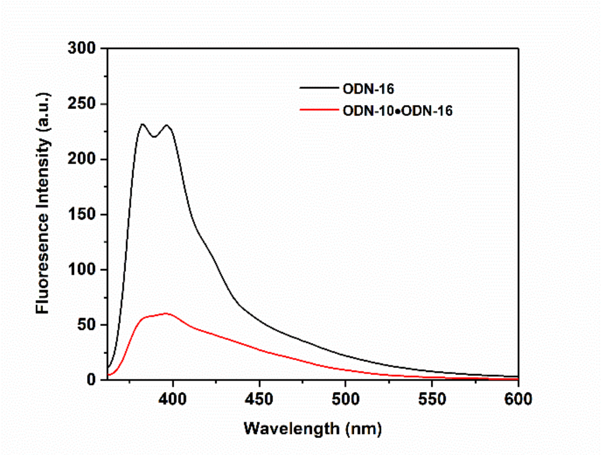

β-5'-d(TAG GTC AAT ACT) (ODN-**10**)
β-3'-d(ATC C20G TTA TGA) (ODN-**16**)

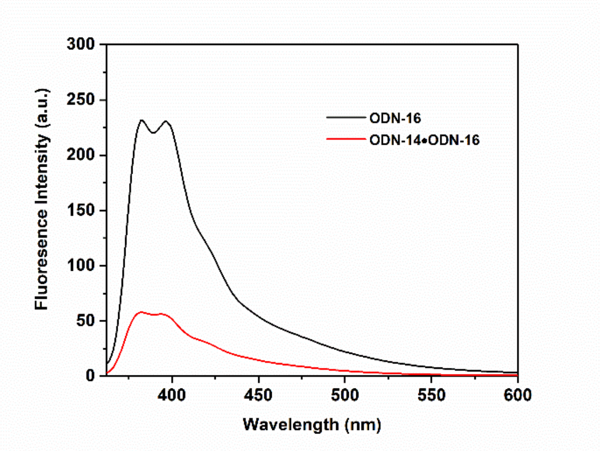

β-5'-d(TAG G13C AAT ACT) (ODN-**14**)
β-3'-d(ATC C20G TTA TGA) (ODN-**16**)

Figure S7. A) and B) Fluorescence emission spectra of pyrene click conjugate **17** and **18** measured in various solvents with a nucleoside concentration of 2 µM. C) Fluorescence spectra of oligonucleotides and duplexes determined in 100 mM NaCl, 10 mM $MgCl_2$, 10 mM Na-cacodylate, pH 7.0 with a single strand concentration of 2 µM + 2 µM. The excitation wavelength was 344 nm in all cases.

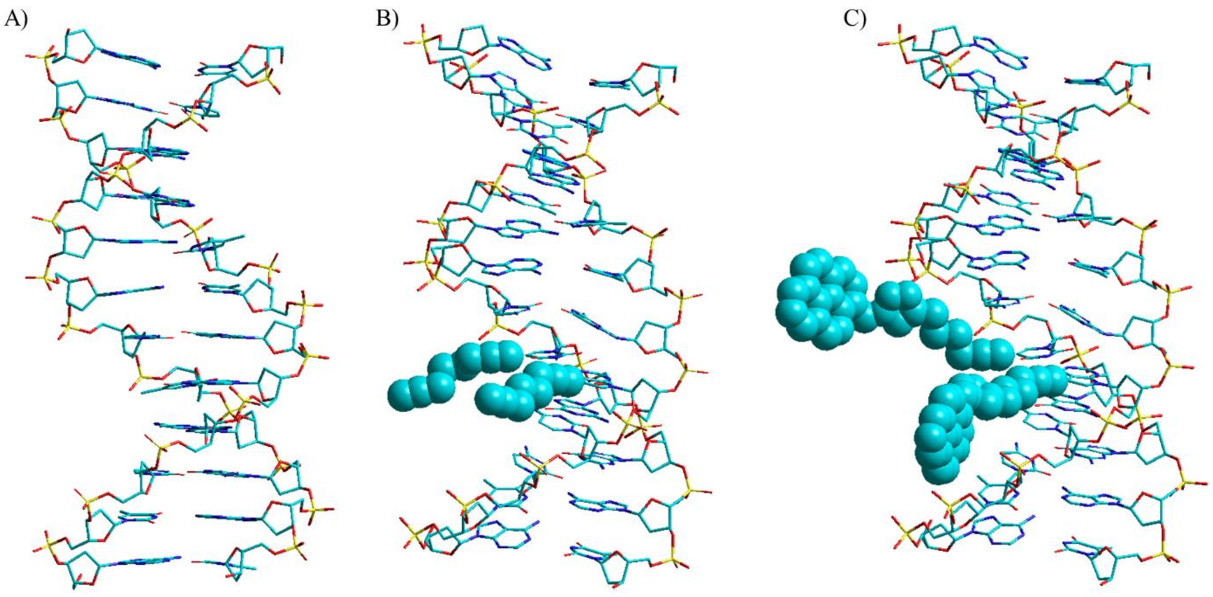

Figure S8. Calculated minimum energy structures of homochiral antiparallel β/β-D duplexes. A) ODN-**12**•ODN-**13**; B) ODN-**19•**ODN-**20**; C) ODN-**21**•ODN-**22** using AMBER force field implemented in the software package *HyperChem* 8.0. The side chains are presented as space filling balls in cyan.

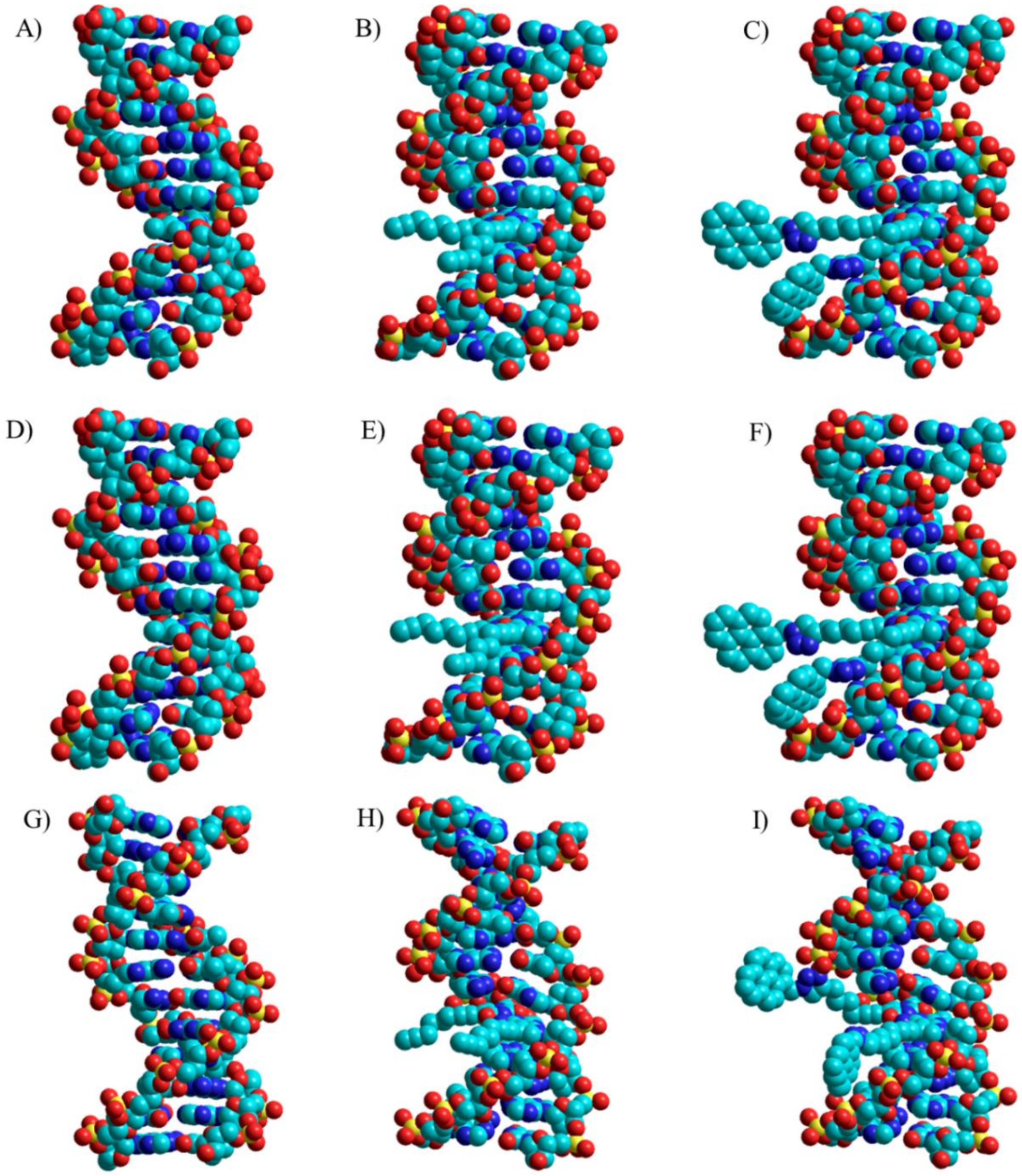

Figure S9. Calculated minimum energy structures of heterochiral parallel α/β-D duplexes. A) ODN-**1**•ODN-**11**; B) ODN-**2**•ODN-**15**; C) ODN-**3**•ODN-**16**. Calculated minimum energy structures of homochiral antiparallel α/α-D duplexes. D) ODN-**1**•ODN-**5**; E) ODN-**2**•ODN-**7**; F) ODN-**3**•ODN-**8**. Calculated minimum energy structures of homochiral antiparallel β/β-D duplexes. G) ODN-**12**•ODN-**13**; H) ODN-**19**•ODN-**20**; I) ODN-**21**•ODN-**22** with overlapping spheres using the AMBER force field as implemented in the software package *HyperChem* 8.0.

References

[1] F. Seela, M. Zulauf, H. Reuter, G. Kastner, *Acta Cryst.* **1999**, C*55*, 1560-1562.

[2] J. A. McDowell, D. H. Turner, *Biochemistry* **1996**, *35*, 14077-14089.

NMR spectra of synthesized compounds

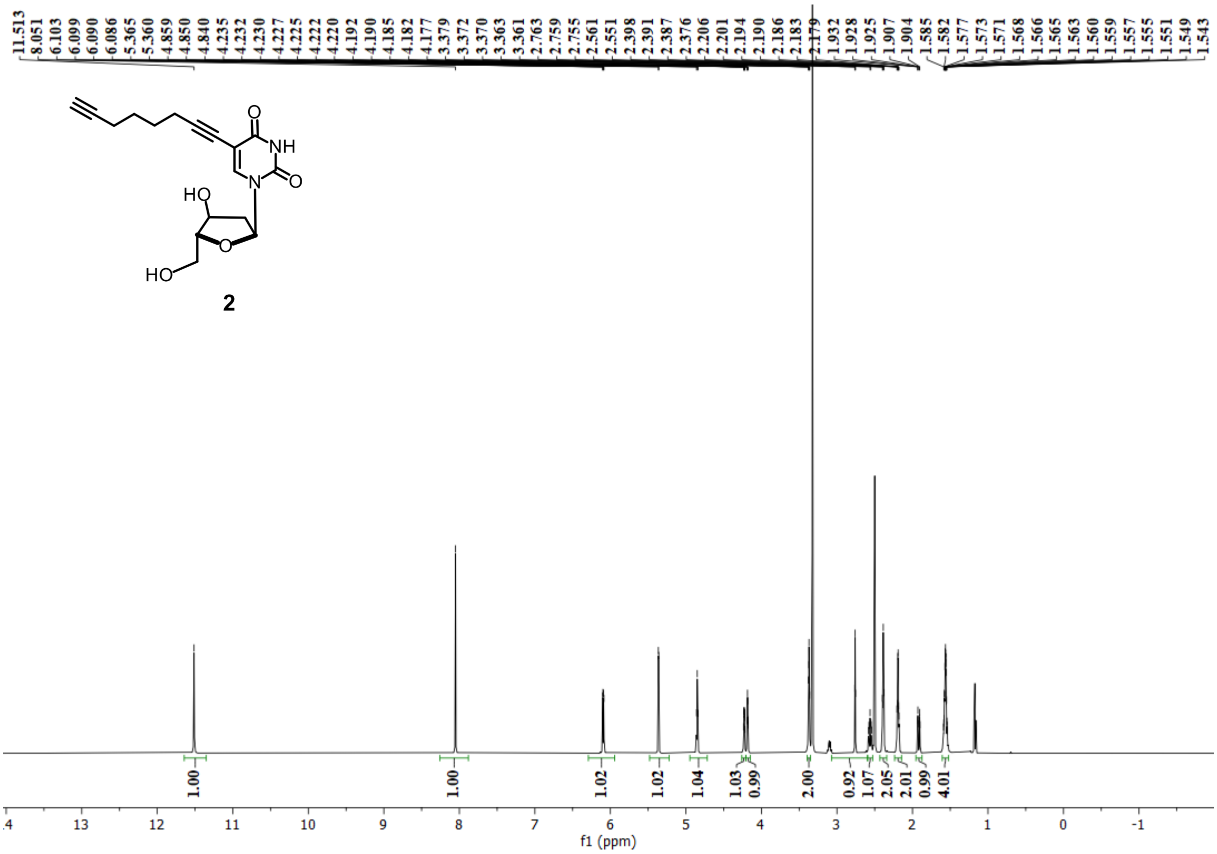

Figure S10. ^{1}H NMR (DMSO-d_6, 600 MHz) spectrum of compound **2**.

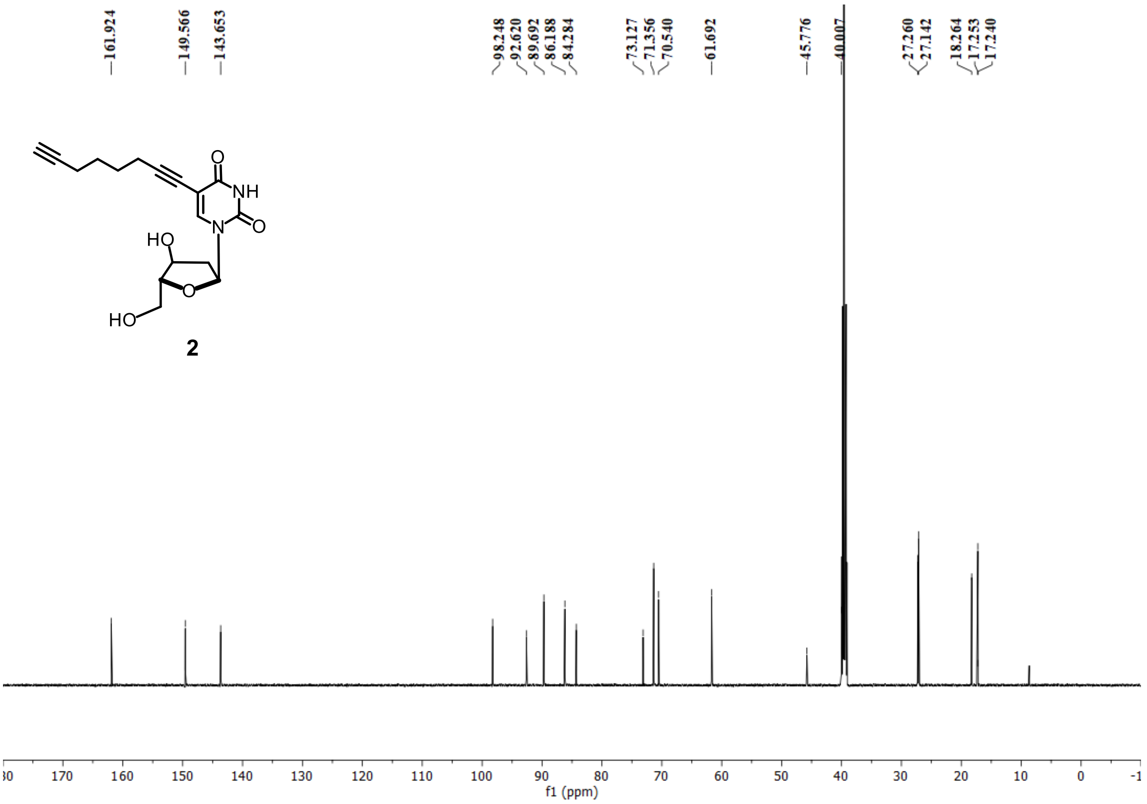

Figure S11. ^{13}C NMR (DMSO-d_6, 150 MHz) spectrum of compound **2**.

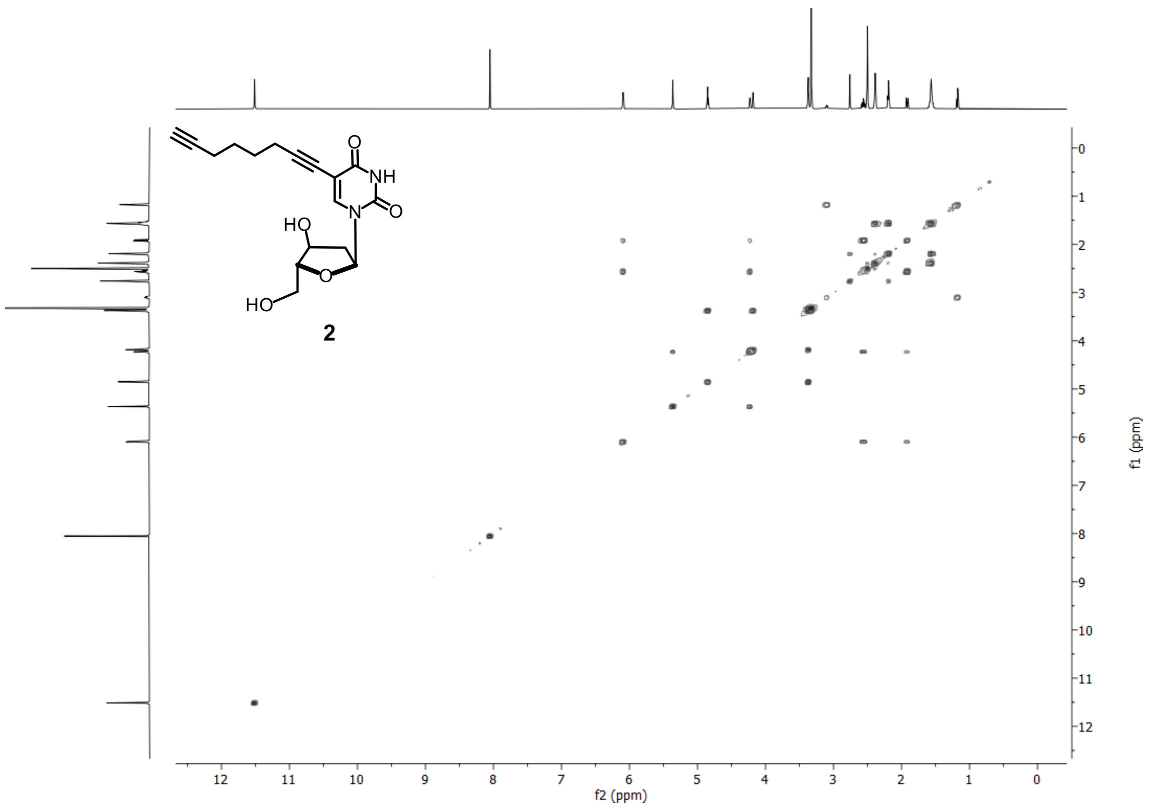

Figure S12. COSY NMR (DMSO-d_6, 600 MHz) spectrum of compound **2**.

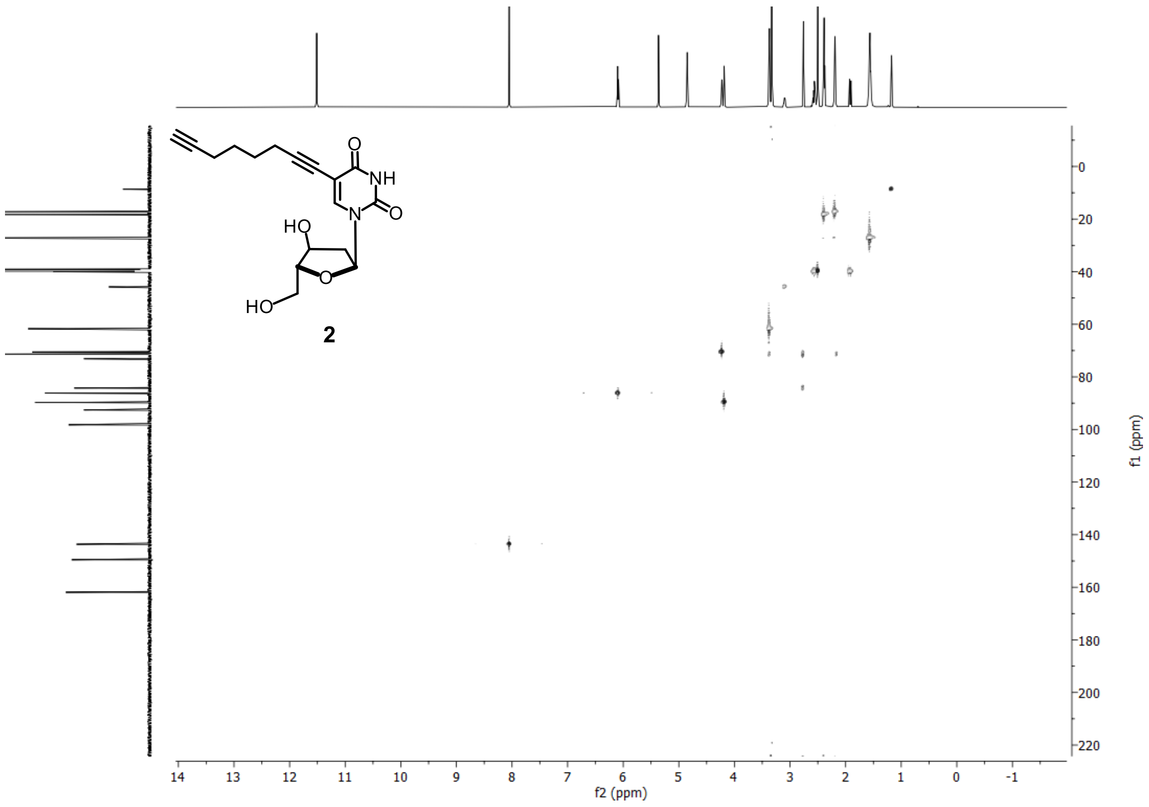

Figure S13. HSQC NMR (DMSO-d_6, 600 MHz) spectrum of compound **2**.

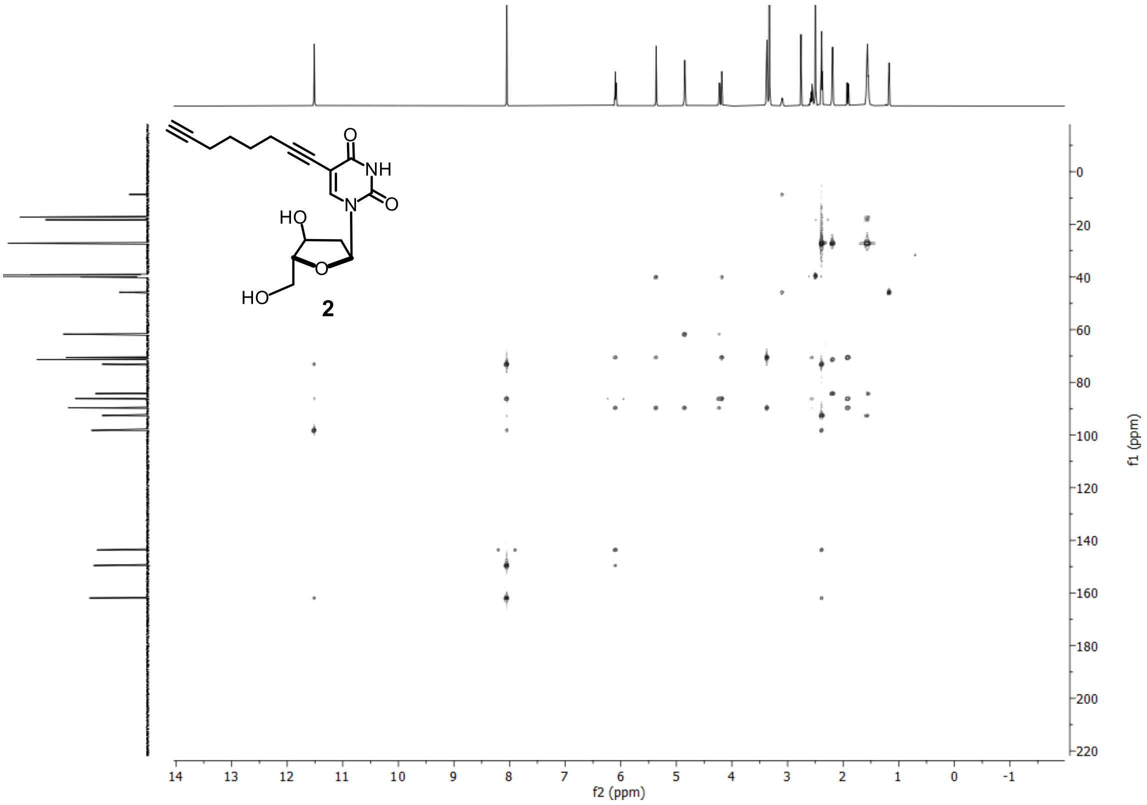

Figure S14. HMBC NMR (DMSO-d_6, 600 MHz) spectrum of compound **2**.

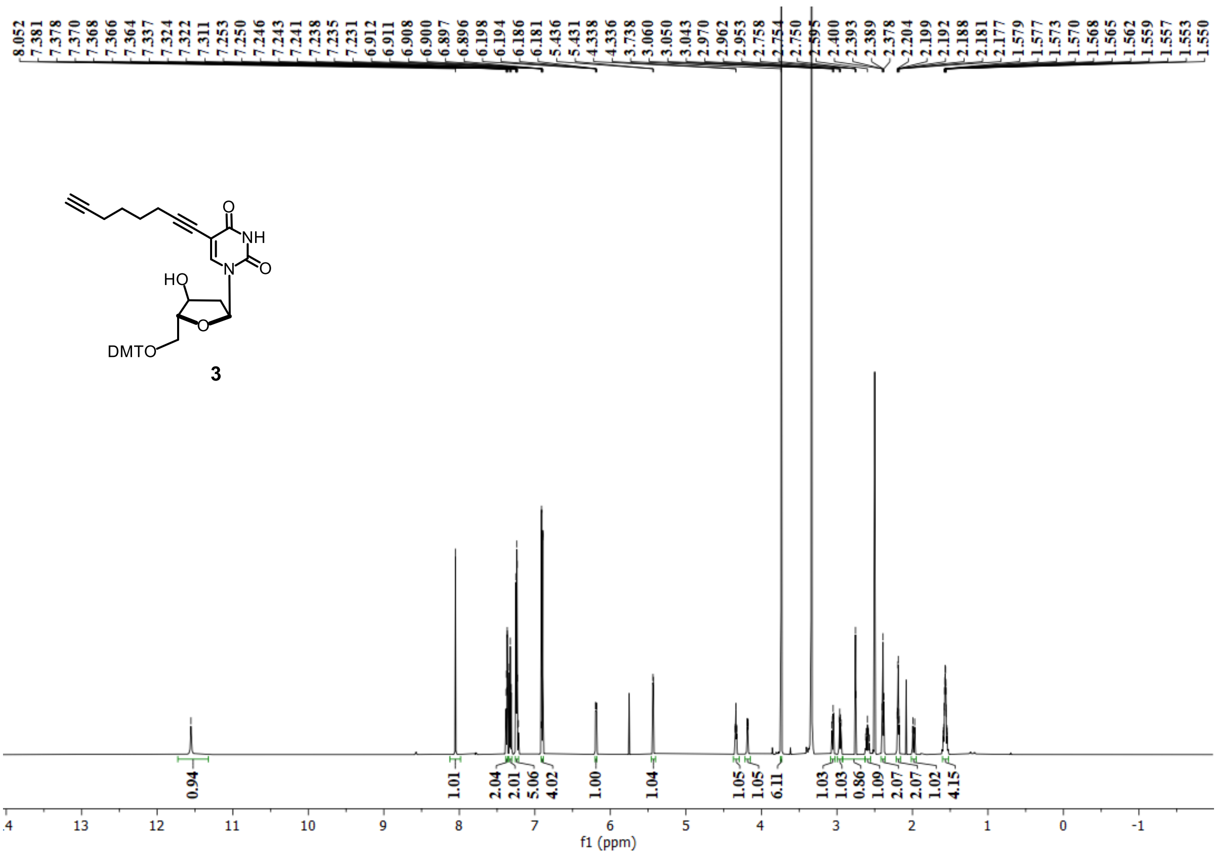

Figure S15. ^{1}H NMR (DMSO-d_6, 600 MHz) spectrum of compound **3**.

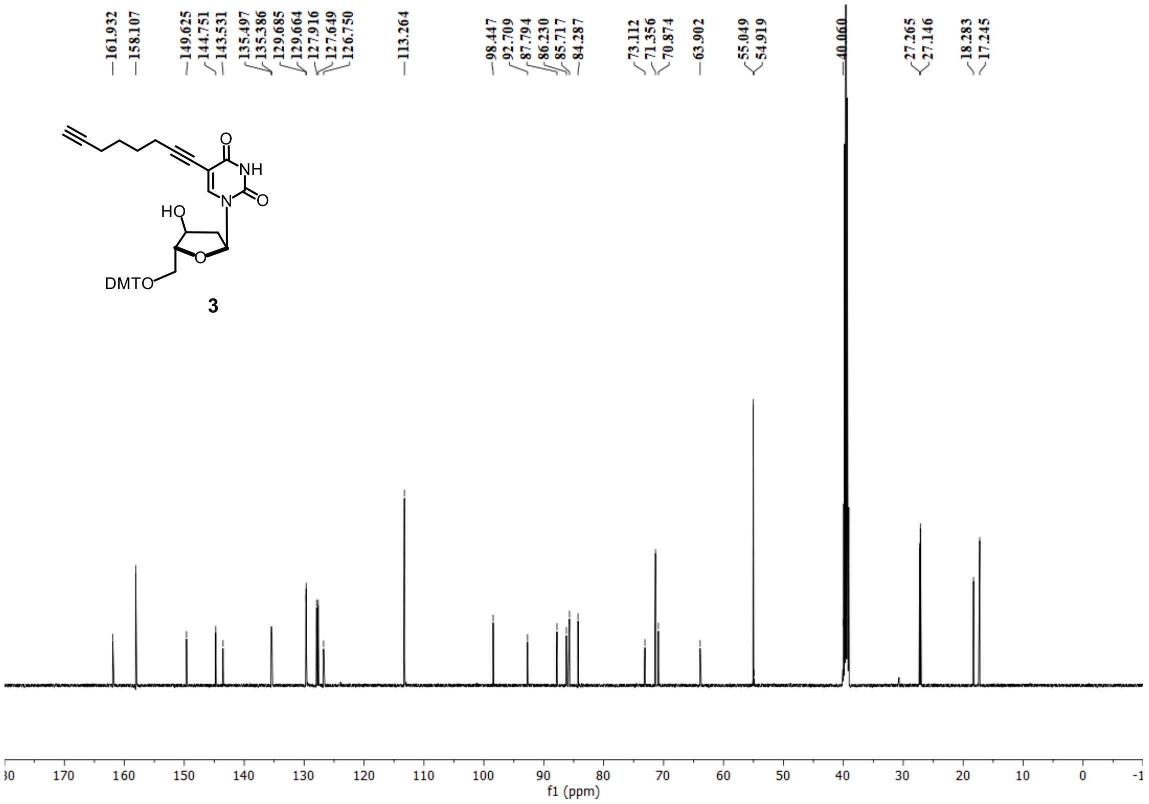

Figure S16. ^{13}C NMR (DMSO-d_6,150 MHz) spectrum of compound **3**.

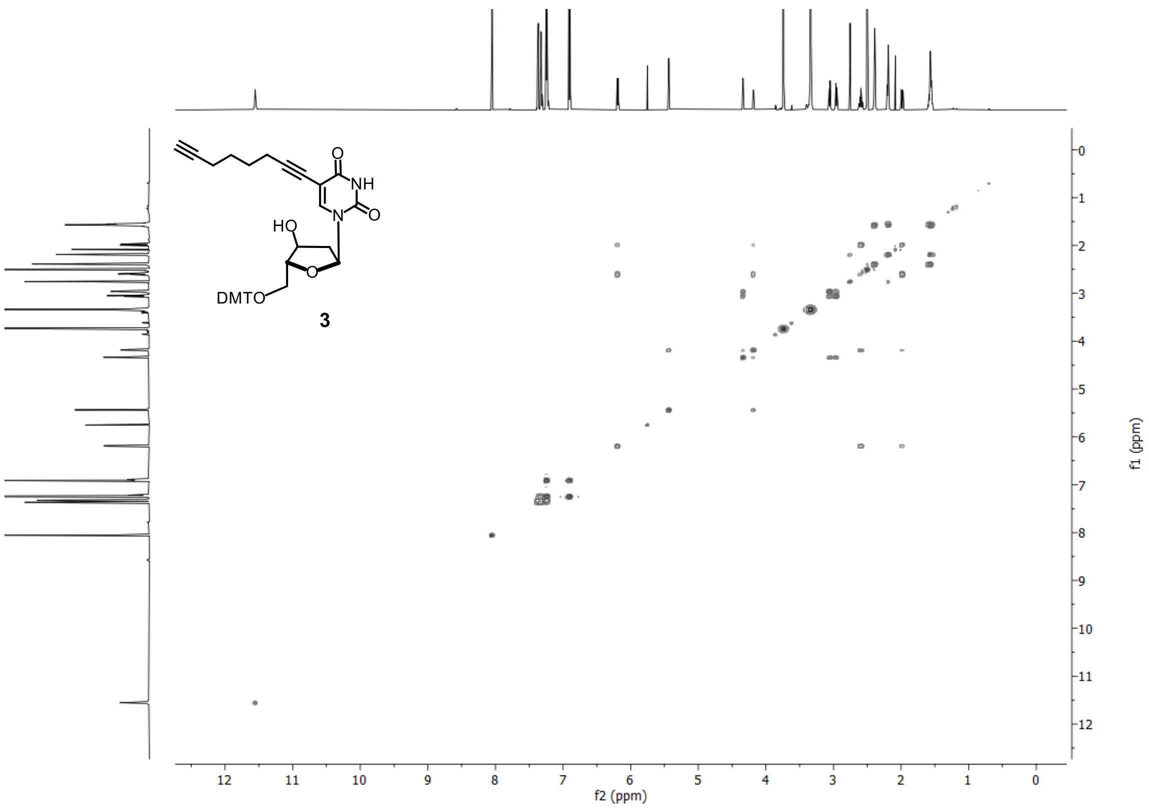

Figure S17. COSY NMR (DMSO-d_6, 600 MHz) spectrum of compound **3**.

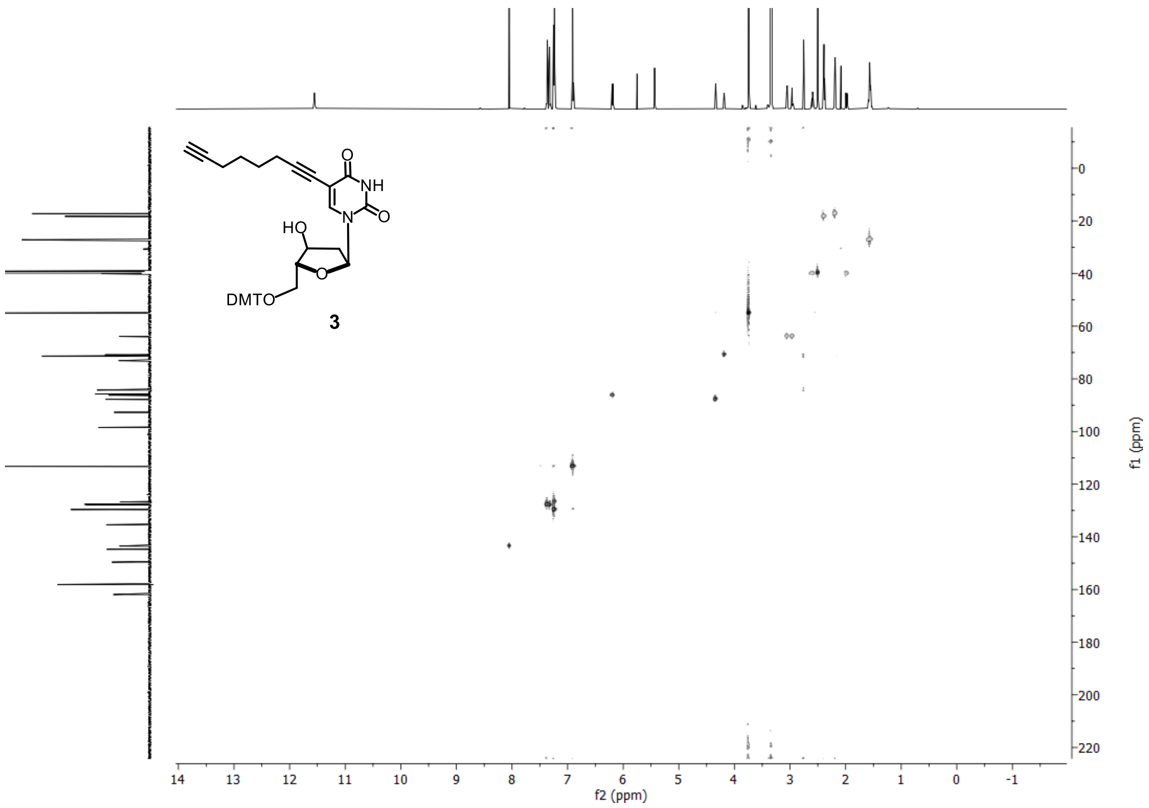

Figure S18. HSQC NMR (DMSO-d_6, 600 MHz) spectrum of compound **3**.

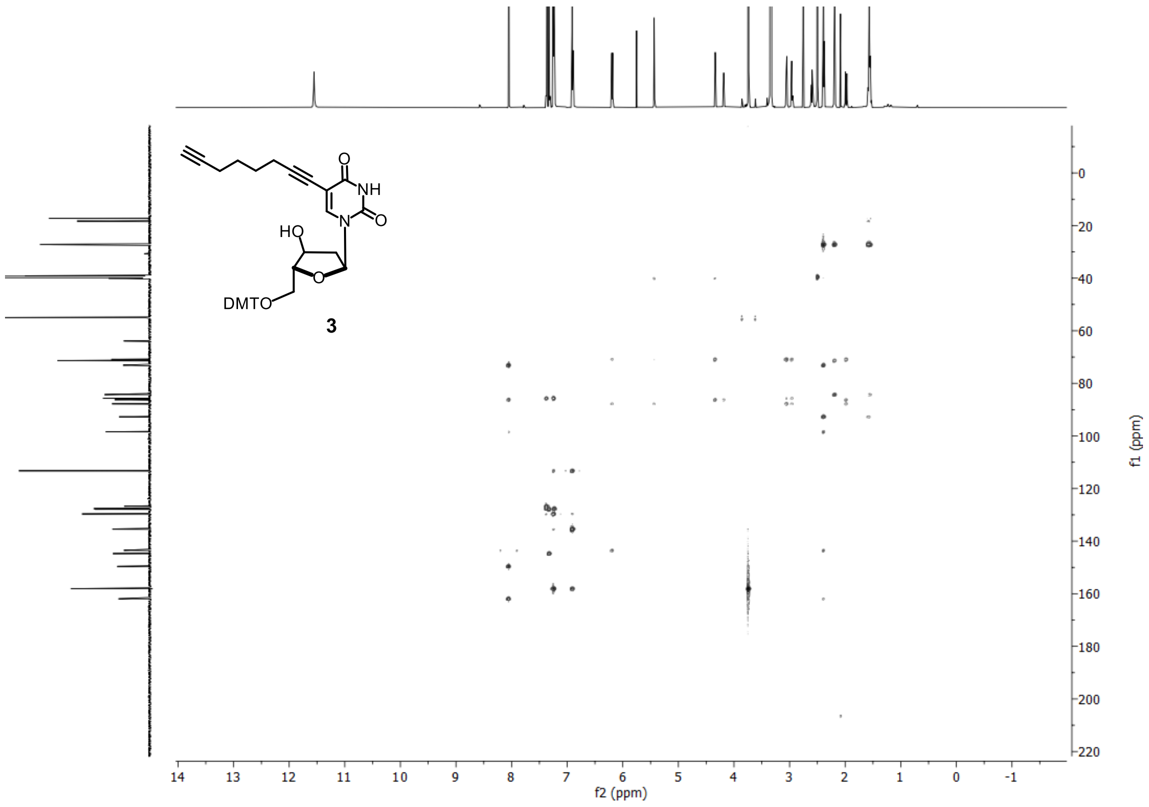

Figure S19. HMBC NMR (DMSO-d_6, 600 MHz) spectrum of compound **3**.

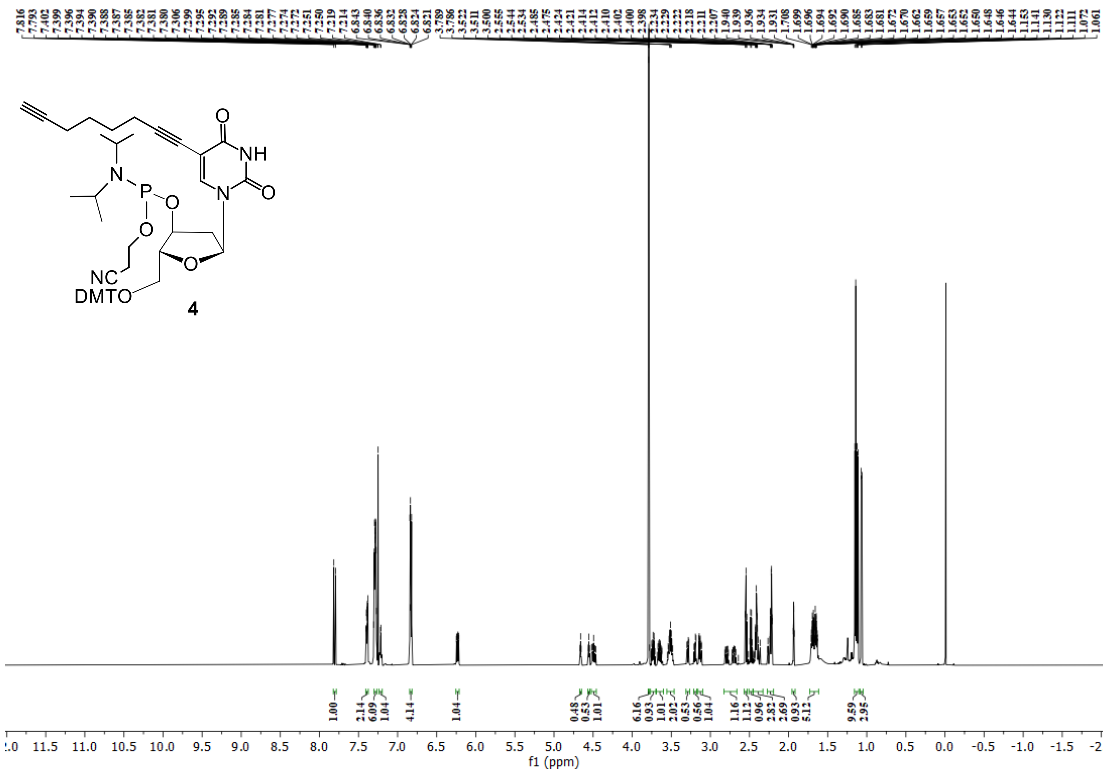

Figure S20. ^{1}H NMR ($CDCl_3$, 600 MHz) spectrum of compound **4**.

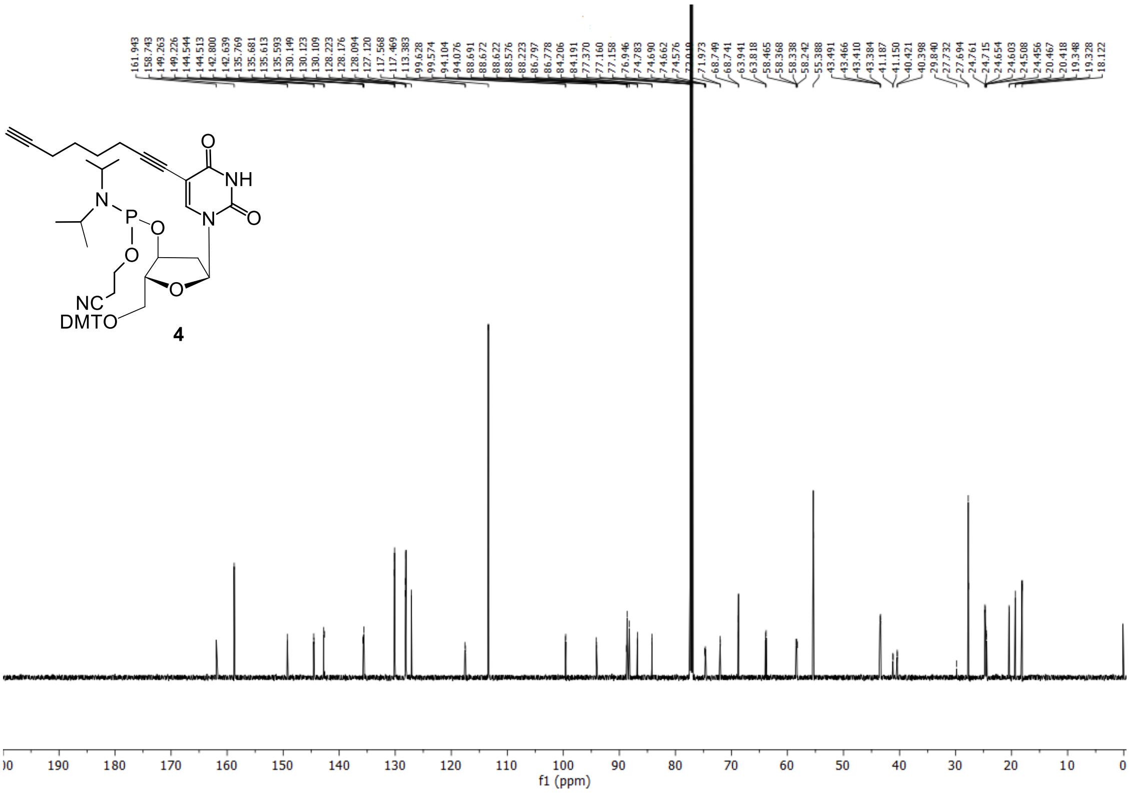

Figure S21. ^{13}C NMR ($CDCl_3$, 150 MHz) spectrum of compound **4**.

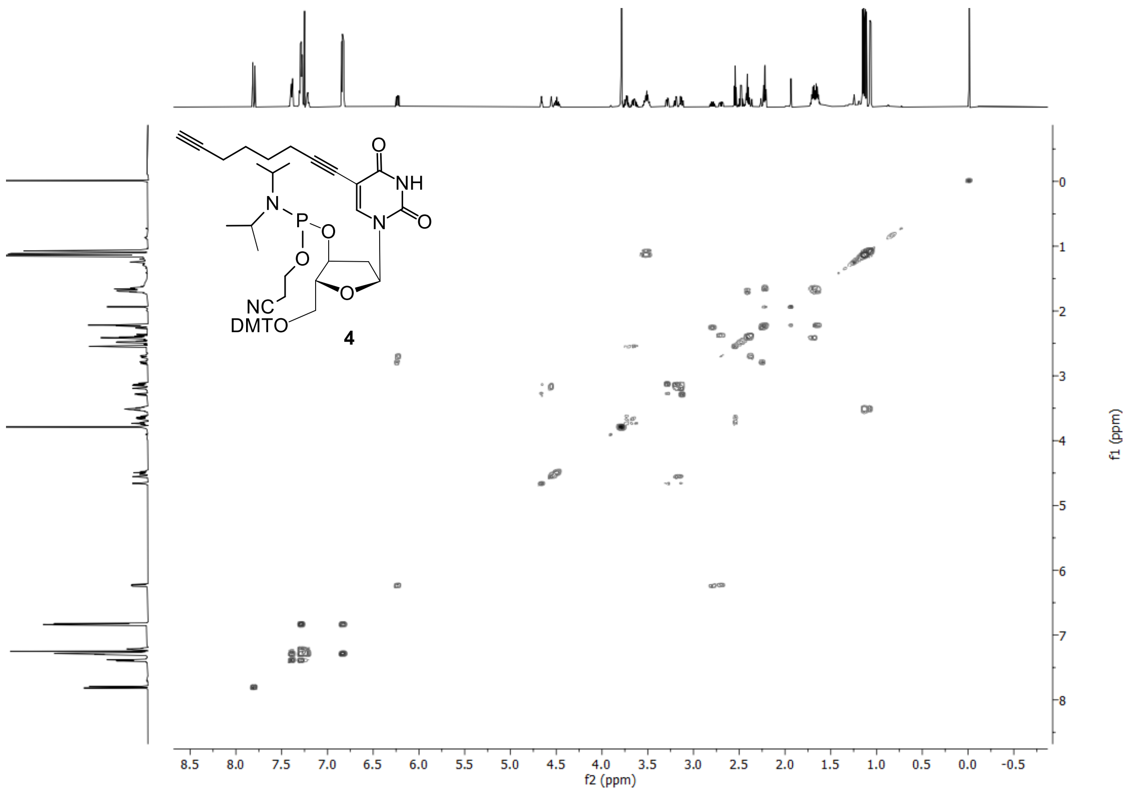

Figure S22. COSY NMR ($CDCl_3$, 600 MHz) spectrum of compound **4**.

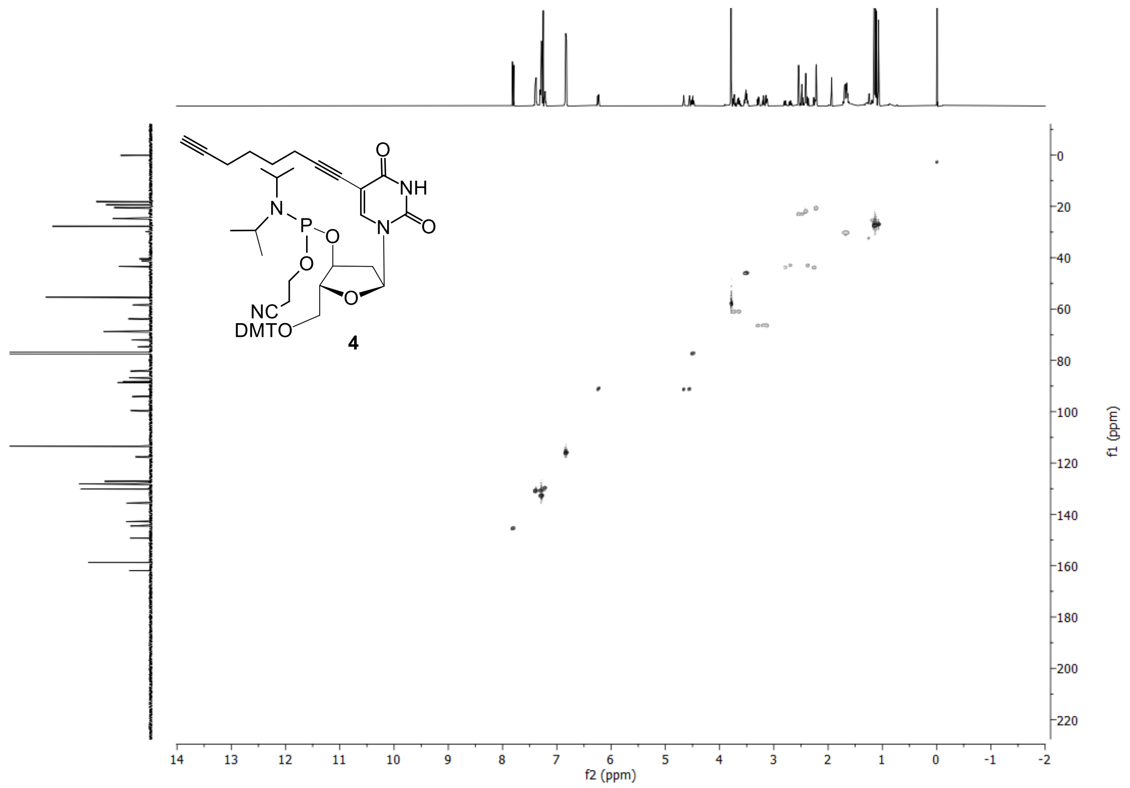

Figure S23. HSQC NMR ($CDCl_3$, 600 MHz) spectrum of compound **4**.

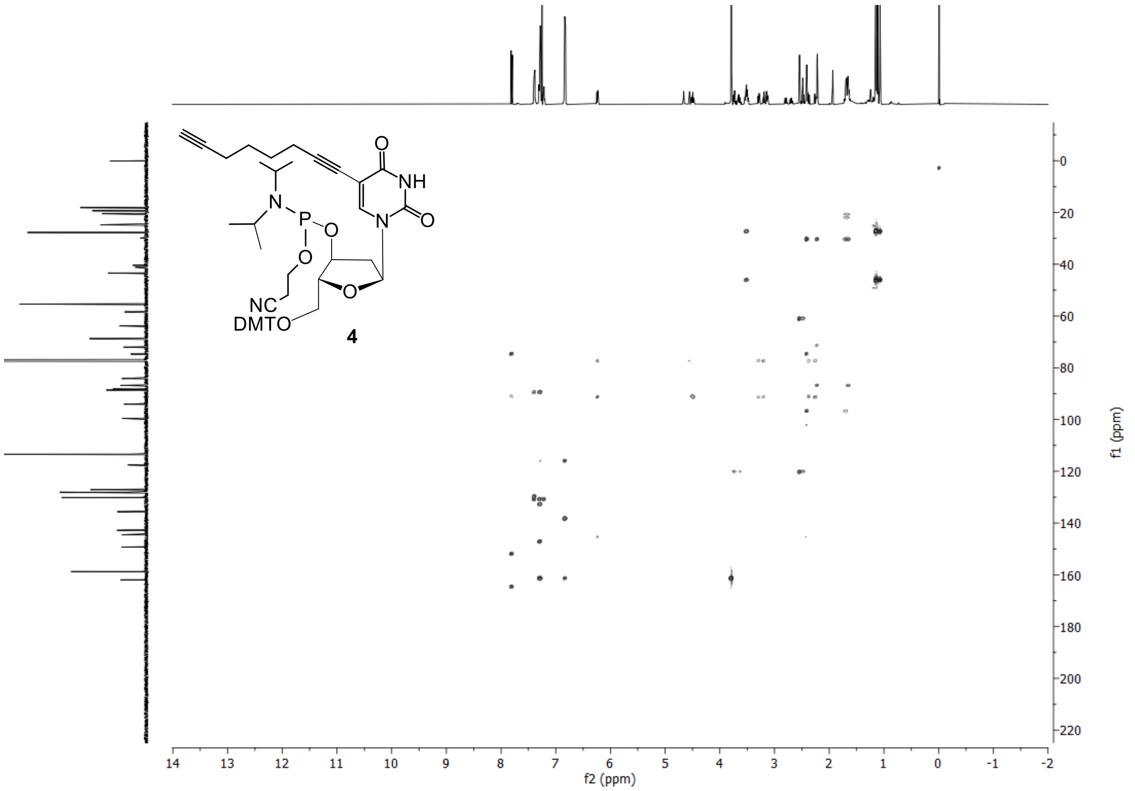

Figure S24. HMBC NMR ($CDCl_3$, 600 MHz) spectrum of compound **4**.

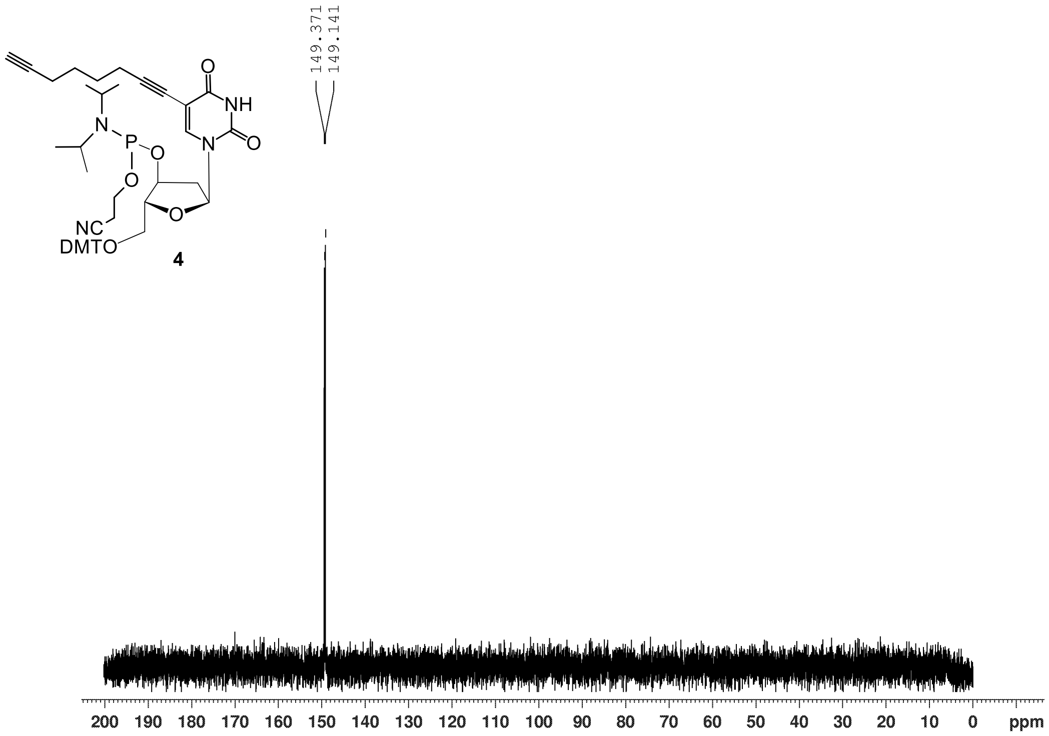

Figure S25. ^{31}P NMR ($CDCl_3$, 121 MHz) spectrum of compound **4**.

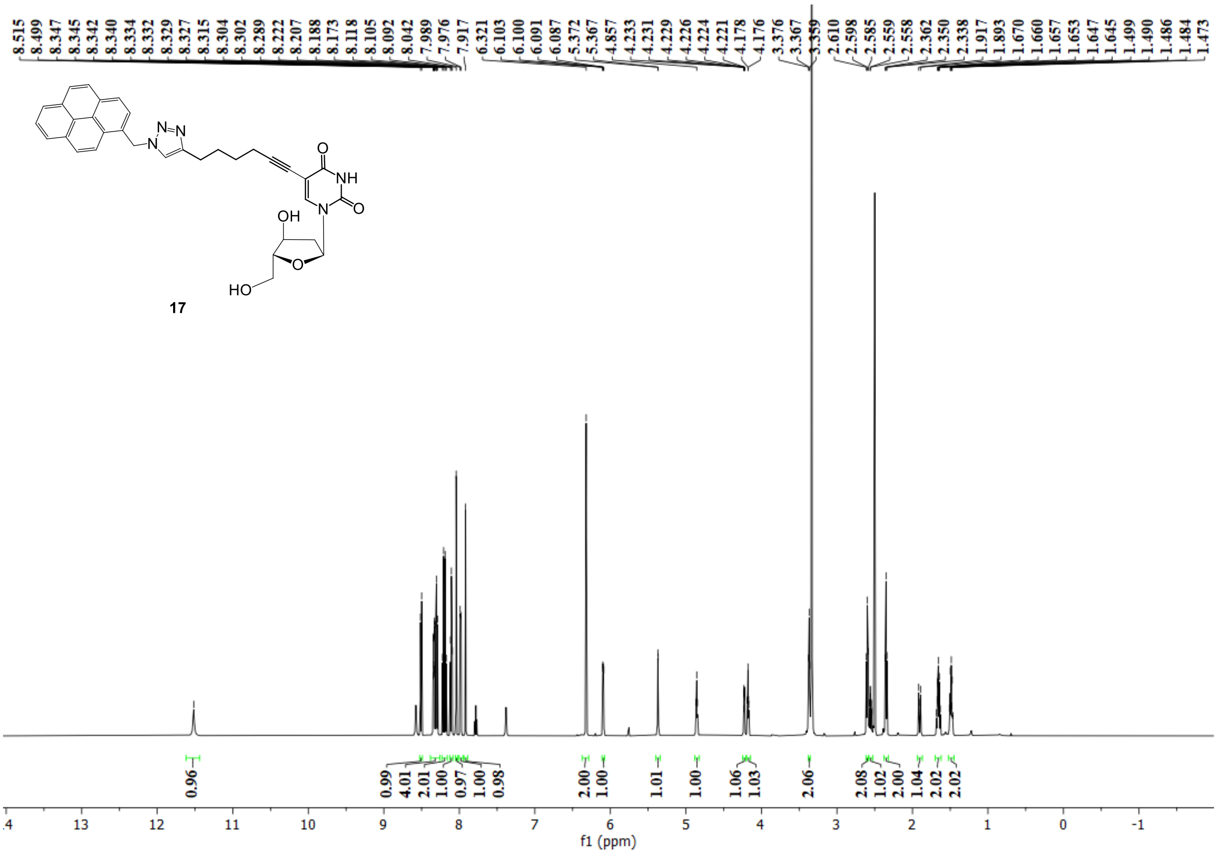

Figure S26. ^{1}H NMR (DMSO-d_6, 600 MHz) spectrum of compound **17**.

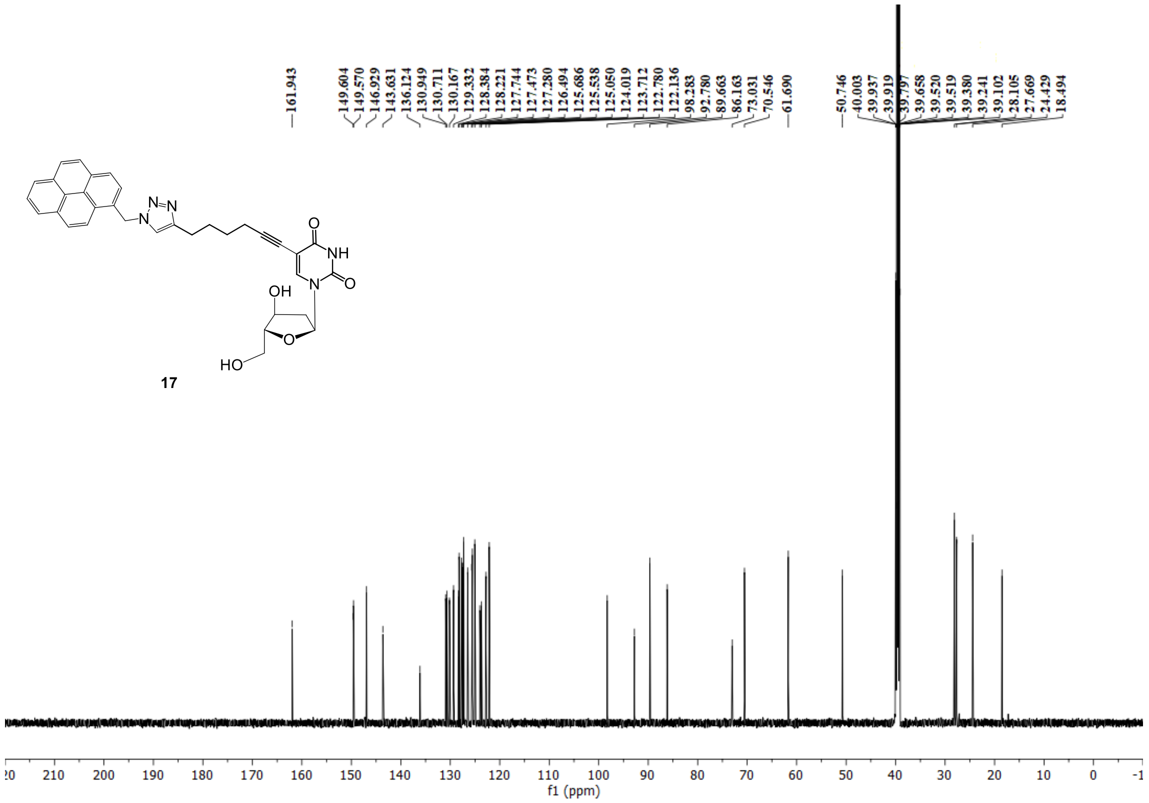

Figure S27. ^{13}C NMR (DMSO-d_6, 150 MHz) spectrum of compound **17**.

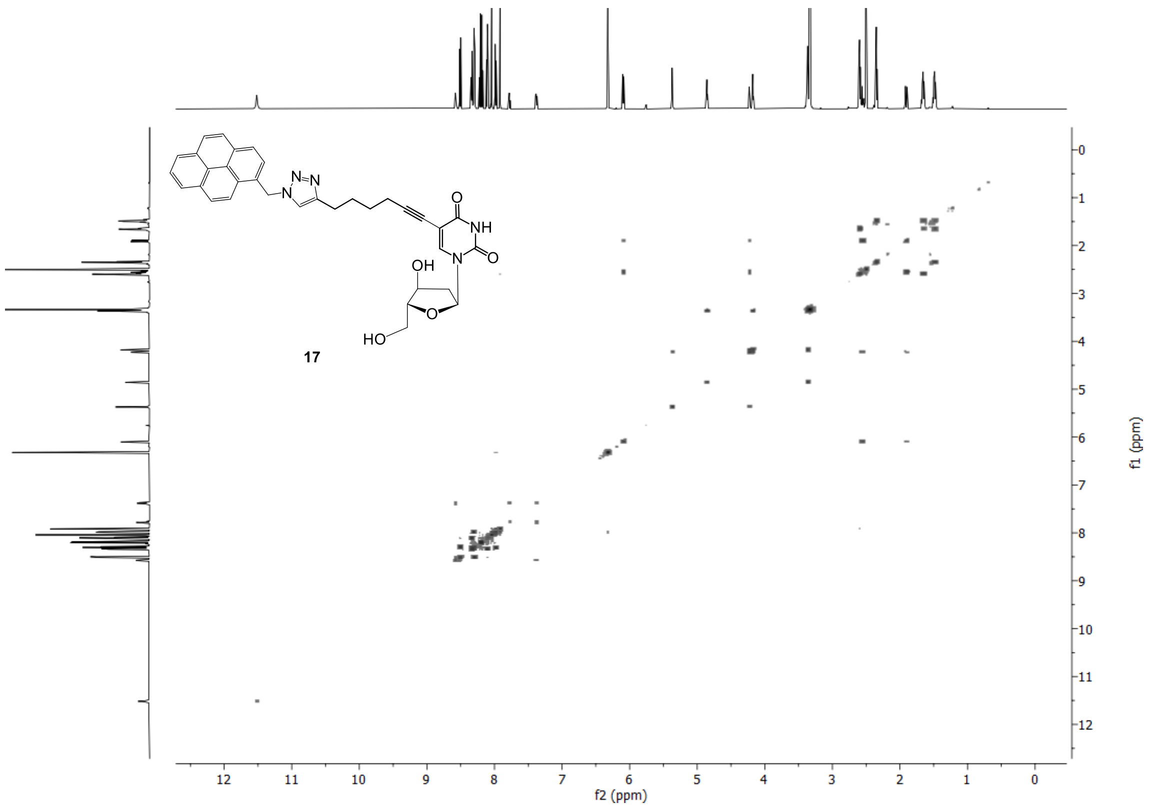

Figure S28. COSY NMR (DMSO-d_6, 600 MHz) spectrum of compound **17**.

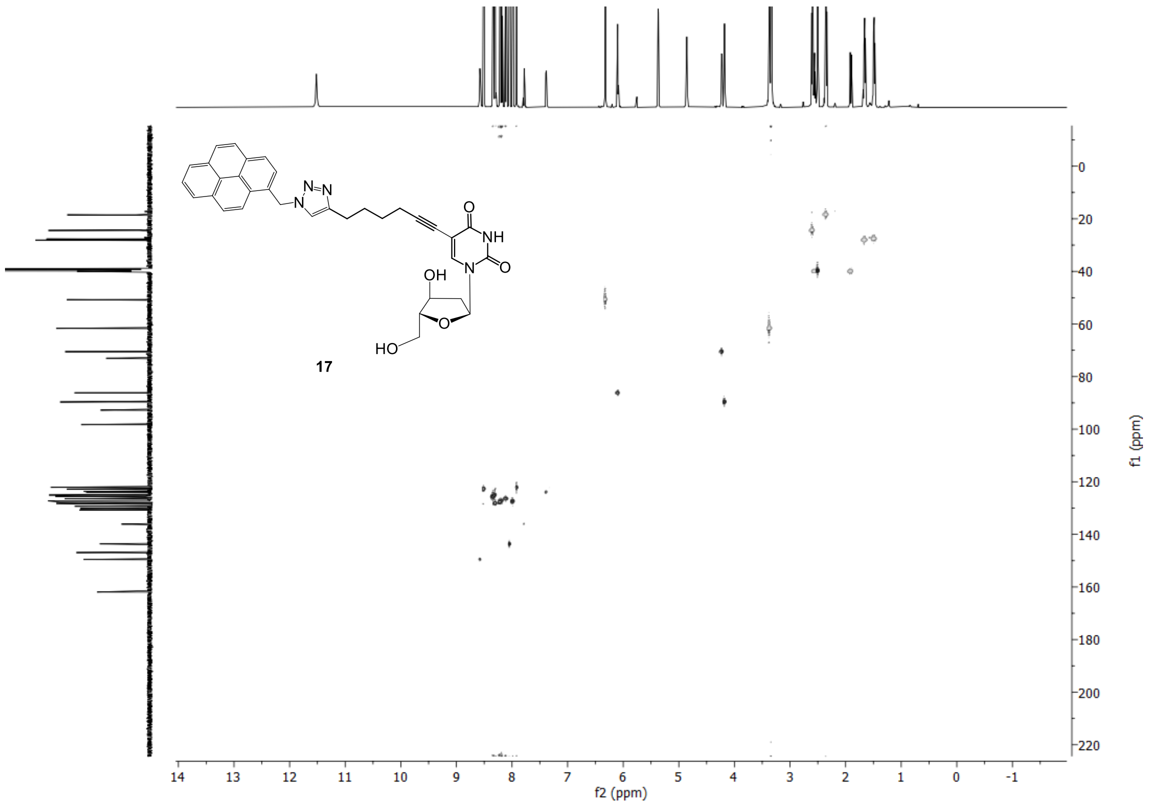

Figure S29. HSQC NMR (DMSO-d_6, 600 MHz) spectrum of compound **17**.

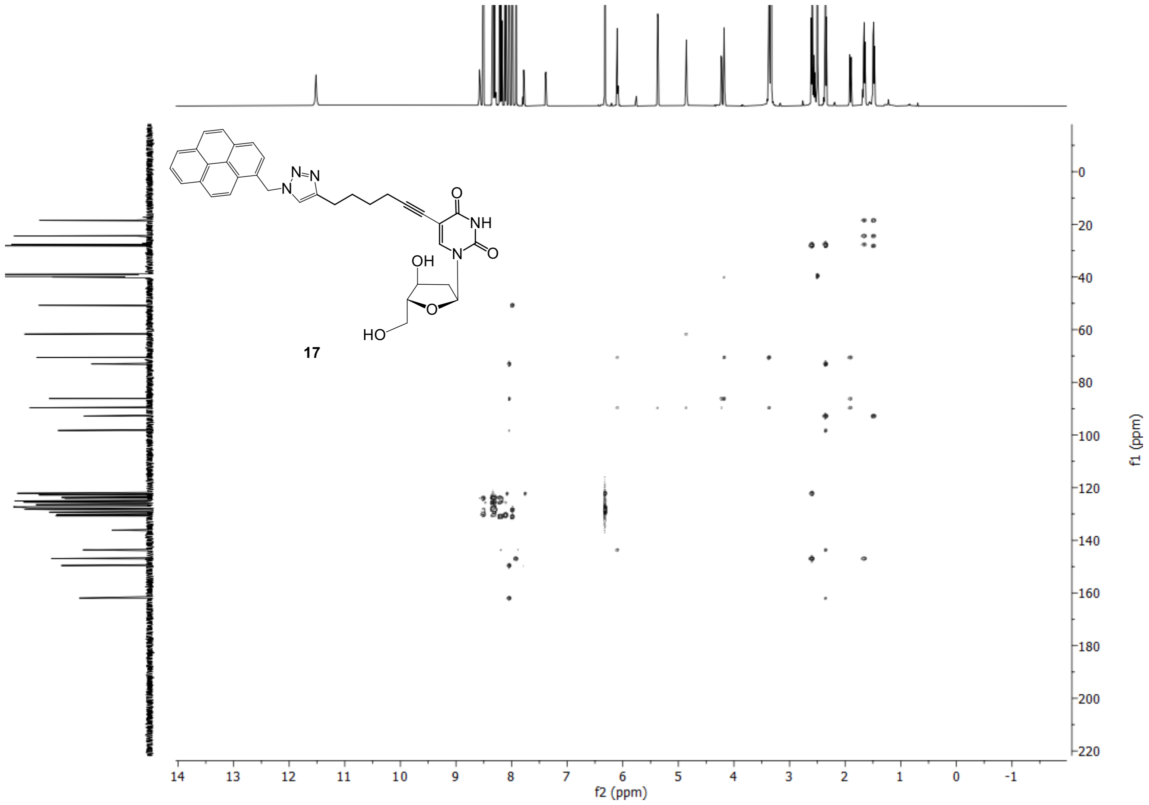

Figure S30. HMBC NMR (DMSO-d_6, 600 MHz) spectrum of compound **17**.

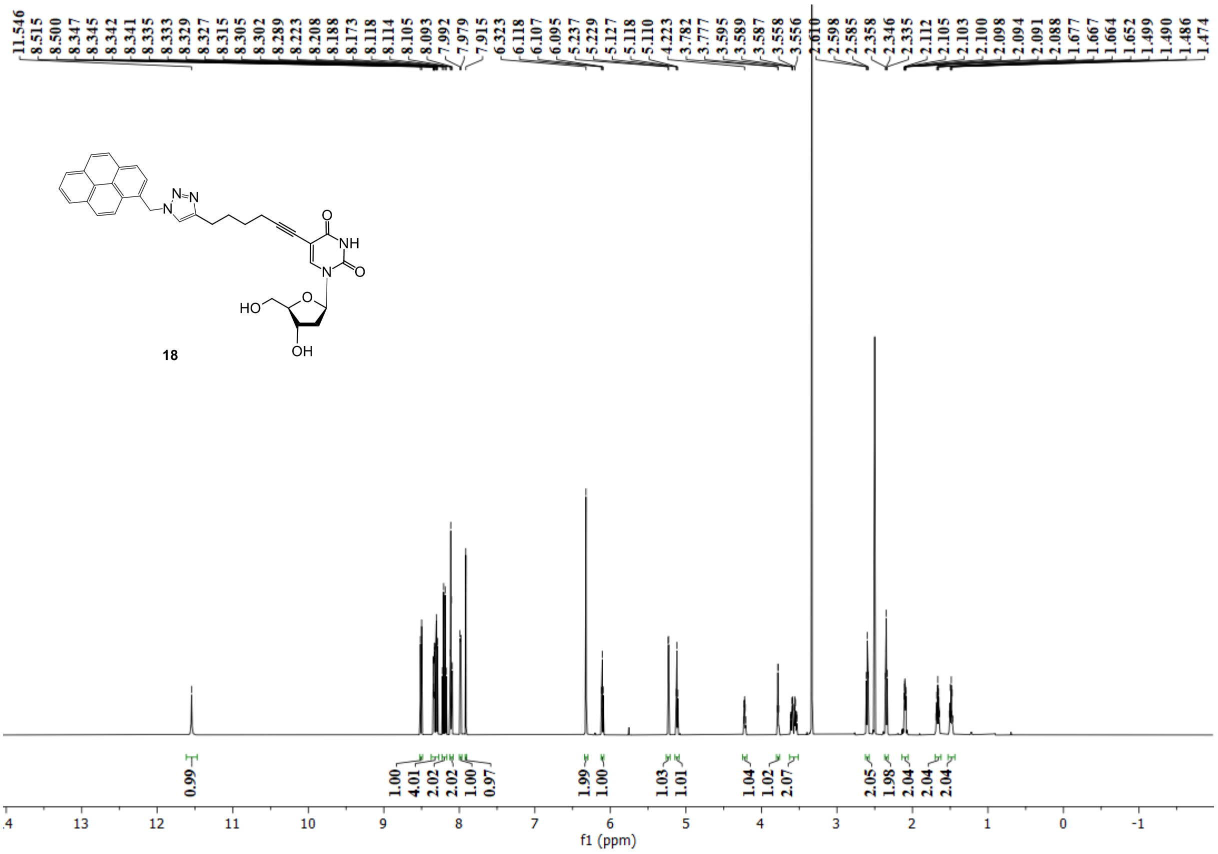

Figure S31. ^{1}H NMR (DMSO-d_6, 600 MHz) spectrum of compound **18**.

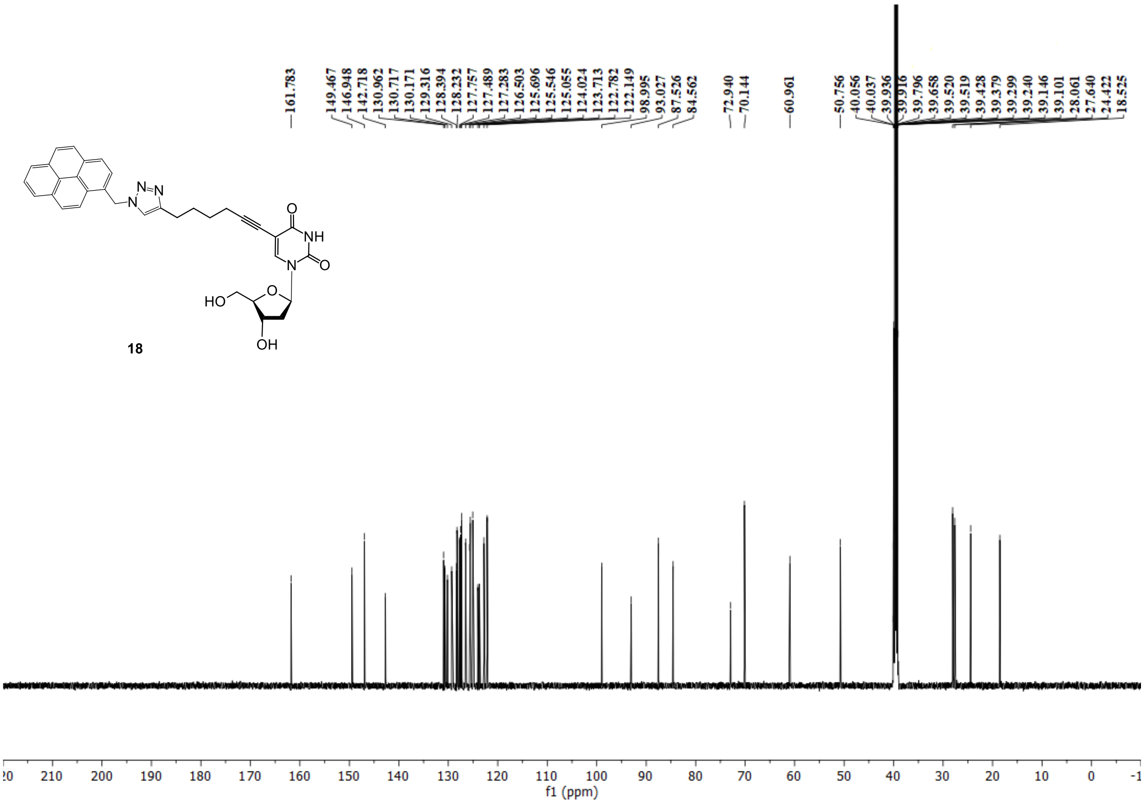

Figure S32. ^{13}C NMR (DMSO-d_6, 150 MHz) spectrum of compound **18**.

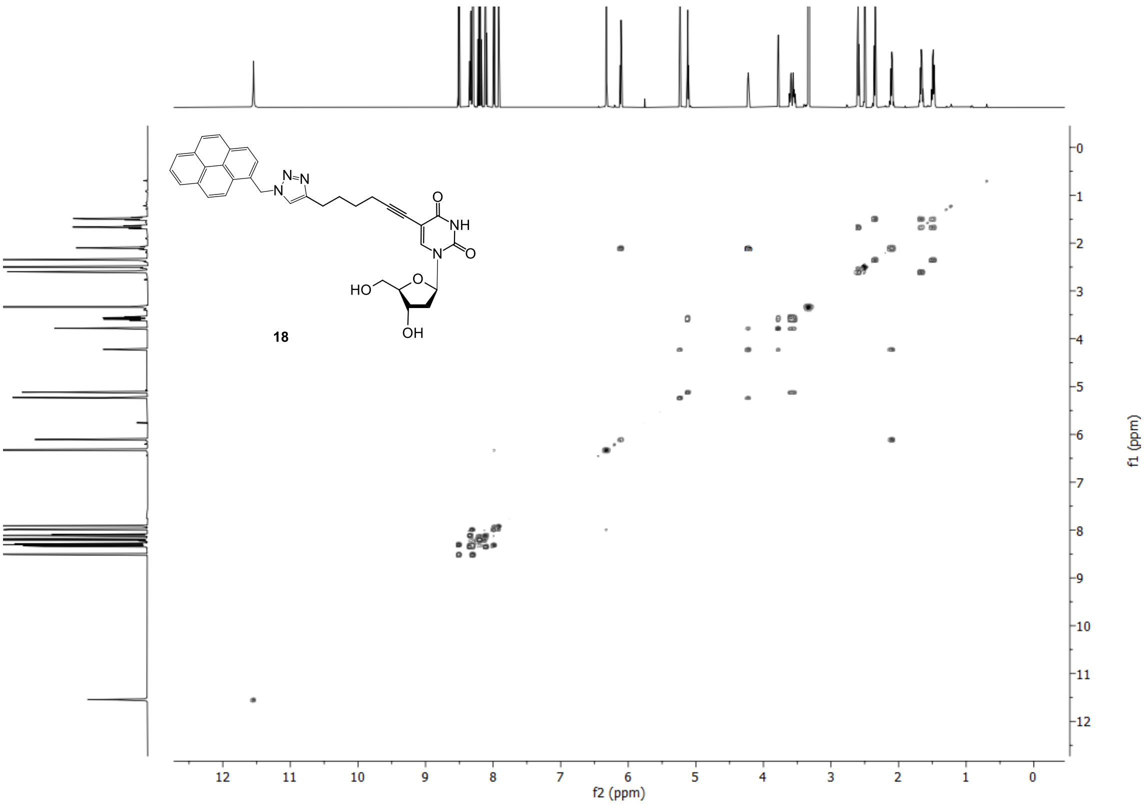

Figure S33. COSY NMR (DMSO-d_6, 600 MHz) spectrum of compound **18**.

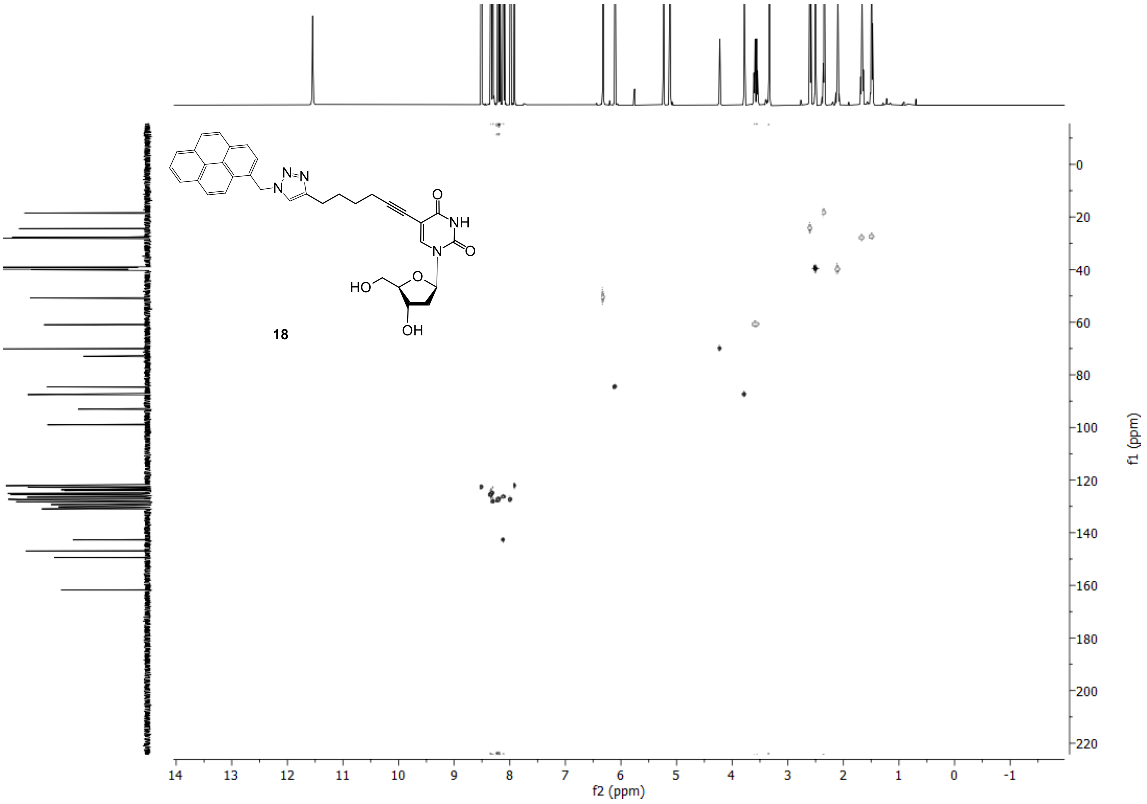

Figure S34. HSQC NMR (DMSO-d_6, 600 MHz) spectrum of compound **18**.

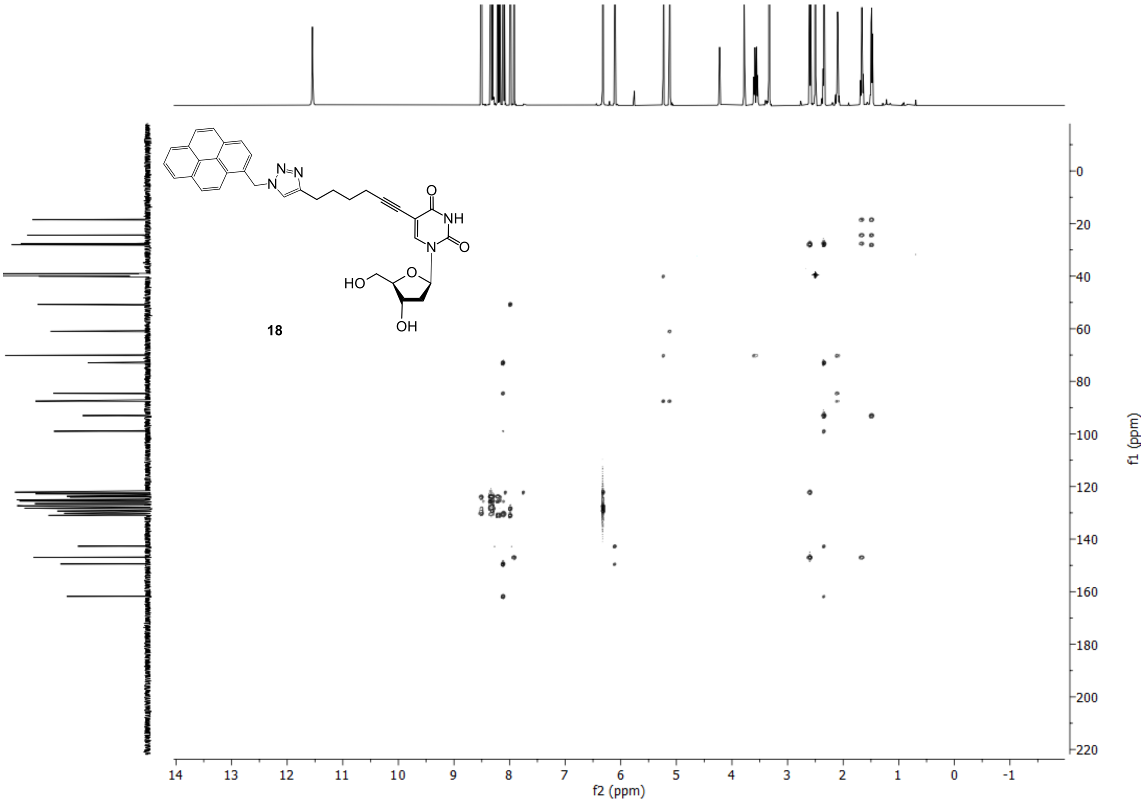

Figure S35. HMBC NMR (DMSO-d_6, 600 MHz) spectrum of compound **18**.

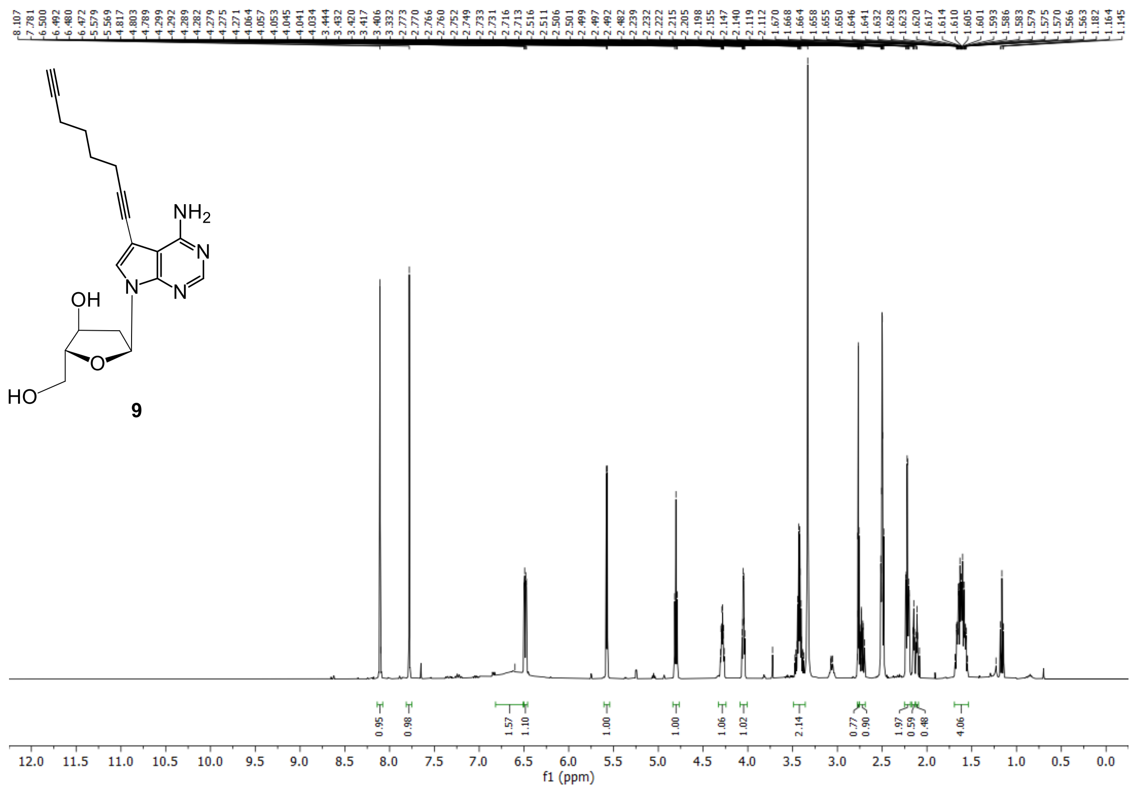

Figure S36. ^{1}H NMR (DMSO-d_6, 600 MHz) spectrum of compound **9**.

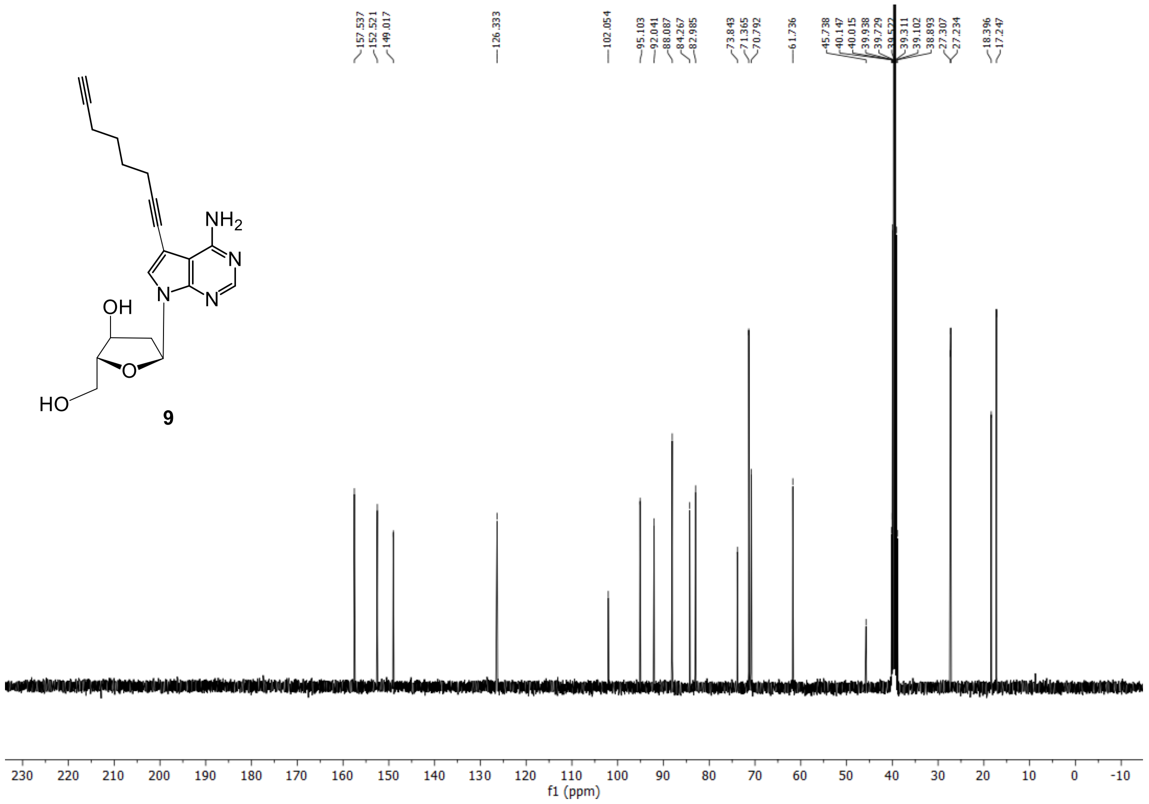

Figure S37. ^{13}C NMR (DMSO-d_6, 150 MHz) spectrum of compound **9**.

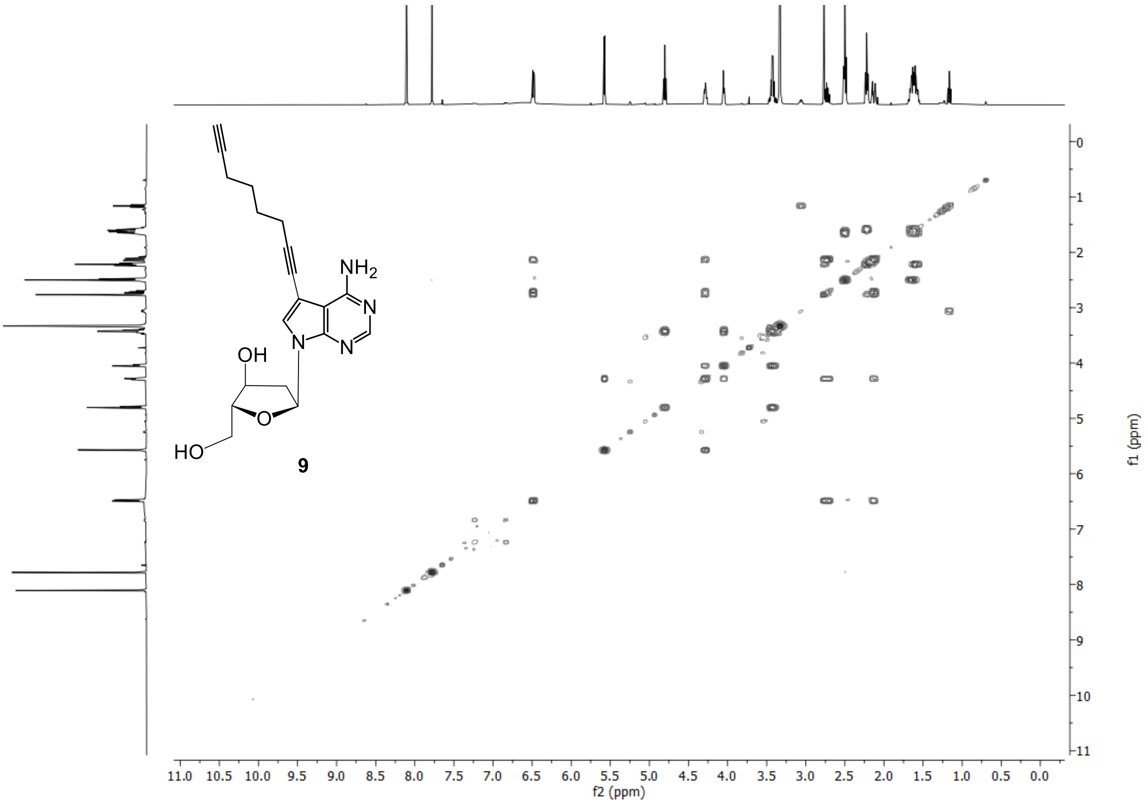

Figure S38. COSY NMR (DMSO-d_6, 600 MHz) spectrum of compound **9**.

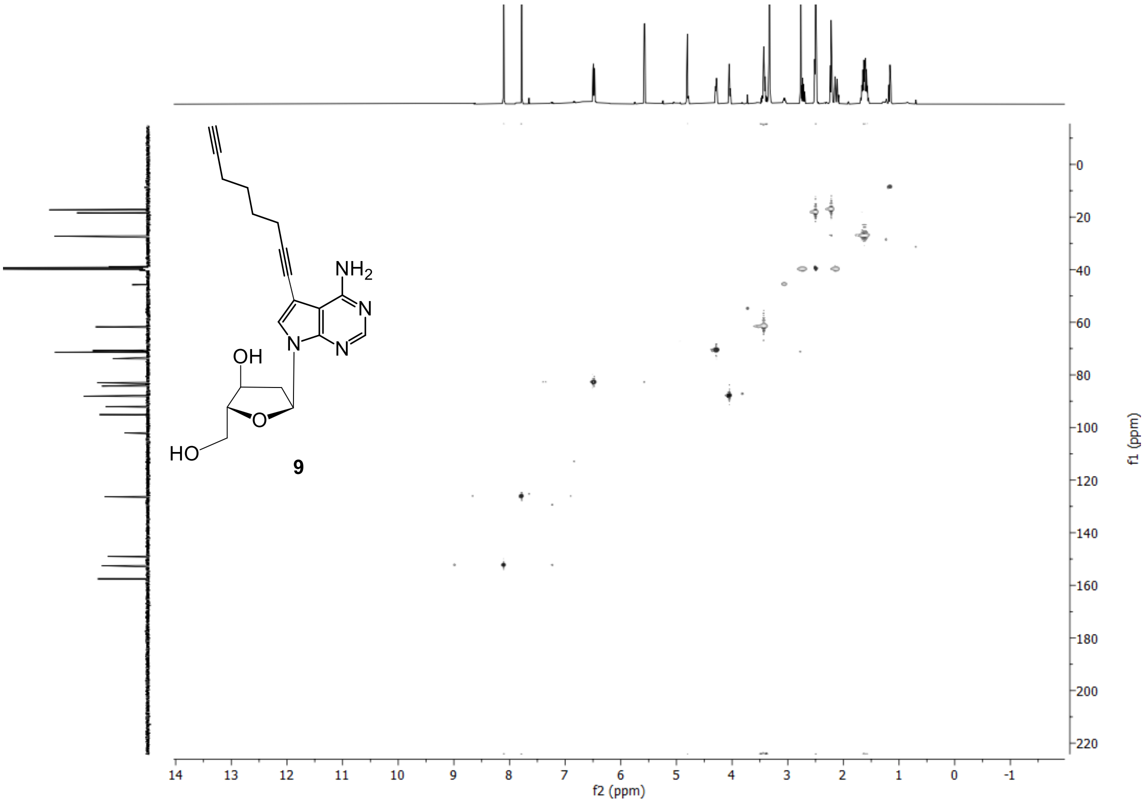

Figure S39. HSQC NMR (DMSO-d_6, 600 MHz) spectrum of compound **9**.

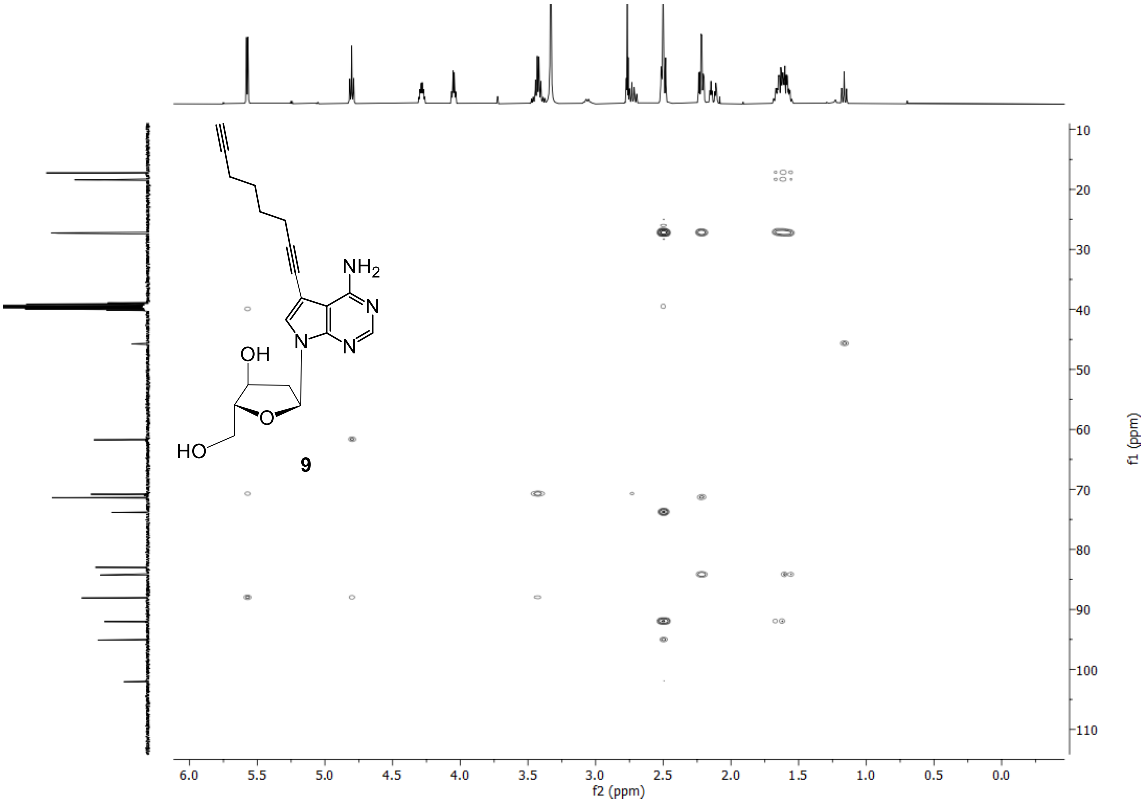

Figure S40. HMBC NMR (DMSO-d_6, 600 MHz) spectrum of compound **9**.

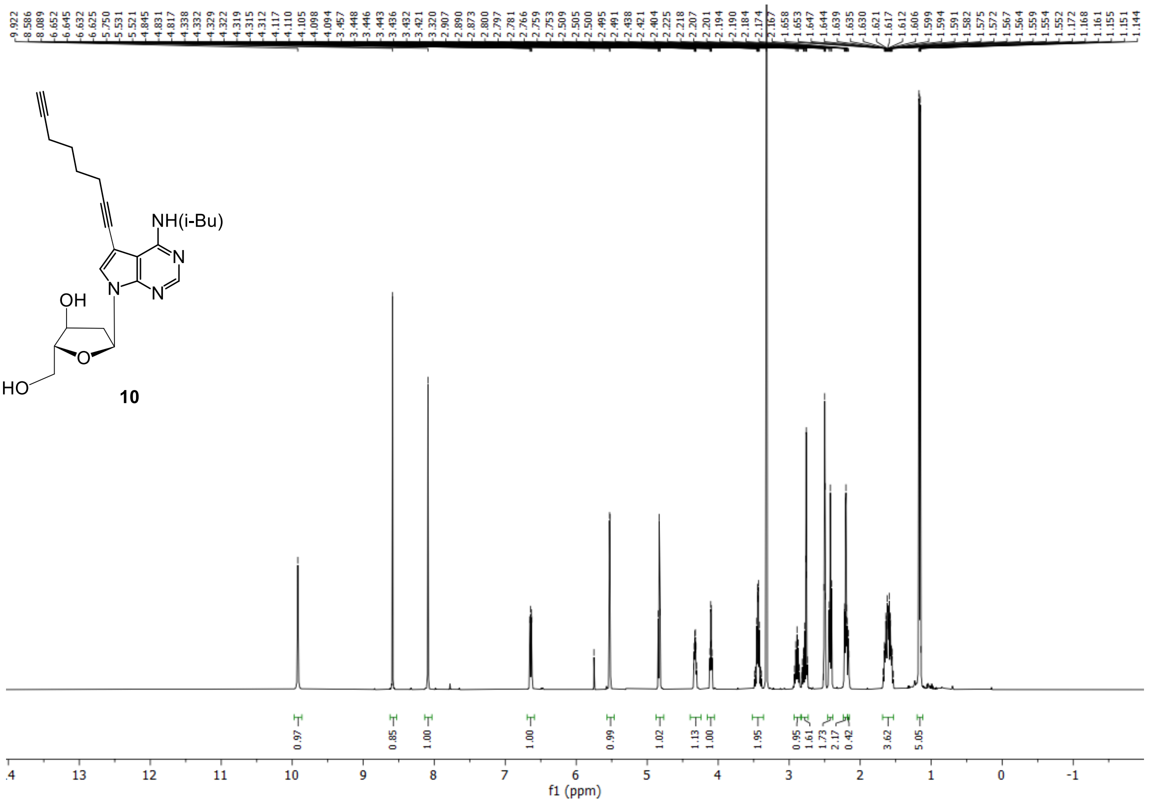

Figure S41. ^{1}H NMR (DMSO-d_6, 600 MHz) spectrum of compound **10**.

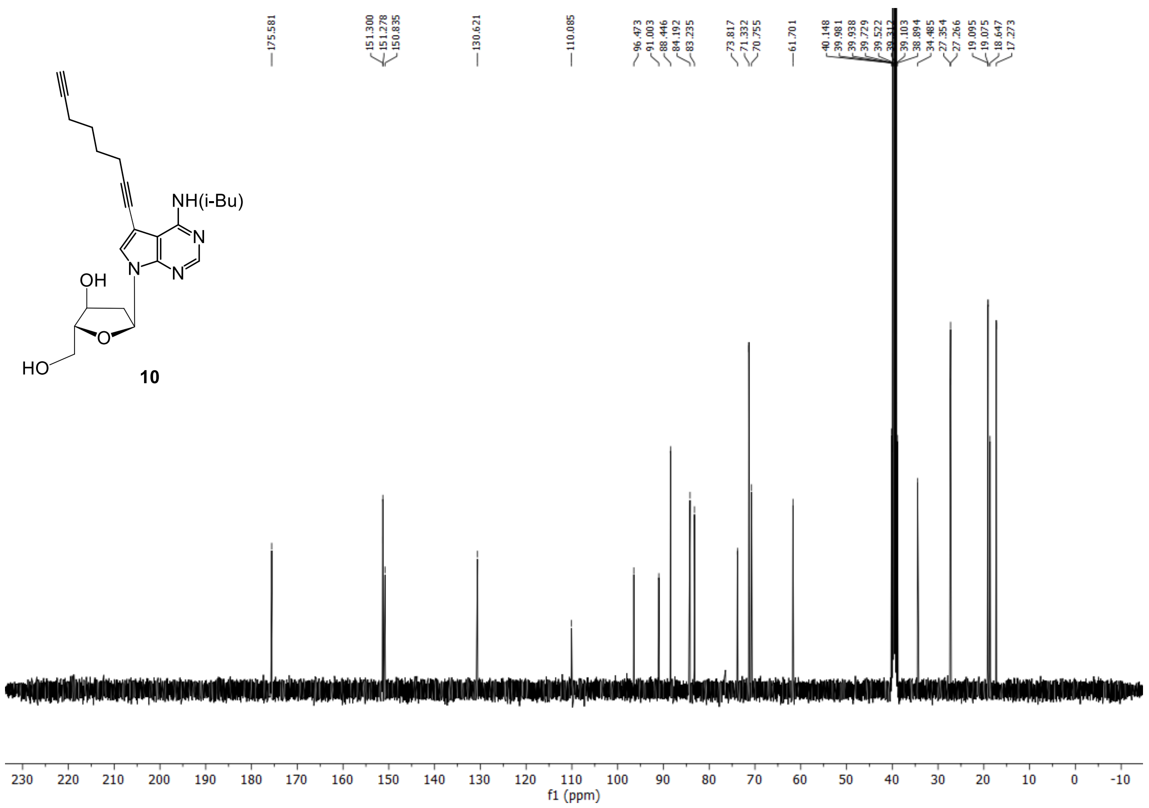

Figure S42. ^{13}C NMR (DMSO-d_6, 150 MHz) spectrum of compound **10**.

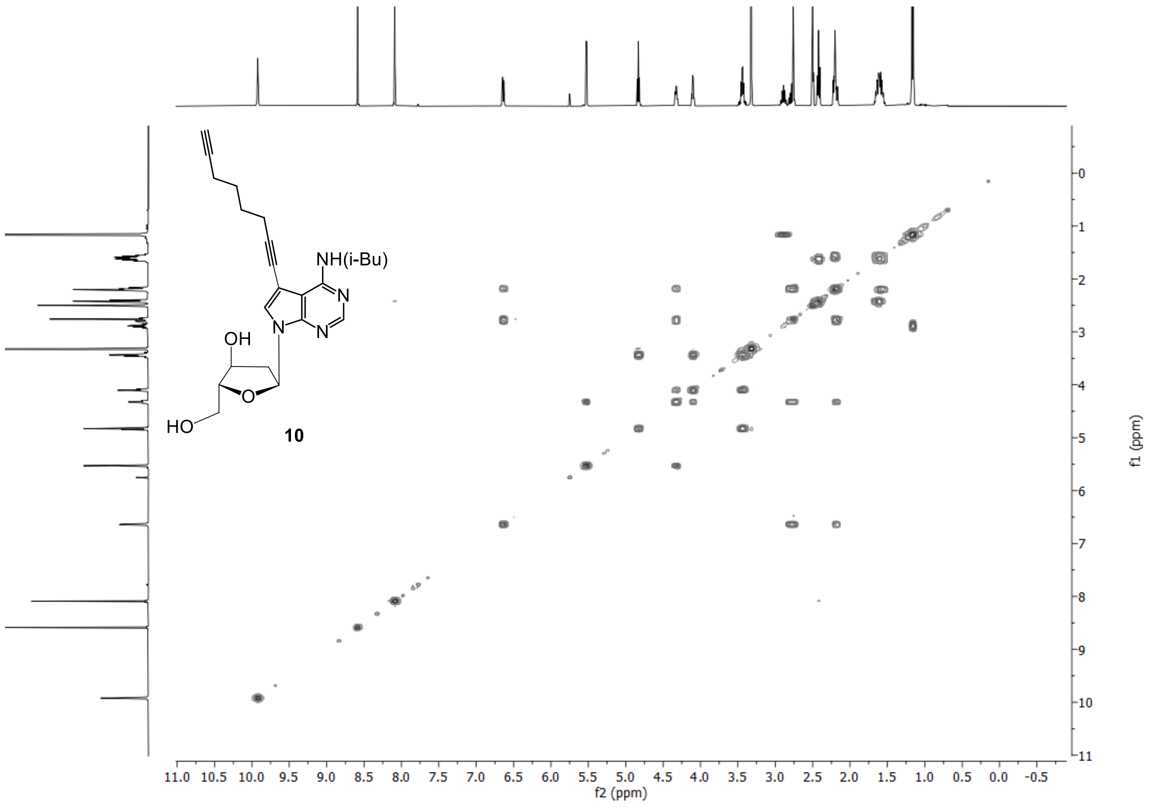

Figure S43. COSY NMR (DMSO-d_6, 600 MHz) spectrum of compound **10**.

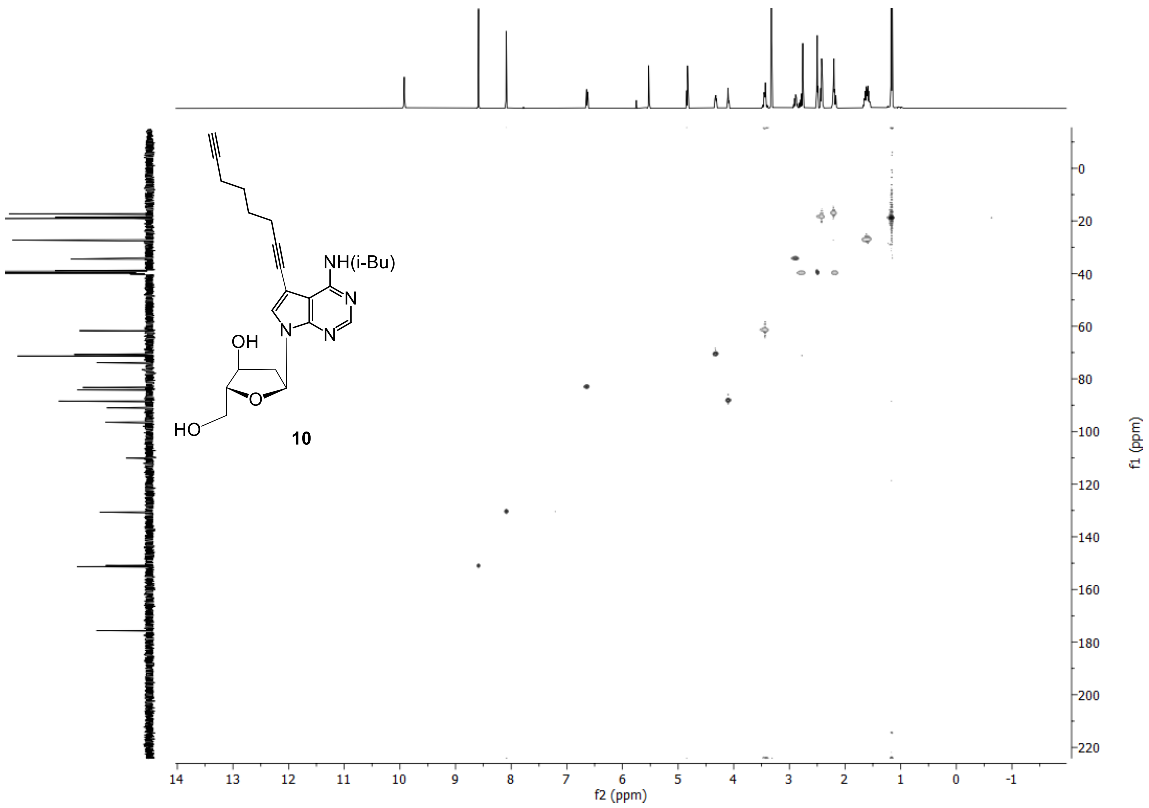

Figure S44. HSQC NMR (DMSO-d_6, 600 MHz) spectrum of compound **10**.

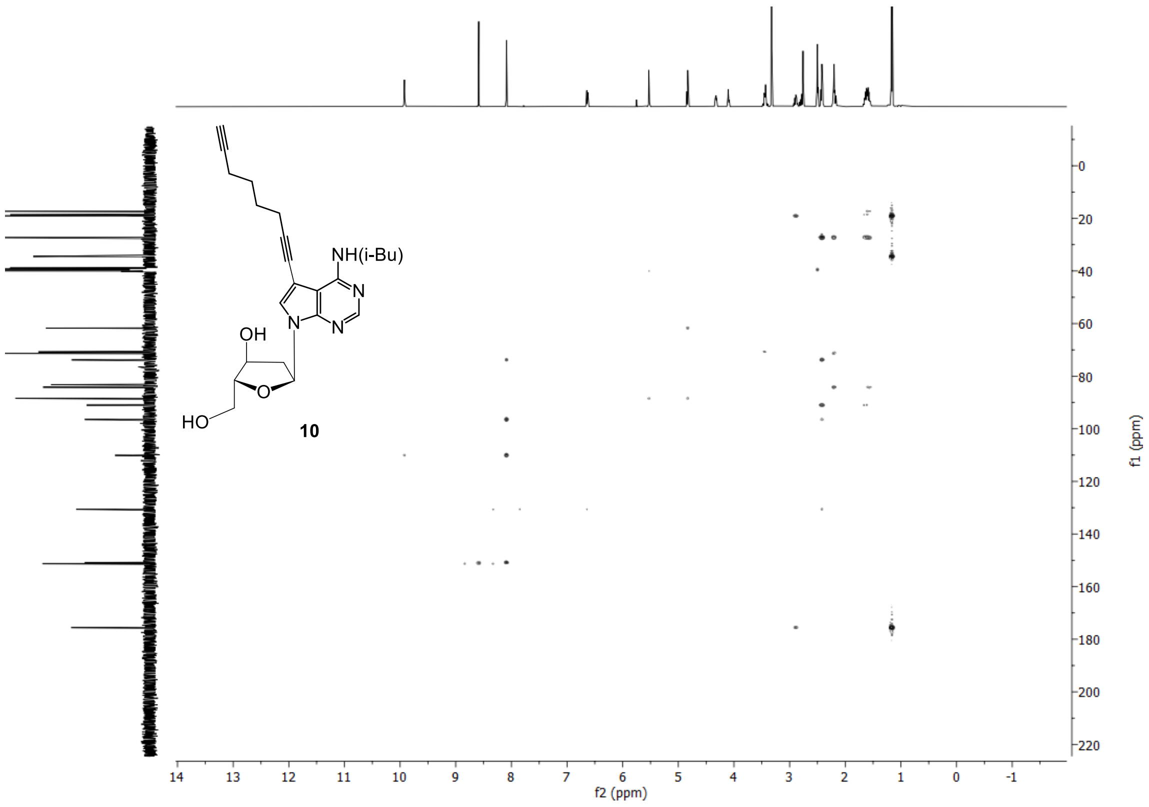

Figure S45. HMBC NMR (DMSO-d_6, 600 MHz) spectrum of compound **10**.

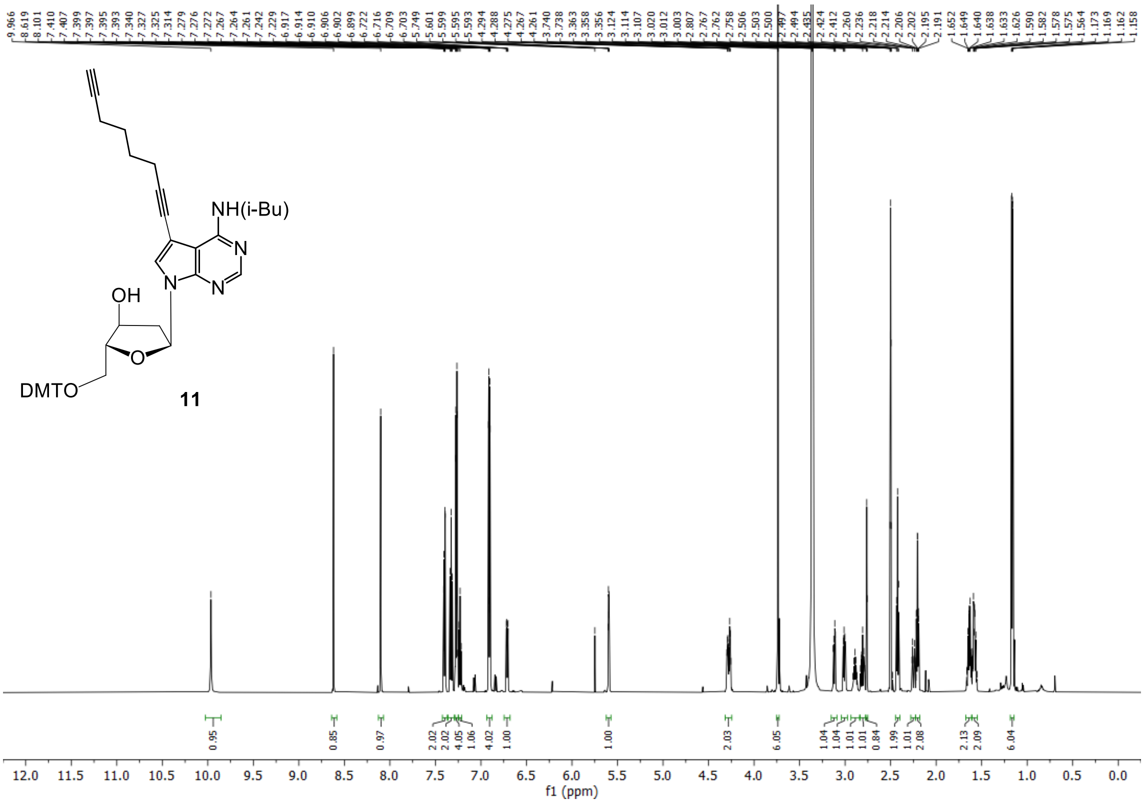

Figure S46. ^{1}H NMR (DMSO-d_6, 600 MHz) spectrum of compound **11**.

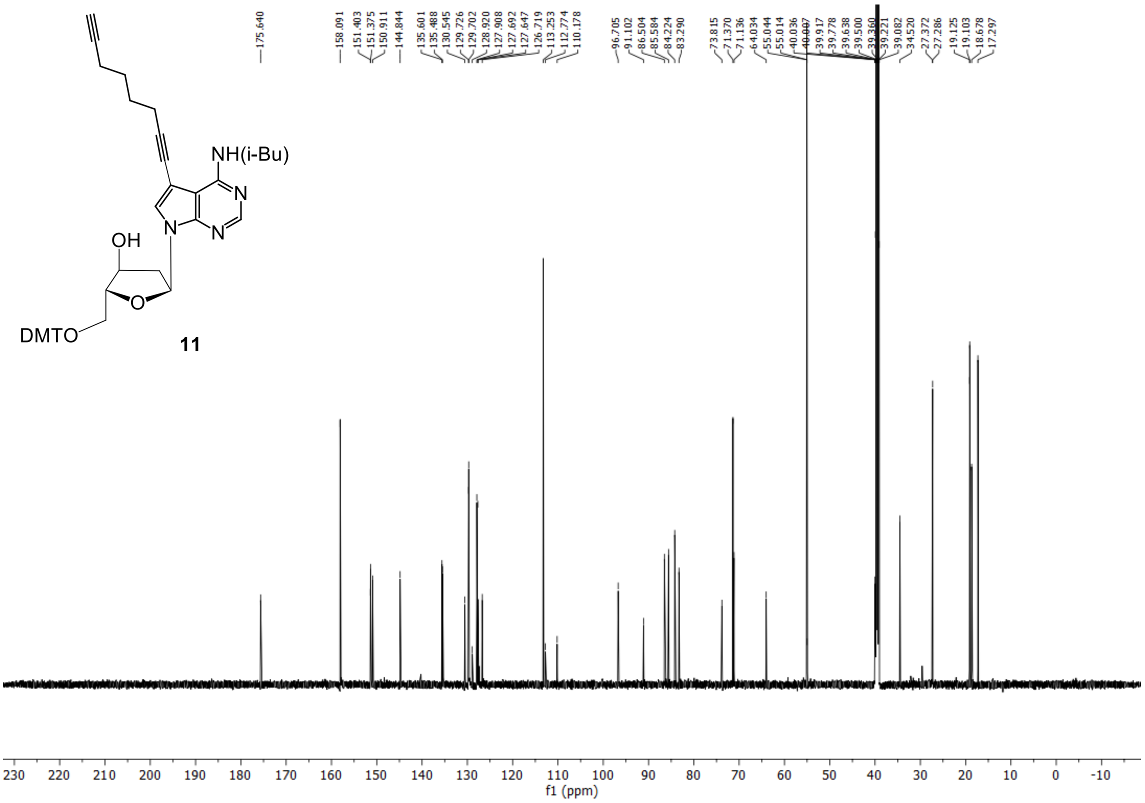

Figure S47. ^{13}C NMR (DMSO-d_6, 150 MHz) spectrum of compound **11**.

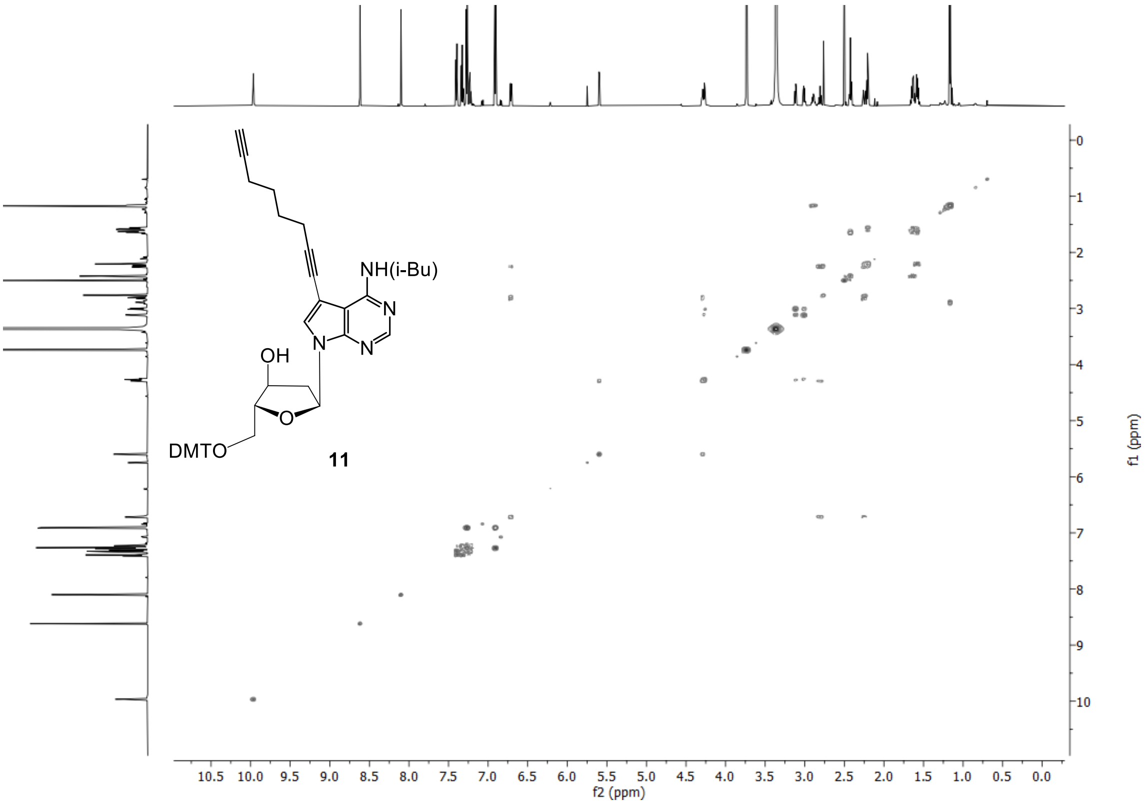

Figure S48. COSY NMR (DMSO-d_6, 600 MHz) spectrum of compound **11**.

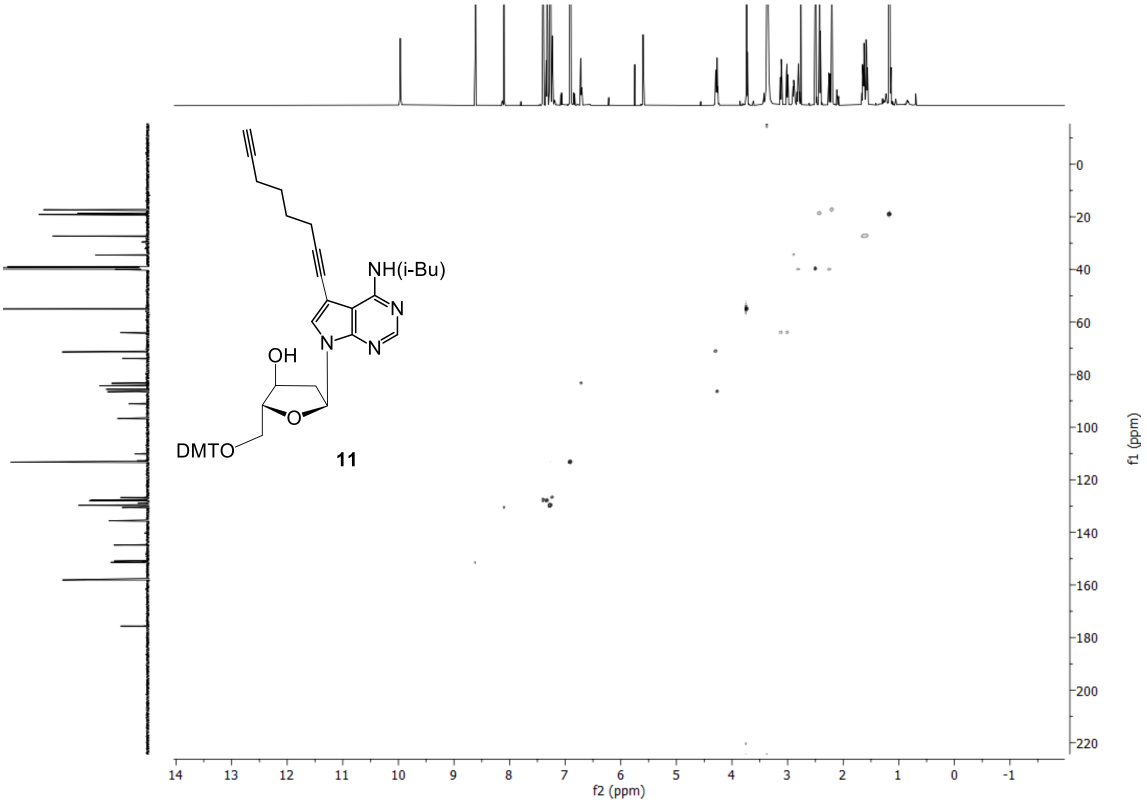

Figure S49. HSQC NMR (DMSO-d_6, 600 MHz) spectrum of compound **11**.

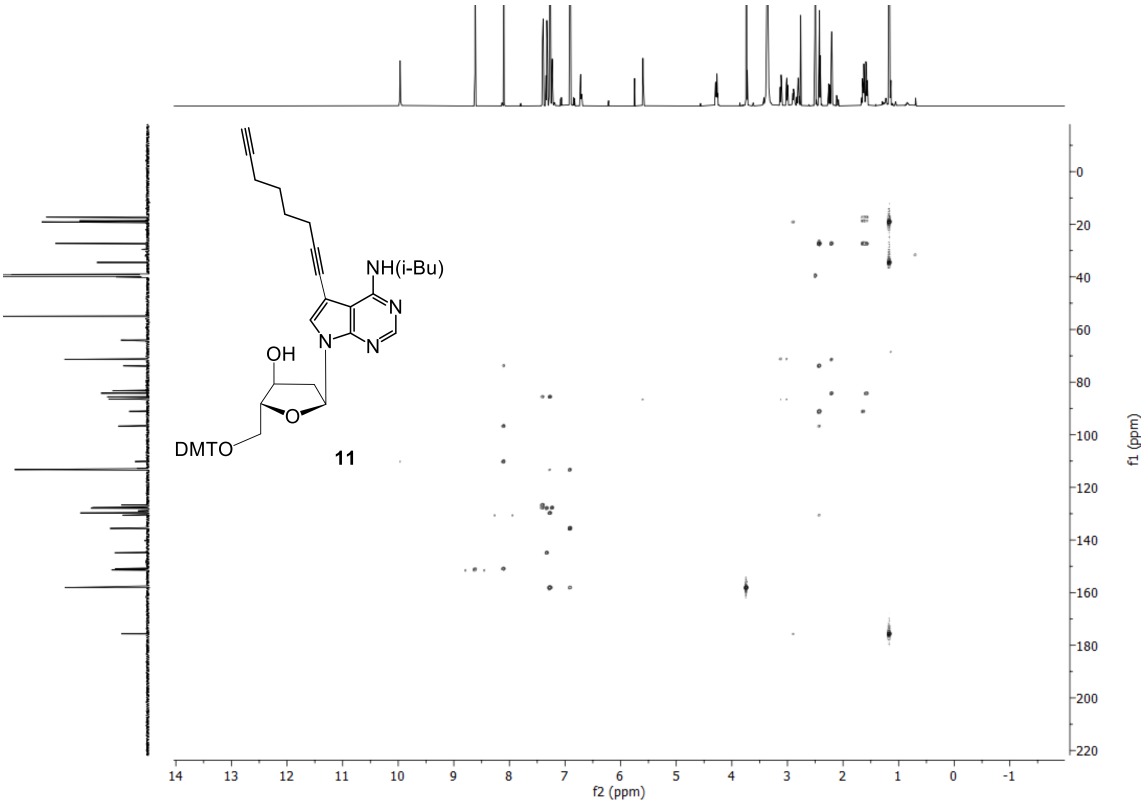

Figure S50. HMBC NMR (DMSO-d_6, 600 MHz) spectrum of compound **11**.

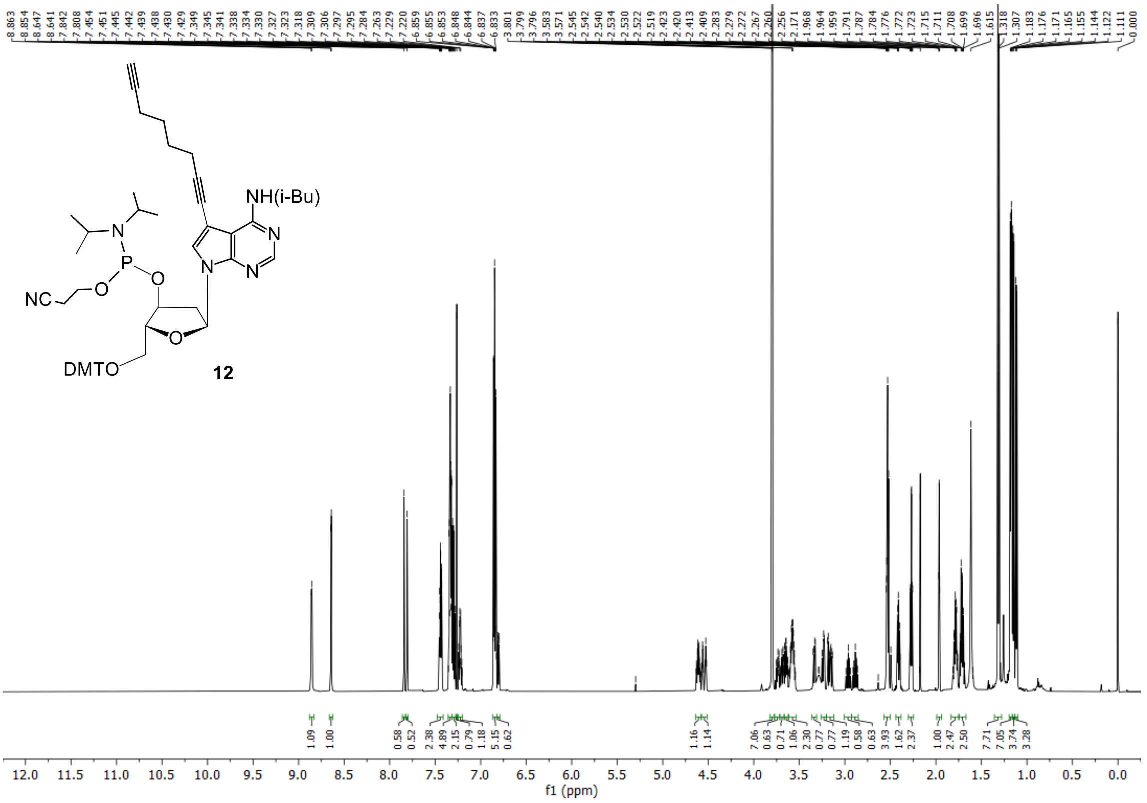

Figure S51. ^{1}H NMR ($CDCl_3$, 600 MHz) spectrum of compound **12**.

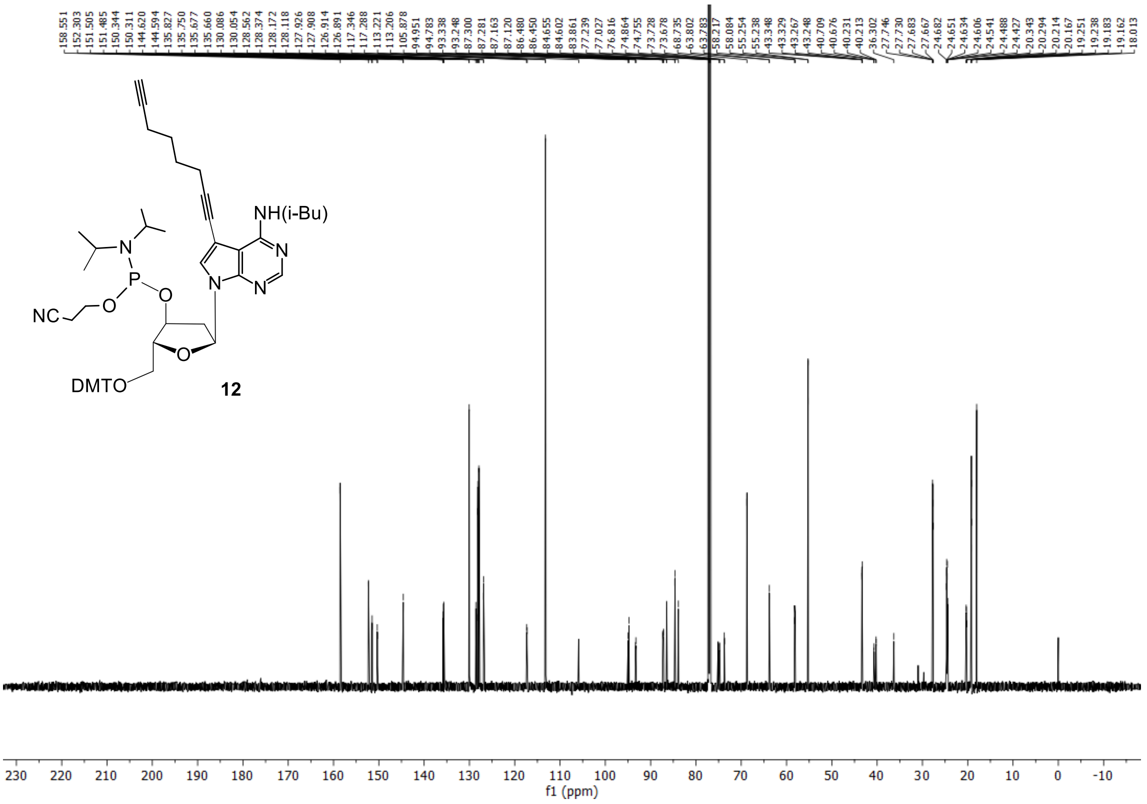

Figure S52. ^{13}C NMR ($CDCl_3$, 150 MHz) spectrum of compound **12**.

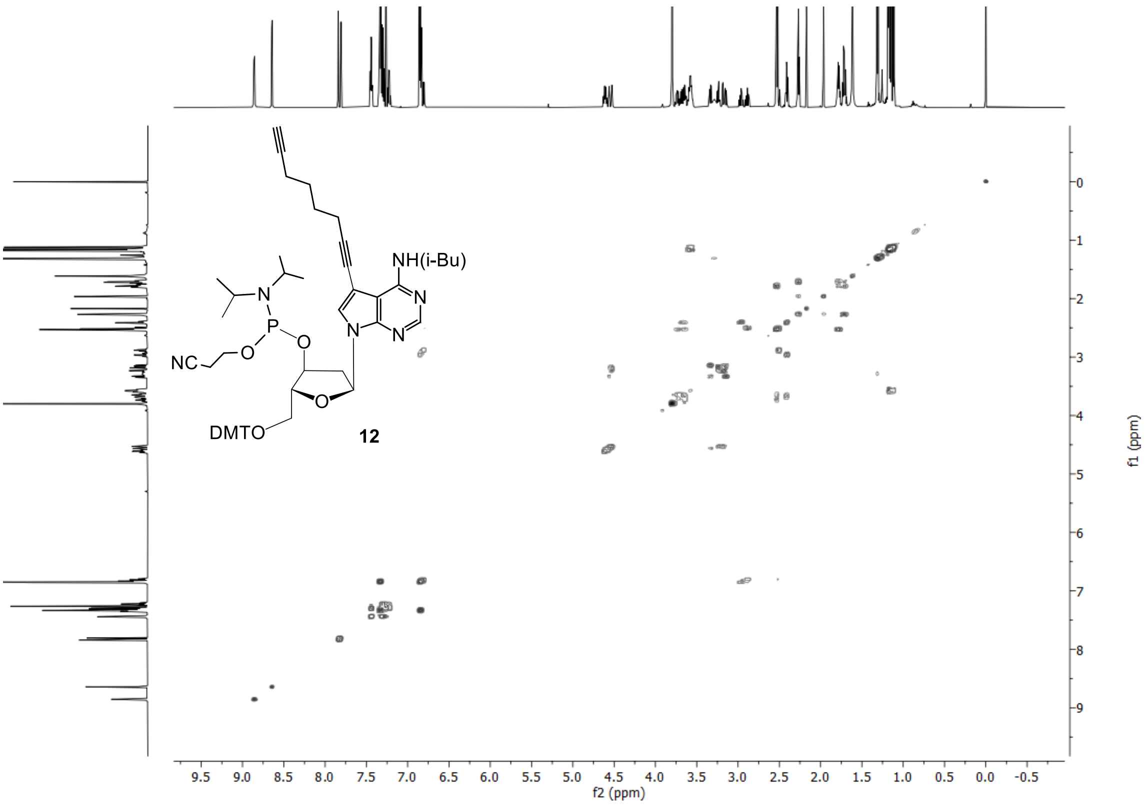

Figure S53. COSY NMR ($CDCl_3$, 600 MHz) spectrum of compound **12**.

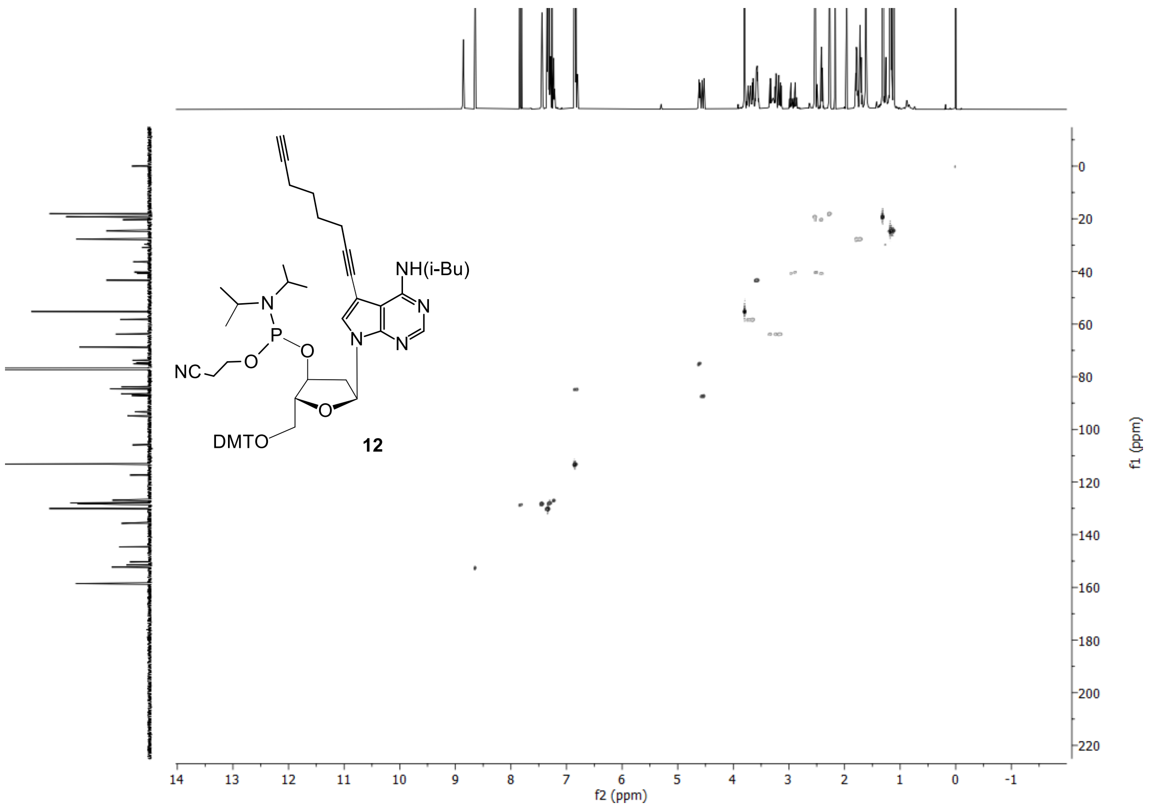

Figure S54. HSQC NMR ($CDCl_3$, 600 MHz) spectrum of compound **12**.

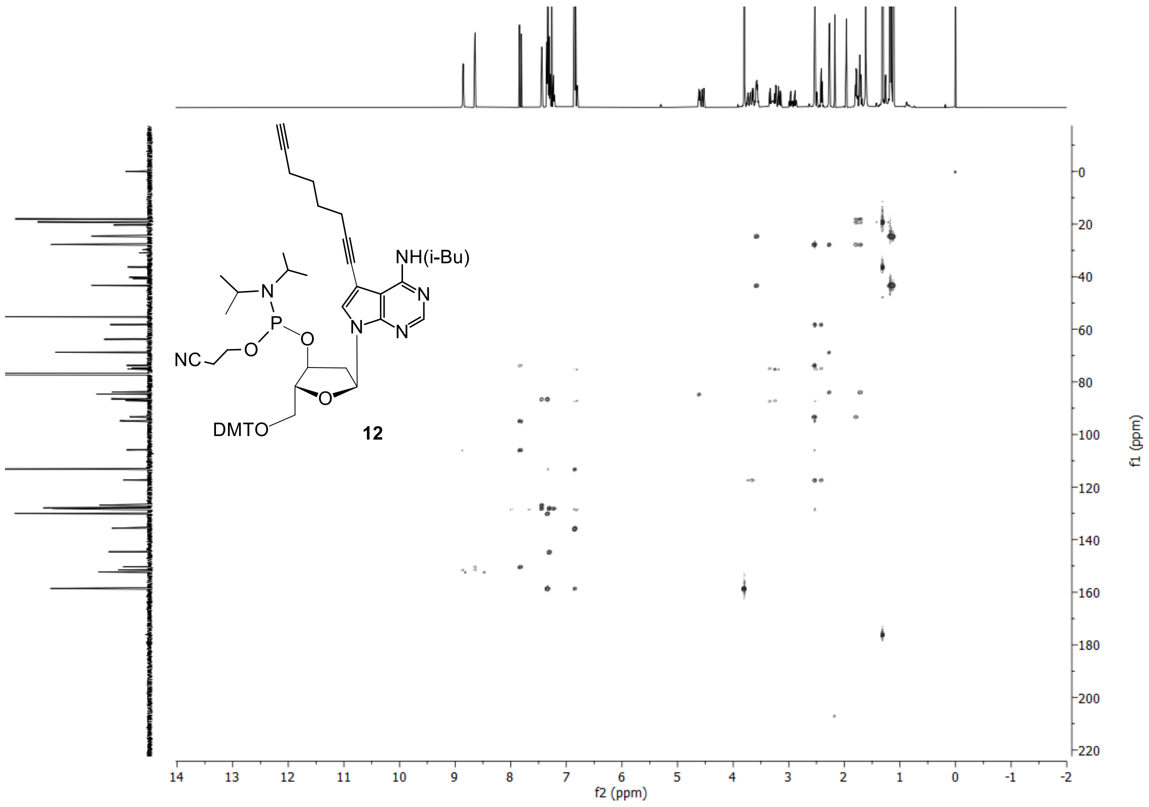

Figure S55. HMBC NMR ($CDCl_3$, 600 MHz) spectrum of compound **12**.

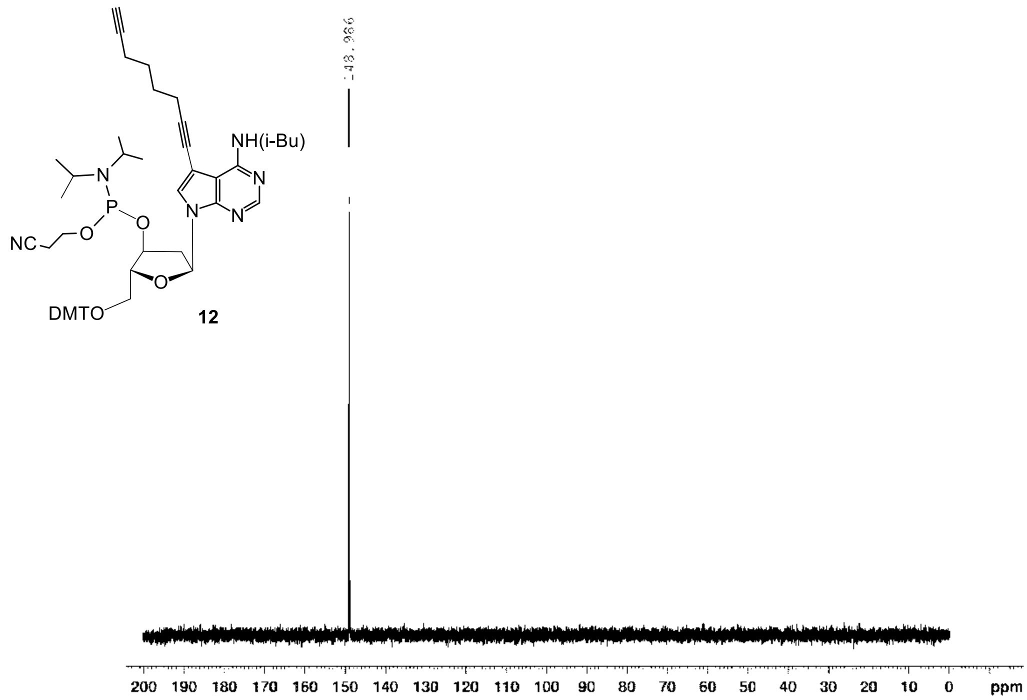

Figure S56. ^{31}P NMR ($CDCl_3$, 121 MHz) spectrum of compound **12**.
